# Mitochondrial genome comparison reveals the evolution of cnidarians

**DOI:** 10.1002/ece3.10157

**Published:** 2023-06-13

**Authors:** Hui Feng, Sitong Lv, Rong Li, Jing Shi, Jianxing Wang, Pinglin Cao

**Affiliations:** ^1^ Marine Microorganism Ecological & Application Lab Zhejiang Ocean University Zhoushan China; ^2^ Graduate School of Life Sciences Tohoku University Sendai Japan

**Keywords:** Cnidaria, evolution, gene order, mtDNA, phylogeny

## Abstract

Cnidarians are the most primitive metazoans, but their evolutionary relationships are poorly understood, although recent studies present several phylogenetic hypotheses. Here, we collected 266 complete cnidarian mitochondrial genomes and re‐evaluated the phylogenetic relationships between the major lineages. We described the gene rearrangement patterns of Cnidaria. Anthozoans had significantly greater mitochondrial genome size and lower A + T content than medusozoans. Most of the protein‐coding genes in anthozoans such as *COX 13*, *ATP6*, and *CYTB* displayed a faster rate of evolution based on selection analysis. There were 19 distinct patterns of mitochondrial gene order, including 16 unique gene orders in anthozoans and 3 mtDNA gene orders pattern in medusozoans, were identified among cnidarians. The gene order arrangement suggested that a linearized mtDNA structure may be more conducive to Medusozoan mtDNA stability. Based on phylogenetic analyses, the monophyly of the Anthozoa was strongly supported compared to previous mitochondrial genome‐based analyses rather than octocorals forming a sister group relationship with medusozoans. In addition, Staurozoa were more closely related to Anthozoa than to Medusozoa. In conclusion, these results largely support the traditional phylogenetic view of the relationships of cnidarians and provide new insights into the evolutionary processes for studying the most ancient animal radiations.

## Graphical abstract

First of all, we used mt‐genomes as many as possible published before to analyze the evolutionary relationship. Secondly, more recent studies based on mitochondrial whole‐genome sequences have suggested that Anthozoa are polyphyletic, with the subclasses Octocorallia being a sister group with Medusozoa. But we strongly supported that Anthozoa is monophyly by both Bayesian and GTR‐based maximum likelihood analyses. Finally, Staurozoa has been hypothesized to be closely related to sea anemones but we supported it was closed to coral animals.

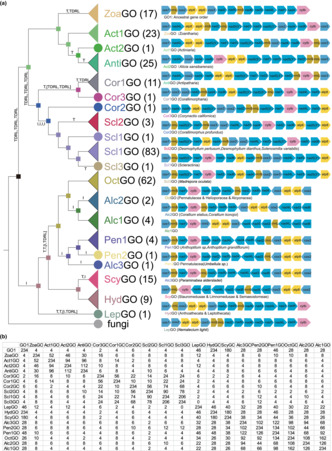

## 1 INTRODUCTION

The Cnidaria are one of the most primitive groups of multicellular animals, comprising corals, sea anemones, hydroids, and jellyfish, and they occupy an important place in modern aquatic ecosystems. Located at the base of the ‘genealogical evolutionary tree’ of the Earth's fauna, cnidarians are the most primitive metazoans and are of great importance to the study of the origin and evolution of higher animal groups. However, little is known about their early evolutionary history.

**FIGURE 1:**
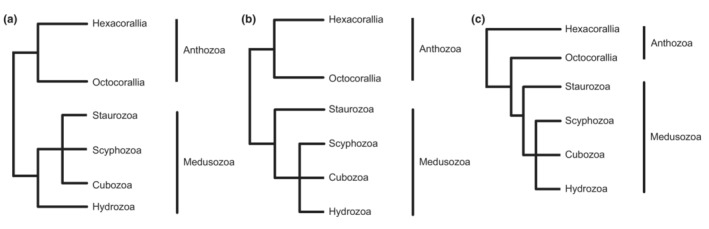
Phylogenetic hypothesis of Cnidaria.

Recent phylogenetic analyses have supported the monophyletic nature of cnidarians and the status of cnidarians as a sister group to bilaterally symmetric animals (Zapata et al., 2015), but the exact relationships between the different cnidarian taxa are unclear. In most theories of the evolution of the phylum Cnidaria, radial symmetry and the level of organization are seen as evidence that the group is primitive (i.e., that it evolved before bilateral symmetry evolved) and that jellyfish are primitive body forms reflecting the sexual reproductive stage of the life cycle. Another theory is that the original cnidarian was a flattened organism that preceded the hydroids and jellyfish. There has been disagreement within the ancient cnidarians, and some Cambrian fossil representatives of the major cnidarian lineages are very similar to extant forms (Marques & Collins, 2004). The presence of these Cambrian fossils suggests that multiple extant clades of cnidarians already existed over 500 million years ago (Bridge et al., 1995). The likely relatively rapid divergence times within the cnidarians combined with widespread extinctions (Park et al., 2011) make it difficult to reconstruct higher‐level phylogenetic relationships within the group. The cnidarians were formerly grouped with the ctenophores in the phylum Coelenterata, but with increasing awareness of the differences between them these were separated into different phyla (Dunn et al., 2015). One view that has now received widespread support from anatomy, life history, genome structure, and DNA sequences is that cnidarians consist of two relatively independent branches (Anthozoa and Medusozoa) (Figure 1a).

The Medusozoa are usually divided into four groups, Scyphozoa, Cubozoa, Staurozoa, and Hydrozoa (Daly et al., 2007). Although Medusozoan are generally considered to be characterized by the presence of a free‐swimming jellyfish stage, this is far from universal in this group (Collins et al., 2006; McFadden et al., 2010). Instead, all medusozoans have a linear mitochondrial DNA genome (Bridge et al., 1992, 1995) and a hinged cap called an operculum at the apex of their nematocysts (Reft & Daly, 2012). These synapomorphies are consistent with the monophyly of Medusozoa recovered from molecular phylogenetic studies using nuclear ribosomal DNA sequences (Cartwright & Collins, 2007; Collins, 2002; Collins et al., 2006). In medusozoans, symmetry is quite varied. Different species exhibit bilateral or radial symmetry, and some even show directional asymmetry (Dunn, 2005; Hyman, 1940; Manuel, 2009). Staurozoa comprises about 50 species, and because their benthic polyp forms also exhibit features known in the jellyfish stages of cubozoans and scyphozoans such as gastric filaments, coronal muscles, and structures from the primary tentacles of the polyp (rhopalioids/rhopalia), they have therefore long been difficult and confusing for cnidarian systematists (Zapata et al., 2015). Maximum‐likelihood analysis of nuclear ribosome sequences recovered Staurozoa as a sister taxon to other Medusozoa as well as the monophyletic Cubozoa and Scyphozoa as sister groups to Hydrozoa (Cartwright & Collins, 2007; Collins et al., 2006) (Figure 1b). These results contradict an analysis of protein‐coding mitochondrial gene sequences that indicated a paraphyletic Scyphozoa, Staurozoa, and Cubozoa evolutionary branch as a sister taxon to Hydrozoa (Kayal et al., 2013). In an analysis of morphological data, Marques and Collins (Marques & Collins, 2004) reported Cubozoa and Staurozoa as sister groups to Scyphozoa, while the analysis of a corrected version of the same dataset was consistent with results derived from nuclear ribosomal sequences (Van Iten et al., 2006). Resolving the relationships between these lineages is important to our understanding of key innovations within the Medusozoa, including the origin of pelagic medusozoans and associated sensory structures and swimming muscle organization as well as patterns of medusozoan metamorphosis and development.

Anthozoa is a taxonomically well‐defined group whose monophyly is supported by extensive analysis of rRNA data. However, studies by Kayal et al. (2013) and Park et al. (2012) based on mitochondrial genome sequences have shown that Anthozoa is paraphyletic, with Octocorallia being a sister taxon to Medusozoa (Figure 1c). This differs from the traditional classification, but their results were supported by a high degree of statistical significance in the maximum likelihood framework. Such a result of paraphyly has been noted in previous studies (Lavrov et al., 2008; Shao et al., 2006) that also used mitochondrial genomic data. Such differences in phylogenetic relationships may be caused by long branching attractions in molecular data (Bergsten, 2005; Brinkmann et al., 2005), heterogeneous nucleotide substitution rates between different lineages (Baurain et al., 2007), compositional heterogeneity (Foster, 2004; Foster & Hickey, 1999; Jermiin et al., 2004), generation time effects (Thomas et al., 2010), incomplete sampling of taxonomic units (Rannala et al., 1998; Rosenberg & Kumar, 2001; Zwickl & Hillis, 2002) or a combination of these factors.

Mitochondrial DNA (mtDNA) is a common molecular marker used to determine the phylogenetic relationships among animals. Recent technological advances in whole‐genome sequencing have provided easier access to mitochondrial genomic data for phylogenomic studies (Kan et al., 2010; Podsiadlowski et al., 2009; Rota‐Stabelli et al., 2010). Some advantages of mtDNA over nuclear DNA (nDNA) in phylogenetics are the asserted homology of all genes (Hoelzer, 1997) and the relatively conserved small genome structure that provides some additional features such as gene order (considered rare genetic variation or RGC) (Boore & Fuerstenberg, 2008; Lavrov et al., 2005). Despite some limitations, mtDNA‐based phylogenetic trees are considered to be useful tools for the evolutionary history within and between most groups of metazoans. However, the small sample sizes of cnidarian taxa in previous studies have raised some questions concerning the validity of phylogenetic results. It is well‐known that undersampling of taxonomic units and systematic errors can override the true phylogenetic signal, resulting in flawed phylogenetic reconstructions (Pick et al., 2010).

Here, we presented a comprehensive analysis of the phylogeny of Cnidaria based on 266 complete mitochondrial genomes, including a phylogenetically diverse set of species representing each of the four medusozoan orders Anthoathecata, Leptothecata, Rhizostomeae, and Semaeostomeae. All sequences were re‐annotated to resolve potential annotation errors or inconsistencies in published mitochondrial genomes. Phylogenetic reconstruction of the maximum likelihood framework was based on comparative data of protein‐coding genes. This study presents the largest number of mitogenomes and provides an in‐depth exploration of the phylogenetic relationships among the Cnidaria.

## 2 MATERIALS AND METHODS

### 2.1 Data acquisition and mitochondrial genome characterization

We retrieved 48,966 mitochondrial genome sequences of all available cnidarians from the NCBI database, then preferentially selected reference sequences when species were represented by multiple mitochondrial genomes and filtered the partial genomes, resulting in a dataset of 266 genomic sequences. These included four of the six classes of cnidarians (Anthozoa, Hydrozoa, Scyphozoa, and Staurozoa). Cubozoa, Myxozoa, and *Hydra magnipapillata* were not included in our analysis because the mitochondrial genomes of Cubozoa and *H. magnipapillata* were distributed over multiple chromosomes (Smith et al., 2012; Voigt et al., 2008), while incomplete annotation results were obtained for the mitochondrial genome of Myxozoa (Takeuchi et al., 2015). To avoid potential inconsistencies or errors in the published sequence annotations, we re‐annotated all sequences using GeSeq (Tillich et al., 2017).

Based on the invertebrate mitochondrial genetic code, the nucleotide composition was calculated in MEGA X (Kumar et al., 2018). AT‐skew and GC‐skew for the complete mitotic genome were determined according to the following equations: AT‐skew = (A − T)/(A + T) and GC‐skew = (G − C)/(G + C) (Perna & Kocher, 1995).

### 2.2 Sequence alignment

We aligned the nucleotide sequence and amino acid sequence of each protein‐coding gene separately using the default parameter values of MAFFT v7.313 (Katoh & Standley, 2013). Poorly aligned positions were eliminated using Gblocks 0.91b (Castresana, 2000) under default conditions. The individually aligned sequences were then concatenated. To explore the diverse patterns of mtDNA protein‐coding genes in cnidarians, we estimated the number of polymorphic sites (S) and nucleotide diversity (π) for 13 protein‐coding genes using DNAsp v. 6.12.03 (Rozas et al., 2017).

### 2.3 Selection analysis

To test whether the ⍵ ratios (dN/dS, where dN is the non‐synonymous substitution rate and dS is the synonymous substitution) differed between anthozoans and medusozoans, we used the codeML program of PAML v 4.9 software (Yang, 2007), employing a branch model to detect each mitochondrial protein‐coding gene in 266 species. We first estimated unique ω values for all branches in the tree using the one‐ratio model (model = 0). Then, using the free‐ratios model (model = 1), we assumed that each branch had an independent value of ω. Finally, we labeled the medusozoans as foreground branches and anthozoans as background branches by the algorithm, using a two‐ratio model to estimate ω for the mtDNA protein‐coding genes of each species.

### 2.4 Mitochondrial genome gene order

The orders of mitochondrial gene alignments were extracted using PhyloSuite v1.2.2 (Zhang et al., 2020), and the matching gene orders were aggregated into groups to obtain an MGO dataset. Because the number of tRNAs in the mtDNA of cnidarians varies, we only considered protein‐coding genes and rRNA genes. Inter‐genomic rearrangement analysis was performed using the common interval algorithm of CREx (Bernt et al., 2007), and ancestral traits were inferred for the gene order of cnidarians using MLGO (maximum likelihood algorithm) and TreeREx (Bernt et al., 2008) (common interval algorithm).

### 2.5 Phylogenetic reconstruction

Phylogenetic reconstruction was based on protein‐coding genes using the mitochondrial genome sequences of seven fungal species as an outgroup. A maximum likelihood tree of the nucleotide and amino acid sequence dataset was reconstructed using IQ‐TREE software (Nguyen et al., 2015) using 1000 ultrafast bootstrap replicates to assess node support, and only nodes with bootstrap values >95 were considered reliable. We also estimated Bayesian trees for the nucleotide and amino acid datasets using MRBAYES v3.2.6 (Ronquist et al., 2012) and estimated partitioning models using PARTITION FINDER 2 (Lanfear et al., 2017) based on the Bayesian Information Criterion (BIC). Markov Chain Monte Carlo (MCMC) was run with four chains for 5,000,000 generations, sampling the tree every 1000 generations to allow sufficient time for convergence. The Bayesian posterior probability was obtained from the majority rule of 50% of the trees sampled at equilibrium, with the top 25% discarded as ‘aged’. The resulting phylogenetic tree was visualized in iTOL (Letunic & Bork, 2007).

## 3 RESULTS

### 3.1 Characteristics of the mtDNA genome structure of cnidarians

**FIGURE 2:**
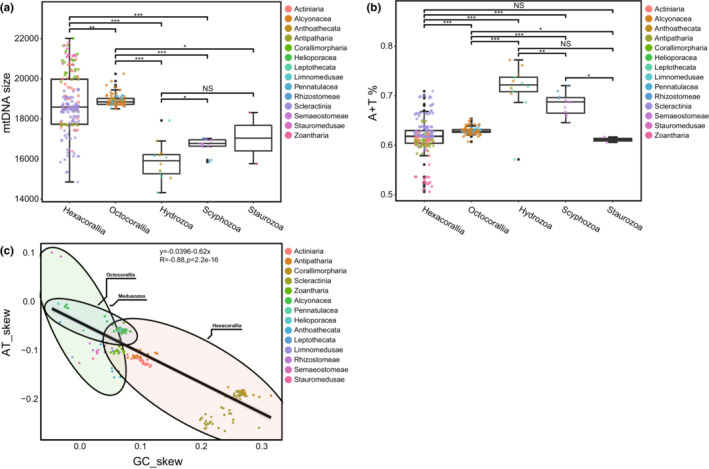
Characteristics of the mtDNA of cnidarian (a) MtDNA size of cnidarian, (b) A + T content of cnidarians, (c) AT and GC skew in cnidarian.

The 266 mitochondrial genomes contained four of the six classes, Anthozoa (241 species), Hydrozoa (12 species), Scyphozoa (11 species), and Staurozoa (2 species), with complete mitogenome sizes ranging between 14,320 and 22,015 bp (Table 1 and Table S1). In anthozoans with a high number of mitochondrial genomes, the size of mitogenomes was stable and had a low standard deviation, whereas the size of mitogenomes in medusozoans showed a high level of heterogeneity (with a high standard deviation) (Figure S1a). The sizes of mitogenomes in Hexacorallia were significantly larger than those of Octocorallia, Hydrozoa, and Scyphozoa (*p* < .01). The mitochondrial genome size of Octocorallia was significantly larger than those of Hydrozoa, Scyphozoa, and Staurozoa (*p* < .05, Figure 2a). Overall, the sizes of the mitogenomes of medusozoans were significantly smaller than those of anthozoans (*p* < .001, Figure S1b). Unlike the vertebrate mitochondrial genome that typically encoded 37 genes (including 13 protein‐coding genes (PCGs), 22 transfer RNAs (tRNAs), and two ribosomal RNA (rRNA) genes), the cnidarian mitochondrial genome encoded only 16 to 20 genes (Table 1 and Table S2), and this variation in gene number rarely involved protein‐coding or rRNA genes, primarily differences in the number of tRNA genes. The cnidarians encoded only one or two transfer RNAs (tRNA‐Met, tRNA‐Trp), whereas all species of anthozoans, octocorallians, and zoantharians encoded only tRNA‐Met, and medusozoans and the remaining anthozoans all encoded two tRNAs, with most mitochondrial tRNAs being imported from the cytoplasm (Beagley et al., 1995). In addition to the 13 protein‐coding genes found in metazoan mtDNA (*ATP6*, *ATP8*, *CYTB*, *COX13*, *NAD16*, and *NAD4L*), cnidarian mtDNA also encoded several additional protein‐coding genes (Table 1). Some of these genes were open reading frames (ORFs) with no similarity to known proteins and some proteins that can interact with DNA such as the mutS protein unique to Octocorallia, a member of the ABC ATPase superfamily that recognizes mismatched and unpaired bases in double‐stranded DNA and initiates mismatch repair.

**TABLE 1:** Taxonomic distribution, gene content, genome size, base‐composition, and additional protein of the cnidarian mtDNAs.

Class, classification			Number of mtDNA	No of gene		Length	AT%	AT_skew	GC_skew	Additional protein
Class	Subclass	Order		All	tRNA					
Anthozoa	Hexacorallia	Actiniaria	24	17.8 ± 0.9	2.0 ± 0.2	19041.7 ± 1232.7	61.1 ± 1.0	−0.12 ± 0.01	0.11 ± 0.01	HEG, ORF, ORFA, ORFB, ORFC, ORFD
		Antipatharia	25	17.1 ± 0.3	2.0 ± 0.0	18779.5 ± 1405.3	60.5 ± 1.8	−0.11 ± 0.01	0.10 ± 0.01	HEG
		Corallimorpharia	13	16.8 ± 0.4	1.8 ± 0.4	20774.8 ± 560.1	61.1 ± 0.6	−0.19 ± 0.01	0.28 ± 0.02	
		Scleractinia	88	17.3 ± 0.8	2.3 ± 1.0	18022.7 ± 1051.9	63.8 ± 2.7	−0.21 ± 0.03	0.26 ± 0.02	
		Zoantharia	17	16.0 ± 0.0	1.0 ± 0.0	20976.4 ± 703.6	53.7 ± 2.7	−0.10 ± 0.02	0.07 ± 0.01	
	Octocorallia	Alcyonacea	54	17.0 ± 0.0	1.0 ± 0.0	18947.2 ± 328.6	62.9 ± 0.8	−0.05 ± 0.02	0.06 ± 0.03	mutS
		Pennatulacea	19	17.0 ± 0.0	1.0 ± 0.0	18854.1 ± 183.1	63.0 ± 0.4	−0.05 ± 0.02	0.06 ± 0.03	
		Helioporacea	1	17.0	1.0	18957.0	63.1	−0.06	0.07	
Hydrozoa	Hydroidolina	Anthoathecata	6	17.0 ± 0.0	2.0 ± 0.0	15937.5 ± 666.0	73.7 ± 2.6	−0.12 ± 0.03	0.06 ± 0.01	
		Leptothecata	4	17.0 ± 0.0	2.0 ± 0.0	15204.8 ± 721.4	72.2 ± 1.1	−0.11 ± 0.05	0.04 ± 0.05	
	Trachylinae	Limnomedusae	2	19.0 ± 0.0	2.0 ± 0.0	17035.0 ± 1254.4	62.9 ± 8.1	−0.10 ± 0.01	0.02 ± 0.05	dnaB, orf314
Scyphozoa		Rhizostomeae	4	19.3 ± 1.7	3.8 ± 2.1	16458.5 ± 643.8	70.6 ± 1.2	−0.09 ± 0.01	0.04 ± 0.01	dnaB, orf314
		Semaeostomeae	7	18.3 ± 1.0	2.1 ± 0.4	16808.1 ± 143.6	66.9 ± 1.6	−0.05 ± 0.10	0.02 ± 0.03	
Staurozoa		Stauromedusae	2	18.5 ± 2.1	3.5 ± 2.1	17041.0 ± 1803.1	61.1 ± 0.8	−0.12 ± 0.01	0.02 ± 0.02	

Whether changes in nucleotide composition affect amino acid alignment and subsequent phylogenetic analysis is unclear, with changes in A + T content of mitogenomes from 50.61% (*Parazoanthus swiftii*) to 77.24% (*Hydra sinensis*) in cnidarians. The highest A + T content was found in Anthoathecata (mean 73.7%, SD = 2.6%) and the lowest A + T content was found in Zoantharia (mean 53.7%, SD = 2.7%) (Table 1). In contrast, the A + T content of Hexacorallia was significantly lower than those of Octocorallia, Hydrozoa, and Scyphozoa (*p* < .001), which in Octocorallia was significantly lower than in Hydrozoa (*p* < .05) and Scyphozoa, and significantly higher than in Staurozoa (*p* < .001, Figure 2b). Overall, the A + T content of mitogenomes was significantly lower in anthozoans than in medusozoans (Figure S1c, Table S2).

The A + T content and strand skew were also factors in generating differences in genomic nucleotide frequencies. GC‐skew and AT‐skew can represent differences between two strands due to asymmetries in the mitochondrial genome replication process, where one strand favors G/T over C/A. With the exception of *Aurelia aurita* and *Aurelia limbate*, the mtDNA of all cnidarians had a negative AT‐skew, and most species had a positive GC‐skew. The results indicated that cnidarians are biased towards using GT and not AC. We found a strong negative correlation (*R* = −.88, *p* < .001) between the AT and GC skew of mtDNA in cnidarians (Figure 2c).

**FIGURE 3:**
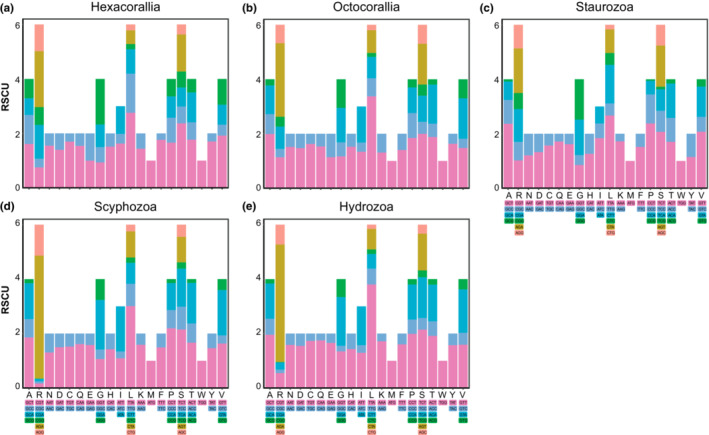
Relative synonymous codon usage in mitochondrial genome of cnidarian.

The result of the bias in nucleotide composition towards A and T was also reflected in the use of codons. Overall, the amino acids used more frequently in cnidarians were Phe (TTT), Leu (TTA), Ile (ATT), Val (GTT), Tyr (TAT), and Asn (AAT), almost all of which were composed of A and T (Figure 3). This may have played an important role in the high A + T content of cnidarian mtDNA sequences.

Estimates of genetic diversity from protein‐coding genes retrieved ATP8 as the most diverse gene among all species (π = 0.31763) followed by NADH dehydrogenase genes with *NAD2* (π = 0.31325) presenting higher diversity. The cytochrome c oxidase genes (*COX1*, *COX2*, and *COX3*) were the least diverse among the mtDNA genes (Table 2).

**TABLE 2:** Interspecific diversity for all 13 protein‐coding genes on cnidarian.

Gene	Bp	Nucleotide diversity (π)	Monomorphic sites	Polymorphic sites
ATP6	739	0.28689	113	481
ATP8	332	0.31763	8	110
COX1	2544	0.20318	328	451
COX2	1253	0.24171	151	423
COX3	820	0.24629	185	467
ND1	1229	0.24504	233	648
ND2	1786	0.31325	104	708
ND3	372	0.26017	69	233
ND4	1621	0.28574	245	970
ND4L	312	0.28169	37	205
ND5	2187	0.28913	301	1204
ND6	917	0.29096	42	294
CYTB	1732	0.26974	287	818

### 3.2 Selection analysis

**FIGURE 4:**
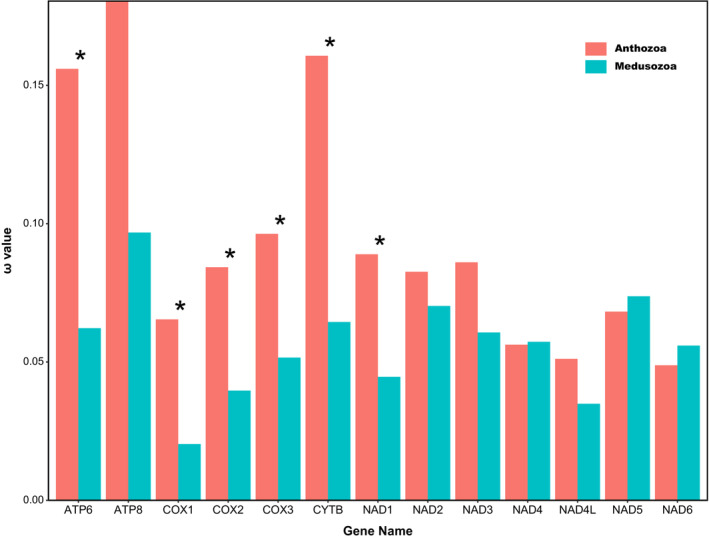
Comparisons of ω values of mtDNA among the anthozoans and medusozoans base on 13 mtDNA PCGs (*p < .05).

For all 13 protein‐coding genes, branch model analysis identified 10 rapidly evolving genes in anthozoans relative to medusozoans, but the two‐ratio model used to calculate selection pressure was better suited (*p* < .05) for the genes as follows: *ATP6* (anthozoans: 0.015596, medusozoans: 0.06222), *COX1* (anthozoans: 0.0654, medusozoans: 0.02034), *COX2* (anthozoans: 0.08426, medusozoans: 0.03966), *COX3* (anthozoans: 0.09632, medusozoans: 0.0516), *CYTB* (anthozoans: 0.16069, medusozoans: 0.06447), *NAD1* (anthozoans: 0.08895, medusozoans: 0.04464). Furthermore, *ATP8* showed the highest ω values for this gene in both the foreground and background branches compared to the other genes. However, LRT was unable to indicate the two‐ratio model as the most appropriate to explain the difference in ω values for this gene (*p* = .152) (Figure 4).

**FIGURE 5:**
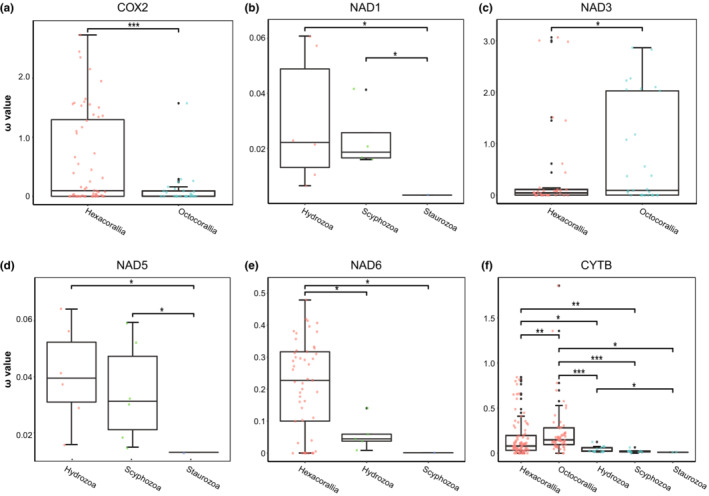
Comparisons of ω values among Cnidaria of different classes based on 13 mtDNA PCGs. The other pairs showed no significant differences. (*p < .05; **p < .01; ***p < .001).

The results of the free‐ratio model showed that the ω values of *COX2* were significantly higher in the hexacorallians than octocorallians (Figure 5a), while the ω values of *NAD3* were significantly lower than in octocorallians (Figure 5c); the ω values of *NAD6* were significantly higher in hexacorallians than in hydrozoans and staurozoans (Figure 5e). The ω values of *NAD1* and *NAD5* in hydrozoans were significantly higher than in scyphozoans and staurozoans (Figure 5b, d), and the ω values of *NAD6* were significantly higher than in scyphozoans (Figure 5e). The ω values of *NAD1*, *NAD5*, and *NAD6* in scyphozoans were significantly higher than in staurozoans (Figure 5b, d, e). The Wilcoxon test results for other genes were not significant (*p* > .05) (Figure S2).

### 3.3 Mitogenome‐based phylogeny of cnidarians

**FIGURE 6:**
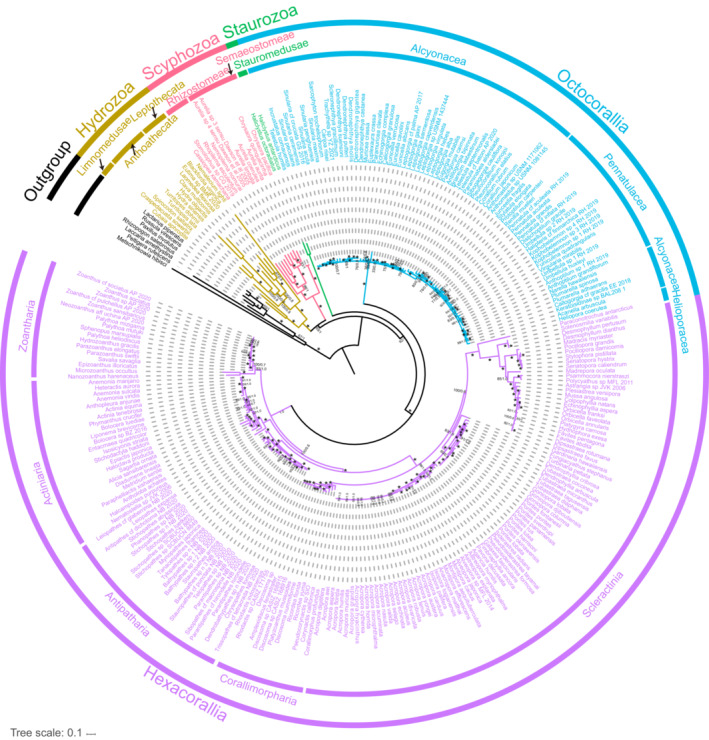
The phylogenetic tree of Cnidaria derived from Bayesian inference and Maximum Likelihood analyses using 13 PCGs. The numbers at each node are bootstrap values (BS) for ML and Bayesian posterior probability (BPP) for BI. Stars denote maximum support values.

In previous studies, the small sample size of cnidarian taxa has raised some questions concerning the validity of the phylogenetic results. Here, we reconstructed the phylogenetic relationships of cnidarians using all available complete cnidarian mitochondrial genomes, with seven fungal species as outgroups. The ML phylogeny was similar to the results obtained with Bayesian trees (Figure 6). However, the developmental tree obtained based on the nucleotides of 13 protein‐coding genes strongly supported monophyly at the level of class in cnidarians. There has been good molecular and morphological support for the sister taxon results for medusozoans and anthozoans, and our results strongly supported the monophyly of the branch Anthozoa [Hexacorallia + Octocorallia] (BS = 100, BPP = 1.0), while Staurozoa was shown to be more closely related to the Anthozoa clade and was also strongly supported (BS = 92, BPP = 1).

Among the anthozoans, the monophyly of Actiniaria, Antipatharia, Zoantharia, and Corallimorpharia of the subclass Hexacorallia was strongly supported (BS > 90, BPP > 0.9), but the orders Corallimorpharia and Scleractinia in the ML results were combined as paraphyletic groups, while in the BI results, they were separated (Figure S3). The relationship between the order Corallimorpharia and Scleractinia has been controversial in previous studies; the former was either the closest outgroup to Scleractinia or was formed from the organisms of Scleractinia through skeletal loss. Our results for paraphyletic groups in ML did not receive high support values (BS < 90), but the sister taxon relationship between Scleractinia and Corallimorpharia was strongly supported in the BI tree results (BPP = 1.00) (Figure S3). The internal relationships of Octocorallia were not resolved, and our results only restored the monophyly of Pennatulacea, although it formed a paraphyletic group of Octocorallia with Helioporacea and Alcyonacea.

Among the medusozoans, Staurozoa was clearly more closely related to the Anthozoa clade in our results. We similarly recovered the monophyly of two other classes, Hydrozoa and Scyphozoa with maximum support, and within Scyphozoa, our data rejected the monophyly of Semaeostomeae and Rhizostomeae, where Cassiopea as a sister taxonomic unit to the remaining Scyphozoa species, and uniting the others. In Hydrozoa, Limnomedusae as the sole representative of Trachylina constituted a sister taxon relationship with Hydroidolina and received maximum support (BS = 100, BPP = 1.0), however, the relationship within Hydroidolina was not resolved.

### 3.4 The gene order of the mtDNA of cnidarians

**FIGURE 7:**
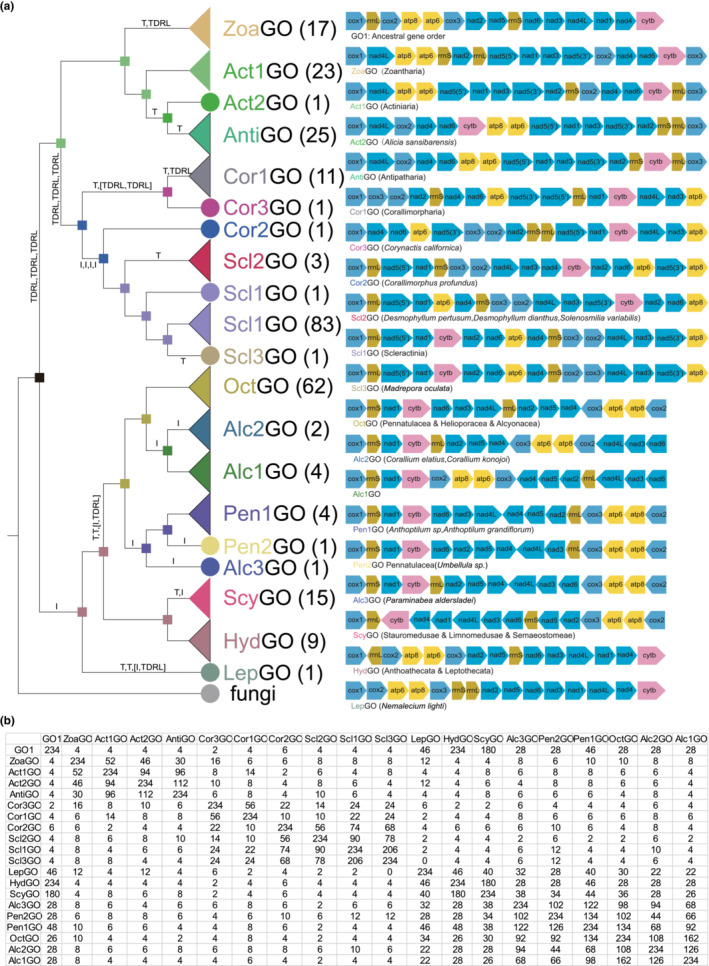
The gene order of cnidarian mitogenomes. (a) ML tree based on gene order (left) and the gene order of Cnidaria (right) (TDRL: the tandem duplication and random loss, T: transposition, I: inversion); (b) The number of common intervals of different gene orders.

A total of 19 different ancestral gene orders were identified in 266 species of cnidarians (Figure 7). GO1 was predicted to be the ancestral gene order of cnidarians. A comparison of the mitochondrial genomes of species of the subclass Hexacorallia showed that all five orders had different gene orders; a unique feature was the presence of a self‐snipping intron in *NAD5* that contained many complete genes, and that the intron contained different numbers of genes in the different orders in Hexacorallia. In the data obtained, the *NAD5* intron contained only two genes, *NAD1* and *NAD3*, in the mtDNA of species of Actiniaria, Antipatharia, and Zoanthidea, but in Corallimorpharia the number of genes contained in the *NAD5* intron varied considerably between species of different genetic order. In the species represented by Cor1GO, all genes were contained in the *NAD5* intron; in Cor2GO, 11 genes were contained in the *NAD5* intron, while in Cor3GO, the number of genes was reduced to nine. The same occurs in Scleractinia, where the *NAD5* introns of Scl1GO and Scl3GO contained the same 11 genes as Cor2GO, and the number of genes in the Scl1GO intron was reduced to eight (Figure 7).

Among the Octocorallia organisms, 83.78% of the species (Alcyonacea (47/54), Pennatulacea (14/19), and Helioporacea (1/1)) shared the OctGO pattern. In contrast, the other Octocorallia mtDNA gene sequences involved the reversal or transposition of only a few gene blocks, including *NAD6*‐*NAD3*‐*NAD4L*, *rrnL*‐*NAD2*‐*NAD5*‐*NAD4*, and *COX2*‐*ATP8*‐*ATP6*‐*COX3* (Figure 7).

In Medusozoa, three gene orders were found (HydGO, ScyGO, LepGO), with LepGO found only in the species *Nemalecium lighti* and with low similarity to the other two gene orders (46 and 40, respectively) (Figure 7). In addition, HydGO was found in Anthoathecata and Leptothecata species and ScyGO in Limnomedusae, Rhizostomeae, Semaeostomeae, and Stauromedusae. The similarity between them was high, with gene rearrangements involving only two gene blocks, *rrnL*, and *COX2*‐*ATP8*‐*ATP6*‐*COX3*‐*NAD2*‐*NAD5*‐*rrnS*‐*NAD6*‐*NAD3*‐*NAD4L*‐*NAD1*‐*NAD4*‐*CYTB* reversal.

The results from a Maximum Likelihood evolutionary tree based on gene order composition and inference of ancestral characters showed that with the exception of the unusual *Nemalecium lighti*, the rest of the medusozoans and Octocorallians form a sister taxon, with the Hexacorallia and Octocorallia of Anthozoa forming a paraphyletic group. Among the Hexacorallia, Scleractinia, and Corallimorpharia were more closely related to each other than to the remaining taxa. Ancestral gene order inference suggested that the GO1 pattern was the ancestral gene order of the cnidarians. The evolution of the other mitochondrial gene orders by mechanistic analysis of TreeREx could be summarized as follows: (1) GO1 was derived as the ancestral gene order of the Hexacorallia branch through three TDRL events, followed by a transposition event and a TDRL event to form the ancestral gene order of Zoantharia, or through three TDRL events to form Scleractinia and Corallimorpharia; (2) GO1 was derived via an inversion event as the ancestral gene order common to Medusozoa and Octocorallia.

## 4 DISCUSSION

### 4.1 The phylogeny of cnidarians

In phylogenetic analyses of cnidarians, it has been common for Anthozoa to be placed as a sister taxon to Medusozoa, a hypothesis supported by morphology and rDNA sequences (Collins, 2002; Daly et al., 2007). However, more recent studies based on mitochondrial whole‐genome sequences have suggested that Anthozoa are polyphyletic, with the subclasses Octocorallia being a sister group with Medusozoa (Kayal et al., 2013; Lavrov et al., 2008; Park et al., 2012). Earlier it was thought that this was the result of limitations in taxon sampling, as it also occurred in earlier rDNA analyses (Bridge et al., 1995). Our results again demonstrated that the Anthozoa being identified as a polyphyletic group may be due to the limitations in taxon sampling, and both Bayesian and GTR‐based maximum likelihood analyses strongly supported the monophyly of Anthozoa. More recent studies have suggested that the close association between Octocorallia and Medusozoa is likely due to the use of Porifera as roots for Cnidaria, with each of the three taxa forming a separate and well‐supported branch in analyses that included only Anthozoa and Medusozoa (Figueroa & Baco, 2015). The choice of an appropriate outgroup was extremely important in phylogenetic studies, the difference between the nuclear and mitochondrial genomes may be due to the incorrect choice of Porifera as an outgroup, and we also reconstructed the phylogenetic tree using the Bilateria as outgroup which was the sister taxon of Cnidaria (Augustin et al., 2010; Dunn et al., 2014; Wallberg et al., 2004), with results similar to those of fungi as outgroup (Figure S4). It showed that it's inappropriate that choice Porifera as an outgroup to explore the phylogenetic relationship of Cnidaria. Staurozoa was probably the earliest divergent lineage in a taxon traditionally considered to be part of Medusozoa (Collins, 2009) and was probably the sister taxon to all other Medusozoa (Daly et al., 2007). However, unlike previous results based on either nuclear rRNA or mitochondrial protein‐coding genes, our results showed that Staurozoa is a sister taxon to Anthozoa (BS = 92, BPP = 1.0). Staurozoa was a group of benthic cnidarians, the so‐called stemmed jellyfish, that currently consists of only about 50 species (Miranda et al., 2016, 2017). The group has a long taxonomic history and was once referred to as a ‘puzzling group’ (Gwilliam, 1956), having been hypothesized to be closely related to sea anemones (Cuvier, 1829). Staurozoa species in our analysis were also closer to anthozoans in terms of codon usage preference than other species, suggesting a closer relationship between Staurozoa and Anthozoa. This result may be due to the fact that members of fungi were included in Cnidaria in our phylogenetic reconstruction, and the resulting unrooted phylogeny suggests that fungi are sister branches of Medusozoa. If the tree was redrawn and rooted by fungi, then the resulting phylogeny appears as if the Medusozoa with the exception of Staurozoa were the basal branches of Cnidaria, while the Anthozoa branch slightly later and formed a sister clade with Staurozoa.

Our BI analysis restored the currently generally accepted phylogenetic relationships of the six putative Anthozoa subclasses (Kitahara et al., 2010), i.e. Zoantharia was the earliest divergent lineage, followed by Actiniaria, Antipatharia, Corallimorpharia, and Scleractinia. However, in the ML analysis, Actiniaria replaced Zoantharia as the earliest divergent lineage. The monophyly of Scleractinia has been questioned in analyses based on mitochondrial genomic data (Medina et al., 2006), but more thorough studies using alternative datasets have rejected this hypothesis (Budd et al., 2010; Fukami et al., 2008). The monophyly of stony corals was also strongly supported in our Bayesian‐based analysis (BB = 1.0), but Scleractinia were paraphyletic in the GTR‐based maximum likelihood analysis, possibly because the main assumption behind the GTR model, namely homogeneity across loci substitution patterns, was violated by most molecular data, making it more difficult to correctly capture the phylogenetic signal present in our comparisons (Lartillot et al., 2007).

Among the medusozoans, Scyphozoa, Staurozoa, and Hydrozoa included in the dataset were all strongly supported as monophyletic. Although Staurozoa has always been classified into Medusozoa, Staurozoa was much closer to the Anthozoa clade in our phylogenetic tree, and the results for [Staurozoa + Anthozoa] were strongly supported by BI and ML (BS = 92, BPP = 1.0). The key feature that distinguishes anthozoans from medusozoans is the presence or absence of the jellyfish stage (Khalturin et al., 2019), where jellyfish‐specific organs and tissues are not present in the polyp stage, and the genes and transcription factors required for their development are activated only during the polyp‐to‐jellyfish transition and in the adult (Brekhman et al., 2015; Fuchs et al., 2014; Khalturin et al., 2019). In contrast, Staurozoa settle as juveniles and develop into juvenile polyps that then exhibit many features of adult medusozoans, similar to scyphozoans and cubozoans in the structure of the oral end of the polyp during metamorphosis such as hollow structures of tentacle origin (rhopalioids/rhopalia), rounded corpuscles, gonads, and eye spots (Kikinger & von Salvini‐Plawen, 1995; Uchida, 1929). However, the ventral portion of the adult retains polyp features and does not give rise to free‐living jellyfish (Collins, 2002). Also, studies of their ovary and ocelli ultrastructures have shown starozoans to be very different from other scyphozoans (Blumer et al., 1995; Eckelbarger & Larson, 1993). This implies that Staurozoa occupies a key position in the transition between Anthozoa and Medusozoa.

### 4.2 Evolution of the mitochondrial gene order in cnidarians

Gene rearrangements within the mitochondrial genome are present in many taxa such as Ctenophora (Rosengarten et al., 2008), Anomura (Tan et al., 2018), and Brachyura (Tan et al., 2018), and for these lineages and taxa, gene rearrangements can also be used as molecular markers to support phylogenetic hypotheses (Boore, 1999; Boore & Brown, 1998; Ren et al., 2010; Wang et al., 2020). The structure of mtDNA in different taxa of cnidarians is also variable; in Anthozoa, the mtDNA is a single ring‐like molecule, whereas Medusozoan mtDNA is composed of one or more purely linear molecules (Bridge et al., 1992; Ender & Schierwater, 2003). Linear mtDNA molecules pose some difficulties for polymerase chain reaction amplification and sequencing, and this may also be one of the reasons why Medusozoa have far fewer mtDNAs than Anthozoa. Among the anthozoans, a unique feature of the Hexacorallia mtDNA was the presence of a self‐splicing intron in *NAD5* that contains many intact genes.

A total of 19 different ancestral gene orders were identified in the mitochondrial genome dataset we obtained for cnidarians. In the phylogenetic tree constructed based on gene order, cnidarians are clearly divided into two branches, Hexacorallia and [Octocorallia + Medusozoa], due to the uniqueness of the *NAD5* intron in the mtDNA of Hexacorallia. Mechanistic analysis by TreeREx revealed that most of the different ancestral gene sequences of mtDNA in cnidarians can be obtained through evolutionary rearrangement mechanisms. The GO1 order was predicted to be the ancestral gene order of all Hexacorallians, and that this evolved from the predicted ancestral order through three TDRL events; a series of simple rearrangement mechanisms then yielded the gene order of Zoantharia, Actiniaria, and Antipatharia. Unlike [Zoantharia + Actiniaria + Antipatharia], the [Corallimorpharia + Scleractinia] group has a rich and complex rearrangement pattern of the gene order, consistent with previous findings that the gene order of Corallimorpharia evolved from Cor2GO and that of Scleractinia evolved from Scl1GO (Lin et al., 2014). *Corallimorphus profundus* is more similar in genetic organization to Scleractinia than to other Corallimorpharia. Previous studies (Cairns, 1989; de Hartog, 1980; Fautin & Lowenstein, 1994; Owens, 1984) have also pointed out the degree of similarity between Corallimorphus and members of the Scleractinia characterized by reduced skeletons and fleshy polyps completely covering the underlying coral. This means that Corallimorphus occupies a key position in the Corallimorpharian and Scleractinian transitions.

The rearrangement mechanism within [Octocorallia + Medusozoa] was relatively simple, and involved only a few TDRLs and some inversion and transposition events. The rearrangements between the mtDNA of Octocorallia were relatively conserved, and as with previous findings, OctGO is the ancestral order for all Octocorallia (Figueroa & Baco, 2015). According to the rearrangement mechanism analysis, the gene order of Anthoptilum represented by Pen1GO was obtained by the inversion of the rrnL‐*NAD2*‐*NAD5*‐*NAD4* gene block of OctGO followed by the inversion of the gene block *NAD6*‐*NAD3*‐*NAD4L*‐*NAD4*‐*NAD5*‐*NAD2* into the Pen2GO pattern of *Umbellula* sp. The rearrangement of gene block *COX2*‐*ATP8*‐*ATP6*‐*COX3*‐*NAD4*‐*NAD5*‐*NAD2*‐*rrnL*‐*NAD4L*‐*NAD3*‐*NAD6* occurred in the evolution of Alc1GO and Alc2GO. All mtDNA of Octocorallia contain homologues of the *E. coli mutS* gene, and animal *MSH1* may be involved in mitochondrial DNA repair based on its direct homology to yeast‐*MSH1*, conserved protein structural domain structure, and inferred mitochondrial localization (Muthye & Lavrov, 2021). It has been shown that the sequence and structural domain composition of the Octocorallia mitochondrial *MutS* gene is very conserved and has conserved protein structural domain content as well as retaining amino acid residues that are functionally important for mismatch identification (Bilewitch & Degnan, 2011; Ogata et al., 2011). All of these results indirectly indicated the repair function of *mutS* in the mtDNA of the Octocorallia, thus making the evolutionary process of the gene sequence and structure of the Octocorallia mtDNA simpler and more conservative compared with other cnidarians.

The mtDNA of Medusozoa had a unique feature relative to Anthozoa in that all medusozoans have linear mtDNA. It has been shown that there is no clear phylogenetic signal for the distribution of linear and circular mtDNA (Nosek et al., 1998), but in animals, strictly linear mtDNA has only been found in Medusozoa (Kayal et al., 2012). Only three different gene orders were found in the mtDNA of Medusozoa. With the exception of the LepGO pattern represented only by *Nemalecium lighti*, the remaining 24 medusozoans shared two gene arrangement patterns (HydGO and ScyGO), with high similarity between them (number of common intervals = 180, maximum = 234) and only one transposition and inversion event were required for the transformation between arrangements. Hydrozoa's Hydroidolina animals shared the HydGO mode, and the rest of the Medusozoa shared the ScyGO mode. The differences in mtDNA gene order in Medusozoa were smaller and the variation is simpler than the 16 unique gene orders found in Anthozoa. These results suggested that a linearized mtDNA structure may be more conducive to Medusozoan mtDNA stability.

Although molecular phylogenetic analyses based on genes encoding mitochondrial proteins clearly supported the monophyly of Anthozoa, gene order analyses did not, possibly because the unique *NAD5* intron of hexagonal corals plays an important role in the phylogenetic analysis.

## 5 CONCLUSION

Our phylogenetic reconstruction based on genes encoding mitochondrial proteins supported Anthozoa being monophyletic and did not support the result of a paraphyletic group of Octocorallia and Medusozoa forming a sister taxon. However, we did not resolve the phylogenetic relationships within the Octocorallia, because there was little variability between the mtDNA of Octocorallians. Staurozoa in Medusozoa appears to be more closely related to Anthozoa. The generally high ω values for most protein‐coding genes in the Anthozoa suggest that the protein‐coding genes of mtDNA in anthozoans have undergone stronger purifying selection compared to medusozoans.

The mtDNA of cnidarians was structurally diverse and complex, with all medusozoans having linear mtDNA, while all Hexacorallians had a self‐splicing intron in the NAD5 gene that contained many intact genes. Further studies are needed to determine the evolutionary relationships between the different mitochondrial gene orders in Cnidaria. In our study, GO1 was predicted to be the ancestral order of the cnidarian mtDNA. Moreover, due to the specificity of the presence of a self‐splicing intron in all NAD5 genes of the Hexacorallia, the use of mtDNA gene order to reconstruct phylogenetic relationships at the level of the Cnidaria is somewhat limited, but it appears to be reliable at a lower taxonomic level. Therefore, we suggest that the involvement of multiple markers such as nuclear genes is required when resolving phylogenetic relationships at higher levels in Metazoans, even when a complete mitochondrial genome is available.

## AUTHOR CONTRIBUTIONS

**Hui Feng:** Data curation (equal); formal analysis (lead); methodology (equal); software (equal); writing – original draft (equal). **Sitong Lv:** Methodology (equal). **Rong Li:** Investigation (equal). **Jing Shi:** Investigation (equal). **Jianxin Wang:** Investigation (equal); validation (equal); writing – review and editing (supporting). **Pinglin Cao:** Funding acquisition (equal); project administration (equal); writing – original draft (equal); writing – review and editing (equal).

## FUNDING INFORMATION

Zhejiang Provincial Natural Science Foundation of China (Q20C060004).

## CONFLICT OF INTEREST STATEMENT

The authors declare that there is no conflict of interests.

## Supporting information


Figure S1.

Figure S2.

Figure S3.

Figure S4.

**Table S1.** **–S2.**

Data S1.

## Data Availability

All the data was collected from NCBI (https://www.ncbi.nlm.nih.gov/). Please check accessions in the supplemental information.
