# Supplementary figures and images for: Mitochondrial genome comparison reveals the evolution of cnidarians
Source: Ecol Evol. 2023 Jun 13;13(6):e10157. doi: 10.1002/ece3.10157 (PMC10261974; doi:10.1002/ece3.10157)

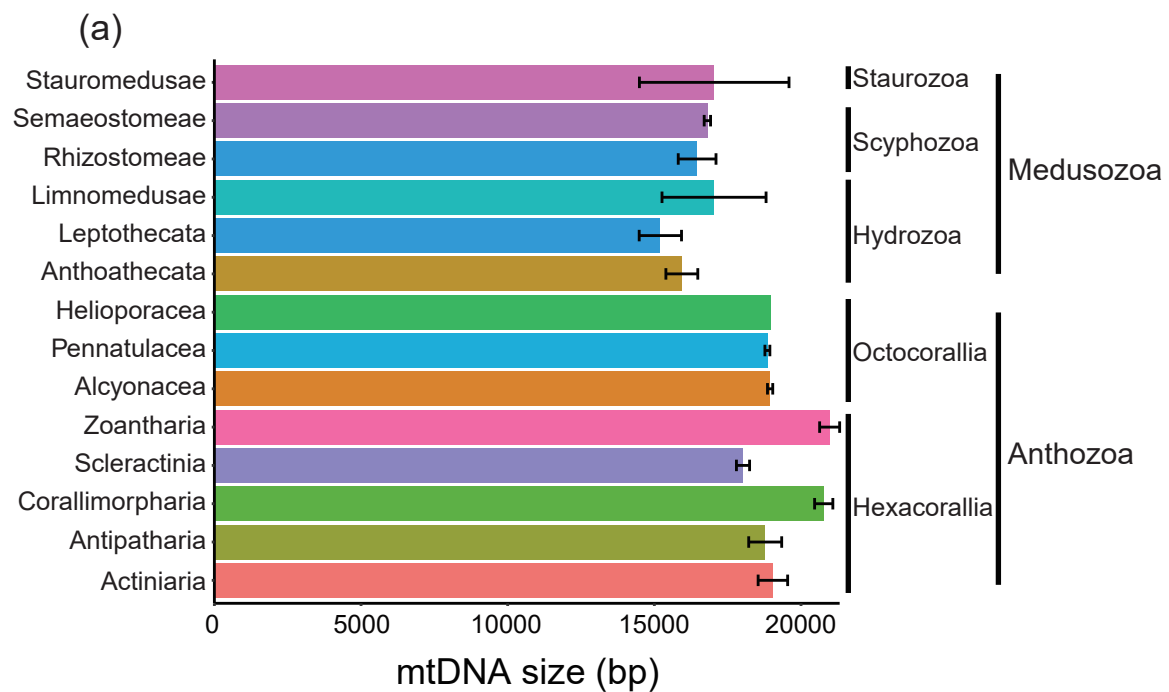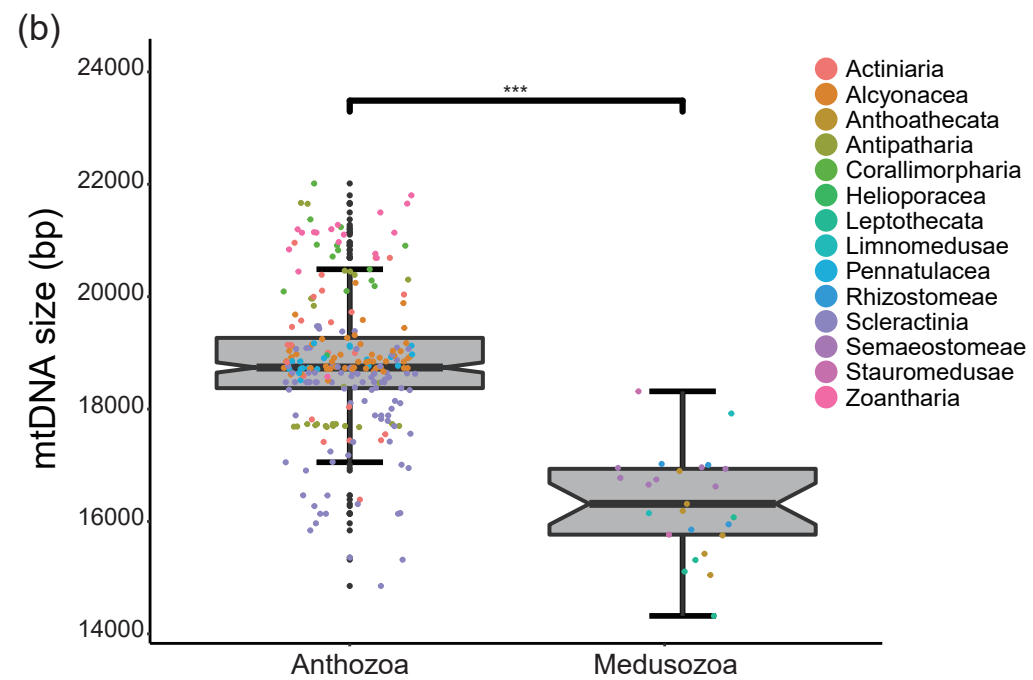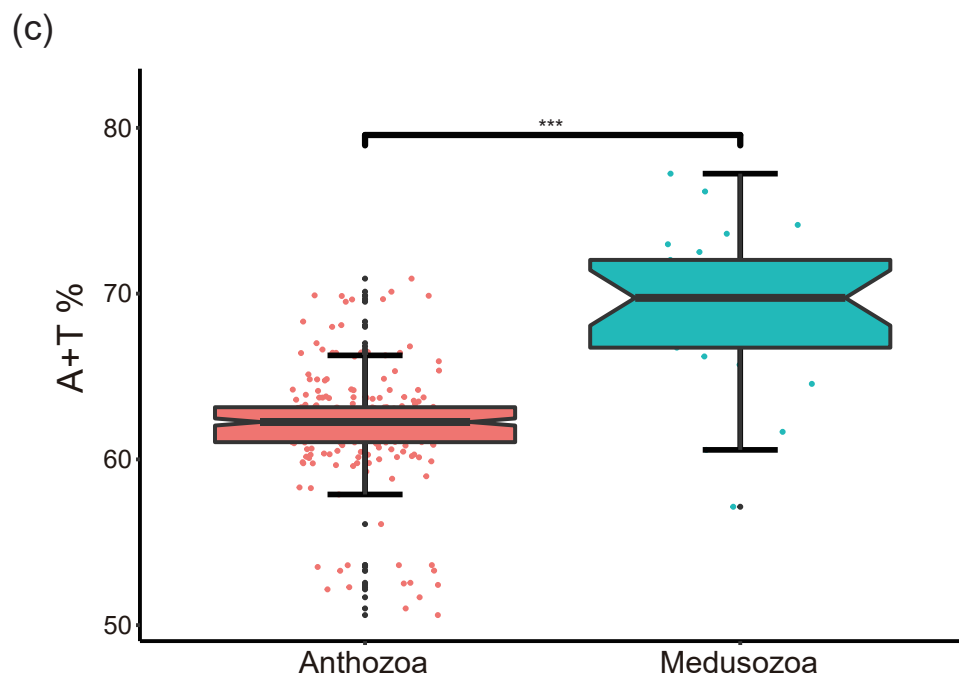

Supplement: Supplementary file 1 — Figure S1. [file ECE3-13-e10157-s003.pdf]

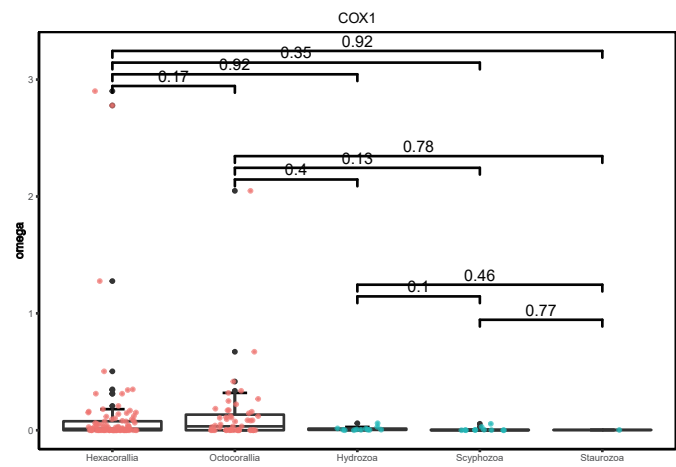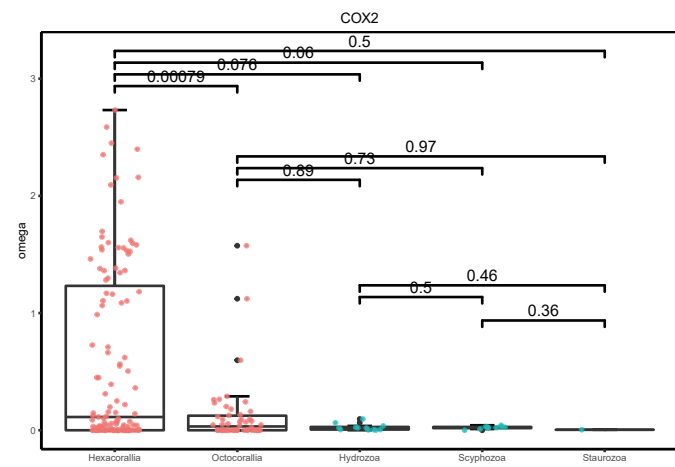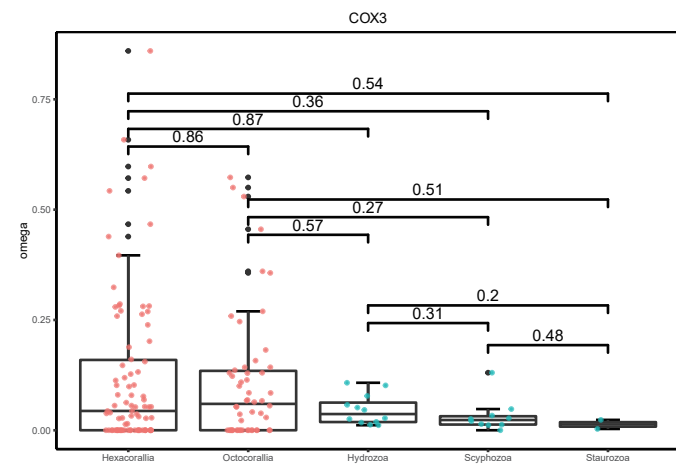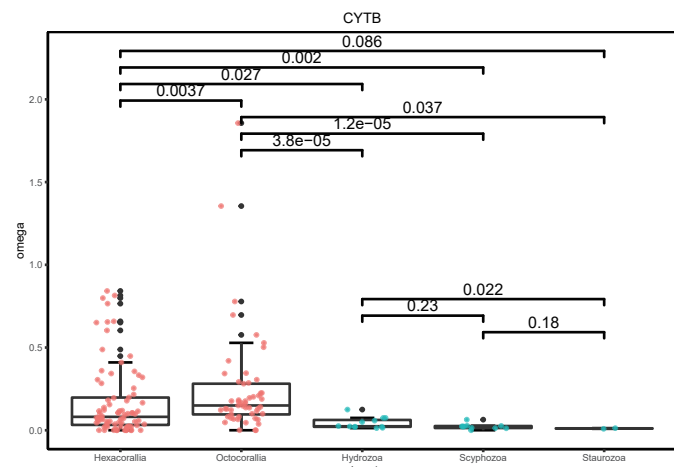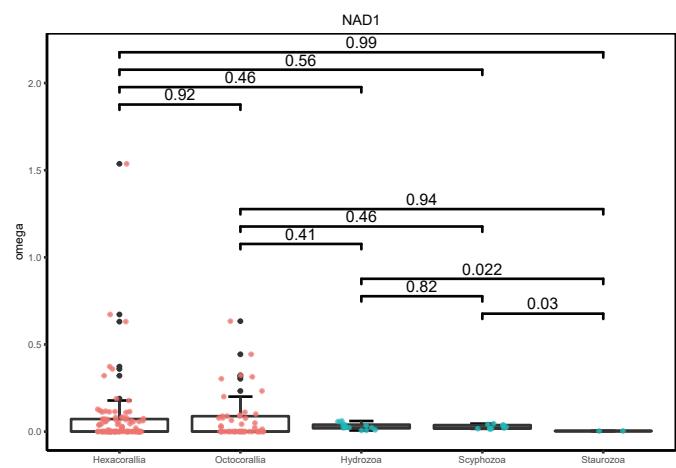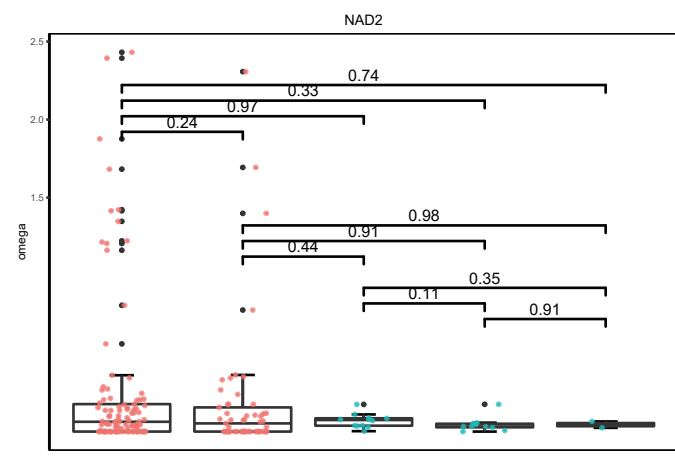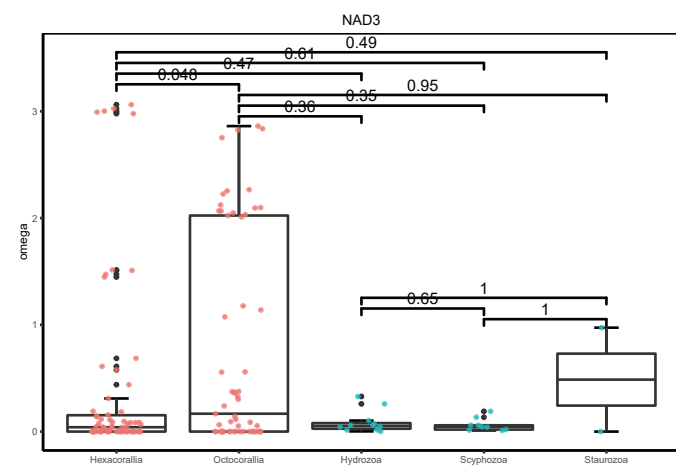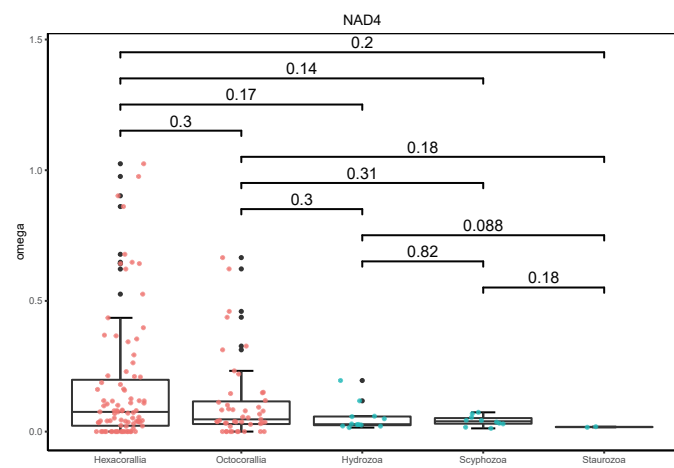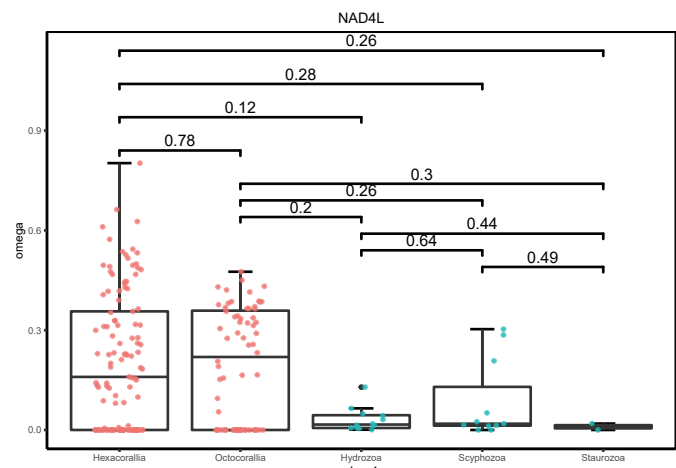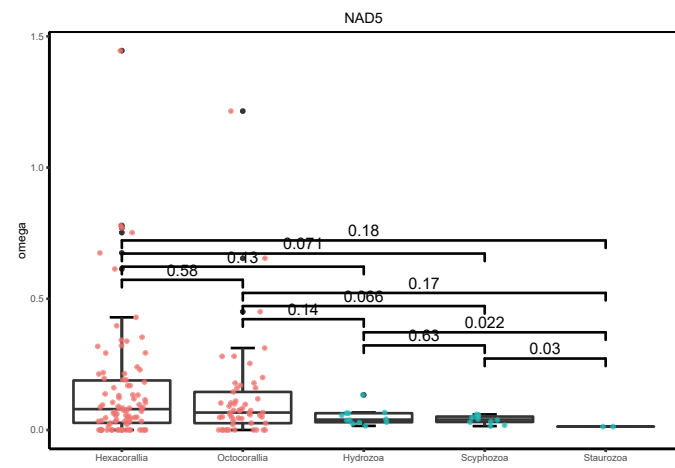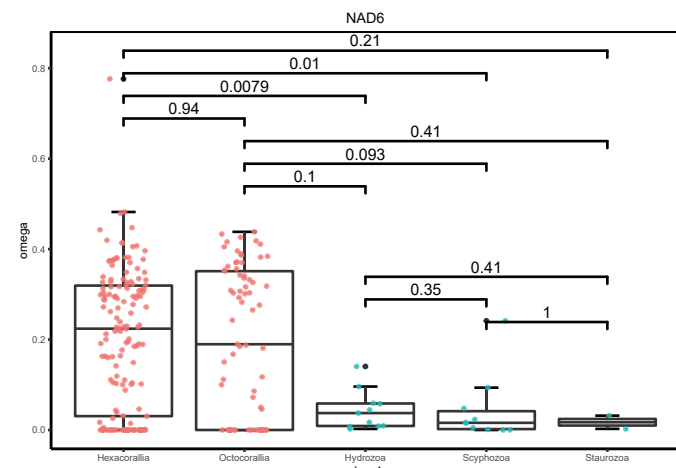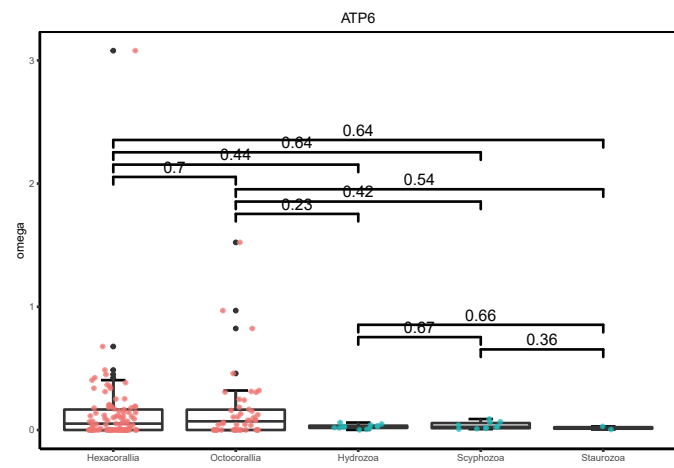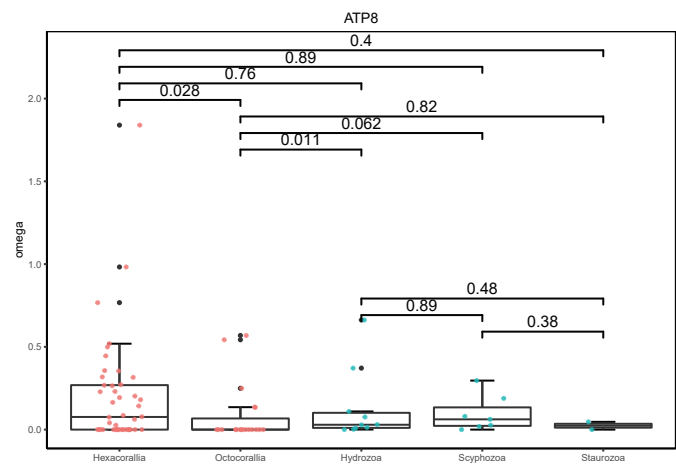

Supplement: Supplementary file 2 — Figure S2. [file ECE3-13-e10157-s006.pdf]

ML

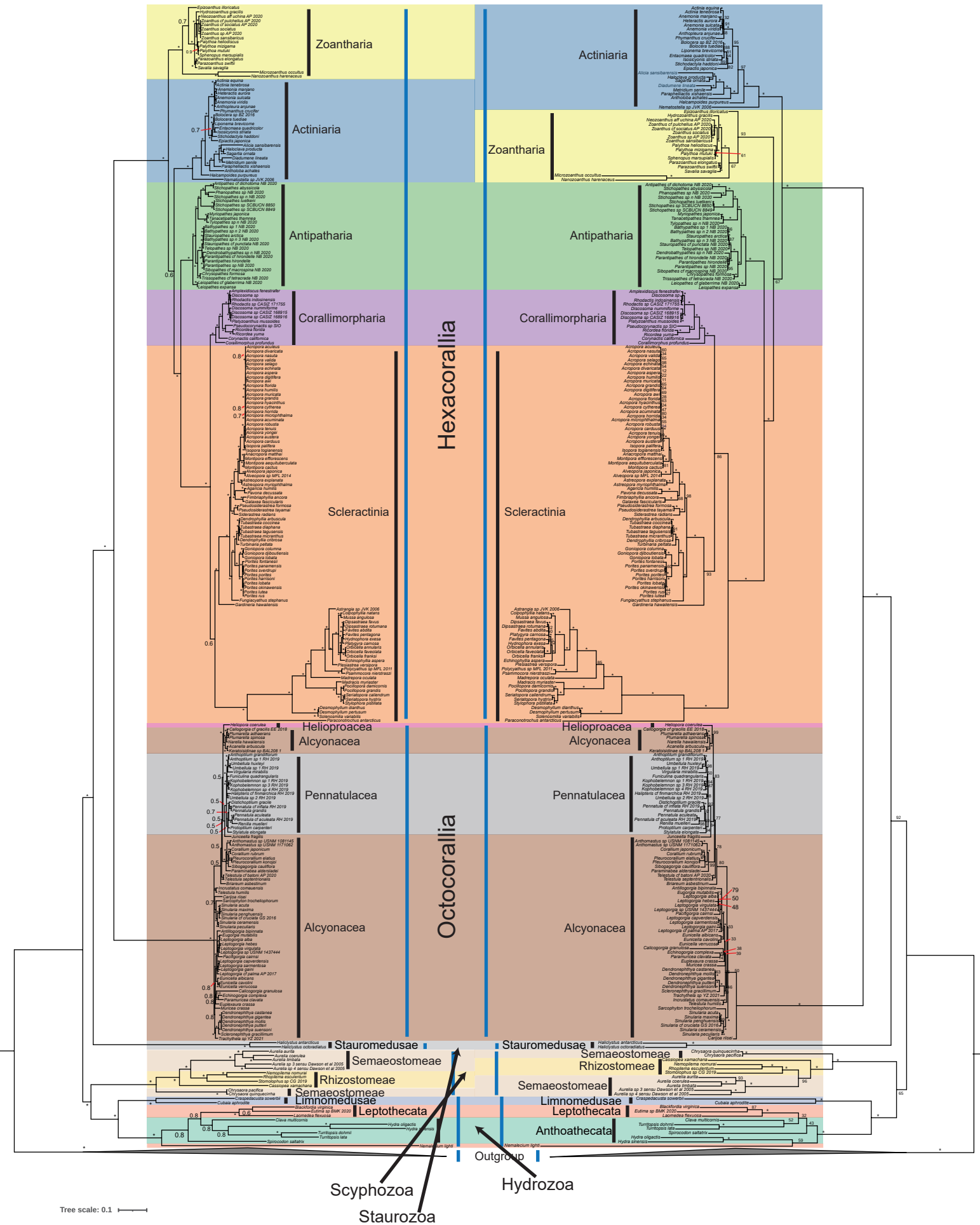

Supplement: Supplementary file 3 — Figure S3. [file ECE3-13-e10157-s001.pdf]

- Hexacorallia
- Octocorallia
- Staurozoa
- Scyphozoa
- Hydrozoa
- Outgroup

Tree scale: 0.1

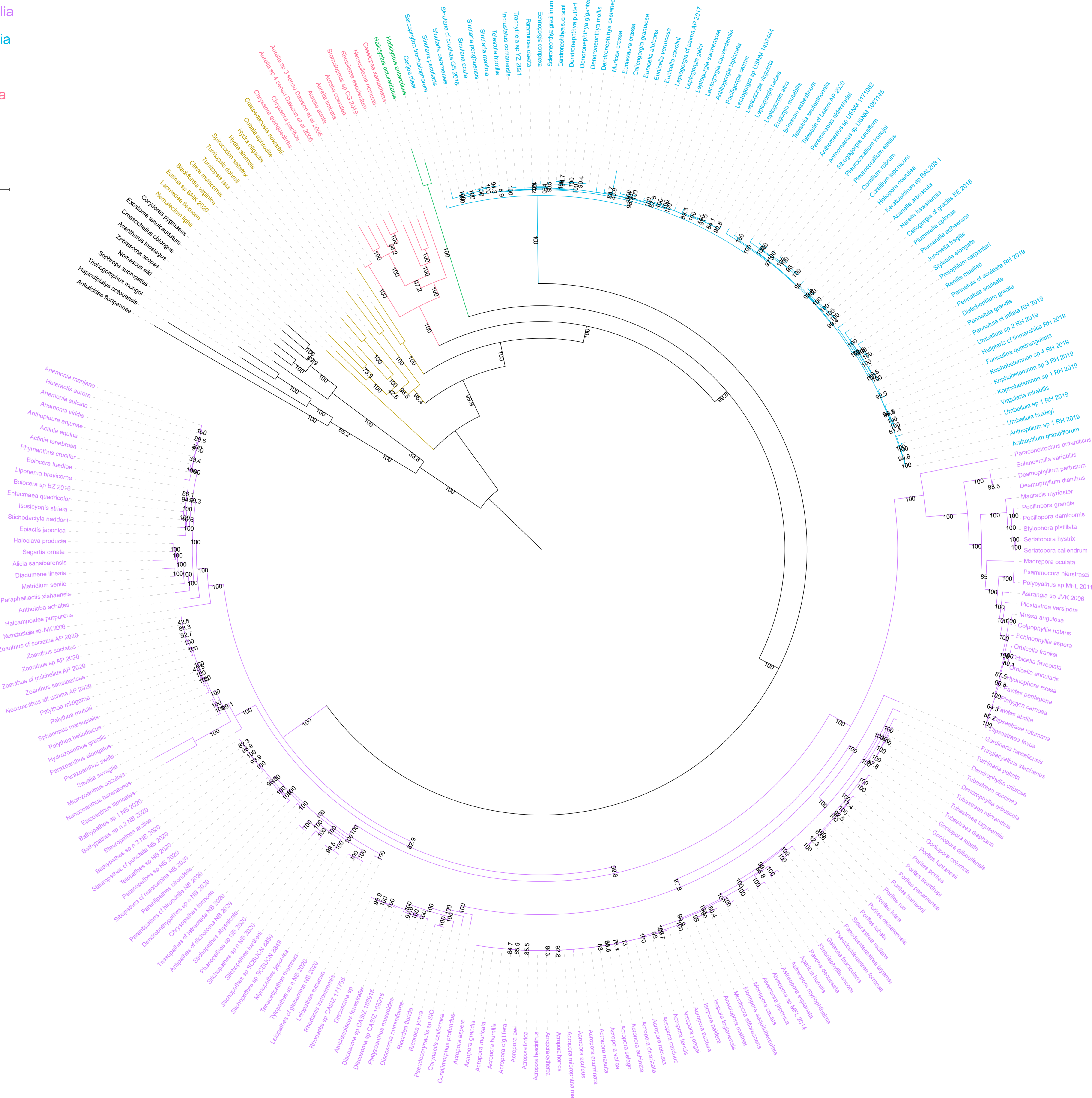

Supplement: Supplementary file 4 — Figure S4. [file ECE3-13-e10157-s004.pdf]
