# Supplementary material for: Mitochondrial genome comparison reveals the evolution of cnidarians
Source: Ecol Evol. 2023 Jun 13;13(6):e10157. doi: 10.1002/ece3.10157 (PMC10261974; doi:10.1002/ece3.10157)
Supplement: Supplementary file 6 — Data S1. [file ECE3-13-e10157-s005.docx]

Figure S1. Characteristics of the mtDNA of cnidarians (a) Mean length (bp ± standard deviation bars) of complete mtDNAs calculated for the order of cnidarians. (b) Comparisons of lengths of mtDNA among the Anthozoans and Medusozoans. (c) Comparisons of A + T content of mtDNA among the Anthozoans and Medusozoans.

Figure S2. Comparisons of ω values among Cnidarian of different Classes base on 13 mtDNA PCGs

Figure S3. The phylogenetic tree of Cnidaria derived from Bayesian inference (BI) and Maximum Likelihood (ML) analyses using 13 PCGs

Figure S4. The phylogenetic tree of Cnidaria derived from Maximum Likelihood (ML) analyses using using the Bilateria as outgroup

**Supplementary Data:** dN, dS and ω values information of every mitochondrial PCG estimated with free-model on codeML.

**ATP6:**

**dS tree:**

(Acanella_arbuscula_NC_011016: 0.023477, ((((((((((((((((((((((((((((((Acropora_aculeus_KT001202: 0.000000, (Acropora_horrida_NC_022825: 0.000000, LC201842_1_LC201842_1__LC201842_1: 0.000000): 0.000000): 0.000000, LC201815_1_LC201815_1__LC201815_1: 0.000000): 0.000000, ((((Acropora_aspera_NC_022827: 0.000000, Acropora_muricata_NC_022824: 0.000000): 0.000000, LC201828_1_LC201828_1__LC201828_1: 0.000000): 0.000000, (((Acropora_digitifera_NC_022830: 0.000000, LC201849_1_LC201849_1__LC201849_1: 0.000000): 0.000000, Acropora_florida_NC_022828: 0.000000): 0.000000, Acropora_humilis_NC_022823: 0.000000): 0.000000): 0.000000, (Acropora_hyacinthus_NC_022826: 0.000000, LC201817_1_LC201817_1__LC201817_1: 0.000000): 0.000000): 0.000000): 0.000000, (Acropora_divaricata_NC_022832: 0.000000, (((Acropora_nasuta_NC_022831: 0.000000, Acropora_valida_NC_042742: 0.000000): 0.000000, LC201847_1_LC201847_1__LC201847_1: 0.000000): 0.000000, LC201820_1_LC201820_1__LC201820_1: 0.000000): 0.000000): 0.000000): 0.000000, Acropora_robusta_NC_022833: 0.000000): 0.000000, LC201813_1_LC201813_1__LC201813_1: 0.000000): 0.000000, ((Acropora_tenuis_NC_003522: 0.000000, Acropora_yongei_NC_022829: 0.000000): 0.000000, LC201816_1_LC201816_1__LC201816_1: 0.013649): 0.000000): 0.000000, (Isopora_palifera_NC_024091: 0.006780, Isopora_togianensis_NC_024089: 0.000000): 0.013675): 0.080023, ((Anacropora_matthai_NC_006898: 0.006567, Montipora_efflorescens_NC_040137: 0.006707): 0.006745, (Montipora_aequituberculata_NC_037359: 0.006656, Montipora_cactus_NC_006902: 0.000000): 0.000000): 0.072064): 0.024163, (Alveopora_japonica_NC_040136: 0.000031, Alveopora_sp__MFL_2014_KJ634271: 0.000000): 0.158225): 0.044607, (Astreopora_explanata_NC_024090: 0.000000, Astreopora_myriophthalma_NC_024092: 0.006971): 0.028761): 0.050617, ((Agaricia_humilis_NC_008160: 0.028079, Pavona_decussata_NC_026527: 0.034969): 0.133635, (Fimbriaphyllia_ancora_NC_015641: 0.013710, Galaxea_fascicularis_NC_029696: 0.015851): 0.184309): 0.034617): 0.058983, ((Pseudosiderastrea_formosa_NC_026530: 0.014287, Pseudosiderastrea_tayamai_NC_026531: 0.008666): 0.115270, Siderastrea_radians_NC_008167: 0.093722): 0.054363): 0.037354, ((((((Dendrophyllia_arbuscula_NC_027590: 0.014911, Tubastraea_coccinea_NC_026025: 0.000005): 0.000005, ((Tubastraea_diaphana_MK959042: 0.000005, Tubastraea_tagusensis_NC_030352: 0.000005): 0.000005, Tubastraea_micranthus_MK959041: 0.000013): 0.000005): 0.018448, Dendrophyllia_cribrosa_NC_026026: 0.011494): 0.037448, Turbinaria_peltata_NC_024671: 0.053425): 0.020199, (((Goniopora_columna_NC_015643: 0.000000, Goniopora_djiboutiensis_NC_045931: 0.000000): 0.011265, Goniopora_lobata_MN795054: 0.033516): 0.142927, ((Porites_fontanesii_NC_037434: 0.047821, ((Porites_panamensis_NC_024182: 0.000005, Porites_sverdrupi_KU956960: 0.007415): 0.000005, Porites_porites_NC_008166: 0.000005): 0.014282): 0.023414, (Porites_harrisoni_NC_037435: 0.007438, ((Porites_lobata_NC_030186: 0.000005, Porites_okinawensis_NC_015644: 0.000005): 0.007446, (Porites_lutea_NC_029695: 0.007439, Porites_rus_NC_027526: 0.007490): 0.000005): 0.000005): 0.023838): 0.091122): 0.086049): 0.017628, Fungiacyathus_stephanus_NC_015640: 0.094782): 0.000005): 0.234506, Gardineria_hawaiiensis_NC_056290: 0.265065): 0.146821, (((((Amplexidiscus_fenestrafer_NC_027101: 0.022765, (Discosoma_sp__MH308003: 0.015173, (Rhodactis_indosinensis_NC_027103: 0.007502, Rhodactis_sp__CASIZ_171755_NC_008158: 0.000005): 0.015161): 0.000005): 0.039271, (Discosoma_nummiforme_NC_027100: 0.015225, ((Discosoma_sp__CASIZ_168915_NC_008071: 0.000005, Discosoma_sp__CASIZ_168916_NC_008072: 0.000005): 0.000005, Platyzoanthus_mussoides_NC_027104: 0.000005): 0.000005): 0.000014): 0.096882, (Pseudocorynactis_sp__SIO_KP938437: 0.116707, (Ricordea_florida_NC_008159: 0.026272, Ricordea_yuma_NC_027106: 0.003785): 0.110193): 0.075898): 0.013838, Corynactis_californica_NC_027102: 0.155504): 0.063732, Corallimorphus_profundus_KP938440: 0.080597): 0.053369): 0.000005, ((((((Astrangia_sp__JVK_2006_NC_008161: 0.130135, (((Colpophyllia_natans_NC_008162: 0.016889, Mussa_angulosa_NC_008163: 0.000013): 0.024822, ((((((Dipsastraea_favus_NC_046690: 0.007772, Dipsastraea_rotumana_NC_044074: 0.034598): 0.017279, Favites_abdita_NC_035879: 0.060008): 0.000005, Platygyra_carnosa_NC_020049: 0.060648): 0.000005, (Favites_pentagona_NC_034916: 0.034057, Hydnophora_exesa_NC_042795: 0.016713): 0.008541): 0.033065, ((Orbicella_annularis_NC_007224: 0.000005, Orbicella_faveolata_NC_007226: 0.000005): 0.000005, Orbicella_franksi_NC_007225: 0.000005): 0.035952): 0.010272, Echinophyllia_aspera_NC_040169: 0.109579): 0.021946): 0.027408, Plesiastrea_versipora_NC_042481: 0.066781): 0.105996): 0.065126, (Polycyathus_sp__MFL_2011_NC_015642: 0.043485, Psammocora_nierstraszi_MT576637: 0.022289): 0.343310): 0.217797, Madrepora_oculata_NC_018364: 0.327158): 0.000028, (Madracis_myriaster_NC_011160: 0.315770, ((Pocillopora_damicornis_NC_009797: 0.000005, Pocillopora_grandis_NC_009798: 0.017409): 0.132521, ((Seriatopora_caliendrum_NC_010245: 0.022300, Seriatopora_hystrix_NC_010244: 0.036999): 0.089917, Stylophora_pistillata_NC_011162: 0.112047): 0.158069): 0.107355): 0.349462): 1.386240, ((Desmophyllum_dianthus_NC_034275: 0.000005, Desmophyllum_pertusum_NC_015143: 0.007775): 0.000032, Solenosmilia_variabilis_NC_025472: 0.007720): 1.437378): 0.550097, Paraconotrochus_antarcticus_NC_056275: 17.555160): 0.004138): 0.329783, ((((((Antipathes_cf__dichotoma_NB_2020_MT318841: 0.000000, Stichopathes_abyssicola_MT318856: 0.000000): 0.150864, (Phanopathes_sp__NB_2020_MT318852: 0.127923, Stichopathes_sp__n__NB_2020_MT318857: 0.167752): 0.036006): 0.114226, ((Stichopathes_luetkeni_NC_018377: 0.003003, Stichopathes_sp__SCBUCN_8850_MZ157399: 0.007606): 0.010719, Stichopathes_sp__SCBUCN_8849_MZ157400: 0.012781): 0.373931): 0.008809, ((Myriopathes_japonica_NC_027667: 0.022454, Tanacetipathes_thamnea_NC_046843: 0.014923): 0.024409, Tylopathes_sp__n__NB_2020_MT318859: 0.027894): 0.169605): 0.038774, (((((((Bathypathes_sp__1_NB_2020_MT318844: 0.000000, Bathypathes_sp__n__2_NB_2020_MT318842: 0.000000): 0.000000, Stauropathes_arctica_MT318854: 0.000000): 0.007198, Bathypathes_sp__n__3_NB_2020_MT318843: 0.000000): 0.028939, Stauropathes_cf__punctata_NB_2020_MT318855: 0.000005): 0.007117, Telopathes_sp__NB_2020_MT318858: 0.007035): 0.021384, (Dendrobathypathes_sp__n__NB_2020_MT318845: 0.021759, ((Parantipathes_cf__hirondelle_NB_2020_MT318849: 0.000000, Parantipathes_hirondelle_MT318850: 0.000000): 0.000000, (Parantipathes_sp__NB_2020_MT318851: 0.000000, Sibopathes_cf__macrospina_NB_2020_MT318853: 0.000000): 0.007086): 0.021507): 0.000014): 0.052015, (Chrysopathes_formosa_NC_008411: 0.107855, Trissopathes_cf__tetracrada_NB_2020_MT318840: 0.004925): 0.039914): 0.005894): 0.093901, (Leiopathes_cf__glaberrima_NB_2020_MT318846: 0.000000, Leiopathes_expansa_MT318847: 0.000000): 0.026636): 0.407335): 0.474100, (Epizoanthus_illoricatus_NC_046474: 0.128159, (((Hydrozoanthus_gracilis_NC_046400: 0.034983, ((Neozoanthus_aff__uchina_AP_2020_MN873592: 0.007009, ((Zoanthus_cf__pulchellus_AP_2020_MN873599: 0.000000, ((Zoanthus_cf__sociatus_AP_2020_MN873600: 0.000000, Zoanthus_sociatus_NC_046476: 0.000000): 0.000000, Zoanthus_sp__AP_2020_MN873602: 0.000000): 0.000000): 0.000000, Zoanthus_sansibaricus_NC_035578: 0.000000): 0.013963): 0.015055, (Palythoa_heliodiscus_NC_035579: 0.071261, ((Palythoa_mizigama_NC_046403: 0.000005, Palythoa_mutuki_NC_046404: 0.013980): 0.000000, Sphenopus_marsupialis_NC_046406: 0.006954): 0.051133): 0.000000): 0.049429): 0.036432, ((Parazoanthus_elongatus_NC_046405: 0.006562, Parazoanthus_swiftii_NC_046475: 0.006541): 0.013826, Savalia_savaglia_DQ825686: 0.038201): 0.000005): 0.091286, (Microzoanthus_occultus_NC_046401: 0.756531, Nanozoanthus_harenaceus_NC_046402: 0.601256): 0.433402): 0.078697): 0.405031): 0.000140, (((((((Actinia_equina_NC_039929: 0.000000, Actinia_tenebrosa_NC_044902: 0.000000): 0.021898, (((Anemonia_manjano_NC_037178: 0.038145, Heteractis_aurora_NC_047219: 0.009840): 0.020356, (Anemonia_sulcata_NC_049065: 0.000000, Anemonia_viridis_NC_037177: 0.000000): 0.007364): 0.022421, Anthopleura_anjunae_NC_030274: 0.007310): 0.000014): 0.000000, Phymanthus_crucifer_NC_027614: 0.068969): 0.016845, ((((Bolocera_sp__BZ_2016_KU507297: 0.000022, (Bolocera_tuediae_NC_022470: 0.000000, Liponema_brevicorne_NC_047221: 0.000000): 0.000000): 0.000000, (Entacmaea_quadricolor_NC_049066: 0.101302, Isosicyonis_striata_KR051006: 0.036812): 0.000000): 0.007089, Stichodactyla_haddoni_MW760873: 0.069544): 0.000000, Epiactis_japonica_NC_047217: 0.077184): 0.005345): 0.164562, ((((Alicia_sansibarensis_NC_027610: 0.245587, (Haloclava_producta_NC_047218: 0.011890, Sagartia_ornata_NC_027615: 0.011217): 0.070862): 0.022221, (Diadumene_lineata_NC_045515: 0.160451, Metridium_senile_NC_000933: 0.065859): 0.088200): 0.069445, Paraphelliactis_xishaensis_MT997141: 0.171452): 0.094593, Antholoba_achates_NC_027611: 0.147242): 0.143379): 0.061697, Halcampoides_purpureus_NC_027612: 0.170383): 0.422905, Nematostella_sp__JVK_2006_NC_008164: 0.786605): 0.903552): 3.434749, (((((((Aurelia_aurita_NC_008446: 0.829540, (Aurelia_coerulea_NC_046792: 0.430740, Aurelia_limbata_NC_046691: 0.487645): 0.124808): 0.000059, (Aurelia_sp__3_sensu_Dawson_et_al___2005__LC005413: 0.482472, Aurelia_sp__4_sensu_Dawson_et_al___2005__LC005414: 0.551999): 0.643079): 1.036393, ((Nemopilema_nomurai_NC_035740: 1.766247, Rhopilema_esculentum_NC_035741: 1.294019): 0.152581, Stomolophus_sp__CG_2019_MK157198: 1.114000): 0.659934): 0.000142, Cassiopea_xamachana_NC_016466: 1.233520): 0.000163, (Chrysaora_pacifica_NC_046775: 0.000005, Chrysaora_quinquecirrha_NC_020459: 0.040868): 2.831552): 1.592232, ((((((Blackfordia_virginica_NC_053946: 4.377323, Eutima_sp__BMK_2020_MW066348: 2.146204): 0.000109, Laomedea_flexuosa_JN700945: 2.429264): 0.000176, (Clava_multicornis_NC_016465: 1.623561, (Turritopsis_dohrnii_NC_031213: 0.734634, Turritopsis_lata_NC_056128: 1.064136): 0.296003): 0.000098): 0.000092, Spirocodon_saltatrix_NC_049868: 2.684490): 0.000097, ((Hydra_oligactis_NC_010214: 2.056025, Hydra_sinensis_NC_021406: 1.750843): 0.000513, Nemalecium_lighti_MZ457217: 23.409658): 0.000271): 0.000620, (Craspedacusta_sowerbii_NC_018537: 63.827352, Cubaia_aphrodite_NC_016467: 3.182183): 0.000335): 0.000270): 0.000005, (Haliclystus_antarcticus_NC_030337: 1.202555, OU744455_1_OU744455_1__OU744455_1: 0.737408): 63.381517): 0.000138): 63.482269, Carijoa_riisei_NC_048963: 0.111327): 0.000005, (Sarcophyton_trocheliophorum_MK994517: 0.130948, (((Sinularia_acuta_MW987591: 0.000014, (Sinularia_maxima_MN485891: 0.000014, Sinularia_penghuensis_MW256412: 0.000014): 0.000000): 0.008358, Sinularia_cf__cruciata_GS_2016_NC_034318: 0.008445): 0.033674, (Sinularia_ceramensis_NC_044122: 0.000005, Sinularia_peculiaris_NC_018379: 0.000005): 0.017689): 0.036820): 0.063701): 0.007212, (((((((Antillogorgia_bipinnata_NC_008157: 0.134302, ((((Eugorgia_mutabilis_NC_035665: 0.059447, Leptogorgia_alba_NC_035669: 0.000005): 0.016799, (Leptogorgia_hebes_MN052675: 0.016769, Leptogorgia_virgulata_MK301586: 0.008338): 0.000005): 0.000005, Leptogorgia_sp__USNM_1437444_KY559412: 0.016747): 0.008044, Pacifigorgia_cairnsi_NC_035668: 0.061191): 0.025426): 0.000000, (((Leptogorgia_capverdensis_NC_035663: 0.000005, Leptogorgia_sarmentosa_NC_035670: 0.000014): 0.016458, Leptogorgia_gaini_KY559404: 0.033160): 0.007901, Leptogorgia_cf__palma_AP_2017_KY559406: 0.032870): 0.000005): 0.008306, (Eunicella_albicans_NC_035666: 0.008178, (Eunicella_cavolini_NC_035667: 0.000005, Eunicella_verrucosa_MW588805: 0.000005): 0.024274): 0.007748): 0.016385, (Calicogorgia_granulosa_NC_023345: 0.804050, Euplexaura_crassa_NC_020458: 0.066277): 0.000005): 0.000014, Muricea_crassa_NC_029697: 0.070500): 0.000005, ((((((Dendronephthya_castanea_NC_023343: 0.000005, Dendronephthya_mollis_NC_020456: 0.000005): 0.000005, Dendronephthya_gigantea_NC_013573: 0.000005): 0.008095, (Dendronephthya_putteri_NC_036022: 0.000005, Dendronephthya_suensoni_NC_022809: 0.000005): 0.000005): 0.084717, Scleronephthya_gracillimum_NC_023344: 0.037970): 0.085212, (Echinogorgia_complexa_NC_020457: 0.033916, Paramuricea_clavata_NC_034749: 0.053251): 0.033587): 0.000005, Trachythela_sp__YZ_2021_MW238423: 0.067607): 0.000005): 0.007511, (Incrustatus_comauensis_MT254531: 0.016726, Telestula_humilis_MT254527: 0.016635): 0.034465): 0.008605): 0.052721, Briareum_asbestinum_NC_008073: 0.024255): 0.025574, (Telestula_cf__batoni_AP_2020_MT254530: 0.016120, Telestula_septentrionalis_MT254532: 0.000005): 0.000014): 0.000014, (((Anthomastus_sp__USNM_1081145_KM015353: 0.008019, Anthomastus_sp__USNM_1171062_KM015352: 0.000005): 0.007905, (((Corallium_japonicum_AB595189: 0.007997, Corallium_rubrum_NC_022864: 0.000022): 0.028606, (Pleurocorallium_elatius_NC_022804: 0.000005, Pleurocorallium_konojoi_NC_015406: 0.000005): 0.056768): 0.014505, Sibogagorgia_cauliflora_NC_026193: 0.041197): 0.000054): 0.000005, Paraminabea_aldersladei_NC_018790: 0.033148): 0.041631): 0.000005, (((((((Anthoptilum_grandiflorum_NC_044086: 0.008132, Anthoptilum_sp__1_RH_2019_MK919656: 0.000005): 0.008215, (Umbellula_huxleyi_MK919668: 0.000005, Umbellula_sp__1_RH_2019_MK919669: 0.016235): 0.016483): 0.008687, Virgularia_mirabilis_NC_044091: 0.078361): 0.016030, (Funiculina_quadrangularis_NC_044078: 0.024358, ((Kophobelemnon_sp__1_RH_2019_MK919660: 0.008030, Kophobelemnon_sp__3_RH_2019_MK919661: 0.000005): 0.000014, Kophobelemnon_sp__4_RH_2019_MK919662: 0.042183): 0.000022): 0.008271): 0.007769, (Halipteris_cf__finmarchica_RH_2019_MK919659: 0.033440, Umbellula_sp__2_RH_2019_MK919670: 0.032424): 0.016424): 0.000005, ((((Distichoptilum_gracile_NC_044077: 0.016486, (Pennatula_cf__inflata_RH_2019_MK919666: 0.000005, Pennatula_grandis_NC_044088: 0.000014): 0.015479): 0.015890, ((Pennatula_aculeata_NC_044087: 0.000005, Pennatula_cf__aculeata_RH_2019_MK919664: 0.007902): 0.046923, Renilla_muelleri_NC_018378: 0.115291): 0.057716): 0.017357, Protoptilum_carpenteri_NC_044089: 0.008016): 0.007980, Stylatula_elongata_NC_018380: 0.075336): 0.000014): 0.029850, Junceella_fragilis_NC_024181: 0.130703): 0.055789): 0.014456, Heliopora_coerulea_NC_020375: 0.083810): 0.027707, ((Callogorgia_cf__gracilis_EE_2018_MH719202: 0.049635, (Plumarella_adhaerans_NC_046480: 0.000005, Plumarella_spinosa_NC_046465: 0.000005): 0.015803): 0.008152, Narella_hawaiiensis_NC_026192: 0.008016): 0.041197): 0.042305, Keratoisidinae_sp__BAL208_1_NC_010764: 0.008481);

**dN tree:**

(Acanella_arbuscula_NC_011016: 0.000002, ((((((((((((((((((((((((((((((Acropora_aculeus_KT001202: 0.000002, (Acropora_horrida_NC_022825: 0.000002, LC201842_1_LC201842_1__LC201842_1: 0.000002): 0.000002): 0.000002, LC201815_1_LC201815_1__LC201815_1: 0.000002): 0.000002, ((((Acropora_aspera_NC_022827: 0.000002, Acropora_muricata_NC_022824: 0.000002): 0.000002, LC201828_1_LC201828_1__LC201828_1: 0.000002): 0.000002, (((Acropora_digitifera_NC_022830: 0.000002, LC201849_1_LC201849_1__LC201849_1: 0.000002): 0.000002, Acropora_florida_NC_022828: 0.000002): 0.000002, Acropora_humilis_NC_022823: 0.000002): 0.000002): 0.000002, (Acropora_hyacinthus_NC_022826: 0.000002, LC201817_1_LC201817_1__LC201817_1: 0.000002): 0.000002): 0.000002): 0.000002, (Acropora_divaricata_NC_022832: 0.000002, (((Acropora_nasuta_NC_022831: 0.000002, Acropora_valida_NC_042742: 0.000002): 0.000002, LC201847_1_LC201847_1__LC201847_1: 0.000002): 0.000002, LC201820_1_LC201820_1__LC201820_1: 0.000002): 0.000002): 0.000002): 0.000002, Acropora_robusta_NC_022833: 0.000002): 0.000002, LC201813_1_LC201813_1__LC201813_1: 0.000002): 0.000002, ((Acropora_tenuis_NC_003522: 0.000002, Acropora_yongei_NC_022829: 0.000002): 0.000002, LC201816_1_LC201816_1__LC201816_1: 0.000001): 0.000002): 0.000002, (Isopora_palifera_NC_024091: 0.000001, Isopora_togianensis_NC_024089: 0.000002): 0.000001): 0.000008, ((Anacropora_matthai_NC_006898: 0.002784, Montipora_efflorescens_NC_040137: 0.000001): 0.000001, (Montipora_aequituberculata_NC_037359: 0.000001, Montipora_cactus_NC_006902: 0.000002): 0.000002): 0.000007): 0.000002, (Alveopora_japonica_NC_040136: 0.008067, Alveopora_sp__MFL_2014_KJ634271: 0.000002): 0.002692): 0.000004, (Astreopora_explanata_NC_024090: 0.000002, Astreopora_myriophthalma_NC_024092: 0.000001): 0.005350): 0.008592, ((Agaricia_humilis_NC_008160: 0.001981, Pavona_decussata_NC_026527: 0.003505): 0.011873, (Fimbriaphyllia_ancora_NC_015641: 0.002580, Galaxea_fascicularis_NC_029696: 0.005348): 0.014822): 0.003355): 0.009781, ((Pseudosiderastrea_formosa_NC_026530: 0.002545, Pseudosiderastrea_tayamai_NC_026531: 0.000001): 0.002618, Siderastrea_radians_NC_008167: 0.045636): 0.000005): 0.002599, ((((((Dendrophyllia_arbuscula_NC_027590: 0.002572, Tubastraea_coccinea_NC_026025: 0.000000): 0.000000, ((Tubastraea_diaphana_MK959042: 0.000000, Tubastraea_tagusensis_NC_030352: 0.000000): 0.000000, Tubastraea_micranthus_MK959041: 0.002553): 0.000000): 0.007769, Dendrophyllia_cribrosa_NC_026026: 0.005183): 0.002562, Turbinaria_peltata_NC_024671: 0.010216): 0.007736, (((Goniopora_columna_NC_015643: 0.000002, Goniopora_djiboutiensis_NC_045931: 0.000002): 0.000001, Goniopora_lobata_MN795054: 0.000003): 0.024252, ((Porites_fontanesii_NC_037434: 0.000005, ((Porites_panamensis_NC_024182: 0.000000, Porites_sverdrupi_KU956960: 0.000001): 0.000000, Porites_porites_NC_008166: 0.000000): 0.000001): 0.000002, (Porites_harrisoni_NC_037435: 0.000001, ((Porites_lobata_NC_030186: 0.000000, Porites_okinawensis_NC_015644: 0.000000): 0.000001, (Porites_lutea_NC_029695: 0.000001, Porites_rus_NC_027526: 0.000001): 0.000000): 0.000000): 0.000002): 0.007682): 0.002601): 0.000002, Fungiacyathus_stephanus_NC_015640: 0.018263): 0.000000): 0.014882, Gardineria_hawaiiensis_NC_056290: 0.047591): 0.000015, (((((Amplexidiscus_fenestrafer_NC_027101: 0.002604, (Discosoma_sp__MH308003: 0.000002, (Rhodactis_indosinensis_NC_027103: 0.000001, Rhodactis_sp__CASIZ_171755_NC_008158: 0.000000): 0.000002): 0.000000): 0.000004, (Discosoma_nummiforme_NC_027100: 0.005161, ((Discosoma_sp__CASIZ_168915_NC_008071: 0.000000, Discosoma_sp__CASIZ_168916_NC_008072: 0.000000): 0.000000, Platyzoanthus_mussoides_NC_027104: 0.000000): 0.000000): 0.002568): 0.005181, (Pseudocorynactis_sp__SIO_KP938437: 0.000012, (Ricordea_florida_NC_008159: 0.008187, Ricordea_yuma_NC_027106: 0.002565): 0.010771): 0.002640): 0.002684, Corynactis_californica_NC_027102: 0.014146): 0.007577, Corallimorphus_profundus_KP938440: 0.032610): 0.030547): 0.000000, ((((((Astrangia_sp__JVK_2006_NC_008161: 0.004628, (((Colpophyllia_natans_NC_008162: 0.002386, Mussa_angulosa_NC_008163: 0.002376): 0.004749, ((((((Dipsastraea_favus_NC_046690: 0.000001, Dipsastraea_rotumana_NC_044074: 0.000003): 0.002396, Favites_abdita_NC_035879: 0.000006): 0.000000, Platygyra_carnosa_NC_020049: 0.007244): 0.000000, (Favites_pentagona_NC_034916: 0.004863, Hydnophora_exesa_NC_042795: 0.002389): 0.002419): 0.002370, ((Orbicella_annularis_NC_007224: 0.000000, Orbicella_faveolata_NC_007226: 0.000000): 0.000000, Orbicella_franksi_NC_007225: 0.000000): 0.004830): 0.000001, Echinophyllia_aspera_NC_040169: 0.012129): 0.000002): 0.020497, Plesiastrea_versipora_NC_042481: 0.005052): 0.010554): 0.006373, (Polycyathus_sp__MFL_2011_NC_015642: 0.002292, Psammocora_nierstraszi_MT576637: 0.000002): 0.032046): 0.029278, Madrepora_oculata_NC_018364: 0.054383): 0.001810, (Madracis_myriaster_NC_011160: 0.004209, ((Pocillopora_damicornis_NC_009797: 0.000000, Pocillopora_grandis_NC_009798: 0.000002): 0.012406, ((Seriatopora_caliendrum_NC_010245: 0.002442, Seriatopora_hystrix_NC_010244: 0.000004): 0.007478, Stylophora_pistillata_NC_011162: 0.004880): 0.012103): 0.023313): 0.086906): 0.073081, ((Desmophyllum_dianthus_NC_034275: 0.000000, Desmophyllum_pertusum_NC_015143: 0.000001): 0.002583, Solenosmilia_variabilis_NC_025472: 0.000001): 0.040856): 0.019051, Paraconotrochus_antarcticus_NC_056275: 0.167286): 0.016388): 0.023782, ((((((Antipathes_cf__dichotoma_NB_2020_MT318841: 0.000002, Stichopathes_abyssicola_MT318856: 0.000002): 0.011415, (Phanopathes_sp__NB_2020_MT318852: 0.005427, Stichopathes_sp__n__NB_2020_MT318857: 0.002754): 0.002819): 0.005510, ((Stichopathes_luetkeni_NC_018377: 0.009245, Stichopathes_sp__SCBUCN_8850_MZ157399: 0.000001): 0.000001, Stichopathes_sp__SCBUCN_8849_MZ157400: 0.000001): 0.000037): 0.000001, ((Myriopathes_japonica_NC_027667: 0.000002, Tanacetipathes_thamnea_NC_046843: 0.002751): 0.000002, Tylopathes_sp__n__NB_2020_MT318859: 0.005575): 0.008392): 0.000004, (((((((Bathypathes_sp__1_NB_2020_MT318844: 0.000002, Bathypathes_sp__n__2_NB_2020_MT318842: 0.000002): 0.000002, Stauropathes_arctica_MT318854: 0.000002): 0.000001, Bathypathes_sp__n__3_NB_2020_MT318843: 0.000002): 0.000003, Stauropathes_cf__punctata_NB_2020_MT318855: 0.000000): 0.000001, Telopathes_sp__NB_2020_MT318858: 0.000001): 0.000002, (Dendrobathypathes_sp__n__NB_2020_MT318845: 0.008362, ((Parantipathes_cf__hirondelle_NB_2020_MT318849: 0.000002, Parantipathes_hirondelle_MT318850: 0.000002): 0.000002, (Parantipathes_sp__NB_2020_MT318851: 0.000002, Sibopathes_cf__macrospina_NB_2020_MT318853: 0.000002): 0.002770): 0.000002): 0.002764): 0.002768, (Chrysopathes_formosa_NC_008411: 0.039587, Trissopathes_cf__tetracrada_NB_2020_MT318840: 0.000000): 0.002744): 0.002761): 0.005120, (Leiopathes_cf__glaberrima_NB_2020_MT318846: 0.000002, Leiopathes_expansa_MT318847: 0.000002): 0.005871): 0.019328): 0.011431, (Epizoanthus_illoricatus_NC_046474: 0.006105, (((Hydrozoanthus_gracilis_NC_046400: 0.008862, ((Neozoanthus_aff__uchina_AP_2020_MN873592: 0.000001, ((Zoanthus_cf__pulchellus_AP_2020_MN873599: 0.000002, ((Zoanthus_cf__sociatus_AP_2020_MN873600: 0.000002, Zoanthus_sociatus_NC_046476: 0.000002): 0.000002, Zoanthus_sp__AP_2020_MN873602: 0.000002): 0.000002): 0.000002, Zoanthus_sansibaricus_NC_035578: 0.000002): 0.005861): 0.002962, (Palythoa_heliodiscus_NC_035579: 0.017898, ((Palythoa_mizigama_NC_046403: 0.000000, Palythoa_mutuki_NC_046404: 0.002885): 0.000002, Sphenopus_marsupialis_NC_046406: 0.000001): 0.002948): 0.000002): 0.003238): 0.014965, ((Parazoanthus_elongatus_NC_046405: 0.000001, Parazoanthus_swiftii_NC_046475: 0.000001): 0.002840, Savalia_savaglia_DQ825686: 0.013565): 0.000000): 0.003086, (Microzoanthus_occultus_NC_046401: 0.043798, Nanozoanthus_harenaceus_NC_046402: 0.021244): 0.017944): 0.003124): 0.022770): 0.016427, (((((((Actinia_equina_NC_039929: 0.000002, Actinia_tenebrosa_NC_044902: 0.000002): 0.000002, (((Anemonia_manjano_NC_037178: 0.000004, Heteractis_aurora_NC_047219: 0.000001): 0.002752, (Anemonia_sulcata_NC_049065: 0.000002, Anemonia_viridis_NC_037177: 0.000002): 0.000001): 0.000002, Anthopleura_anjunae_NC_030274: 0.000001): 0.002744): 0.000002, Phymanthus_crucifer_NC_027614: 0.008239): 0.002747, ((((Bolocera_sp__BZ_2016_KU507297: 0.005541, (Bolocera_tuediae_NC_022470: 0.000002, Liponema_brevicorne_NC_047221: 0.000002): 0.000002): 0.000002, (Entacmaea_quadricolor_NC_049066: 0.011166, Isosicyonis_striata_KR051006: 0.005731): 0.000002): 0.000001, Stichodactyla_haddoni_MW760873: 0.005487): 0.000002, Epiactis_japonica_NC_047217: 0.005474): 0.000001): 0.008583, ((((Alicia_sansibarensis_NC_027610: 0.016392, (Haloclava_producta_NC_047218: 0.000001, Sagartia_ornata_NC_027615: 0.000001): 0.002597): 0.000002, (Diadumene_lineata_NC_045515: 0.016015, Metridium_senile_NC_000933: 0.002658): 0.000009): 0.005430, Paraphelliactis_xishaensis_MT997141: 0.005324): 0.002563, Antholoba_achates_NC_027611: 0.013510): 0.005486): 0.000006, Halcampoides_purpureus_NC_027612: 0.023463): 0.029943, Nematostella_sp__JVK_2006_NC_008164: 0.040641): 0.025085): 0.082469, (((((((Aurelia_aurita_NC_008446: 0.011100, (Aurelia_coerulea_NC_046792: 0.012863, Aurelia_limbata_NC_046691: 0.005017): 0.012349): 0.005836, (Aurelia_sp__3_sensu_Dawson_et_al___2005__LC005413: 0.019354, Aurelia_sp__4_sensu_Dawson_et_al___2005__LC005414: 0.036894): 0.018482): 0.054076, ((Nemopilema_nomurai_NC_035740: 0.012875, Rhopilema_esculentum_NC_035741: 0.022357): 0.020468, Stomolophus_sp__CG_2019_MK157198: 0.024834): 0.005408): 0.019921, Cassiopea_xamachana_NC_016466: 0.109786): 0.016819, (Chrysaora_pacifica_NC_046775: 0.000000, Chrysaora_quinquecirrha_NC_020459: 0.002524): 0.076312): 0.093177, ((((((Blackfordia_virginica_NC_053946: 0.023555, Eutima_sp__BMK_2020_MW066348: 0.042973): 0.007190, Laomedea_flexuosa_JN700945: 0.066506): 0.054432, (Clava_multicornis_NC_016465: 0.078531, (Turritopsis_dohrnii_NC_031213: 0.024538, Turritopsis_lata_NC_056128: 0.029735): 0.101932): 0.010969): 0.029654, Spirocodon_saltatrix_NC_049868: 0.068121): 0.017206, ((Hydra_oligactis_NC_010214: 0.084324, Hydra_sinensis_NC_021406: 0.105919): 0.081441, Nemalecium_lighti_MZ457217: 0.327110): 0.022127): 0.285694, (Craspedacusta_sowerbii_NC_018537: 0.058855, Cubaia_aphrodite_NC_016467: 0.055822): 0.032950): 0.028112): 0.000000, (Haliclystus_antarcticus_NC_030337: 0.005080, OU744455_1_OU744455_1__OU744455_1: 0.021500): 0.215857): 0.005472): 0.180377, Carijoa_riisei_NC_048963: 0.020568): 0.000000, (Sarcophyton_trocheliophorum_MK994517: 0.021922, (((Sinularia_acuta_MW987591: 0.002520, (Sinularia_maxima_MN485891: 0.002519, Sinularia_penghuensis_MW256412: 0.002518): 0.000002): 0.002485, Sinularia_cf__cruciata_GS_2016_NC_034318: 0.012858): 0.005169, (Sinularia_ceramensis_NC_044122: 0.000000, Sinularia_peculiaris_NC_018379: 0.000000): 0.000002): 0.026480): 0.005787): 0.004679, (((((((Antillogorgia_bipinnata_NC_008157: 0.000013, ((((Eugorgia_mutabilis_NC_035665: 0.002584, Leptogorgia_alba_NC_035669: 0.000000): 0.000002, (Leptogorgia_hebes_MN052675: 0.000002, Leptogorgia_virgulata_MK301586: 0.000001): 0.000000): 0.000000, Leptogorgia_sp__USNM_1437444_KY559412: 0.000002): 0.000001, Pacifigorgia_cairnsi_NC_035668: 0.002587): 0.000003): 0.000002, (((Leptogorgia_capverdensis_NC_035663: 0.000000, Leptogorgia_sarmentosa_NC_035670: 0.002585): 0.000002, Leptogorgia_gaini_KY559404: 0.002602): 0.002599, Leptogorgia_cf__palma_AP_2017_KY559406: 0.002592): 0.000000): 0.007820, (Eunicella_albicans_NC_035666: 0.002561, (Eunicella_cavolini_NC_035667: 0.000000, Eunicella_verrucosa_MW588805: 0.000000): 0.002626): 0.010521): 0.000002, (Calicogorgia_granulosa_NC_023345: 0.065449, Euplexaura_crassa_NC_020458: 0.000007): 0.000000): 0.002594, Muricea_crassa_NC_029697: 0.032359): 0.000000, ((((((Dendronephthya_castanea_NC_023343: 0.000000, Dendronephthya_mollis_NC_020456: 0.000000): 0.000000, Dendronephthya_gigantea_NC_013573: 0.000000): 0.000001, (Dendronephthya_putteri_NC_036022: 0.000000, Dendronephthya_suensoni_NC_022809: 0.000000): 0.000000): 0.002608, Scleronephthya_gracillimum_NC_023344: 0.000004): 0.010492, (Echinogorgia_complexa_NC_020457: 0.005190, Paramuricea_clavata_NC_034749: 0.000005): 0.000003): 0.000000, Trachythela_sp__YZ_2021_MW238423: 0.002612): 0.000000): 0.002694, (Incrustatus_comauensis_MT254531: 0.005124, Telestula_humilis_MT254527: 0.000002): 0.007721): 0.003618): 0.010440, Briareum_asbestinum_NC_008073: 0.023507): 0.005464, (Telestula_cf__batoni_AP_2020_MT254530: 0.000002, Telestula_septentrionalis_MT254532: 0.000000): 0.002587): 0.002579, (((Anthomastus_sp__USNM_1081145_KM015353: 0.002569, Anthomastus_sp__USNM_1171062_KM015352: 0.000000): 0.009324, (((Corallium_japonicum_AB595189: 0.000001, Corallium_rubrum_NC_022864: 0.005150): 0.000003, (Pleurocorallium_elatius_NC_022804: 0.000000, Pleurocorallium_konojoi_NC_015406: 0.000000): 0.010371): 0.000001, Sibogagorgia_cauliflora_NC_026193: 0.002563): 0.016668): 0.000000, Paraminabea_aldersladei_NC_018790: 0.027310): 0.006865): 0.000000, (((((((Anthoptilum_grandiflorum_NC_044086: 0.000001, Anthoptilum_sp__1_RH_2019_MK919656: 0.000000): 0.000001, (Umbellula_huxleyi_MK919668: 0.000000, Umbellula_sp__1_RH_2019_MK919669: 0.002573): 0.002554): 0.002509, Virgularia_mirabilis_NC_044091: 0.010262): 0.002619, (Funiculina_quadrangularis_NC_044078: 0.002589, ((Kophobelemnon_sp__1_RH_2019_MK919660: 0.000001, Kophobelemnon_sp__3_RH_2019_MK919661: 0.000000): 0.002560, Kophobelemnon_sp__4_RH_2019_MK919662: 0.010499): 0.005174): 0.000001): 0.000001, (Halipteris_cf__finmarchica_RH_2019_MK919659: 0.002633, Umbellula_sp__2_RH_2019_MK919670: 0.007882): 0.005224): 0.000000, ((((Distichoptilum_gracile_NC_044077: 0.000002, (Pennatula_cf__inflata_RH_2019_MK919666: 0.000000, Pennatula_grandis_NC_044088: 0.002597): 0.000002): 0.000002, ((Pennatula_aculeata_NC_044087: 0.000000, Pennatula_cf__aculeata_RH_2019_MK919664: 0.000001): 0.005325, Renilla_muelleri_NC_018378: 0.005181): 0.007905): 0.000002, Protoptilum_carpenteri_NC_044089: 0.000001): 0.002607, Stylatula_elongata_NC_018380: 0.007920): 0.002610): 0.010509, Junceella_fragilis_NC_024181: 0.013487): 0.007882): 0.000001, Heliopora_coerulea_NC_020375: 0.010378): 0.021221, ((Callogorgia_cf__gracilis_EE_2018_MH719202: 0.002590, (Plumarella_adhaerans_NC_046480: 0.000000, Plumarella_spinosa_NC_046465: 0.000000): 0.005230): 0.000001, Narella_hawaiiensis_NC_026192: 0.000001): 0.006105): 0.002611, Keratoisidinae_sp__BAL208_1_NC_010764: 0.002611);

**w ratios as labels for TreeView:**

(Acanella_arbuscula_NC_011016 #0.0001 , ((((((((((((((((((((((((((((((Acropora_aculeus_KT001202 #16.2932 , (Acropora_horrida_NC_022825 #16.4692 , LC201842_1_LC201842_1__LC201842_1 #16.1381 ) #16.1678 ) #16.1787 , LC201815_1_LC201815_1__LC201815_1 #16.413 ) #16.1378 , ((((Acropora_aspera_NC_022827 #16.1461 , Acropora_muricata_NC_022824 #16.4247 ) #16.1682 , LC201828_1_LC201828_1__LC201828_1 #16.2704 ) #16.1421 , (((Acropora_digitifera_NC_022830 #16.138 , LC201849_1_LC201849_1__LC201849_1 #16.3489 ) #16.1479 , Acropora_florida_NC_022828 #16.271 ) #16.2714 , Acropora_humilis_NC_022823 #16.2642 ) #16.3651 ) #16.3922 , (Acropora_hyacinthus_NC_022826 #16.4281 , LC201817_1_LC201817_1__LC201817_1 #16.4103 ) #16.1114 ) #16.1643 ) #16.164 , (Acropora_divaricata_NC_022832 #16.1781 , (((Acropora_nasuta_NC_022831 #16.3464 , Acropora_valida_NC_042742 #16.3392 ) #16.3982 , LC201847_1_LC201847_1__LC201847_1 #16.3975 ) #16.1333 , LC201820_1_LC201820_1__LC201820_1 #16.1454 ) #16.4282 ) #16.2675 ) #16.1873 , Acropora_robusta_NC_022833 #16.1584 ) #16.1429 , LC201813_1_LC201813_1__LC201813_1 #16.132 ) #16.1264 , ((Acropora_tenuis_NC_003522 #16.1233 , Acropora_yongei_NC_022829 #16.1767 ) #16.2431 , LC201816_1_LC201816_1__LC201816_1 #0.0001 ) #16.1247 ) #41.063 , (Isopora_palifera_NC_024091 #0.0001 , Isopora_togianensis_NC_024089 #15.5863 ) #0.0001 ) #0.0001 , ((Anacropora_matthai_NC_006898 #0.423898 , Montipora_efflorescens_NC_040137 #0.0001 ) #0.0001 , (Montipora_aequituberculata_NC_037359 #0.0001 , Montipora_cactus_NC_006902 #17.3829 ) #16.0916 ) #0.0001 ) #0.0001 , (Alveopora_japonica_NC_040136 #260.94 , Alveopora_sp__MFL_2014_KJ634271 #13.176 ) #0.0170112 ) #0.0001 , (Astreopora_explanata_NC_024090 #12.0178 , Astreopora_myriophthalma_NC_024092 #0.0001 ) #0.186003 ) #0.169737 , ((Agaricia_humilis_NC_008160 #0.0705441 , Pavona_decussata_NC_026527 #0.100232 ) #0.0888452 , (Fimbriaphyllia_ancora_NC_015641 #0.188199 , Galaxea_fascicularis_NC_029696 #0.337366 ) #0.080417 ) #0.0969214 ) #0.16583 , ((Pseudosiderastrea_formosa_NC_026530 #0.178106 , Pseudosiderastrea_tayamai_NC_026531 #0.0001 ) #0.022714 , Siderastrea_radians_NC_008167 #0.486926 ) #0.0001 ) #0.0695725 , ((((((Dendrophyllia_arbuscula_NC_027590 #0.172477 , Tubastraea_coccinea_NC_026025 #0.0001 ) #0.0001 , ((Tubastraea_diaphana_MK959042 #0.0001 , Tubastraea_tagusensis_NC_030352 #0.0001 ) #0.0001 , Tubastraea_micranthus_MK959041 #192.902 ) #0.0001 ) #0.421132 , Dendrophyllia_cribrosa_NC_026026 #0.450895 ) #0.0684144 , Turbinaria_peltata_NC_024671 #0.191214 ) #0.38299 , (((Goniopora_columna_NC_015643 #9.58281 , Goniopora_djiboutiensis_NC_045931 #9.64753 ) #0.0001 , Goniopora_lobata_MN795054 #0.0001 ) #0.169679 , ((Porites_fontanesii_NC_037434 #0.0001 , ((Porites_panamensis_NC_024182 #0.0001 , Porites_sverdrupi_KU956960 #0.0001 ) #0.0001 , Porites_porites_NC_008166 #0.0001 ) #0.0001 ) #0.0001 , (Porites_harrisoni_NC_037435 #0.0001 , ((Porites_lobata_NC_030186 #0.0001 , Porites_okinawensis_NC_015644 #0.0001 ) #0.0001 , (Porites_lutea_NC_029695 #0.0001 , Porites_rus_NC_027526 #0.0001 ) #0.0001 ) #0.0001 ) #0.0001 ) #0.0843099 ) #0.0302223 ) #0.0001 , Fungiacyathus_stephanus_NC_015640 #0.192682 ) #0.0001 ) #0.063461 , Gardineria_hawaiiensis_NC_056290 #0.179546 ) #0.0001 , (((((Amplexidiscus_fenestrafer_NC_027101 #0.114394 , (Discosoma_sp__MH308003 #0.0001 , (Rhodactis_indosinensis_NC_027103 #0.0001 , Rhodactis_sp__CASIZ_171755_NC_008158 #0.0001 ) #0.0001 ) #0.0001 ) #0.0001 , (Discosoma_nummiforme_NC_027100 #0.338963 , ((Discosoma_sp__CASIZ_168915_NC_008071 #0.0001 , Discosoma_sp__CASIZ_168916_NC_008072 #0.0001 ) #0.0001 , Platyzoanthus_mussoides_NC_027104 #0.0001 ) #0.0001 ) #185.253 ) #0.0534735 , (Pseudocorynactis_sp__SIO_KP938437 #0.0001 , (Ricordea_florida_NC_008159 #0.311641 , Ricordea_yuma_NC_027106 #0.677636 ) #0.0977479 ) #0.0347838 ) #0.193955 , Corynactis_californica_NC_027102 #0.0909663 ) #0.118895 , Corallimorphus_profundus_KP938440 #0.4046 ) #0.572367 ) #0.0001 , ((((((Astrangia_sp__JVK_2006_NC_008161 #0.0355639 , (((Colpophyllia_natans_NC_008162 #0.141247 , Mussa_angulosa_NC_008163 #180.27 ) #0.191324 , ((((((Dipsastraea_favus_NC_046690 #0.0001 , Dipsastraea_rotumana_NC_044074 #0.0001 ) #0.138646 , Favites_abdita_NC_035879 #0.0001 ) #0.0001 , Platygyra_carnosa_NC_020049 #0.119452 ) #0.0001 , (Favites_pentagona_NC_034916 #0.142794 , Hydnophora_exesa_NC_042795 #0.142947 ) #0.283215 ) #0.0716681 , ((Orbicella_annularis_NC_007224 #0.0001 , Orbicella_faveolata_NC_007226 #0.0001 ) #0.0001 , Orbicella_franksi_NC_007225 #0.0001 ) #0.13434 ) #0.0001 , Echinophyllia_aspera_NC_040169 #0.110682 ) #0.0001 ) #0.747859 , Plesiastrea_versipora_NC_042481 #0.0756546 ) #0.0995731 ) #0.0978518 , (Polycyathus_sp__MFL_2011_NC_015642 #0.0527127 , Psammocora_nierstraszi_MT576637 #0.0001 ) #0.0933452 ) #0.134429 , Madrepora_oculata_NC_018364 #0.166228 ) #65.1493 , (Madracis_myriaster_NC_011160 #0.0133299 , ((Pocillopora_damicornis_NC_009797 #0.0001 , Pocillopora_grandis_NC_009798 #0.0001 ) #0.0936189 , ((Seriatopora_caliendrum_NC_010245 #0.109498 , Seriatopora_hystrix_NC_010244 #0.0001 ) #0.0831693 , Stylophora_pistillata_NC_011162 #0.0435511 ) #0.0765704 ) #0.217157 ) #0.248686 ) #0.0527187 , ((Desmophyllum_dianthus_NC_034275 #0.0001 , Desmophyllum_pertusum_NC_015143 #0.0001 ) #81.1751 , Solenosmilia_variabilis_NC_025472 #0.0001 ) #0.028424 ) #0.0346314 , Paraconotrochus_antarcticus_NC_056275 #0.00952917 ) #3.95987 ) #0.0721128 , ((((((Antipathes_cf__dichotoma_NB_2020_MT318841 #15.4069 , Stichopathes_abyssicola_MT318856 #14.8035 ) #0.0756668 , (Phanopathes_sp__NB_2020_MT318852 #0.0424238 , Stichopathes_sp__n__NB_2020_MT318857 #0.0164191 ) #0.0782809 ) #0.0482379 , ((Stichopathes_luetkeni_NC_018377 #3.07878 , Stichopathes_sp__SCBUCN_8850_MZ157399 #0.0001 ) #0.0001 , Stichopathes_sp__SCBUCN_8849_MZ157400 #0.0001 ) #0.0001 ) #0.0001 , ((Myriopathes_japonica_NC_027667 #0.0001 , Tanacetipathes_thamnea_NC_046843 #0.184366 ) #0.0001 , Tylopathes_sp__n__NB_2020_MT318859 #0.199876 ) #0.0494802 ) #0.0001 , (((((((Bathypathes_sp__1_NB_2020_MT318844 #12.3036 , Bathypathes_sp__n__2_NB_2020_MT318842 #12.3058 ) #11.6667 , Stauropathes_arctica_MT318854 #12.2923 ) #0.0001 , Bathypathes_sp__n__3_NB_2020_MT318843 #11.7214 ) #0.0001 , Stauropathes_cf__punctata_NB_2020_MT318855 #0.0001 ) #0.0001 , Telopathes_sp__NB_2020_MT318858 #0.0001 ) #0.0001 , (Dendrobathypathes_sp__n__NB_2020_MT318845 #0.384311 , ((Parantipathes_cf__hirondelle_NB_2020_MT318849 #13.6602 , Parantipathes_hirondelle_MT318850 #13.75 ) #14.279 , (Parantipathes_sp__NB_2020_MT318851 #13.693 , Sibopathes_cf__macrospina_NB_2020_MT318853 #13.8105 ) #0.390969 ) #0.0001 ) #196.115 ) #0.0532252 , (Chrysopathes_formosa_NC_008411 #0.367044 , Trissopathes_cf__tetracrada_NB_2020_MT318840 #0.0001 ) #0.0687522 ) #0.468343 ) #0.0545295 , (Leiopathes_cf__glaberrima_NB_2020_MT318846 #12.0102 , Leiopathes_expansa_MT318847 #11.8341 ) #0.220426 ) #0.0474505 ) #0.0241101 , (Epizoanthus_illoricatus_NC_046474 #0.0476344 , (((Hydrozoanthus_gracilis_NC_046400 #0.253325 , ((Neozoanthus_aff__uchina_AP_2020_MN873592 #0.0001 , ((Zoanthus_cf__pulchellus_AP_2020_MN873599 #15.9194 , ((Zoanthus_cf__sociatus_AP_2020_MN873600 #16.0787 , Zoanthus_sociatus_NC_046476 #15.9851 ) #16.1977 , Zoanthus_sp__AP_2020_MN873602 #16.3626 ) #16.1598 ) #16.0222 , Zoanthus_sansibaricus_NC_035578 #16.0501 ) #0.419725 ) #0.196753 , (Palythoa_heliodiscus_NC_035579 #0.251163 , ((Palythoa_mizigama_NC_046403 #0.0001 , Palythoa_mutuki_NC_046404 #0.206371 ) #15.4381 , Sphenopus_marsupialis_NC_046406 #0.0001 ) #0.0576513 ) #16.7049 ) #0.0655026 ) #0.410754 , ((Parazoanthus_elongatus_NC_046405 #0.0001 , Parazoanthus_swiftii_NC_046475 #0.0001 ) #0.205407 , Savalia_savaglia_DQ825686 #0.355089 ) #0.0001 ) #0.0338059 , (Microzoanthus_occultus_NC_046401 #0.0578933 , Nanozoanthus_harenaceus_NC_046402 #0.035332 ) #0.0414029 ) #0.039698 ) #0.0562181 ) #117.344 , (((((((Actinia_equina_NC_039929 #14.3156 , Actinia_tenebrosa_NC_044902 #14.1804 ) #0.0001 , (((Anemonia_manjano_NC_037178 #0.0001 , Heteractis_aurora_NC_047219 #0.0001 ) #0.135174 , (Anemonia_sulcata_NC_049065 #15.2264 , Anemonia_viridis_NC_037177 #15.2267 ) #0.0001 ) #0.0001 , Anthopleura_anjunae_NC_030274 #0.0001 ) #191.147 ) #10.1082 , Phymanthus_crucifer_NC_027614 #0.119457 ) #0.163067 , ((((Bolocera_sp__BZ_2016_KU507297 #251.939 , (Bolocera_tuediae_NC_022470 #13.9148 , Liponema_brevicorne_NC_047221 #14.9812 ) #15.0863 ) #13.7849 , (Entacmaea_quadricolor_NC_049066 #0.110227 , Isosicyonis_striata_KR051006 #0.155675 ) #12.4313 ) #0.0001 , Stichodactyla_haddoni_MW760873 #0.0788974 ) #22.794 , Epiactis_japonica_NC_047217 #0.0709239 ) #0.0001 ) #0.0521561 , ((((Alicia_sansibarensis_NC_027610 #0.0667456 , (Haloclava_producta_NC_047218 #0.0001 , Sagartia_ornata_NC_027615 #0.0001 ) #0.0366548 ) #0.0001 , (Diadumene_lineata_NC_045515 #0.0998125 , Metridium_senile_NC_000933 #0.0403549 ) #0.0001 ) #0.0781937 , Paraphelliactis_xishaensis_MT997141 #0.0310513 ) #0.0270998 , Antholoba_achates_NC_027611 #0.0917506 ) #0.0382657 ) #0.0001 , Halcampoides_purpureus_NC_027612 #0.13771 ) #0.0708027 , Nematostella_sp__JVK_2006_NC_008164 #0.0516663 ) #0.0277626 ) #0.0240101 , (((((((Aurelia_aurita_NC_008446 #0.0133812 , (Aurelia_coerulea_NC_046792 #0.0298632 , Aurelia_limbata_NC_046691 #0.0102889 ) #0.0989469 ) #98.1104 , (Aurelia_sp__3_sensu_Dawson_et_al___2005__LC005413 #0.0401146 , Aurelia_sp__4_sensu_Dawson_et_al___2005__LC005414 #0.0668379 ) #0.02874 ) #0.0521773 , ((Nemopilema_nomurai_NC_035740 #0.00728948 , Rhopilema_esculentum_NC_035741 #0.0172772 ) #0.134143 , Stomolophus_sp__CG_2019_MK157198 #0.0222929 ) #0.00819465 ) #139.888 , Cassiopea_xamachana_NC_016466 #0.0890019 ) #103.454 , (Chrysaora_pacifica_NC_046775 #0.0001 , Chrysaora_quinquecirrha_NC_020459 #0.0617485 ) #0.0269504 ) #0.0585196 , ((((((Blackfordia_virginica_NC_053946 #0.00538123 , Eutima_sp__BMK_2020_MW066348 #0.020023 ) #65.817 , Laomedea_flexuosa_JN700945 #0.027377 ) #309.05 , (Clava_multicornis_NC_016465 #0.0483696 , (Turritopsis_dohrnii_NC_031213 #0.0334022 , Turritopsis_lata_NC_056128 #0.027943 ) #0.344362 ) #111.431 ) #323.843 , Spirocodon_saltatrix_NC_049868 #0.0253757 ) #177.329 , ((Hydra_oligactis_NC_010214 #0.0410132 , Hydra_sinensis_NC_021406 #0.0604959 ) #158.738 , Nemalecium_lighti_MZ457217 #0.0139733 ) #81.5503 ) #460.563 , (Craspedacusta_sowerbii_NC_018537 #0.000922093 , Cubaia_aphrodite_NC_016467 #0.0175419 ) #98.2671 ) #104.092 ) #0.0001 , (Haliclystus_antarcticus_NC_030337 #0.00422403 , OU744455_1_OU744455_1__OU744455_1 #0.0291564 ) #0.00340568 ) #39.577 ) #0.00284137 , Carijoa_riisei_NC_048963 #0.184756 ) #0.0001 , (Sarcophyton_trocheliophorum_MK994517 #0.167412 , (((Sinularia_acuta_MW987591 #184.985 , (Sinularia_maxima_MN485891 #184.861 , Sinularia_penghuensis_MW256412 #184.712 ) #18.1828 ) #0.297297 , Sinularia_cf__cruciata_GS_2016_NC_034318 #1.52267 ) #0.153513 , (Sinularia_ceramensis_NC_044122 #0.0001 , Sinularia_peculiaris_NC_018379 #0.0001 ) #0.0001 ) #0.719168 ) #0.090846 ) #0.648779 , (((((((Antillogorgia_bipinnata_NC_008157 #0.0001 , ((((Eugorgia_mutabilis_NC_035665 #0.0434666 , Leptogorgia_alba_NC_035669 #0.0001 ) #0.0001 , (Leptogorgia_hebes_MN052675 #0.0001 , Leptogorgia_virgulata_MK301586 #0.0001 ) #0.0001 ) #0.0001 , Leptogorgia_sp__USNM_1437444_KY559412 #0.0001 ) #0.0001 , Pacifigorgia_cairnsi_NC_035668 #0.0422787 ) #0.0001 ) #15.6737 , (((Leptogorgia_capverdensis_NC_035663 #0.0001 , Leptogorgia_sarmentosa_NC_035670 #187.952 ) #0.0001 , Leptogorgia_gaini_KY559404 #0.0784705 ) #0.32898 , Leptogorgia_cf__palma_AP_2017_KY559406 #0.0788599 ) #0.0001 ) #0.941516 , (Eunicella_albicans_NC_035666 #0.313181 , (Eunicella_cavolini_NC_035667 #0.0001 , Eunicella_verrucosa_MW588805 #0.0001 ) #0.1082 ) #1.3579 ) #0.0001 , (Calicogorgia_granulosa_NC_023345 #0.0813997 , Euplexaura_crassa_NC_020458 #0.0001 ) #0.0001 ) #179.36 , Muricea_crassa_NC_029697 #0.458994 ) #0.0001 , ((((((Dendronephthya_castanea_NC_023343 #0.0001 , Dendronephthya_mollis_NC_020456 #0.0001 ) #0.0001 , Dendronephthya_gigantea_NC_013573 #0.0001 ) #0.0001 , (Dendronephthya_putteri_NC_036022 #0.0001 , Dendronephthya_suensoni_NC_022809 #0.0001 ) #0.0001 ) #0.0307888 , Scleronephthya_gracillimum_NC_023344 #0.0001 ) #0.123129 , (Echinogorgia_complexa_NC_020457 #0.153036 , Paramuricea_clavata_NC_034749 #0.0001 ) #0.0001 ) #0.0001 , Trachythela_sp__YZ_2021_MW238423 #0.0386399 ) #0.0001 ) #0.35866 , (Incrustatus_comauensis_MT254531 #0.306342 , Telestula_humilis_MT254527 #0.0001 ) #0.22403 ) #0.420474 ) #0.198022 , Briareum_asbestinum_NC_008073 #0.969162 ) #0.213658 , (Telestula_cf__batoni_AP_2020_MT254530 #0.0001 , Telestula_septentrionalis_MT254532 #0.0001 ) #188.739 ) #188.076 , (((Anthomastus_sp__USNM_1081145_KM015353 #0.320387 , Anthomastus_sp__USNM_1171062_KM015352 #0.0001 ) #1.17946 , (((Corallium_japonicum_AB595189 #0.0001 , Corallium_rubrum_NC_022864 #236.076 ) #0.0001 , (Pleurocorallium_elatius_NC_022804 #0.0001 , Pleurocorallium_konojoi_NC_015406 #0.0001 ) #0.18269 ) #0.0001 , Sibogagorgia_cauliflora_NC_026193 #0.0622223 ) #311.32 ) #0.0001 , Paraminabea_aldersladei_NC_018790 #0.823895 ) #0.164906 ) #0.0001 , (((((((Anthoptilum_grandiflorum_NC_044086 #0.0001 , Anthoptilum_sp__1_RH_2019_MK919656 #0.0001 ) #0.0001 , (Umbellula_huxleyi_MK919668 #0.0001 , Umbellula_sp__1_RH_2019_MK919669 #0.158483 ) #0.154931 ) #0.288824 , Virgularia_mirabilis_NC_044091 #0.130954 ) #0.163389 , (Funiculina_quadrangularis_NC_044078 #0.106288 , ((Kophobelemnon_sp__1_RH_2019_MK919660 #0.0001 , Kophobelemnon_sp__3_RH_2019_MK919661 #0.0001 ) #184.363 , Kophobelemnon_sp__4_RH_2019_MK919662 #0.248897 ) #236.729 ) #0.0001 ) #0.0001 , (Halipteris_cf__finmarchica_RH_2019_MK919659 #0.0787354 , Umbellula_sp__2_RH_2019_MK919670 #0.243102 ) #0.31806 ) #0.0001 , ((((Distichoptilum_gracile_NC_044077 #0.0001 , (Pennatula_cf__inflata_RH_2019_MK919666 #0.0001 , Pennatula_grandis_NC_044088 #188.638 ) #0.0001 ) #0.0001 , ((Pennatula_aculeata_NC_044087 #0.0001 , Pennatula_cf__aculeata_RH_2019_MK919664 #0.0001 ) #0.113482 , Renilla_muelleri_NC_018378 #0.0449396 ) #0.136955 ) #0.0001 , Protoptilum_carpenteri_NC_044089 #0.0001 ) #0.326727 , Stylatula_elongata_NC_018380 #0.105124 ) #187.819 ) #0.352071 , Junceella_fragilis_NC_024181 #0.103189 ) #0.141281 ) #0.0001 , Heliopora_coerulea_NC_020375 #0.123824 ) #0.765896 , ((Callogorgia_cf__gracilis_EE_2018_MH719202 #0.0521819 , (Plumarella_adhaerans_NC_046480 #0.0001 , Plumarella_spinosa_NC_046465 #0.0001 ) #0.330973 ) #0.0001 , Narella_hawaiiensis_NC_026192 #0.0001 ) #0.14819 ) #0.0617296 , Keratoisidinae_sp__BAL208_1_NC_010764 #0.307893 );

ATP8:

**dS tree:**

(Acanella_arbuscula_NC_011016: 0.048664, ((((((((((((((((((((((((((((((Acropora_aculeus_KT001202: 0.000006, (Acropora_horrida_NC_022825: 0.000006, LC201842_1_LC201842_1__LC201842_1: 0.000006): 0.000006): 0.000006, LC201815_1_LC201815_1__LC201815_1: 0.000006): 0.000006, ((((Acropora_aspera_NC_022827: 0.000006, Acropora_muricata_NC_022824: 0.000006): 0.000006, LC201828_1_LC201828_1__LC201828_1: 0.000006): 0.000006, (((Acropora_digitifera_NC_022830: 0.000006, LC201849_1_LC201849_1__LC201849_1: 0.000006): 0.000006, Acropora_florida_NC_022828: 0.000006): 0.000006, Acropora_humilis_NC_022823: 0.000006): 0.000006): 0.000006, (Acropora_hyacinthus_NC_022826: 0.000006, LC201817_1_LC201817_1__LC201817_1: 0.000006): 0.000006): 0.000006): 0.000006, (Acropora_divaricata_NC_022832: 0.000006, (((Acropora_nasuta_NC_022831: 0.000006, Acropora_valida_NC_042742: 0.000006): 0.000006, LC201847_1_LC201847_1__LC201847_1: 0.000006): 0.000006, LC201820_1_LC201820_1__LC201820_1: 0.000006): 0.000006): 0.000006): 0.000006, Acropora_robusta_NC_022833: 0.000006): 0.000006, LC201813_1_LC201813_1__LC201813_1: 0.000006): 0.000006, ((Acropora_tenuis_NC_003522: 0.000006, Acropora_yongei_NC_022829: 0.000006): 0.000006, LC201816_1_LC201816_1__LC201816_1: 0.000006): 0.000006): 0.000006, (Isopora_palifera_NC_024091: 0.000006, Isopora_togianensis_NC_024089: 0.000026): 0.041372): 0.091889, ((Anacropora_matthai_NC_006898: 0.000006, Montipora_efflorescens_NC_040137: 0.000006): 0.000006, (Montipora_aequituberculata_NC_037359: 0.000006, Montipora_cactus_NC_006902: 0.000006): 0.000006): 0.041405): 0.000006, (Alveopora_japonica_NC_040136: 0.000006, Alveopora_sp__MFL_2014_KJ634271: 0.000006): 0.045288): 0.000006, (Astreopora_explanata_NC_024090: 0.000014, Astreopora_myriophthalma_NC_024092: 0.000006): 0.140982): 0.088562, ((Agaricia_humilis_NC_008160: 0.000000, Pavona_decussata_NC_026527: 0.000026): 0.182677, (Fimbriaphyllia_ancora_NC_015641: 0.136485, Galaxea_fascicularis_NC_029696: 0.000006): 0.080760): 0.052532): 0.000020, ((Pseudosiderastrea_formosa_NC_026530: 0.000006, Pseudosiderastrea_tayamai_NC_026531: 0.000006): 0.261021, Siderastrea_radians_NC_008167: 0.000018): 0.000006): 0.039390, ((((((Dendrophyllia_arbuscula_NC_027590: 0.000000, Tubastraea_coccinea_NC_026025: 0.000000): 0.000000, ((Tubastraea_diaphana_MK959042: 0.000000, Tubastraea_tagusensis_NC_030352: 0.000000): 0.000000, Tubastraea_micranthus_MK959041: 0.000000): 0.000000): 0.000000, Dendrophyllia_cribrosa_NC_026026: 0.000000): 0.039293, Turbinaria_peltata_NC_024671: 0.038744): 0.000015, (((Goniopora_columna_NC_015643: 0.000000, Goniopora_djiboutiensis_NC_045931: 0.000000): 0.000000, Goniopora_lobata_MN795054: 0.000000): 0.000014, ((Porites_fontanesii_NC_037434: 0.041581, ((Porites_panamensis_NC_024182: 0.000006, Porites_sverdrupi_KU956960: 0.000006): 0.000006, Porites_porites_NC_008166: 0.041511): 0.000014): 0.000006, (Porites_harrisoni_NC_037435: 0.000000, ((Porites_lobata_NC_030186: 0.000000, Porites_okinawensis_NC_015644: 0.000000): 0.000001, (Porites_lutea_NC_029695: 0.000000, Porites_rus_NC_027526: 0.000000): 0.000000): 0.000000): 0.000001): 0.040129): 0.000000): 0.098591, Fungiacyathus_stephanus_NC_015640: 0.168121): 0.000024): 0.000027, Gardineria_hawaiiensis_NC_056290: 0.302105): 0.451092, (((((Amplexidiscus_fenestrafer_NC_027101: 0.000000, (Discosoma_sp__MH308003: 0.000014, (Rhodactis_indosinensis_NC_027103: 0.039438, Rhodactis_sp__CASIZ_171755_NC_008158: 0.000000): 0.000014): 0.000000): 0.000000, (Discosoma_nummiforme_NC_027100: 0.000025, ((Discosoma_sp__CASIZ_168915_NC_008071: 0.000000, Discosoma_sp__CASIZ_168916_NC_008072: 0.000000): 0.000000, Platyzoanthus_mussoides_NC_027104: 0.000000): 0.000000): 0.000000): 0.060135, (Pseudocorynactis_sp__SIO_KP938437: 0.524966, (Ricordea_florida_NC_008159: 0.000015, Ricordea_yuma_NC_027106: 0.039448): 0.124360): 0.069208): 0.000019, Corynactis_californica_NC_027102: 0.146443): 0.000032, Corallimorphus_profundus_KP938440: 0.753509): 0.000006): 0.852986, ((((((Astrangia_sp__JVK_2006_NC_008161: 0.000023, (((Colpophyllia_natans_NC_008162: 0.000006, Mussa_angulosa_NC_008163: 0.053928): 0.000006, ((((((Dipsastraea_favus_NC_046690: 0.000006, Dipsastraea_rotumana_NC_044074: 0.103059): 0.000006, Favites_abdita_NC_035879: 0.000006): 0.000006, Platygyra_carnosa_NC_020049: 0.048415): 0.000006, (Favites_pentagona_NC_034916: 0.056426, Hydnophora_exesa_NC_042795: 0.042992): 0.054837): 0.000006, ((Orbicella_annularis_NC_007224: 0.000006, Orbicella_faveolata_NC_007226: 0.000006): 0.000006, Orbicella_franksi_NC_007225: 0.000006): 0.048572): 0.000006, Echinophyllia_aspera_NC_040169: 0.159405): 0.247627): 0.000000, Plesiastrea_versipora_NC_042481: 0.176675): 0.000006): 0.069187, (Polycyathus_sp__MFL_2011_NC_015642: 0.000090, Psammocora_nierstraszi_MT576637: 0.101839): 0.072905): 0.397651, Madrepora_oculata_NC_018364: 0.000138): 2.668803, (Madracis_myriaster_NC_011160: 0.686510, ((Pocillopora_damicornis_NC_009797: 0.000000, Pocillopora_grandis_NC_009798: 0.000000): 0.208428, ((Seriatopora_caliendrum_NC_010245: 0.000006, Seriatopora_hystrix_NC_010244: 0.000006): 0.000006, Stylophora_pistillata_NC_011162: 0.000015): 0.518399): 0.644454): 68.687310): 1.485383, ((Desmophyllum_dianthus_NC_034275: 0.000006, Desmophyllum_pertusum_NC_015143: 1.036467): 0.000000, Solenosmilia_variabilis_NC_025472: 0.000016): 3.383261): 0.000425, Paraconotrochus_antarcticus_NC_056275: 7.123397): 0.302417): 0.000118, ((((((Antipathes_cf__dichotoma_NB_2020_MT318841: 0.000000, Stichopathes_abyssicola_MT318856: 0.000000): 0.269661, (Phanopathes_sp__NB_2020_MT318852: 0.000000, Stichopathes_sp__n__NB_2020_MT318857: 0.000000): 0.079869): 0.000006, ((Stichopathes_luetkeni_NC_018377: 0.000000, Stichopathes_sp__SCBUCN_8850_MZ157399: 0.000000): 0.081862, Stichopathes_sp__SCBUCN_8849_MZ157400: 0.000006): 0.227094): 0.150816, ((Myriopathes_japonica_NC_027667: 0.000000, Tanacetipathes_thamnea_NC_046843: 0.000000): 0.000000, Tylopathes_sp__n__NB_2020_MT318859: 0.037980): 0.079767): 0.000006, (((((((Bathypathes_sp__1_NB_2020_MT318844: 0.000006, Bathypathes_sp__n__2_NB_2020_MT318842: 0.000006): 0.000006, Stauropathes_arctica_MT318854: 0.000006): 0.000006, Bathypathes_sp__n__3_NB_2020_MT318843: 0.040973): 0.000006, Stauropathes_cf__punctata_NB_2020_MT318855: 0.040973): 0.000006, Telopathes_sp__NB_2020_MT318858: 0.000006): 0.043835, (Dendrobathypathes_sp__n__NB_2020_MT318845: 0.000027, ((Parantipathes_cf__hirondelle_NB_2020_MT318849: 0.000000, Parantipathes_hirondelle_MT318850: 0.000000): 0.000000, (Parantipathes_sp__NB_2020_MT318851: 0.000000, Sibopathes_cf__macrospina_NB_2020_MT318853: 0.000000): 0.000000): 0.039983): 0.038911): 0.083902, (Chrysopathes_formosa_NC_008411: 0.000000, Trissopathes_cf__tetracrada_NB_2020_MT318840: 0.040117): 0.000006): 0.041745): 0.057621, (Leiopathes_cf__glaberrima_NB_2020_MT318846: 0.000006, Leiopathes_expansa_MT318847: 0.000006): 0.078810): 0.000006): 0.000057, (Epizoanthus_illoricatus_NC_046474: 0.278312, (((Hydrozoanthus_gracilis_NC_046400: 0.136293, ((Neozoanthus_aff__uchina_AP_2020_MN873592: 0.000000, ((Zoanthus_cf__pulchellus_AP_2020_MN873599: 0.000000, ((Zoanthus_cf__sociatus_AP_2020_MN873600: 0.000000, Zoanthus_sociatus_NC_046476: 0.000000): 0.000000, Zoanthus_sp__AP_2020_MN873602: 0.000000): 0.000000): 0.000000, Zoanthus_sansibaricus_NC_035578: 0.000000): 0.000000): 0.000000, (Palythoa_heliodiscus_NC_035579: 0.038107, ((Palythoa_mizigama_NC_046403: 0.000000, Palythoa_mutuki_NC_046404: 0.000028): 0.000015, Sphenopus_marsupialis_NC_046406: 0.000000): 0.000000): 0.000000): 0.000000): 0.000015, ((Parazoanthus_elongatus_NC_046405: 0.038984, Parazoanthus_swiftii_NC_046475: 0.000015): 0.038648, Savalia_savaglia_DQ825686: 0.038334): 0.000000): 0.000006, (Microzoanthus_occultus_NC_046401: 0.000187, Nanozoanthus_harenaceus_NC_046402: 69.808875): 3.929614): 0.000006): 2.144388): 0.648943, (((((((Actinia_equina_NC_039929: 0.000000, Actinia_tenebrosa_NC_044902: 0.000000): 0.000000, (((Anemonia_manjano_NC_037178: 0.000000, Heteractis_aurora_NC_047219: 0.000000): 0.038854, (Anemonia_sulcata_NC_049065: 0.000000, Anemonia_viridis_NC_037177: 0.000000): 0.000000): 0.000000, Anthopleura_anjunae_NC_030274: 0.039131): 0.000000): 0.000000, Phymanthus_crucifer_NC_027614: 0.040352): 0.000000, ((((Bolocera_sp__BZ_2016_KU507297: 0.000000, (Bolocera_tuediae_NC_022470: 0.000000, Liponema_brevicorne_NC_047221: 0.000000): 0.000000): 0.000000, (Entacmaea_quadricolor_NC_049066: 0.081306, Isosicyonis_striata_KR051006: 0.000000): 0.000000): 0.000000, Stichodactyla_haddoni_MW760873: 0.085008): 0.000000, Epiactis_japonica_NC_047217: 0.040251): 0.000000): 0.233362, ((((Alicia_sansibarensis_NC_027610: 0.905583, (Haloclava_producta_NC_047218: 0.160844, Sagartia_ornata_NC_027615: 0.000006): 0.000027): 0.000006, (Diadumene_lineata_NC_045515: 0.000006, Metridium_senile_NC_000933: 0.429265): 0.048477): 0.240702, Paraphelliactis_xishaensis_MT997141: 0.133490): 0.218345, Antholoba_achates_NC_027611: 0.090749): 0.331906): 0.000006, Halcampoides_purpureus_NC_027612: 0.047329): 1.056443, Nematostella_sp__JVK_2006_NC_008164: 0.501617): 0.000270): 1.407362, (((((((Aurelia_aurita_NC_008446: 0.604907, (Aurelia_coerulea_NC_046792: 0.461956, Aurelia_limbata_NC_046691: 0.000006): 0.313964): 0.651619, (Aurelia_sp__3_sensu_Dawson_et_al___2005__LC005413: 0.788619, Aurelia_sp__4_sensu_Dawson_et_al___2005__LC005414: 0.470632): 0.077844): 0.000106, ((Nemopilema_nomurai_NC_035740: 0.000078, Rhopilema_esculentum_NC_035741: 1.007031): 0.639894, Stomolophus_sp__CG_2019_MK157198: 0.508007): 41.763576): 0.000122, Cassiopea_xamachana_NC_016466: 6.508620): 0.000254, (Chrysaora_pacifica_NC_046775: 0.000006, Chrysaora_quinquecirrha_NC_020459: 0.000006): 68.771472): 0.000225, ((((((Blackfordia_virginica_NC_053946: 1.667895, Eutima_sp__BMK_2020_MW066348: 11.963285): 0.000121, Laomedea_flexuosa_JN700945: 0.956122): 0.000170, (Clava_multicornis_NC_016465: 3.172761, (Turritopsis_dohrnii_NC_031213: 0.000123, Turritopsis_lata_NC_056128: 1.913190): 1.825833): 69.999454): 0.000006, Spirocodon_saltatrix_NC_049868: 1.037966): 0.000301, ((Hydra_oligactis_NC_010214: 0.000136, Hydra_sinensis_NC_021406: 0.646502): 0.000123, Nemalecium_lighti_MZ457217: 0.851547): 0.000171): 1.658587, (Craspedacusta_sowerbii_NC_018537: 69.903407, Cubaia_aphrodite_NC_016467: 69.515540): 69.568915): 0.000236): 0.000116, (Haliclystus_antarcticus_NC_030337: 0.227739, OU744455_1_OU744455_1__OU744455_1: 2.069660): 67.663297): 0.588410): 0.002100, Carijoa_riisei_NC_048963: 0.000015): 0.000000, (Sarcophyton_trocheliophorum_MK994517: 0.102519, (((Sinularia_acuta_MW987591: 0.000006, (Sinularia_maxima_MN485891: 0.000006, Sinularia_penghuensis_MW256412: 0.000006): 0.000006): 0.000006, Sinularia_cf__cruciata_GS_2016_NC_034318: 0.000006): 0.108470, (Sinularia_ceramensis_NC_044122: 0.000006, Sinularia_peculiaris_NC_018379: 0.000006): 0.000006): 0.051003): 0.000006): 0.049707, (((((((Antillogorgia_bipinnata_NC_008157: 0.046711, ((((Eugorgia_mutabilis_NC_035665: 0.052542, Leptogorgia_alba_NC_035669: 0.000006): 0.000006, (Leptogorgia_hebes_MN052675: 0.000025, Leptogorgia_virgulata_MK301586: 0.000006): 0.000006): 0.000006, Leptogorgia_sp__USNM_1437444_KY559412: 0.000006): 0.000006, Pacifigorgia_cairnsi_NC_035668: 0.101207): 0.000006): 0.000006, (((Leptogorgia_capverdensis_NC_035663: 0.000006, Leptogorgia_sarmentosa_NC_035670: 0.000006): 0.000006, Leptogorgia_gaini_KY559404: 0.000006): 0.047133, Leptogorgia_cf__palma_AP_2017_KY559406: 0.000006): 0.000006): 0.000006, (Eunicella_albicans_NC_035666: 0.047865, (Eunicella_cavolini_NC_035667: 0.000006, Eunicella_verrucosa_MW588805: 0.000006): 0.048651): 0.000006): 0.000006, (Calicogorgia_granulosa_NC_023345: 0.162413, Euplexaura_crassa_NC_020458: 0.000014): 0.000006): 0.000006, Muricea_crassa_NC_029697: 0.106613): 0.000006, ((((((Dendronephthya_castanea_NC_023343: 0.000006, Dendronephthya_mollis_NC_020456: 0.000006): 0.000006, Dendronephthya_gigantea_NC_013573: 0.000006): 0.000006, (Dendronephthya_putteri_NC_036022: 0.000006, Dendronephthya_suensoni_NC_022809: 0.000006): 0.000006): 0.050673, Scleronephthya_gracillimum_NC_023344: 0.000006): 0.000006, (Echinogorgia_complexa_NC_020457: 0.089804, Paramuricea_clavata_NC_034749: 0.193739): 0.068023): 0.049729, Trachythela_sp__YZ_2021_MW238423: 0.097229): 0.000006): 0.000006, (Incrustatus_comauensis_MT254531: 0.000006, Telestula_humilis_MT254527: 0.000006): 0.000006): 0.000006): 0.046711, Briareum_asbestinum_NC_008073: 0.000015): 0.000006, (Telestula_cf__batoni_AP_2020_MT254530: 0.000006, Telestula_septentrionalis_MT254532: 0.000006): 0.000006): 0.000006, (((Anthomastus_sp__USNM_1081145_KM015353: 0.000006, Anthomastus_sp__USNM_1171062_KM015352: 0.048062): 0.000006, (((Corallium_japonicum_AB595189: 0.000006, Corallium_rubrum_NC_022864: 0.000006): 0.000006, (Pleurocorallium_elatius_NC_022804: 0.000006, Pleurocorallium_konojoi_NC_015406: 0.000006): 0.000015): 0.000006, Sibogagorgia_cauliflora_NC_026193: 0.000015): 0.000006): 0.000006, Paraminabea_aldersladei_NC_018790: 0.047600): 0.097881): 0.000015, (((((((Anthoptilum_grandiflorum_NC_044086: 0.000006, Anthoptilum_sp__1_RH_2019_MK919656: 0.000006): 0.000006, (Umbellula_huxleyi_MK919668: 0.000006, Umbellula_sp__1_RH_2019_MK919669: 0.000039): 0.000006): 0.000006, Virgularia_mirabilis_NC_044091: 0.000015): 0.000006, (Funiculina_quadrangularis_NC_044078: 0.000015, ((Kophobelemnon_sp__1_RH_2019_MK919660: 0.000006, Kophobelemnon_sp__3_RH_2019_MK919661: 0.000006): 0.000006, Kophobelemnon_sp__4_RH_2019_MK919662: 0.000006): 0.050633): 0.000006): 0.000006, (Halipteris_cf__finmarchica_RH_2019_MK919659: 0.050068, Umbellula_sp__2_RH_2019_MK919670: 0.000006): 0.000006): 0.000006, ((((Distichoptilum_gracile_NC_044077: 0.049786, (Pennatula_cf__inflata_RH_2019_MK919666: 0.000006, Pennatula_grandis_NC_044088: 0.000006): 0.049628): 0.000006, ((Pennatula_aculeata_NC_044087: 0.000006, Pennatula_cf__aculeata_RH_2019_MK919664: 0.000006): 0.000006, Renilla_muelleri_NC_018378: 0.050861): 0.050549): 0.000006, Protoptilum_carpenteri_NC_044089: 0.000006): 0.000006, Stylatula_elongata_NC_018380: 0.106707): 0.000006): 0.000006, Junceella_fragilis_NC_024181: 0.050633): 0.000015): 0.000006, Heliopora_coerulea_NC_020375: 0.094354): 0.000000, ((Callogorgia_cf__gracilis_EE_2018_MH719202: 0.000006, (Plumarella_adhaerans_NC_046480: 0.000006, Plumarella_spinosa_NC_046465: 0.000006): 0.050633): 0.000006, Narella_hawaiiensis_NC_026192: 0.000006): 0.000015): 0.000006, Keratoisidinae_sp__BAL208_1_NC_010764: 0.000006);

**dN tree:**

(Acanella_arbuscula_NC_011016: 0.000005, ((((((((((((((((((((((((((((((Acropora_aculeus_KT001202: 0.000000, (Acropora_horrida_NC_022825: 0.000000, LC201842_1_LC201842_1__LC201842_1: 0.000000): 0.000000): 0.000000, LC201815_1_LC201815_1__LC201815_1: 0.000000): 0.000000, ((((Acropora_aspera_NC_022827: 0.000000, Acropora_muricata_NC_022824: 0.000000): 0.000000, LC201828_1_LC201828_1__LC201828_1: 0.000000): 0.000000, (((Acropora_digitifera_NC_022830: 0.000000, LC201849_1_LC201849_1__LC201849_1: 0.000000): 0.000000, Acropora_florida_NC_022828: 0.000000): 0.000000, Acropora_humilis_NC_022823: 0.000000): 0.000000): 0.000000, (Acropora_hyacinthus_NC_022826: 0.000000, LC201817_1_LC201817_1__LC201817_1: 0.000000): 0.000000): 0.000000): 0.000000, (Acropora_divaricata_NC_022832: 0.000000, (((Acropora_nasuta_NC_022831: 0.000000, Acropora_valida_NC_042742: 0.000000): 0.000000, LC201847_1_LC201847_1__LC201847_1: 0.000000): 0.000000, LC201820_1_LC201820_1__LC201820_1: 0.000000): 0.000000): 0.000000): 0.000000, Acropora_robusta_NC_022833: 0.000000): 0.000000, LC201813_1_LC201813_1__LC201813_1: 0.000000): 0.000000, ((Acropora_tenuis_NC_003522: 0.000000, Acropora_yongei_NC_022829: 0.000000): 0.000000, LC201816_1_LC201816_1__LC201816_1: 0.000000): 0.000000): 0.000000, (Isopora_palifera_NC_024091: 0.000000, Isopora_togianensis_NC_024089: 0.025627): 0.000004): 0.000009, ((Anacropora_matthai_NC_006898: 0.000000, Montipora_efflorescens_NC_040137: 0.000000): 0.000000, (Montipora_aequituberculata_NC_037359: 0.000000, Montipora_cactus_NC_006902: 0.000000): 0.000000): 0.000004): 0.000000, (Alveopora_japonica_NC_040136: 0.000000, Alveopora_sp__MFL_2014_KJ634271: 0.000000): 0.011862): 0.000000, (Astreopora_explanata_NC_024090: 0.012173, Astreopora_myriophthalma_NC_024092: 0.000000): 0.011991): 0.000009, ((Agaricia_humilis_NC_008160: 0.000002, Pavona_decussata_NC_026527: 0.026063): 0.038361, (Fimbriaphyllia_ancora_NC_015641: 0.104709, Galaxea_fascicularis_NC_029696: 0.000000): 0.026344): 0.000005): 0.012327, ((Pseudosiderastrea_formosa_NC_026530: 0.000000, Pseudosiderastrea_tayamai_NC_026531: 0.000000): 0.000026, Siderastrea_radians_NC_008167: 0.012574): 0.000000): 0.012224, ((((((Dendrophyllia_arbuscula_NC_027590: 0.000002, Tubastraea_coccinea_NC_026025: 0.000002): 0.000002, ((Tubastraea_diaphana_MK959042: 0.000002, Tubastraea_tagusensis_NC_030352: 0.000002): 0.000002, Tubastraea_micranthus_MK959041: 0.000002): 0.000002): 0.000002, Dendrophyllia_cribrosa_NC_026026: 0.000002): 0.012280, Turbinaria_peltata_NC_024671: 0.000004): 0.012428, (((Goniopora_columna_NC_015643: 0.000002, Goniopora_djiboutiensis_NC_045931: 0.000002): 0.000002, Goniopora_lobata_MN795054: 0.000002): 0.012484, ((Porites_fontanesii_NC_037434: 0.000004, ((Porites_panamensis_NC_024182: 0.000000, Porites_sverdrupi_KU956960: 0.000000): 0.000000, Porites_porites_NC_008166: 0.000004): 0.012271): 0.000000, (Porites_harrisoni_NC_037435: 0.000002, ((Porites_lobata_NC_030186: 0.000002, Porites_okinawensis_NC_015644: 0.000002): 0.000001, (Porites_lutea_NC_029695: 0.000002, Porites_rus_NC_027526: 0.000002): 0.000002): 0.000002): 0.000002): 0.000004): 0.000002): 0.000010, Fungiacyathus_stephanus_NC_015640: 0.013122): 0.013103): 0.013198, Gardineria_hawaiiensis_NC_056290: 0.069115): 0.000045, (((((Amplexidiscus_fenestrafer_NC_027101: 0.000002, (Discosoma_sp__MH308003: 0.012613, (Rhodactis_indosinensis_NC_027103: 0.000004, Rhodactis_sp__CASIZ_171755_NC_008158: 0.000002): 0.012371): 0.000002): 0.000002, (Discosoma_nummiforme_NC_027100: 0.024896, ((Discosoma_sp__CASIZ_168915_NC_008071: 0.000002, Discosoma_sp__CASIZ_168916_NC_008072: 0.000002): 0.000002, Platyzoanthus_mussoides_NC_027104: 0.000002): 0.000002): 0.000002): 0.000006, (Pseudocorynactis_sp__SIO_KP938437: 0.039269, (Ricordea_florida_NC_008159: 0.012153, Ricordea_yuma_NC_027106: 0.000004): 0.041053): 0.024627): 0.012561, Corynactis_californica_NC_027102: 0.012570): 0.025683, Corallimorphus_profundus_KP938440: 0.391168): 0.000000): 0.000085, ((((((Astrangia_sp__JVK_2006_NC_008161: 0.022238, (((Colpophyllia_natans_NC_008162: 0.000000, Mussa_angulosa_NC_008163: 0.010917): 0.000000, ((((((Dipsastraea_favus_NC_046690: 0.000000, Dipsastraea_rotumana_NC_044074: 0.000010): 0.000000, Favites_abdita_NC_035879: 0.000000): 0.000000, Platygyra_carnosa_NC_020049: 0.000005): 0.000000, (Favites_pentagona_NC_034916: 0.000006, Hydnophora_exesa_NC_042795: 0.011519): 0.011453): 0.000000, ((Orbicella_annularis_NC_007224: 0.000000, Orbicella_faveolata_NC_007226: 0.000000): 0.000000, Orbicella_franksi_NC_007225: 0.000000): 0.011688): 0.000000, Echinophyllia_aspera_NC_040169: 0.036750): 0.000025): 0.000002, Plesiastrea_versipora_NC_042481: 0.046793): 0.000000): 0.027089, (Polycyathus_sp__MFL_2011_NC_015642: 0.089685, Psammocora_nierstraszi_MT576637: 0.100081): 0.052163): 0.040079, Madrepora_oculata_NC_018364: 0.065375): 0.085283, (Madracis_myriaster_NC_011160: 0.186306, ((Pocillopora_damicornis_NC_009797: 0.000002, Pocillopora_grandis_NC_009798: 0.000002): 0.168521, ((Seriatopora_caliendrum_NC_010245: 0.000000, Seriatopora_hystrix_NC_010244: 0.000000): 0.000000, Stylophora_pistillata_NC_011162: 0.012973): 0.177400): 0.000064): 0.416877): 0.000149, ((Desmophyllum_dianthus_NC_034275: 0.000000, Desmophyllum_pertusum_NC_015143: 0.461833): 0.000002, Solenosmilia_variabilis_NC_025472: 0.012418): 0.225483): 0.206210, Paraconotrochus_antarcticus_NC_056275: 0.292304): 0.026671): 0.075111, ((((((Antipathes_cf__dichotoma_NB_2020_MT318841: 0.000002, Stichopathes_abyssicola_MT318856: 0.000002): 0.040329, (Phanopathes_sp__NB_2020_MT318852: 0.000002, Stichopathes_sp__n__NB_2020_MT318857: 0.000002): 0.012801): 0.000000, ((Stichopathes_luetkeni_NC_018377: 0.000002, Stichopathes_sp__SCBUCN_8850_MZ157399: 0.000002): 0.000008, Stichopathes_sp__SCBUCN_8849_MZ157400: 0.000000): 0.024924): 0.012490, ((Myriopathes_japonica_NC_027667: 0.000002, Tanacetipathes_thamnea_NC_046843: 0.000002): 0.000002, Tylopathes_sp__n__NB_2020_MT318859: 0.000004): 0.038249): 0.000000, (((((((Bathypathes_sp__1_NB_2020_MT318844: 0.000000, Bathypathes_sp__n__2_NB_2020_MT318842: 0.000000): 0.000000, Stauropathes_arctica_MT318854: 0.000000): 0.000000, Bathypathes_sp__n__3_NB_2020_MT318843: 0.000004): 0.000000, Stauropathes_cf__punctata_NB_2020_MT318855: 0.000004): 0.000000, Telopathes_sp__NB_2020_MT318858: 0.000000): 0.000004, (Dendrobathypathes_sp__n__NB_2020_MT318845: 0.007659, ((Parantipathes_cf__hirondelle_NB_2020_MT318849: 0.000002, Parantipathes_hirondelle_MT318850: 0.000002): 0.000002, (Parantipathes_sp__NB_2020_MT318851: 0.000002, Sibopathes_cf__macrospina_NB_2020_MT318853: 0.000002): 0.000002): 0.005437): 0.024963): 0.000008, (Chrysopathes_formosa_NC_008411: 0.000002, Trissopathes_cf__tetracrada_NB_2020_MT318840: 0.012661): 0.000000): 0.000004): 0.010003, (Leiopathes_cf__glaberrima_NB_2020_MT318846: 0.000000, Leiopathes_expansa_MT318847: 0.000000): 0.014536): 0.000000): 0.027813, (Epizoanthus_illoricatus_NC_046474: 0.000028, (((Hydrozoanthus_gracilis_NC_046400: 0.068035, ((Neozoanthus_aff__uchina_AP_2020_MN873592: 0.000002, ((Zoanthus_cf__pulchellus_AP_2020_MN873599: 0.000002, ((Zoanthus_cf__sociatus_AP_2020_MN873600: 0.000002, Zoanthus_sociatus_NC_046476: 0.000002): 0.000002, Zoanthus_sp__AP_2020_MN873602: 0.000002): 0.000002): 0.000002, Zoanthus_sansibaricus_NC_035578: 0.000002): 0.000002): 0.000002, (Palythoa_heliodiscus_NC_035579: 0.013594, ((Palythoa_mizigama_NC_046403: 0.000002, Palythoa_mutuki_NC_046404: 0.028015): 0.013724, Sphenopus_marsupialis_NC_046406: 0.000002): 0.000002): 0.000002): 0.000002): 0.013690, ((Parazoanthus_elongatus_NC_046405: 0.000004, Parazoanthus_swiftii_NC_046475: 0.013288): 0.000004, Savalia_savaglia_DQ825686: 0.000004): 0.000002): 0.000000, (Microzoanthus_occultus_NC_046401: 0.100991, Nanozoanthus_harenaceus_NC_046402: 0.066532): 0.222757): 0.000000): 0.013195): 0.000065, (((((((Actinia_equina_NC_039929: 0.000002, Actinia_tenebrosa_NC_044902: 0.000002): 0.000002, (((Anemonia_manjano_NC_037178: 0.000002, Heteractis_aurora_NC_047219: 0.000002): 0.014228, (Anemonia_sulcata_NC_049065: 0.000002, Anemonia_viridis_NC_037177: 0.000002): 0.000002): 0.000002, Anthopleura_anjunae_NC_030274: 0.000004): 0.000002): 0.000002, Phymanthus_crucifer_NC_027614: 0.014298): 0.000002, ((((Bolocera_sp__BZ_2016_KU507297: 0.000002, (Bolocera_tuediae_NC_022470: 0.000002, Liponema_brevicorne_NC_047221: 0.000002): 0.000002): 0.000002, (Entacmaea_quadricolor_NC_049066: 0.014693, Isosicyonis_striata_KR051006: 0.000002): 0.000002): 0.000002, Stichodactyla_haddoni_MW760873: 0.013993): 0.000002, Epiactis_japonica_NC_047217: 0.074048): 0.000002): 0.014525, ((((Alicia_sansibarensis_NC_027610: 0.056646, (Haloclava_producta_NC_047218: 0.000016, Sagartia_ornata_NC_027615: 0.000000): 0.026818): 0.000000, (Diadumene_lineata_NC_045515: 0.000000, Metridium_senile_NC_000933: 0.000043): 0.013353): 0.000024, Paraphelliactis_xishaensis_MT997141: 0.025845): 0.000022, Antholoba_achates_NC_027611: 0.012970): 0.000033): 0.000000, Halcampoides_purpureus_NC_027612: 0.015063): 0.012955, Nematostella_sp__JVK_2006_NC_008164: 0.013321): 0.114802): 0.117447, (((((((Aurelia_aurita_NC_008446: 0.037587, (Aurelia_coerulea_NC_046792: 0.012262, Aurelia_limbata_NC_046691: 0.000000): 0.000031): 0.021306, (Aurelia_sp__3_sensu_Dawson_et_al___2005__LC005413: 0.000079, Aurelia_sp__4_sensu_Dawson_et_al___2005__LC005414: 0.088818): 0.055130): 0.093712, ((Nemopilema_nomurai_NC_035740: 0.036368, Rhopilema_esculentum_NC_035741: 0.080553): 0.004470, Stomolophus_sp__CG_2019_MK157198: 0.150467): 0.056361): 0.116699, Cassiopea_xamachana_NC_016466: 0.122331): 0.253730, (Chrysaora_pacifica_NC_046775: 0.000000, Chrysaora_quinquecirrha_NC_020459: 0.000000): 0.390587): 0.200128, ((((((Blackfordia_virginica_NC_053946: 0.047210, Eutima_sp__BMK_2020_MW066348: 0.113221): 0.038015, Laomedea_flexuosa_JN700945: 0.072867): 0.166001, (Clava_multicornis_NC_016465: 0.042894, (Turritopsis_dohrnii_NC_031213: 0.063930, Turritopsis_lata_NC_056128: 0.058227): 0.371120): 0.007000): 0.000000, Spirocodon_saltatrix_NC_049868: 0.114102): 0.300295, ((Hydra_oligactis_NC_010214: 0.136313, Hydra_sinensis_NC_021406: 0.240199): 0.113021, Nemalecium_lighti_MZ457217: 0.564012): 0.170586): 0.244811, (Craspedacusta_sowerbii_NC_018537: 0.037003, Cubaia_aphrodite_NC_016467: 0.158161): 0.141489): 0.094615): 0.014462, (Haliclystus_antarcticus_NC_030337: 0.000023, OU744455_1_OU744455_1__OU744455_1: 0.097204): 0.736750): 0.000059): 0.272048, Carijoa_riisei_NC_048963: 0.011822): 0.000002, (Sarcophyton_trocheliophorum_MK994517: 0.000010, (((Sinularia_acuta_MW987591: 0.000000, (Sinularia_maxima_MN485891: 0.000000, Sinularia_penghuensis_MW256412: 0.000000): 0.000000): 0.000000, Sinularia_cf__cruciata_GS_2016_NC_034318: 0.000000): 0.000011, (Sinularia_ceramensis_NC_044122: 0.000000, Sinularia_peculiaris_NC_018379: 0.000000): 0.000000): 0.000005): 0.000000): 0.011944, (((((((Antillogorgia_bipinnata_NC_008157: 0.000005, ((((Eugorgia_mutabilis_NC_035665: 0.000005, Leptogorgia_alba_NC_035669: 0.000000): 0.000000, (Leptogorgia_hebes_MN052675: 0.024572, Leptogorgia_virgulata_MK301586: 0.000000): 0.000000): 0.000000, Leptogorgia_sp__USNM_1437444_KY559412: 0.000000): 0.000000, Pacifigorgia_cairnsi_NC_035668: 0.000010): 0.000000): 0.000000, (((Leptogorgia_capverdensis_NC_035663: 0.000000, Leptogorgia_sarmentosa_NC_035670: 0.000000): 0.000000, Leptogorgia_gaini_KY559404: 0.000000): 0.000005, Leptogorgia_cf__palma_AP_2017_KY559406: 0.000000): 0.000000): 0.000000, (Eunicella_albicans_NC_035666: 0.000005, (Eunicella_cavolini_NC_035667: 0.000000, Eunicella_verrucosa_MW588805: 0.000000): 0.012093): 0.000000): 0.000000, (Calicogorgia_granulosa_NC_023345: 0.092383, Euplexaura_crassa_NC_020458: 0.011954): 0.000000): 0.000000, Muricea_crassa_NC_029697: 0.000011): 0.000000, ((((((Dendronephthya_castanea_NC_023343: 0.000000, Dendronephthya_mollis_NC_020456: 0.000000): 0.000000, Dendronephthya_gigantea_NC_013573: 0.000000): 0.000000, (Dendronephthya_putteri_NC_036022: 0.000000, Dendronephthya_suensoni_NC_022809: 0.000000): 0.000000): 0.000005, Scleronephthya_gracillimum_NC_023344: 0.000000): 0.000000, (Echinogorgia_complexa_NC_020457: 0.012182, Paramuricea_clavata_NC_034749: 0.000019): 0.000007): 0.000005, Trachythela_sp__YZ_2021_MW238423: 0.000010): 0.000000): 0.000000, (Incrustatus_comauensis_MT254531: 0.000000, Telestula_humilis_MT254527: 0.000000): 0.000000): 0.000000): 0.000005, Briareum_asbestinum_NC_008073: 0.012237): 0.000000, (Telestula_cf__batoni_AP_2020_MT254530: 0.000000, Telestula_septentrionalis_MT254532: 0.000000): 0.000000): 0.000000, (((Anthomastus_sp__USNM_1081145_KM015353: 0.000000, Anthomastus_sp__USNM_1171062_KM015352: 0.000005): 0.000000, (((Corallium_japonicum_AB595189: 0.000000, Corallium_rubrum_NC_022864: 0.000000): 0.000000, (Pleurocorallium_elatius_NC_022804: 0.000000, Pleurocorallium_konojoi_NC_015406: 0.000000): 0.013029): 0.000000, Sibogagorgia_cauliflora_NC_026193: 0.012983): 0.000000): 0.000000, Paraminabea_aldersladei_NC_018790: 0.025837): 0.012605): 0.012450, (((((((Anthoptilum_grandiflorum_NC_044086: 0.000000, Anthoptilum_sp__1_RH_2019_MK919656: 0.000000): 0.000000, (Umbellula_huxleyi_MK919668: 0.000000, Umbellula_sp__1_RH_2019_MK919669: 0.039269): 0.000000): 0.000000, Virgularia_mirabilis_NC_044091: 0.012530): 0.000000, (Funiculina_quadrangularis_NC_044078: 0.012500, ((Kophobelemnon_sp__1_RH_2019_MK919660: 0.000000, Kophobelemnon_sp__3_RH_2019_MK919661: 0.000000): 0.000000, Kophobelemnon_sp__4_RH_2019_MK919662: 0.000000): 0.000005): 0.000000): 0.000000, (Halipteris_cf__finmarchica_RH_2019_MK919659: 0.012499, Umbellula_sp__2_RH_2019_MK919670: 0.000000): 0.000000): 0.000000, ((((Distichoptilum_gracile_NC_044077: 0.000005, (Pennatula_cf__inflata_RH_2019_MK919666: 0.000000, Pennatula_grandis_NC_044088: 0.000000): 0.025893): 0.000000, ((Pennatula_aculeata_NC_044087: 0.000000, Pennatula_cf__aculeata_RH_2019_MK919664: 0.000000): 0.000000, Renilla_muelleri_NC_018378: 0.000005): 0.000005): 0.000000, Protoptilum_carpenteri_NC_044089: 0.000000): 0.000000, Stylatula_elongata_NC_018380: 0.000011): 0.000000): 0.000000, Junceella_fragilis_NC_024181: 0.000005): 0.012531): 0.000000, Heliopora_coerulea_NC_020375: 0.012719): 0.000002, ((Callogorgia_cf__gracilis_EE_2018_MH719202: 0.000000, (Plumarella_adhaerans_NC_046480: 0.000000, Plumarella_spinosa_NC_046465: 0.000000): 0.000005): 0.000000, Narella_hawaiiensis_NC_026192: 0.000000): 0.012530): 0.000000, Keratoisidinae_sp__BAL208_1_NC_010764: 0.000000);

**w ratios as labels for TreeView:**

(Acanella_arbuscula_NC_011016 #0.0001 , ((((((((((((((((((((((((((((((Acropora_aculeus_KT001202 #0.0001 , (Acropora_horrida_NC_022825 #0.0001 , LC201842_1_LC201842_1__LC201842_1 #0.0001 ) #0.0001 ) #0.0001 , LC201815_1_LC201815_1__LC201815_1 #0.0001 ) #0.0001 , ((((Acropora_aspera_NC_022827 #0.0001 , Acropora_muricata_NC_022824 #0.0001 ) #0.0001 , LC201828_1_LC201828_1__LC201828_1 #0.0001 ) #0.0001 , (((Acropora_digitifera_NC_022830 #0.0001 , LC201849_1_LC201849_1__LC201849_1 #0.0001 ) #0.0001 , Acropora_florida_NC_022828 #0.0001 ) #0.0001 , Acropora_humilis_NC_022823 #0.0001 ) #0.0001 ) #0.0001 , (Acropora_hyacinthus_NC_022826 #0.0001 , LC201817_1_LC201817_1__LC201817_1 #0.0001 ) #0.0001 ) #0.0001 ) #0.0001 , (Acropora_divaricata_NC_022832 #0.0001 , (((Acropora_nasuta_NC_022831 #0.0001 , Acropora_valida_NC_042742 #0.0001 ) #0.0001 , LC201847_1_LC201847_1__LC201847_1 #0.0001 ) #0.0001 , LC201820_1_LC201820_1__LC201820_1 #0.0001 ) #0.0001 ) #0.0001 ) #0.0001 , Acropora_robusta_NC_022833 #0.0001 ) #0.0001 , LC201813_1_LC201813_1__LC201813_1 #0.0001 ) #0.0001 , ((Acropora_tenuis_NC_003522 #0.0001 , Acropora_yongei_NC_022829 #0.0001 ) #0.0001 , LC201816_1_LC201816_1__LC201816_1 #0.0001 ) #0.0001 ) #0.0001 , (Isopora_palifera_NC_024091 #0.0001 , Isopora_togianensis_NC_024089 #999 ) #0.0001 ) #0.0001 , ((Anacropora_matthai_NC_006898 #0.0001 , Montipora_efflorescens_NC_040137 #0.0001 ) #0.0001 , (Montipora_aequituberculata_NC_037359 #0.0001 , Montipora_cactus_NC_006902 #0.0001 ) #0.0001 ) #0.0001 ) #0.0001 , (Alveopora_japonica_NC_040136 #0.0001 , Alveopora_sp__MFL_2014_KJ634271 #0.0001 ) #0.261918 ) #0.0001 , (Astreopora_explanata_NC_024090 #858.892 , Astreopora_myriophthalma_NC_024092 #0.0001 ) #0.085054 ) #0.0001 , ((Agaricia_humilis_NC_008160 #25.3446 , Pavona_decussata_NC_026527 #999 ) #0.209992 , (Fimbriaphyllia_ancora_NC_015641 #0.767185 , Galaxea_fascicularis_NC_029696 #0.0001 ) #0.326204 ) #0.0001 ) #615.817 , ((Pseudosiderastrea_formosa_NC_026530 #0.0001 , Pseudosiderastrea_tayamai_NC_026531 #0.0001 ) #0.0001 , Siderastrea_radians_NC_008167 #687.6 ) #0.0001 ) #0.310328 , ((((((Dendrophyllia_arbuscula_NC_027590 #13.1593 , Tubastraea_coccinea_NC_026025 #12.8324 ) #13.1841 , ((Tubastraea_diaphana_MK959042 #13.1882 , Tubastraea_tagusensis_NC_030352 #12.4887 ) #12.6016 , Tubastraea_micranthus_MK959041 #12.8341 ) #13.1573 ) #13.17 , Dendrophyllia_cribrosa_NC_026026 #11.8249 ) #0.31251 , Turbinaria_peltata_NC_024671 #0.0001 ) #852.338 , (((Goniopora_columna_NC_015643 #9.06109 , Goniopora_djiboutiensis_NC_045931 #7.8486 ) #8.50405 , Goniopora_lobata_MN795054 #8.72236 ) #881.721 , ((Porites_fontanesii_NC_037434 #0.0001 , ((Porites_panamensis_NC_024182 #0.0001 , Porites_sverdrupi_KU956960 #0.0001 ) #0.0001 , Porites_porites_NC_008166 #0.0001 ) #864.241 ) #0.0001 , (Porites_harrisoni_NC_037435 #6.5 , ((Porites_lobata_NC_030186 #4.96475 , Porites_okinawensis_NC_015644 #4.3575 ) #0.948616 , (Porites_lutea_NC_029695 #4.91712 , Porites_rus_NC_027526 #4.49296 ) #3.79163 ) #3.78306 ) #2.42375 ) #0.0001 ) #29.61 ) #0.0001 , Fungiacyathus_stephanus_NC_015640 #0.0780538 ) #536.29 ) #487.582 , Gardineria_hawaiiensis_NC_056290 #0.228779 ) #0.0001 , (((((Amplexidiscus_fenestrafer_NC_027101 #16.9694 , (Discosoma_sp__MH308003 #893.119 , (Rhodactis_indosinensis_NC_027103 #0.0001 , Rhodactis_sp__CASIZ_171755_NC_008158 #10.3904 ) #875.377 ) #18.2357 ) #17.284 , (Discosoma_nummiforme_NC_027100 #999 , ((Discosoma_sp__CASIZ_168915_NC_008071 #17.1278 , Discosoma_sp__CASIZ_168916_NC_008072 #17.4387 ) #17.4335 , Platyzoanthus_mussoides_NC_027104 #16.084 ) #17.1182 ) #17.1846 ) #0.0001 , (Pseudocorynactis_sp__SIO_KP938437 #0.0748029 , (Ricordea_florida_NC_008159 #838.037 , Ricordea_yuma_NC_027106 #0.0001 ) #0.330115 ) #0.355836 ) #654.588 , Corynactis_californica_NC_027102 #0.085832 ) #798.173 , Corallimorphus_profundus_KP938440 #0.519129 ) #0.0001 ) #0.0001 , ((((((Astrangia_sp__JVK_2006_NC_008161 #968.716 , (((Colpophyllia_natans_NC_008162 #0.0001 , Mussa_angulosa_NC_008163 #0.202446 ) #0.0001 , ((((((Dipsastraea_favus_NC_046690 #0.0001 , Dipsastraea_rotumana_NC_044074 #0.0001 ) #0.0001 , Favites_abdita_NC_035879 #0.0001 ) #0.0001 , Platygyra_carnosa_NC_020049 #0.0001 ) #0.0001 , (Favites_pentagona_NC_034916 #0.0001 , Hydnophora_exesa_NC_042795 #0.267925 ) #0.208859 ) #0.0001 , ((Orbicella_annularis_NC_007224 #0.0001 , Orbicella_faveolata_NC_007226 #0.0001 ) #0.0001 , Orbicella_franksi_NC_007225 #0.0001 ) #0.240632 ) #0.0001 , Echinophyllia_aspera_NC_040169 #0.230545 ) #0.0001 ) #39.783 , Plesiastrea_versipora_NC_042481 #0.264853 ) #0.0001 ) #0.391532 , (Polycyathus_sp__MFL_2011_NC_015642 #999 , Psammocora_nierstraszi_MT576637 #0.982739 ) #0.715487 ) #0.100788 , Madrepora_oculata_NC_018364 #474.684 ) #0.0319557 , (Madracis_myriaster_NC_011160 #0.271382 , ((Pocillopora_damicornis_NC_009797 #19.6912 , Pocillopora_grandis_NC_009798 #19.8272 ) #0.808531 , ((Seriatopora_caliendrum_NC_010245 #0.0001 , Seriatopora_hystrix_NC_010244 #0.0001 ) #0.0001 , Stylophora_pistillata_NC_011162 #844.353 ) #0.342206 ) #0.0001 ) #0.0060692 ) #0.0001 , ((Desmophyllum_dianthus_NC_034275 #0.0001 , Desmophyllum_pertusum_NC_015143 #0.445584 ) #18.9168 , Solenosmilia_variabilis_NC_025472 #780.526 ) #0.0666465 ) #485.658 , Paraconotrochus_antarcticus_NC_056275 #0.0410343 ) #0.0881921 ) #635.672 , ((((((Antipathes_cf__dichotoma_NB_2020_MT318841 #25.4466 , Stichopathes_abyssicola_MT318856 #25.5689 ) #0.149553 , (Phanopathes_sp__NB_2020_MT318852 #24.3841 , Stichopathes_sp__n__NB_2020_MT318857 #24.3866 ) #0.160273 ) #0.0001 , ((Stichopathes_luetkeni_NC_018377 #19.1897 , Stichopathes_sp__SCBUCN_8850_MZ157399 #19.0273 ) #0.0001 , Stichopathes_sp__SCBUCN_8849_MZ157400 #0.0001 ) #0.109753 ) #0.082815 , ((Myriopathes_japonica_NC_027667 #20.0346 , Tanacetipathes_thamnea_NC_046843 #20.0567 ) #16.6174 , Tylopathes_sp__n__NB_2020_MT318859 #0.0001 ) #0.479509 ) #0.0001 , (((((((Bathypathes_sp__1_NB_2020_MT318844 #0.0001 , Bathypathes_sp__n__2_NB_2020_MT318842 #0.0001 ) #0.0001 , Stauropathes_arctica_MT318854 #0.0001 ) #0.0001 , Bathypathes_sp__n__3_NB_2020_MT318843 #0.0001 ) #0.0001 , Stauropathes_cf__punctata_NB_2020_MT318855 #0.0001 ) #0.0001 , Telopathes_sp__NB_2020_MT318858 #0.0001 ) #0.0001 , (Dendrobathypathes_sp__n__NB_2020_MT318845 #287.82 , ((Parantipathes_cf__hirondelle_NB_2020_MT318849 #12.6262 , Parantipathes_hirondelle_MT318850 #12.3365 ) #12.6069 , (Parantipathes_sp__NB_2020_MT318851 #11.6062 , Sibopathes_cf__macrospina_NB_2020_MT318853 #12.56 ) #12.5807 ) #0.135994 ) #0.641535 ) #0.0001 , (Chrysopathes_formosa_NC_008411 #14.8343 , Trissopathes_cf__tetracrada_NB_2020_MT318840 #0.315593 ) #0.0001 ) #0.0001 ) #0.173595 , (Leiopathes_cf__glaberrima_NB_2020_MT318846 #0.0001 , Leiopathes_expansa_MT318847 #0.0001 ) #0.184444 ) #0.0001 ) #490.385 , (Epizoanthus_illoricatus_NC_046474 #0.0001 , (((Hydrozoanthus_gracilis_NC_046400 #0.499179 , ((Neozoanthus_aff__uchina_AP_2020_MN873592 #24.6701 , ((Zoanthus_cf__pulchellus_AP_2020_MN873599 #24.582 , ((Zoanthus_cf__sociatus_AP_2020_MN873600 #24.664 , Zoanthus_sociatus_NC_046476 #24.575 ) #24.5809 , Zoanthus_sp__AP_2020_MN873602 #24.5561 ) #24.5832 ) #24.6988 , Zoanthus_sansibaricus_NC_035578 #24.676 ) #24.6657 ) #24.5807 , (Palythoa_heliodiscus_NC_035579 #0.356731 , ((Palythoa_mizigama_NC_046403 #25.9564 , Palythoa_mutuki_NC_046404 #999 ) #928.234 , Sphenopus_marsupialis_NC_046406 #24.6638 ) #24.5525 ) #24.5035 ) #26.9642 ) #926.449 , ((Parazoanthus_elongatus_NC_046405 #0.0001 , Parazoanthus_swiftii_NC_046475 #897.685 ) #0.0001 , Savalia_savaglia_DQ825686 #0.0001 ) #23.5349 ) #0.0001 , (Microzoanthus_occultus_NC_046401 #540.056 , Nanozoanthus_harenaceus_NC_046402 #0.000953052 ) #0.0566868 ) #0.0001 ) #0.00615313 ) #0.0001 , (((((((Actinia_equina_NC_039929 #25.3337 , Actinia_tenebrosa_NC_044902 #25.8594 ) #25.8044 , (((Anemonia_manjano_NC_037178 #25.3223 , Heteractis_aurora_NC_047219 #25.1333 ) #0.366176 , (Anemonia_sulcata_NC_049065 #25.7953 , Anemonia_viridis_NC_037177 #25.8119 ) #25.4728 ) #25.47 , Anthopleura_anjunae_NC_030274 #0.0001 ) #25.7949 ) #25.8562 , Phymanthus_crucifer_NC_027614 #0.354332 ) #25.5576 , ((((Bolocera_sp__BZ_2016_KU507297 #25.4735 , (Bolocera_tuediae_NC_022470 #25.6879 , Liponema_brevicorne_NC_047221 #25.5002 ) #25.4713 ) #25.4729 , (Entacmaea_quadricolor_NC_049066 #0.180709 , Isosicyonis_striata_KR051006 #25.5659 ) #25.4752 ) #25.4015 , Stichodactyla_haddoni_MW760873 #0.164603 ) #25.5338 , Epiactis_japonica_NC_047217 #1.83964 ) #25.5111 ) #0.062243 , ((((Alicia_sansibarensis_NC_027610 #0.0625522 , (Haloclava_producta_NC_047218 #0.0001 , Sagartia_ornata_NC_027615 #0.0001 ) #976.341 ) #0.0001 , (Diadumene_lineata_NC_045515 #0.0001 , Metridium_senile_NC_000933 #0.0001 ) #0.275442 ) #0.0001 , Paraphelliactis_xishaensis_MT997141 #0.193608 ) #0.0001 , Antholoba_achates_NC_027611 #0.142922 ) #0.0001 ) #0.0001 , Halcampoides_purpureus_NC_027612 #0.318259 ) #0.0122625 , Nematostella_sp__JVK_2006_NC_008164 #0.0265553 ) #424.539 ) #0.0834519 , (((((((Aurelia_aurita_NC_008446 #0.0621373 , (Aurelia_coerulea_NC_046792 #0.0265437 , Aurelia_limbata_NC_046691 #0.0001 ) #0.0001 ) #0.0326967 , (Aurelia_sp__3_sensu_Dawson_et_al___2005__LC005413 #0.0001 , Aurelia_sp__4_sensu_Dawson_et_al___2005__LC005414 #0.18872 ) #0.708203 ) #885.084 , ((Nemopilema_nomurai_NC_035740 #463.741 , Rhopilema_esculentum_NC_035741 #0.0799902 ) #0.00698545 , Stomolophus_sp__CG_2019_MK157198 #0.296191 ) #0.00134953 ) #955.054 , Cassiopea_xamachana_NC_016466 #0.0187952 ) #999 , (Chrysaora_pacifica_NC_046775 #0.0001 , Chrysaora_quinquecirrha_NC_020459 #0.0001 ) #0.0056795 ) #888.901 , ((((((Blackfordia_virginica_NC_053946 #0.028305 , Eutima_sp__BMK_2020_MW066348 #0.009464 ) #314.42 , Laomedea_flexuosa_JN700945 #0.0762111 ) #978.065 , (Clava_multicornis_NC_016465 #0.0135195 , (Turritopsis_dohrnii_NC_031213 #519.201 , Turritopsis_lata_NC_056128 #0.0304345 ) #0.203261 ) #0.0001 ) #0.0001 , Spirocodon_saltatrix_NC_049868 #0.109929 ) #999 , ((Hydra_oligactis_NC_010214 #999 , Hydra_sinensis_NC_021406 #0.371536 ) #918.903 , Nemalecium_lighti_MZ457217 #0.662337 ) #999 ) #0.147602 , (Craspedacusta_sowerbii_NC_018537 #0.000529338 , Cubaia_aphrodite_NC_016467 #0.00227519 ) #0.00203379 ) #401.427 ) #124.551 , (Haliclystus_antarcticus_NC_030337 #0.0001 , OU744455_1_OU744455_1__OU744455_1 #0.0469663 ) #0.0108885 ) #0.0001 ) #129.558 , Carijoa_riisei_NC_048963 #801.01 ) #27.4967 , (Sarcophyton_trocheliophorum_MK994517 #0.0001 , (((Sinularia_acuta_MW987591 #0.0001 , (Sinularia_maxima_MN485891 #0.0001 , Sinularia_penghuensis_MW256412 #0.0001 ) #0.0001 ) #0.0001 , Sinularia_cf__cruciata_GS_2016_NC_034318 #0.0001 ) #0.0001 , (Sinularia_ceramensis_NC_044122 #0.0001 , Sinularia_peculiaris_NC_018379 #0.0001 ) #0.0001 ) #0.0001 ) #0.0001 ) #0.240277 , (((((((Antillogorgia_bipinnata_NC_008157 #0.0001 , ((((Eugorgia_mutabilis_NC_035665 #0.0001 , Leptogorgia_alba_NC_035669 #0.0001 ) #0.0001 , (Leptogorgia_hebes_MN052675 #999 , Leptogorgia_virgulata_MK301586 #0.0001 ) #0.0001 ) #0.0001 , Leptogorgia_sp__USNM_1437444_KY559412 #0.0001 ) #0.0001 , Pacifigorgia_cairnsi_NC_035668 #0.0001 ) #0.0001 ) #0.0001 , (((Leptogorgia_capverdensis_NC_035663 #0.0001 , Leptogorgia_sarmentosa_NC_035670 #0.0001 ) #0.0001 , Leptogorgia_gaini_KY559404 #0.0001 ) #0.0001 , Leptogorgia_cf__palma_AP_2017_KY559406 #0.0001 ) #0.0001 ) #0.0001 , (Eunicella_albicans_NC_035666 #0.0001 , (Eunicella_cavolini_NC_035667 #0.0001 , Eunicella_verrucosa_MW588805 #0.0001 ) #0.248568 ) #0.0001 ) #0.0001 , (Calicogorgia_granulosa_NC_023345 #0.568816 , Euplexaura_crassa_NC_020458 #826.808 ) #0.0001 ) #0.0001 , Muricea_crassa_NC_029697 #0.0001 ) #0.0001 , ((((((Dendronephthya_castanea_NC_023343 #0.0001 , Dendronephthya_mollis_NC_020456 #0.0001 ) #0.0001 , Dendronephthya_gigantea_NC_013573 #0.0001 ) #0.0001 , (Dendronephthya_putteri_NC_036022 #0.0001 , Dendronephthya_suensoni_NC_022809 #0.0001 ) #0.0001 ) #0.0001 , Scleronephthya_gracillimum_NC_023344 #0.0001 ) #0.0001 , (Echinogorgia_complexa_NC_020457 #0.135656 , Paramuricea_clavata_NC_034749 #0.0001 ) #0.0001 ) #0.0001 , Trachythela_sp__YZ_2021_MW238423 #0.0001 ) #0.0001 ) #0.0001 , (Incrustatus_comauensis_MT254531 #0.0001 , Telestula_humilis_MT254527 #0.0001 ) #0.0001 ) #0.0001 ) #0.0001 , Briareum_asbestinum_NC_008073 #837.027 ) #0.0001 , (Telestula_cf__batoni_AP_2020_MT254530 #0.0001 , Telestula_septentrionalis_MT254532 #0.0001 ) #0.0001 ) #0.0001 , (((Anthomastus_sp__USNM_1081145_KM015353 #0.0001 , Anthomastus_sp__USNM_1171062_KM015352 #0.0001 ) #0.0001 , (((Corallium_japonicum_AB595189 #0.0001 , Corallium_rubrum_NC_022864 #0.0001 ) #0.0001 , (Pleurocorallium_elatius_NC_022804 #0.0001 , Pleurocorallium_konojoi_NC_015406 #0.0001 ) #848.718 ) #0.0001 , Sibogagorgia_cauliflora_NC_026193 #845.94 ) #0.0001 ) #0.0001 , Paraminabea_aldersladei_NC_018790 #0.542795 ) #0.12878 ) #842.929 , (((((((Anthoptilum_grandiflorum_NC_044086 #0.0001 , Anthoptilum_sp__1_RH_2019_MK919656 #0.0001 ) #0.0001 , (Umbellula_huxleyi_MK919668 #0.0001 , Umbellula_sp__1_RH_2019_MK919669 #999 ) #0.0001 ) #0.0001 , Virgularia_mirabilis_NC_044091 #816.777 ) #0.0001 , (Funiculina_quadrangularis_NC_044078 #825.022 , ((Kophobelemnon_sp__1_RH_2019_MK919660 #0.0001 , Kophobelemnon_sp__3_RH_2019_MK919661 #0.0001 ) #0.0001 , Kophobelemnon_sp__4_RH_2019_MK919662 #0.0001 ) #0.0001 ) #0.0001 ) #0.0001 , (Halipteris_cf__finmarchica_RH_2019_MK919659 #0.249639 , Umbellula_sp__2_RH_2019_MK919670 #0.0001 ) #0.0001 ) #0.0001 , ((((Distichoptilum_gracile_NC_044077 #0.0001 , (Pennatula_cf__inflata_RH_2019_MK919666 #0.0001 , Pennatula_grandis_NC_044088 #0.0001 ) #0.52174 ) #0.0001 , ((Pennatula_aculeata_NC_044087 #0.0001 , Pennatula_cf__aculeata_RH_2019_MK919664 #0.0001 ) #0.0001 , Renilla_muelleri_NC_018378 #0.0001 ) #0.0001 ) #0.0001 , Protoptilum_carpenteri_NC_044089 #0.0001 ) #0.0001 , Stylatula_elongata_NC_018380 #0.0001 ) #0.0001 ) #0.0001 , Junceella_fragilis_NC_024181 #0.0001 ) #833.318 ) #0.0001 , Heliopora_coerulea_NC_020375 #0.134802 ) #28.5373 , ((Callogorgia_cf__gracilis_EE_2018_MH719202 #0.0001 , (Plumarella_adhaerans_NC_046480 #0.0001 , Plumarella_spinosa_NC_046465 #0.0001 ) #0.0001 ) #0.0001 , Narella_hawaiiensis_NC_026192 #0.0001 ) #833.303 ) #0.0001 , Keratoisidinae_sp__BAL208_1_NC_010764 #0.0001 );

COX1:

**dS tree:**

(Acanella_arbuscula_NC_011016: 0.016789, ((((((((((((((((((((((((((((((Acropora_aculeus_KT001202: 0.000005, (Acropora_horrida_NC_022825: 0.000005, LC201842_1_LC201842_1__LC201842_1: 0.000005): 0.000005): 0.000005, LC201815_1_LC201815_1__LC201815_1: 0.000005): 0.000005, ((((Acropora_aspera_NC_022827: 0.000005, Acropora_muricata_NC_022824: 0.000005): 0.000005, LC201828_1_LC201828_1__LC201828_1: 0.000005): 0.000005, (((Acropora_digitifera_NC_022830: 0.000005, LC201849_1_LC201849_1__LC201849_1: 0.000005): 0.005768, Acropora_florida_NC_022828: 0.005767): 0.000005, Acropora_humilis_NC_022823: 0.000005): 0.000005): 0.000005, (Acropora_hyacinthus_NC_022826: 0.000005, LC201817_1_LC201817_1__LC201817_1: 0.000005): 0.000005): 0.000005): 0.000005, (Acropora_divaricata_NC_022832: 0.000005, (((Acropora_nasuta_NC_022831: 0.000005, Acropora_valida_NC_042742: 0.000005): 0.000005, LC201847_1_LC201847_1__LC201847_1: 0.000005): 0.000005, LC201820_1_LC201820_1__LC201820_1: 0.000005): 0.000005): 0.000005): 0.000005, Acropora_robusta_NC_022833: 0.000005): 0.000005, LC201813_1_LC201813_1__LC201813_1: 0.000005): 0.000005, ((Acropora_tenuis_NC_003522: 0.000005, Acropora_yongei_NC_022829: 0.000005): 0.000005, LC201816_1_LC201816_1__LC201816_1: 0.000005): 0.000005): 0.035499, (Isopora_palifera_NC_024091: 0.000017, Isopora_togianensis_NC_024089: 0.000005): 0.006395): 0.073020, ((Anacropora_matthai_NC_006898: 0.000005, Montipora_efflorescens_NC_040137: 0.000005): 0.000005, (Montipora_aequituberculata_NC_037359: 0.005905, Montipora_cactus_NC_006902: 0.011705): 0.005785): 0.107956): 0.067954, (Alveopora_japonica_NC_040136: 0.000005, Alveopora_sp__MFL_2014_KJ634271: 0.000005): 0.152425): 0.009864, (Astreopora_explanata_NC_024090: 0.000005, Astreopora_myriophthalma_NC_024092: 0.000005): 0.064593): 0.076766, ((Agaricia_humilis_NC_008160: 0.031820, Pavona_decussata_NC_026527: 0.042986): 0.209423, (Fimbriaphyllia_ancora_NC_015641: 0.064305, Galaxea_fascicularis_NC_029696: 0.022458): 0.130175): 0.027381): 0.050615, ((Pseudosiderastrea_formosa_NC_026530: 0.019351, Pseudosiderastrea_tayamai_NC_026531: 0.005202): 0.045342, Siderastrea_radians_NC_008167: 0.079484): 0.066307): 0.000028, ((((((Dendrophyllia_arbuscula_NC_027590: 0.005889, Tubastraea_coccinea_NC_026025: 0.005923): 0.011872, ((Tubastraea_diaphana_MK959042: 0.000005, Tubastraea_tagusensis_NC_030352: 0.000005): 0.005872, Tubastraea_micranthus_MK959041: 0.000005): 0.000005): 0.000005, Dendrophyllia_cribrosa_NC_026026: 0.017784): 0.023749, Turbinaria_peltata_NC_024671: 0.057838): 0.043858, (((Goniopora_columna_NC_015643: 0.005894, Goniopora_djiboutiensis_NC_045931: 0.005935): 0.012259, Goniopora_lobata_MN795054: 0.005621): 0.059019, ((Porites_fontanesii_NC_037434: 0.030741, ((Porites_panamensis_NC_024182: 0.005904, Porites_sverdrupi_KU956960: 0.000005): 0.000005, Porites_porites_NC_008166: 0.005906): 0.024397): 0.007987, (Porites_harrisoni_NC_037435: 0.005902, ((Porites_lobata_NC_030186: 0.000011, Porites_okinawensis_NC_015644: 0.005893): 0.005882, (Porites_lutea_NC_029695: 0.000005, Porites_rus_NC_027526: 0.000005): 0.000005): 0.000005): 0.021493): 0.039502): 0.084332): 0.100489, Fungiacyathus_stephanus_NC_015640: 0.091804): 0.020057): 0.316697, Gardineria_hawaiiensis_NC_056290: 0.275319): 0.125796, (((((Amplexidiscus_fenestrafer_NC_027101: 0.018179, (Discosoma_sp__MH308003: 0.012079, (Rhodactis_indosinensis_NC_027103: 0.011984, Rhodactis_sp__CASIZ_171755_NC_008158: 0.000005): 0.005965): 0.000005): 0.000011, (Discosoma_nummiforme_NC_027100: 0.012072, ((Discosoma_sp__CASIZ_168915_NC_008071: 0.000005, Discosoma_sp__CASIZ_168916_NC_008072: 0.000005): 0.005875, Platyzoanthus_mussoides_NC_027104: 0.000011): 0.005683): 0.031020): 0.068239, (Pseudocorynactis_sp__SIO_KP938437: 0.088014, (Ricordea_florida_NC_008159: 0.009617, Ricordea_yuma_NC_027106: 0.046803): 0.110335): 0.048414): 0.046635, Corynactis_californica_NC_027102: 0.128145): 0.046903, Corallimorphus_profundus_KP938440: 0.124016): 0.115763): 0.007274, ((((((Astrangia_sp__JVK_2006_NC_008161: 0.071063, (((Colpophyllia_natans_NC_008162: 0.022738, Mussa_angulosa_NC_008163: 0.053714): 0.013579, ((((((Dipsastraea_favus_NC_046690: 0.020408, Dipsastraea_rotumana_NC_044074: 0.030024): 0.000005, Favites_abdita_NC_035879: 0.020311): 0.000005, Platygyra_carnosa_NC_020049: 0.023457): 0.013026, (Favites_pentagona_NC_034916: 0.076405, Hydnophora_exesa_NC_042795: 0.026460): 0.000005): 0.000005, ((Orbicella_annularis_NC_007224: 0.000005, Orbicella_faveolata_NC_007226: 0.000005): 0.000005, Orbicella_franksi_NC_007225: 0.000005): 0.056308): 0.011112, Echinophyllia_aspera_NC_040169: 0.054905): 0.033466): 0.095790, Plesiastrea_versipora_NC_042481: 0.134672): 0.036300): 0.074157, (Polycyathus_sp__MFL_2011_NC_015642: 0.012780, Psammocora_nierstraszi_MT576637: 0.159725): 0.181922): 0.199714, Madrepora_oculata_NC_018364: 0.566734): 0.219869, (Madracis_myriaster_NC_011160: 0.137006, ((Pocillopora_damicornis_NC_009797: 0.000005, Pocillopora_grandis_NC_009798: 0.000005): 0.030971, ((Seriatopora_caliendrum_NC_010245: 0.000010, Seriatopora_hystrix_NC_010244: 0.000005): 0.044416, Stylophora_pistillata_NC_011162: 0.006015): 0.132495): 0.050912): 0.396840): 0.233494, ((Desmophyllum_dianthus_NC_034275: 0.000010, Desmophyllum_pertusum_NC_015143: 0.000017): 0.000005, Solenosmilia_variabilis_NC_025472: 0.000005): 0.529637): 0.398081, Paraconotrochus_antarcticus_NC_056275: 1.937705): 0.768137): 0.597214, ((((((Antipathes_cf__dichotoma_NB_2020_MT318841: 0.000000, Stichopathes_abyssicola_MT318856: 0.000000): 0.120818, (Phanopathes_sp__NB_2020_MT318852: 0.171475, Stichopathes_sp__n__NB_2020_MT318857: 0.145786): 0.022650): 0.165463, ((Stichopathes_luetkeni_NC_018377: 0.000005, Stichopathes_sp__SCBUCN_8850_MZ157399: 0.006050): 0.018351, Stichopathes_sp__SCBUCN_8849_MZ157400: 0.006207): 0.385472): 0.033754, ((Myriopathes_japonica_NC_027667: 0.021879, Tanacetipathes_thamnea_NC_046843: 0.000005): 0.039512, Tylopathes_sp__n__NB_2020_MT318859: 0.049324): 0.248341): 0.045232, (((((((Bathypathes_sp__1_NB_2020_MT318844: 0.000005, Bathypathes_sp__n__2_NB_2020_MT318842: 0.000005): 0.000005, Stauropathes_arctica_MT318854: 0.000005): 0.000005, Bathypathes_sp__n__3_NB_2020_MT318843: 0.005477): 0.000011, Stauropathes_cf__punctata_NB_2020_MT318855: 0.005489): 0.000005, Telopathes_sp__NB_2020_MT318858: 0.016570): 0.029890, (Dendrobathypathes_sp__n__NB_2020_MT318845: 0.049301, ((Parantipathes_cf__hirondelle_NB_2020_MT318849: 0.000001, Parantipathes_hirondelle_MT318850: 0.000001): 0.000005, (Parantipathes_sp__NB_2020_MT318851: 0.000000, Sibopathes_cf__macrospina_NB_2020_MT318853: 0.000000): 0.005402): 0.023096): 0.010345): 0.100097, (Chrysopathes_formosa_NC_008411: 0.018917, Trissopathes_cf__tetracrada_NB_2020_MT318840: 0.025018): 0.046161): 0.056234): 0.000021, (Leiopathes_cf__glaberrima_NB_2020_MT318846: 0.000005, Leiopathes_expansa_MT318847: 0.000005): 0.111134): 0.314145): 0.033639, (Epizoanthus_illoricatus_NC_046474: 0.209078, (((Hydrozoanthus_gracilis_NC_046400: 0.053756, ((Neozoanthus_aff__uchina_AP_2020_MN873592: 0.011773, ((Zoanthus_cf__pulchellus_AP_2020_MN873599: 0.000000, ((Zoanthus_cf__sociatus_AP_2020_MN873600: 0.000000, Zoanthus_sociatus_NC_046476: 0.000000): 0.000000, Zoanthus_sp__AP_2020_MN873602: 0.000000): 0.005177): 0.000000, Zoanthus_sansibaricus_NC_035578: 0.000000): 0.057968): 0.025916, (Palythoa_heliodiscus_NC_035579: 0.020598, ((Palythoa_mizigama_NC_046403: 0.010174, Palythoa_mutuki_NC_046404: 0.000011): 0.000000, Sphenopus_marsupialis_NC_046406: 0.000011): 0.000000): 0.008795): 0.047868): 0.041165, ((Parazoanthus_elongatus_NC_046405: 0.015082, Parazoanthus_swiftii_NC_046475: 0.009786): 0.015309, Savalia_savaglia_DQ825686: 0.025773): 0.001393): 0.125773, (Microzoanthus_occultus_NC_046401: 0.833208, Nanozoanthus_harenaceus_NC_046402: 0.580134): 0.697306): 0.065624): 0.661735): 0.017605, (((((((Actinia_equina_NC_039929: 0.000005, Actinia_tenebrosa_NC_044902: 0.000005): 0.027023, (((Anemonia_manjano_NC_037178: 0.005688, Heteractis_aurora_NC_047219: 0.011444): 0.031588, (Anemonia_sulcata_NC_049065: 0.000005, Anemonia_viridis_NC_037177: 0.000005): 0.014929): 0.012318, Anthopleura_anjunae_NC_030274: 0.029612): 0.000005): 0.006492, Phymanthus_crucifer_NC_027614: 0.153268): 0.057469, ((((Bolocera_sp__BZ_2016_KU507297: 0.011268, (Bolocera_tuediae_NC_022470: 0.000005, Liponema_brevicorne_NC_047221: 0.000005): 0.005637): 0.000005, (Entacmaea_quadricolor_NC_049066: 0.040484, Isosicyonis_striata_KR051006: 0.022785): 0.000005): 0.099245, Stichodactyla_haddoni_MW760873: 0.100961): 0.000005, Epiactis_japonica_NC_047217: 0.198797): 0.009999): 0.110734, ((((Alicia_sansibarensis_NC_027610: 0.767676, (Haloclava_producta_NC_047218: 0.029591, Sagartia_ornata_NC_027615: 0.019797): 0.048665): 0.000005, (Diadumene_lineata_NC_045515: 0.128312, Metridium_senile_NC_000933: 0.059393): 0.048480): 0.126520, Paraphelliactis_xishaensis_MT997141: 0.067867): 0.032126, Antholoba_achates_NC_027611: 0.178882): 0.326266): 0.158214, Halcampoides_purpureus_NC_027612: 0.175124): 0.110459, Nematostella_sp__JVK_2006_NC_008164: 0.771824): 0.396749): 1.736180, (((((((Aurelia_aurita_NC_008446: 0.551145, (Aurelia_coerulea_NC_046792: 0.549212, Aurelia_limbata_NC_046691: 0.265659): 0.302356): 0.039809, (Aurelia_sp__3_sensu_Dawson_et_al___2005__LC005413: 0.450359, Aurelia_sp__4_sensu_Dawson_et_al___2005__LC005414: 0.635769): 0.184892): 0.411313, ((Nemopilema_nomurai_NC_035740: 1.155471, Rhopilema_esculentum_NC_035741: 0.934361): 0.053060, Stomolophus_sp__CG_2019_MK157198: 0.965043): 0.432332): 0.044830, Cassiopea_xamachana_NC_016466: 1.209254): 0.072571, (Chrysaora_pacifica_NC_046775: 0.021476, Chrysaora_quinquecirrha_NC_020459: 0.014373): 1.102846): 0.351379, ((((((Blackfordia_virginica_NC_053946: 1.192532, Eutima_sp__BMK_2020_MW066348: 1.597283): 0.000131, Laomedea_flexuosa_JN700945: 1.293898): 0.168893, (Clava_multicornis_NC_016465: 1.186128, (Turritopsis_dohrnii_NC_031213: 0.650014, Turritopsis_lata_NC_056128: 0.585021): 0.623368): 0.099080): 0.000040, Spirocodon_saltatrix_NC_049868: 1.293589): 0.063230, ((Hydra_oligactis_NC_010214: 1.303726, Hydra_sinensis_NC_021406: 0.696354): 0.667841, Nemalecium_lighti_MZ457217: 1.281677): 0.054595): 0.000080, (Craspedacusta_sowerbii_NC_018537: 63.164523, Cubaia_aphrodite_NC_016467: 1.226947): 0.000343): 0.000198): 0.000218, (Haliclystus_antarcticus_NC_030337: 1.561347, OU744455_1_OU744455_1__OU744455_1: 0.000005): 9.618719): 0.000172): 1.817984, Carijoa_riisei_NC_048963: 0.000030): 0.105106, (Sarcophyton_trocheliophorum_MK994517: 0.134656, (((Sinularia_acuta_MW987591: 0.011696, (Sinularia_maxima_MN485891: 0.011748, Sinularia_penghuensis_MW256412: 0.005821): 0.000005): 0.017480, Sinularia_cf__cruciata_GS_2016_NC_034318: 0.000005): 0.017269, (Sinularia_ceramensis_NC_044122: 0.011464, Sinularia_peculiaris_NC_018379: 0.000005): 0.005958): 0.037744): 0.027935): 0.017675, (((((((Antillogorgia_bipinnata_NC_008157: 0.048980, ((((Eugorgia_mutabilis_NC_035665: 0.017484, Leptogorgia_alba_NC_035669: 0.005724): 0.005851, (Leptogorgia_hebes_MN052675: 0.005793, Leptogorgia_virgulata_MK301586: 0.011626): 0.000005): 0.000005, Leptogorgia_sp__USNM_1437444_KY559412: 0.029549): 0.004764, Pacifigorgia_cairnsi_NC_035668: 0.075477): 0.013657): 0.004965, (((Leptogorgia_capverdensis_NC_035663: 0.000005, Leptogorgia_sarmentosa_NC_035670: 0.000005): 0.026058, Leptogorgia_gaini_KY559404: 0.016474): 0.005101, Leptogorgia_cf__palma_AP_2017_KY559406: 0.045393): 0.007671): 0.000000, (Eunicella_albicans_NC_035666: 0.011645, (Eunicella_cavolini_NC_035667: 0.000005, Eunicella_verrucosa_MW588805: 0.000005): 0.017419): 0.011630): 0.006647, (Calicogorgia_granulosa_NC_023345: 0.718255, Euplexaura_crassa_NC_020458: 0.046683): 0.000033): 0.000010, Muricea_crassa_NC_029697: 0.042549): 0.006134, ((((((Dendronephthya_castanea_NC_023343: 0.000005, Dendronephthya_mollis_NC_020456: 0.000005): 0.000005, Dendronephthya_gigantea_NC_013573: 0.000005): 0.000005, (Dendronephthya_putteri_NC_036022: 0.000005, Dendronephthya_suensoni_NC_022809: 0.000005): 0.000005): 0.054716, Scleronephthya_gracillimum_NC_023344: 0.037540): 0.050442, (Echinogorgia_complexa_NC_020457: 0.012282, Paramuricea_clavata_NC_034749: 0.149690): 0.081997): 0.012571, Trachythela_sp__YZ_2021_MW238423: 0.045835): 0.005754): 0.022688, (Incrustatus_comauensis_MT254531: 0.017722, Telestula_humilis_MT254527: 0.017387): 0.023001): 0.002617): 0.104696, Briareum_asbestinum_NC_008073: 0.113611): 0.021956, (Telestula_cf__batoni_AP_2020_MT254530: 0.000005, Telestula_septentrionalis_MT254532: 0.022636): 0.012981): 0.023354, (((Anthomastus_sp__USNM_1081145_KM015353: 0.044511, Anthomastus_sp__USNM_1171062_KM015352: 0.020090): 0.046679, (((Corallium_japonicum_AB595189: 0.011213, Corallium_rubrum_NC_022864: 0.022755): 0.046382, (Pleurocorallium_elatius_NC_022804: 0.000011, Pleurocorallium_konojoi_NC_015406: 0.000001): 0.107394): 0.000005, Sibogagorgia_cauliflora_NC_026193: 0.052013): 0.058199): 0.012522, Paraminabea_aldersladei_NC_018790: 0.076335): 0.072962): 0.015404, (((((((Anthoptilum_grandiflorum_NC_044086: 0.000005, Anthoptilum_sp__1_RH_2019_MK919656: 0.005596): 0.011409, (Umbellula_huxleyi_MK919668: 0.000011, Umbellula_sp__1_RH_2019_MK919669: 0.005558): 0.016620): 0.005642, Virgularia_mirabilis_NC_044091: 0.109059): 0.011170, (Funiculina_quadrangularis_NC_044078: 0.034076, ((Kophobelemnon_sp__1_RH_2019_MK919660: 0.000005, Kophobelemnon_sp__3_RH_2019_MK919661: 0.000011): 0.029359, Kophobelemnon_sp__4_RH_2019_MK919662: 0.011312): 0.040622): 0.000000): 0.000005, (Halipteris_cf__finmarchica_RH_2019_MK919659: 0.046786, Umbellula_sp__2_RH_2019_MK919670: 0.035000): 0.005257): 0.007409, ((((Distichoptilum_gracile_NC_044077: 0.033431, (Pennatula_cf__inflata_RH_2019_MK919666: 0.011116, Pennatula_grandis_NC_044088: 0.000005): 0.039966): 0.006624, ((Pennatula_aculeata_NC_044087: 0.000005, Pennatula_cf__aculeata_RH_2019_MK919664: 0.000005): 0.021944, Renilla_muelleri_NC_018378: 0.106745): 0.086845): 0.000005, Protoptilum_carpenteri_NC_044089: 0.022515): 0.005286, Stylatula_elongata_NC_018380: 0.070866): 0.015394): 0.019420, Junceella_fragilis_NC_024181: 0.110662): 0.051228): 0.005730, Heliopora_coerulea_NC_020375: 0.101495): 0.010575, ((Callogorgia_cf__gracilis_EE_2018_MH719202: 0.022059, (Plumarella_adhaerans_NC_046480: 0.000005, Plumarella_spinosa_NC_046465: 0.000005): 0.020770): 0.010563, Narella_hawaiiensis_NC_026192: 0.025801): 0.045099): 0.025395, Keratoisidinae_sp__BAL208_1_NC_010764: 0.011705);

**dN tree:**

(Acanella_arbuscula_NC_011016: 0.001862, ((((((((((((((((((((((((((((((Acropora_aculeus_KT001202: 0.000000, (Acropora_horrida_NC_022825: 0.000000, LC201842_1_LC201842_1__LC201842_1: 0.000000): 0.000000): 0.000000, LC201815_1_LC201815_1__LC201815_1: 0.000000): 0.000000, ((((Acropora_aspera_NC_022827: 0.000000, Acropora_muricata_NC_022824: 0.000000): 0.000000, LC201828_1_LC201828_1__LC201828_1: 0.000000): 0.000000, (((Acropora_digitifera_NC_022830: 0.000000, LC201849_1_LC201849_1__LC201849_1: 0.000000): 0.000001, Acropora_florida_NC_022828: 0.000001): 0.000000, Acropora_humilis_NC_022823: 0.000000): 0.000000): 0.000000, (Acropora_hyacinthus_NC_022826: 0.000000, LC201817_1_LC201817_1__LC201817_1: 0.000000): 0.000000): 0.000000): 0.000000, (Acropora_divaricata_NC_022832: 0.000000, (((Acropora_nasuta_NC_022831: 0.000000, Acropora_valida_NC_042742: 0.000000): 0.000000, LC201847_1_LC201847_1__LC201847_1: 0.000000): 0.000000, LC201820_1_LC201820_1__LC201820_1: 0.000000): 0.000000): 0.000000): 0.000000, Acropora_robusta_NC_022833: 0.000000): 0.000000, LC201813_1_LC201813_1__LC201813_1: 0.000000): 0.000000, ((Acropora_tenuis_NC_003522: 0.000000, Acropora_yongei_NC_022829: 0.000000): 0.000000, LC201816_1_LC201816_1__LC201816_1: 0.000000): 0.000000): 0.003717, (Isopora_palifera_NC_024091: 0.003705, Isopora_togianensis_NC_024089: 0.000000): 0.000001): 0.000007, ((Anacropora_matthai_NC_006898: 0.000000, Montipora_efflorescens_NC_040137: 0.000000): 0.000000, (Montipora_aequituberculata_NC_037359: 0.007535, Montipora_cactus_NC_006902: 0.001872): 0.000001): 0.001862): 0.001845, (Alveopora_japonica_NC_040136: 0.000000, Alveopora_sp__MFL_2014_KJ634271: 0.000000): 0.000015): 0.001848, (Astreopora_explanata_NC_024090: 0.000000, Astreopora_myriophthalma_NC_024092: 0.000000): 0.005563): 0.001869, ((Agaricia_humilis_NC_008160: 0.000003, Pavona_decussata_NC_026527: 0.000004): 0.003743, (Fimbriaphyllia_ancora_NC_015641: 0.000006, Galaxea_fascicularis_NC_029696: 0.000002): 0.000013): 0.000003): 0.000005, ((Pseudosiderastrea_formosa_NC_026530: 0.001824, Pseudosiderastrea_tayamai_NC_026531: 0.001820): 0.000005, Siderastrea_radians_NC_008167: 0.011006): 0.000007): 0.001929, ((((((Dendrophyllia_arbuscula_NC_027590: 0.000001, Tubastraea_coccinea_NC_026025: 0.000001): 0.000001, ((Tubastraea_diaphana_MK959042: 0.000000, Tubastraea_tagusensis_NC_030352: 0.000000): 0.000001, Tubastraea_micranthus_MK959041: 0.000000): 0.000000): 0.000000, Dendrophyllia_cribrosa_NC_026026: 0.005561): 0.009313, Turbinaria_peltata_NC_024671: 0.003721): 0.001845, (((Goniopora_columna_NC_015643: 0.000001, Goniopora_djiboutiensis_NC_045931: 0.000001): 0.000001, Goniopora_lobata_MN795054: 0.000001): 0.001846, ((Porites_fontanesii_NC_037434: 0.000003, ((Porites_panamensis_NC_024182: 0.000001, Porites_sverdrupi_KU956960: 0.000000): 0.000000, Porites_porites_NC_008166: 0.000001): 0.001837): 0.000001, (Porites_harrisoni_NC_037435: 0.000001, ((Porites_lobata_NC_030186: 0.001828, Porites_okinawensis_NC_015644: 0.000001): 0.000001, (Porites_lutea_NC_029695: 0.000000, Porites_rus_NC_027526: 0.000000): 0.000000): 0.000000): 0.000002): 0.003685): 0.000008): 0.003631, Fungiacyathus_stephanus_NC_015640: 0.003809): 0.000002): 0.004640, Gardineria_hawaiiensis_NC_056290: 0.013958): 0.005376, (((((Amplexidiscus_fenestrafer_NC_027101: 0.009162, (Discosoma_sp__MH308003: 0.000001, (Rhodactis_indosinensis_NC_027103: 0.001815, Rhodactis_sp__CASIZ_171755_NC_008158: 0.000000): 0.000001): 0.000000): 0.001802, (Discosoma_nummiforme_NC_027100: 0.001834, ((Discosoma_sp__CASIZ_168915_NC_008071: 0.000000, Discosoma_sp__CASIZ_168916_NC_008072: 0.000000): 0.000001, Platyzoanthus_mussoides_NC_027104: 0.001840): 0.001835): 0.000003): 0.000007, (Pseudocorynactis_sp__SIO_KP938437: 0.001789, (Ricordea_florida_NC_008159: 0.000001, Ricordea_yuma_NC_027106: 0.000005): 0.001796): 0.001780): 0.000005, Corynactis_californica_NC_027102: 0.010859): 0.001409, Corallimorphus_profundus_KP938440: 0.009431): 0.005468): 0.000001, ((((((Astrangia_sp__JVK_2006_NC_008161: 0.003378, (((Colpophyllia_natans_NC_008162: 0.001716, Mussa_angulosa_NC_008163: 0.001737): 0.001711, ((((((Dipsastraea_favus_NC_046690: 0.000002, Dipsastraea_rotumana_NC_044074: 0.000003): 0.000000, Favites_abdita_NC_035879: 0.000002): 0.000000, Platygyra_carnosa_NC_020049: 0.000002): 0.001704, (Favites_pentagona_NC_034916: 0.000008, Hydnophora_exesa_NC_042795: 0.000003): 0.000000): 0.000000, ((Orbicella_annularis_NC_007224: 0.000000, Orbicella_faveolata_NC_007226: 0.000000): 0.000000, Orbicella_franksi_NC_007225: 0.000000): 0.005191): 0.000001, Echinophyllia_aspera_NC_040169: 0.003417): 0.000003): 0.000010, Plesiastrea_versipora_NC_042481: 0.001703): 0.000004): 0.000007, (Polycyathus_sp__MFL_2011_NC_015642: 0.000001, Psammocora_nierstraszi_MT576637: 0.008639): 0.001687): 0.008471, Madrepora_oculata_NC_018364: 0.006939): 0.001936, (Madracis_myriaster_NC_011160: 0.004504, ((Pocillopora_damicornis_NC_009797: 0.000000, Pocillopora_grandis_NC_009798: 0.000000): 0.002814, ((Seriatopora_caliendrum_NC_010245: 0.001758, Seriatopora_hystrix_NC_010244: 0.000000): 0.000004, Stylophora_pistillata_NC_011162: 0.000001): 0.007748): 0.001693): 0.010664): 0.004437, ((Desmophyllum_dianthus_NC_034275: 0.001802, Desmophyllum_pertusum_NC_015143: 0.003607): 0.000000, Solenosmilia_variabilis_NC_025472: 0.000000): 0.005516): 0.013149, Paraconotrochus_antarcticus_NC_056275: 0.024807): 0.002053): 0.008801, ((((((Antipathes_cf__dichotoma_NB_2020_MT318841: 0.000002, Stichopathes_abyssicola_MT318856: 0.000002): 0.000012, (Phanopathes_sp__NB_2020_MT318852: 0.003888, Stichopathes_sp__n__NB_2020_MT318857: 0.000015): 0.000002): 0.001957, ((Stichopathes_luetkeni_NC_018377: 0.000000, Stichopathes_sp__SCBUCN_8850_MZ157399: 0.000001): 0.000002, Stichopathes_sp__SCBUCN_8849_MZ157400: 0.000001): 0.003717): 0.000003, ((Myriopathes_japonica_NC_027667: 0.000002, Tanacetipathes_thamnea_NC_046843: 0.000000): 0.000004, Tylopathes_sp__n__NB_2020_MT318859: 0.002031): 0.000025): 0.000005, (((((((Bathypathes_sp__1_NB_2020_MT318844: 0.000000, Bathypathes_sp__n__2_NB_2020_MT318842: 0.000000): 0.000000, Stauropathes_arctica_MT318854: 0.000000): 0.000000, Bathypathes_sp__n__3_NB_2020_MT318843: 0.000001): 0.001940, Stauropathes_cf__punctata_NB_2020_MT318855: 0.000001): 0.000000, Telopathes_sp__NB_2020_MT318858: 0.000002): 0.000003, (Dendrobathypathes_sp__n__NB_2020_MT318845: 0.003972, ((Parantipathes_cf__hirondelle_NB_2020_MT318849: 0.000002, Parantipathes_hirondelle_MT318850: 0.000002): 0.000000, (Parantipathes_sp__NB_2020_MT318851: 0.000002, Sibopathes_cf__macrospina_NB_2020_MT318853: 0.000002): 0.000001): 0.000002): 0.000001): 0.001980, (Chrysopathes_formosa_NC_008411: 0.005920, Trissopathes_cf__tetracrada_NB_2020_MT318840: 0.000003): 0.001983): 0.001982): 0.001971, (Leiopathes_cf__glaberrima_NB_2020_MT318846: 0.000000, Leiopathes_expansa_MT318847: 0.000000): 0.000011): 0.018408): 0.000003, (Epizoanthus_illoricatus_NC_046474: 0.002556, (((Hydrozoanthus_gracilis_NC_046400: 0.000005, ((Neozoanthus_aff__uchina_AP_2020_MN873592: 0.002123, ((Zoanthus_cf__pulchellus_AP_2020_MN873599: 0.000002, ((Zoanthus_cf__sociatus_AP_2020_MN873600: 0.000002, Zoanthus_sociatus_NC_046476: 0.000002): 0.000002, Zoanthus_sp__AP_2020_MN873602: 0.000002): 0.000001): 0.000002, Zoanthus_sansibaricus_NC_035578: 0.000002): 0.004164): 0.012817, (Palythoa_heliodiscus_NC_035579: 0.002102, ((Palythoa_mizigama_NC_046403: 0.002118, Palythoa_mutuki_NC_046404: 0.002114): 0.000002, Sphenopus_marsupialis_NC_046406: 0.002111): 0.000002): 0.000001): 0.000005): 0.007980, ((Parazoanthus_elongatus_NC_046405: 0.002216, Parazoanthus_swiftii_NC_046475: 0.000001): 0.000002, Savalia_savaglia_DQ825686: 0.008872): 0.005412): 0.000013, (Microzoanthus_occultus_NC_046401: 0.008550, Nanozoanthus_harenaceus_NC_046402: 0.010511): 0.004139): 0.005699): 0.009384): 0.002877, (((((((Actinia_equina_NC_039929: 0.000000, Actinia_tenebrosa_NC_044902: 0.000000): 0.000003, (((Anemonia_manjano_NC_037178: 0.000001, Heteractis_aurora_NC_047219: 0.000001): 0.001923, (Anemonia_sulcata_NC_049065: 0.000000, Anemonia_viridis_NC_037177: 0.000000): 0.000001): 0.000001, Anthopleura_anjunae_NC_030274: 0.000003): 0.000000): 0.000001, Phymanthus_crucifer_NC_027614: 0.001903): 0.000006, ((((Bolocera_sp__BZ_2016_KU507297: 0.000001, (Bolocera_tuediae_NC_022470: 0.000000, Liponema_brevicorne_NC_047221: 0.000000): 0.000001): 0.000000, (Entacmaea_quadricolor_NC_049066: 0.003830, Isosicyonis_striata_KR051006: 0.003835): 0.000000): 0.000010, Stichodactyla_haddoni_MW760873: 0.000010): 0.000000, Epiactis_japonica_NC_047217: 0.000020): 0.000001): 0.001944, ((((Alicia_sansibarensis_NC_027610: 0.020903, (Haloclava_producta_NC_047218: 0.003627, Sagartia_ornata_NC_027615: 0.000002): 0.001769): 0.000000, (Diadumene_lineata_NC_045515: 0.003605, Metridium_senile_NC_000933: 0.001835): 0.003625): 0.001854, Paraphelliactis_xishaensis_MT997141: 0.001831): 0.001853, Antholoba_achates_NC_027611: 0.020918): 0.000033): 0.000016, Halcampoides_purpureus_NC_027612: 0.004132): 0.003682, Nematostella_sp__JVK_2006_NC_008164: 0.021785): 0.007940): 0.015270, (((((((Aurelia_aurita_NC_008446: 0.000055, (Aurelia_coerulea_NC_046792: 0.001891, Aurelia_limbata_NC_046691: 0.007609): 0.001915): 0.001923, (Aurelia_sp__3_sensu_Dawson_et_al___2005__LC005413: 0.001893, Aurelia_sp__4_sensu_Dawson_et_al___2005__LC005414: 0.000064): 0.000018): 0.004343, ((Nemopilema_nomurai_NC_035740: 0.001867, Rhopilema_esculentum_NC_035741: 0.001875): 0.008036, Stomolophus_sp__CG_2019_MK157198: 0.001362): 0.000043): 0.000004, Cassiopea_xamachana_NC_016466: 0.066525): 0.009277, (Chrysaora_pacifica_NC_046775: 0.000002, Chrysaora_quinquecirrha_NC_020459: 0.000001): 0.010496): 0.030262, ((((((Blackfordia_virginica_NC_053946: 0.006059, Eutima_sp__BMK_2020_MW066348: 0.005311): 0.008796, Laomedea_flexuosa_JN700945: 0.006050): 0.002851, (Clava_multicornis_NC_016465: 0.033597, (Turritopsis_dohrnii_NC_031213: 0.000065, Turritopsis_lata_NC_056128: 0.001877): 0.010996): 0.003843): 0.002145, Spirocodon_saltatrix_NC_049868: 0.017726): 0.004992, ((Hydra_oligactis_NC_010214: 0.009293, Hydra_sinensis_NC_021406: 0.010948): 0.038089, Nemalecium_lighti_MZ457217: 0.076637): 0.004468): 0.010744, (Craspedacusta_sowerbii_NC_018537: 0.021690, Cubaia_aphrodite_NC_016467: 0.004839): 0.028031): 0.019658): 0.016924, (Haliclystus_antarcticus_NC_030337: 0.003668, OU744455_1_OU744455_1__OU744455_1: 0.000000): 0.015068): 0.012703): 0.054558, Carijoa_riisei_NC_048963: 0.001818): 0.000011, (Sarcophyton_trocheliophorum_MK994517: 0.000013, (((Sinularia_acuta_MW987591: 0.000001, (Sinularia_maxima_MN485891: 0.000001, Sinularia_penghuensis_MW256412: 0.000001): 0.000000): 0.001811, Sinularia_cf__cruciata_GS_2016_NC_034318: 0.000000): 0.000002, (Sinularia_ceramensis_NC_044122: 0.000001, Sinularia_peculiaris_NC_018379: 0.000000): 0.001828): 0.000004): 0.003660): 0.000002, (((((((Antillogorgia_bipinnata_NC_008157: 0.000005, ((((Eugorgia_mutabilis_NC_035665: 0.000002, Leptogorgia_alba_NC_035669: 0.000001): 0.001821, (Leptogorgia_hebes_MN052675: 0.000001, Leptogorgia_virgulata_MK301586: 0.000001): 0.000000): 0.000000, Leptogorgia_sp__USNM_1437444_KY559412: 0.000003): 0.000000, Pacifigorgia_cairnsi_NC_035668: 0.001817): 0.000001): 0.000000, (((Leptogorgia_capverdensis_NC_035663: 0.000000, Leptogorgia_sarmentosa_NC_035670: 0.000000): 0.000003, Leptogorgia_gaini_KY559404: 0.000002): 0.001825, Leptogorgia_cf__palma_AP_2017_KY559406: 0.000005): 0.000001): 0.000002, (Eunicella_albicans_NC_035666: 0.003722, (Eunicella_cavolini_NC_035667: 0.000000, Eunicella_verrucosa_MW588805: 0.000000): 0.001791): 0.016830): 0.003909, (Calicogorgia_granulosa_NC_023345: 0.002339, Euplexaura_crassa_NC_020458: 0.012527): 0.005512): 0.001602, Muricea_crassa_NC_029697: 0.003655): 0.000001, ((((((Dendronephthya_castanea_NC_023343: 0.000000, Dendronephthya_mollis_NC_020456: 0.000000): 0.000000, Dendronephthya_gigantea_NC_013573: 0.000000): 0.000000, (Dendronephthya_putteri_NC_036022: 0.000000, Dendronephthya_suensoni_NC_022809: 0.000000): 0.000000): 0.000005, Scleronephthya_gracillimum_NC_023344: 0.001854): 0.000005, (Echinogorgia_complexa_NC_020457: 0.000001, Paramuricea_clavata_NC_034749: 0.000015): 0.003678): 0.001841, Trachythela_sp__YZ_2021_MW238423: 0.001846): 0.001834): 0.000002, (Incrustatus_comauensis_MT254531: 0.007387, Telestula_humilis_MT254527: 0.001839): 0.001832): 0.000000): 0.005607, Briareum_asbestinum_NC_008073: 0.003776): 0.001843, (Telestula_cf__batoni_AP_2020_MT254530: 0.000000, Telestula_septentrionalis_MT254532: 0.001868): 0.000001): 0.000002, (((Anthomastus_sp__USNM_1081145_KM015353: 0.007674, Anthomastus_sp__USNM_1171062_KM015352: 0.001899): 0.000005, (((Corallium_japonicum_AB595189: 0.000001, Corallium_rubrum_NC_022864: 0.000002): 0.003793, (Pleurocorallium_elatius_NC_022804: 0.001941, Pleurocorallium_konojoi_NC_015406: 0.000002): 0.001917): 0.000000, Sibogagorgia_cauliflora_NC_026193: 0.009597): 0.000006): 0.001870, Paraminabea_aldersladei_NC_018790: 0.000008): 0.003729): 0.005728, (((((((Anthoptilum_grandiflorum_NC_044086: 0.000000, Anthoptilum_sp__1_RH_2019_MK919656: 0.003758): 0.000001, (Umbellula_huxleyi_MK919668: 0.001871, Umbellula_sp__1_RH_2019_MK919669: 0.001875): 0.000002): 0.003733, Virgularia_mirabilis_NC_044091: 0.013334): 0.000001, (Funiculina_quadrangularis_NC_044078: 0.007570, ((Kophobelemnon_sp__1_RH_2019_MK919660: 0.000000, Kophobelemnon_sp__3_RH_2019_MK919661: 0.001857): 0.001866, Kophobelemnon_sp__4_RH_2019_MK919662: 0.000001): 0.003773): 0.000002): 0.000000, (Halipteris_cf__finmarchica_RH_2019_MK919659: 0.005670, Umbellula_sp__2_RH_2019_MK919670: 0.003790): 0.001867): 0.001958, ((((Distichoptilum_gracile_NC_044077: 0.005705, (Pennatula_cf__inflata_RH_2019_MK919666: 0.000001, Pennatula_grandis_NC_044088: 0.000000): 0.001881): 0.001902, ((Pennatula_aculeata_NC_044087: 0.000000, Pennatula_cf__aculeata_RH_2019_MK919664: 0.000000): 0.009660, Renilla_muelleri_NC_018378: 0.000011): 0.004020): 0.000000, Protoptilum_carpenteri_NC_044089: 0.005631): 0.000001, Stylatula_elongata_NC_018380: 0.001890): 0.003628): 0.009442, Junceella_fragilis_NC_024181: 0.003653): 0.000005): 0.000001, Heliopora_coerulea_NC_020375: 0.007620): 0.003876, ((Callogorgia_cf__gracilis_EE_2018_MH719202: 0.001867, (Plumarella_adhaerans_NC_046480: 0.000000, Plumarella_spinosa_NC_046465: 0.000000): 0.003743): 0.000001, Narella_hawaiiensis_NC_026192: 0.003743): 0.001858): 0.000003, Keratoisidinae_sp__BAL208_1_NC_010764: 0.001854);

**w ratios as labels for TreeView:**

(Acanella_arbuscula_NC_011016 #0.110907 , ((((((((((((((((((((((((((((((Acropora_aculeus_KT001202 #0.0001 , (Acropora_horrida_NC_022825 #0.0001 , LC201842_1_LC201842_1__LC201842_1 #0.0001 ) #0.0001 ) #0.0001 , LC201815_1_LC201815_1__LC201815_1 #0.0001 ) #0.0001 , ((((Acropora_aspera_NC_022827 #0.0001 , Acropora_muricata_NC_022824 #0.0001 ) #0.0001 , LC201828_1_LC201828_1__LC201828_1 #0.0001 ) #0.0001 , (((Acropora_digitifera_NC_022830 #0.0001 , LC201849_1_LC201849_1__LC201849_1 #0.0001 ) #0.0001 , Acropora_florida_NC_022828 #0.0001 ) #0.0001 , Acropora_humilis_NC_022823 #0.0001 ) #0.0001 ) #0.0001 , (Acropora_hyacinthus_NC_022826 #0.0001 , LC201817_1_LC201817_1__LC201817_1 #0.0001 ) #0.0001 ) #0.0001 ) #0.0001 , (Acropora_divaricata_NC_022832 #0.0001 , (((Acropora_nasuta_NC_022831 #0.0001 , Acropora_valida_NC_042742 #0.0001 ) #0.0001 , LC201847_1_LC201847_1__LC201847_1 #0.0001 ) #0.0001 , LC201820_1_LC201820_1__LC201820_1 #0.0001 ) #0.0001 ) #0.0001 ) #0.0001 , Acropora_robusta_NC_022833 #0.0001 ) #0.0001 , LC201813_1_LC201813_1__LC201813_1 #0.0001 ) #0.0001 , ((Acropora_tenuis_NC_003522 #0.0001 , Acropora_yongei_NC_022829 #0.0001 ) #0.0001 , LC201816_1_LC201816_1__LC201816_1 #0.0001 ) #0.0001 ) #0.10472 , (Isopora_palifera_NC_024091 #219.819 , Isopora_togianensis_NC_024089 #0.0001 ) #0.0001 ) #0.0001 , ((Anacropora_matthai_NC_006898 #0.0001 , Montipora_efflorescens_NC_040137 #0.0001 ) #0.0001 , (Montipora_aequituberculata_NC_037359 #1.27606 , Montipora_cactus_NC_006902 #0.159934 ) #0.0001 ) #0.0172456 ) #0.0271542 , (Alveopora_japonica_NC_040136 #0.0001 , Alveopora_sp__MFL_2014_KJ634271 #0.0001 ) #0.0001 ) #0.187352 , (Astreopora_explanata_NC_024090 #0.0001 , Astreopora_myriophthalma_NC_024092 #0.0001 ) #0.0861279 ) #0.0243489 , ((Agaricia_humilis_NC_008160 #0.0001 , Pavona_decussata_NC_026527 #0.0001 ) #0.0178752 , (Fimbriaphyllia_ancora_NC_015641 #0.0001 , Galaxea_fascicularis_NC_029696 #0.0001 ) #0.0001 ) #0.0001 ) #0.0001 , ((Pseudosiderastrea_formosa_NC_026530 #0.0942729 , Pseudosiderastrea_tayamai_NC_026531 #0.349808 ) #0.0001 , Siderastrea_radians_NC_008167 #0.138464 ) #0.0001 ) #68.6209 , ((((((Dendrophyllia_arbuscula_NC_027590 #0.0001 , Tubastraea_coccinea_NC_026025 #0.0001 ) #0.0001 , ((Tubastraea_diaphana_MK959042 #0.0001 , Tubastraea_tagusensis_NC_030352 #0.0001 ) #0.0001 , Tubastraea_micranthus_MK959041 #0.0001 ) #0.0001 ) #0.0001 , Dendrophyllia_cribrosa_NC_026026 #0.312696 ) #0.392154 , Turbinaria_peltata_NC_024671 #0.0643277 ) #0.0420656 , (((Goniopora_columna_NC_015643 #0.0001 , Goniopora_djiboutiensis_NC_045931 #0.0001 ) #0.0001 , Goniopora_lobata_MN795054 #0.0001 ) #0.0312776 , ((Porites_fontanesii_NC_037434 #0.0001 , ((Porites_panamensis_NC_024182 #0.0001 , Porites_sverdrupi_KU956960 #0.0001 ) #0.0001 , Porites_porites_NC_008166 #0.0001 ) #0.0753071 ) #0.0001 , (Porites_harrisoni_NC_037435 #0.0001 , ((Porites_lobata_NC_030186 #172.642 , Porites_okinawensis_NC_015644 #0.0001 ) #0.0001 , (Porites_lutea_NC_029695 #0.0001 , Porites_rus_NC_027526 #0.0001 ) #0.0001 ) #0.0001 ) #0.0001 ) #0.0932772 ) #0.0001 ) #0.0361334 , Fungiacyathus_stephanus_NC_015640 #0.0414931 ) #0.0001 ) #0.0146521 , Gardineria_hawaiiensis_NC_056290 #0.0506959 ) #0.0427328 , (((((Amplexidiscus_fenestrafer_NC_027101 #0.503982 , (Discosoma_sp__MH308003 #0.0001 , (Rhodactis_indosinensis_NC_027103 #0.151472 , Rhodactis_sp__CASIZ_171755_NC_008158 #0.0001 ) #0.0001 ) #0.0001 ) #159.877 , (Discosoma_nummiforme_NC_027100 #0.151923 , ((Discosoma_sp__CASIZ_168915_NC_008071 #0.0001 , Discosoma_sp__CASIZ_168916_NC_008072 #0.0001 ) #0.0001 , Platyzoanthus_mussoides_NC_027104 #172.905 ) #0.322907 ) #0.0001 ) #0.0001 , (Pseudocorynactis_sp__SIO_KP938437 #0.0203259 , (Ricordea_florida_NC_008159 #0.0001 , Ricordea_yuma_NC_027106 #0.0001 ) #0.0162774 ) #0.0367666 ) #0.0001 , Corynactis_californica_NC_027102 #0.0847406 ) #0.0300339 , Corallimorphus_profundus_KP938440 #0.0760463 ) #0.0472331 ) #0.0001 , ((((((Astrangia_sp__JVK_2006_NC_008161 #0.0475317 , (((Colpophyllia_natans_NC_008162 #0.0754827 , Mussa_angulosa_NC_008163 #0.0323322 ) #0.125976 , ((((((Dipsastraea_favus_NC_046690 #0.0001 , Dipsastraea_rotumana_NC_044074 #0.0001 ) #0.0001 , Favites_abdita_NC_035879 #0.0001 ) #0.0001 , Platygyra_carnosa_NC_020049 #0.0001 ) #0.130791 , (Favites_pentagona_NC_034916 #0.0001 , Hydnophora_exesa_NC_042795 #0.0001 ) #0.0001 ) #0.0001 , ((Orbicella_annularis_NC_007224 #0.0001 , Orbicella_faveolata_NC_007226 #0.0001 ) #0.0001 , Orbicella_franksi_NC_007225 #0.0001 ) #0.0921934 ) #0.0001 , Echinophyllia_aspera_NC_040169 #0.0622429 ) #0.0001 ) #0.0001 , Plesiastrea_versipora_NC_042481 #0.0126442 ) #0.0001 ) #0.0001 , (Polycyathus_sp__MFL_2011_NC_015642 #0.0001 , Psammocora_nierstraszi_MT576637 #0.0540862 ) #0.00927109 ) #0.0424178 , Madrepora_oculata_NC_018364 #0.012243 ) #0.00880465 , (Madracis_myriaster_NC_011160 #0.0328727 , ((Pocillopora_damicornis_NC_009797 #0.0001 , Pocillopora_grandis_NC_009798 #0.0001 ) #0.0908707 , ((Seriatopora_caliendrum_NC_010245 #168.598 , Seriatopora_hystrix_NC_010244 #0.0001 ) #0.0001 , Stylophora_pistillata_NC_011162 #0.0001 ) #0.05848 ) #0.0332539 ) #0.0268718 ) #0.019001 , ((Desmophyllum_dianthus_NC_034275 #172.459 , Desmophyllum_pertusum_NC_015143 #216.997 ) #0.0001 , Solenosmilia_variabilis_NC_025472 #0.0001 ) #0.0104138 ) #0.0330302 , Paraconotrochus_antarcticus_NC_056275 #0.0128023 ) #0.00267276 ) #0.0147373 , ((((((Antipathes_cf__dichotoma_NB_2020_MT318841 #4.01756 , Stichopathes_abyssicola_MT318856 #4.08665 ) #0.0001 , (Phanopathes_sp__NB_2020_MT318852 #0.0226762 , Stichopathes_sp__n__NB_2020_MT318857 #0.0001 ) #0.0001 ) #0.0118285 , ((Stichopathes_luetkeni_NC_018377 #0.0001 , Stichopathes_sp__SCBUCN_8850_MZ157399 #0.0001 ) #0.0001 , Stichopathes_sp__SCBUCN_8849_MZ157400 #0.0001 ) #0.00964351 ) #0.0001 , ((Myriopathes_japonica_NC_027667 #0.0001 , Tanacetipathes_thamnea_NC_046843 #0.0001 ) #0.0001 , Tylopathes_sp__n__NB_2020_MT318859 #0.041181 ) #0.0001 ) #0.0001 , (((((((Bathypathes_sp__1_NB_2020_MT318844 #0.0001 , Bathypathes_sp__n__2_NB_2020_MT318842 #0.0001 ) #0.000100001 , Stauropathes_arctica_MT318854 #0.0001 ) #0.0001 , Bathypathes_sp__n__3_NB_2020_MT318843 #0.0001 ) #180.4 , Stauropathes_cf__punctata_NB_2020_MT318855 #0.0001 ) #0.0001 , Telopathes_sp__NB_2020_MT318858 #0.0001 ) #0.0001 , (Dendrobathypathes_sp__n__NB_2020_MT318845 #0.0805566 , ((Parantipathes_cf__hirondelle_NB_2020_MT318849 #2.90132 , Parantipathes_hirondelle_MT318850 #2.77838 ) #0.0001 , (Parantipathes_sp__NB_2020_MT318851 #3.44488 , Sibopathes_cf__macrospina_NB_2020_MT318853 #3.34873 ) #0.0001 ) #0.0001 ) #0.0001 ) #0.0197767 , (Chrysopathes_formosa_NC_008411 #0.312935 , Trissopathes_cf__tetracrada_NB_2020_MT318840 #0.0001 ) #0.0429494 ) #0.0352412 ) #94.9014 , (Leiopathes_cf__glaberrima_NB_2020_MT318846 #0.0001 , Leiopathes_expansa_MT318847 #0.0001 ) #0.0001 ) #0.0585967 ) #0.0001 , (Epizoanthus_illoricatus_NC_046474 #0.0122228 , (((Hydrozoanthus_gracilis_NC_046400 #0.0001 , ((Neozoanthus_aff__uchina_AP_2020_MN873592 #0.180327 , ((Zoanthus_cf__pulchellus_AP_2020_MN873599 #9.52044 , ((Zoanthus_cf__sociatus_AP_2020_MN873600 #7.44364 , Zoanthus_sociatus_NC_046476 #7.06541 ) #8.51928 , Zoanthus_sp__AP_2020_MN873602 #7.02671 ) #0.0001 ) #9.17087 , Zoanthus_sansibaricus_NC_035578 #6.9893 ) #0.0718298 ) #0.494533 , (Palythoa_heliodiscus_NC_035579 #0.102044 , ((Palythoa_mizigama_NC_046403 #0.208181 , Palythoa_mutuki_NC_046404 #190.622 ) #10.2491 , Sphenopus_marsupialis_NC_046406 #191.742 ) #15.9937 ) #0.0001 ) #0.0001 ) #0.193849 , ((Parazoanthus_elongatus_NC_046405 #0.146904 , Parazoanthus_swiftii_NC_046475 #0.0001 ) #0.0001 , Savalia_savaglia_DQ825686 #0.344245 ) #3.88436 ) #0.0001 , (Microzoanthus_occultus_NC_046401 #0.0102621 , Nanozoanthus_harenaceus_NC_046402 #0.018119 ) #0.00593642 ) #0.0868448 ) #0.0141808 ) #0.163404 , (((((((Actinia_equina_NC_039929 #0.0001 , Actinia_tenebrosa_NC_044902 #0.0001 ) #0.0001 , (((Anemonia_manjano_NC_037178 #0.0001 , Heteractis_aurora_NC_047219 #0.0001 ) #0.060877 , (Anemonia_sulcata_NC_049065 #0.0001 , Anemonia_viridis_NC_037177 #0.0001 ) #0.0001 ) #0.0001 , Anthopleura_anjunae_NC_030274 #0.0001 ) #0.0001 ) #0.0001 , Phymanthus_crucifer_NC_027614 #0.0124135 ) #0.0001 , ((((Bolocera_sp__BZ_2016_KU507297 #0.0001 , (Bolocera_tuediae_NC_022470 #0.0001 , Liponema_brevicorne_NC_047221 #0.0001 ) #0.0001 ) #0.0001 , (Entacmaea_quadricolor_NC_049066 #0.0946057 , Isosicyonis_striata_KR051006 #0.168318 ) #0.0001 ) #0.0001 , Stichodactyla_haddoni_MW760873 #0.0001 ) #0.0001 , Epiactis_japonica_NC_047217 #0.0001 ) #0.0001 ) #0.0175546 , ((((Alicia_sansibarensis_NC_027610 #0.0272294 , (Haloclava_producta_NC_047218 #0.122571 , Sagartia_ornata_NC_027615 #0.0001 ) #0.0363523 ) #0.0001 , (Diadumene_lineata_NC_045515 #0.0280966 , Metridium_senile_NC_000933 #0.0308905 ) #0.0747756 ) #0.0146524 , Paraphelliactis_xishaensis_MT997141 #0.0269854 ) #0.0576773 , Antholoba_achates_NC_027611 #0.116937 ) #0.0001 ) #0.0001 , Halcampoides_purpureus_NC_027612 #0.0235918 ) #0.0333307 , Nematostella_sp__JVK_2006_NC_008164 #0.0282251 ) #0.0200127 ) #0.00879502 , (((((((Aurelia_aurita_NC_008446 #0.0001 , (Aurelia_coerulea_NC_046792 #0.00344375 , Aurelia_limbata_NC_046691 #0.0286416 ) #0.00633282 ) #0.0483013 , (Aurelia_sp__3_sensu_Dawson_et_al___2005__LC005413 #0.00420372 , Aurelia_sp__4_sensu_Dawson_et_al___2005__LC005414 #0.0001 ) #0.0001 ) #0.010559 , ((Nemopilema_nomurai_NC_035740 #0.00161601 , Rhopilema_esculentum_NC_035741 #0.0020071 ) #0.151453 , Stomolophus_sp__CG_2019_MK157198 #0.00141155 ) #0.0001 ) #0.0001 , Cassiopea_xamachana_NC_016466 #0.055013 ) #0.127829 , (Chrysaora_pacifica_NC_046775 #0.0001 , Chrysaora_quinquecirrha_NC_020459 #0.0001 ) #0.00951688 ) #0.0861236 , ((((((Blackfordia_virginica_NC_053946 #0.00508103 , Eutima_sp__BMK_2020_MW066348 #0.00332498 ) #67.1729 , Laomedea_flexuosa_JN700945 #0.00467595 ) #0.0168779 , (Clava_multicornis_NC_016465 #0.0283246 , (Turritopsis_dohrnii_NC_031213 #0.0001 , Turritopsis_lata_NC_056128 #0.00320826 ) #0.0176399 ) #0.0387865 ) #53.3815 , Spirocodon_saltatrix_NC_049868 #0.0137031 ) #0.0789452 , ((Hydra_oligactis_NC_010214 #0.00712837 , Hydra_sinensis_NC_021406 #0.0157219 ) #0.0570327 , Nemalecium_lighti_MZ457217 #0.0597946 ) #0.0818469 ) #135.061 , (Craspedacusta_sowerbii_NC_018537 #0.000343386 , Cubaia_aphrodite_NC_016467 #0.00394382 ) #81.8192 ) #99.1184 ) #77.456 , (Haliclystus_antarcticus_NC_030337 #0.00234954 , OU744455_1_OU744455_1__OU744455_1 #0.0001 ) #0.00156657 ) #73.8237 ) #0.0300101 , Carijoa_riisei_NC_048963 #61.3678 ) #0.0001 , (Sarcophyton_trocheliophorum_MK994517 #0.0001 , (((Sinularia_acuta_MW987591 #0.0001 , (Sinularia_maxima_MN485891 #0.0001 , Sinularia_penghuensis_MW256412 #0.0001 ) #0.0001 ) #0.103609 , Sinularia_cf__cruciata_GS_2016_NC_034318 #0.0001 ) #0.0001 , (Sinularia_ceramensis_NC_044122 #0.0001 , Sinularia_peculiaris_NC_018379 #0.0001 ) #0.306802 ) #0.0001 ) #0.131011 ) #0.0001 , (((((((Antillogorgia_bipinnata_NC_008157 #0.0001 , ((((Eugorgia_mutabilis_NC_035665 #0.0001 , Leptogorgia_alba_NC_035669 #0.0001 ) #0.31128 , (Leptogorgia_hebes_MN052675 #0.0001 , Leptogorgia_virgulata_MK301586 #0.0001 ) #0.0001 ) #0.0001 , Leptogorgia_sp__USNM_1437444_KY559412 #0.0001 ) #0.0001 , Pacifigorgia_cairnsi_NC_035668 #0.0240704 ) #0.0001 ) #0.0001 , (((Leptogorgia_capverdensis_NC_035663 #0.0001 , Leptogorgia_sarmentosa_NC_035670 #0.0001 ) #0.0001 , Leptogorgia_gaini_KY559404 #0.0001 ) #0.357788 , Leptogorgia_cf__palma_AP_2017_KY559406 #0.0001 ) #0.0001 ) #13.9416 , (Eunicella_albicans_NC_035666 #0.319638 , (Eunicella_cavolini_NC_035667 #0.0001 , Eunicella_verrucosa_MW588805 #0.0001 ) #0.102813 ) #1.44716 ) #0.588058 , (Calicogorgia_granulosa_NC_023345 #0.00325649 , Euplexaura_crassa_NC_020458 #0.268333 ) #167.695 ) #156.268 , Muricea_crassa_NC_029697 #0.0858947 ) #0.0001 , ((((((Dendronephthya_castanea_NC_023343 #0.0001 , Dendronephthya_mollis_NC_020456 #0.0001 ) #0.0001 , Dendronephthya_gigantea_NC_013573 #0.0001 ) #0.0001 , (Dendronephthya_putteri_NC_036022 #0.0001 , Dendronephthya_suensoni_NC_022809 #0.0001 ) #0.0001 ) #0.0001 , Scleronephthya_gracillimum_NC_023344 #0.0493977 ) #0.0001 , (Echinogorgia_complexa_NC_020457 #0.0001 , Paramuricea_clavata_NC_034749 #0.0001 ) #0.0448541 ) #0.146455 , Trachythela_sp__YZ_2021_MW238423 #0.0402847 ) #0.318767 ) #0.0001 , (Incrustatus_comauensis_MT254531 #0.416847 , Telestula_humilis_MT254527 #0.105779 ) #0.0796355 ) #0.0001 ) #0.0535562 , Briareum_asbestinum_NC_008073 #0.0332403 ) #0.0839617 , (Telestula_cf__batoni_AP_2020_MT254530 #0.0001 , Telestula_septentrionalis_MT254532 #0.0825107 ) #0.0001 ) #0.0001 , (((Anthomastus_sp__USNM_1081145_KM015353 #0.172413 , Anthomastus_sp__USNM_1171062_KM015352 #0.094519 ) #0.0001 , (((Corallium_japonicum_AB595189 #0.0001 , Corallium_rubrum_NC_022864 #0.0001 ) #0.0817776 , (Pleurocorallium_elatius_NC_022804 #181.535 , Pleurocorallium_konojoi_NC_015406 #2.04826 ) #0.0178471 ) #0.0001 , Sibogagorgia_cauliflora_NC_026193 #0.184514 ) #0.0001 ) #0.149366 , Paraminabea_aldersladei_NC_018790 #0.0001 ) #0.0511101 ) #0.371817 , (((((((Anthoptilum_grandiflorum_NC_044086 #0.0001 , Anthoptilum_sp__1_RH_2019_MK919656 #0.671489 ) #0.0001 , (Umbellula_huxleyi_MK919668 #177.273 , Umbellula_sp__1_RH_2019_MK919669 #0.337377 ) #0.0001 ) #0.661654 , Virgularia_mirabilis_NC_044091 #0.122267 ) #0.0001 , (Funiculina_quadrangularis_NC_044078 #0.222157 , ((Kophobelemnon_sp__1_RH_2019_MK919660 #0.0001 , Kophobelemnon_sp__3_RH_2019_MK919661 #175.737 ) #0.0635647 , Kophobelemnon_sp__4_RH_2019_MK919662 #0.0001 ) #0.0928824 ) #11.2855 ) #0.0001 , (Halipteris_cf__finmarchica_RH_2019_MK919659 #0.121191 , Umbellula_sp__2_RH_2019_MK919670 #0.10828 ) #0.35518 ) #0.264276 , ((((Distichoptilum_gracile_NC_044077 #0.170647 , (Pennatula_cf__inflata_RH_2019_MK919666 #0.0001 , Pennatula_grandis_NC_044088 #0.0001 ) #0.0470635 ) #0.287085 , ((Pennatula_aculeata_NC_044087 #0.0001 , Pennatula_cf__aculeata_RH_2019_MK919664 #0.0001 ) #0.440195 , Renilla_muelleri_NC_018378 #0.0001 ) #0.0462905 ) #0.0001 , Protoptilum_carpenteri_NC_044089 #0.250115 ) #0.0001 , Stylatula_elongata_NC_018380 #0.0266753 ) #0.235714 ) #0.486181 , Junceella_fragilis_NC_024181 #0.033011 ) #0.0001 ) #0.0001 , Heliopora_coerulea_NC_020375 #0.0750751 ) #0.366555 , ((Callogorgia_cf__gracilis_EE_2018_MH719202 #0.0846215 , (Plumarella_adhaerans_NC_046480 #0.0001 , Plumarella_spinosa_NC_046465 #0.0001 ) #0.180225 ) #0.0001 , Narella_hawaiiensis_NC_026192 #0.145054 ) #0.0412034 ) #0.0001 , Keratoisidinae_sp__BAL208_1_NC_010764 #0.158419 );

COX2:

**dS tree:**

(Acanella_arbuscula_NC_011016: 0.016787, ((((((((((((((((((((((((((((((Acropora_aculeus_KT001202: 0.000001, (Acropora_horrida_NC_022825: 0.008073, LC201842_1_LC201842_1__LC201842_1: 0.000001): 0.000001): 0.000001, LC201815_1_LC201815_1__LC201815_1: 0.000001): 0.000001, ((((Acropora_aspera_NC_022827: 0.000001, Acropora_muricata_NC_022824: 0.000001): 0.000001, LC201828_1_LC201828_1__LC201828_1: 0.000001): 0.000001, (((Acropora_digitifera_NC_022830: 0.000001, LC201849_1_LC201849_1__LC201849_1: 0.000001): 0.000001, Acropora_florida_NC_022828: 0.000001): 0.000001, Acropora_humilis_NC_022823: 0.000001): 0.000001): 0.000001, (Acropora_hyacinthus_NC_022826: 0.000001, LC201817_1_LC201817_1__LC201817_1: 0.000001): 0.000001): 0.000001): 0.000001, (Acropora_divaricata_NC_022832: 0.000001, (((Acropora_nasuta_NC_022831: 0.000001, Acropora_valida_NC_042742: 0.000001): 0.000001, LC201847_1_LC201847_1__LC201847_1: 0.000001): 0.000001, LC201820_1_LC201820_1__LC201820_1: 0.000001): 0.016322): 0.000001): 0.000001, Acropora_robusta_NC_022833: 0.000001): 0.000001, LC201813_1_LC201813_1__LC201813_1: 0.008073): 0.000001, ((Acropora_tenuis_NC_003522: 0.000001, Acropora_yongei_NC_022829: 0.000001): 0.000001, LC201816_1_LC201816_1__LC201816_1: 0.000001): 0.000001): 0.000006, (Isopora_palifera_NC_024091: 0.000002, Isopora_togianensis_NC_024089: 0.000001): 0.016357): 0.084677, ((Anacropora_matthai_NC_006898: 0.000001, Montipora_efflorescens_NC_040137: 0.000001): 0.008006, (Montipora_aequituberculata_NC_037359: 0.007979, Montipora_cactus_NC_006902: 0.000000): 0.000001): 0.017191): 0.025517, (Alveopora_japonica_NC_040136: 0.007463, Alveopora_sp__MFL_2014_KJ634271: 0.017180): 0.145398): 0.000006, (Astreopora_explanata_NC_024090: 0.008254, Astreopora_myriophthalma_NC_024092: 0.000006): 0.060206): 0.028090, ((Agaricia_humilis_NC_008160: 0.047251, Pavona_decussata_NC_026527: 0.043999): 0.146780, (Fimbriaphyllia_ancora_NC_015641: 0.058997, Galaxea_fascicularis_NC_029696: 0.003766): 0.224890): 0.021330): 0.065616, ((Pseudosiderastrea_formosa_NC_026530: 0.054245, Pseudosiderastrea_tayamai_NC_026531: 0.000025): 0.118820, Siderastrea_radians_NC_008167: 0.146210): 0.047958): 0.011517, ((((((Dendrophyllia_arbuscula_NC_027590: 0.008130, Tubastraea_coccinea_NC_026025: 0.000003): 0.016659, ((Tubastraea_diaphana_MK959042: 0.000001, Tubastraea_tagusensis_NC_030352: 0.000001): 0.000001, Tubastraea_micranthus_MK959041: 0.000001): 0.008076): 0.016777, Dendrophyllia_cribrosa_NC_026026: 0.000006): 0.077379, Turbinaria_peltata_NC_024671: 0.053612): 0.019995, (((Goniopora_columna_NC_015643: 0.007959, Goniopora_djiboutiensis_NC_045931: 0.000001): 0.040818, Goniopora_lobata_MN795054: 0.017398): 0.021944, ((Porites_fontanesii_NC_037434: 0.040407, ((Porites_panamensis_NC_024182: 0.000006, Porites_sverdrupi_KU956960: 0.000006): 0.008164, Porites_porites_NC_008166: 0.008333): 0.052017): 0.000006, (Porites_harrisoni_NC_037435: 0.000022, ((Porites_lobata_NC_030186: 0.000006, Porites_okinawensis_NC_015644: 0.000006): 0.016660, (Porites_lutea_NC_029695: 0.000006, Porites_rus_NC_027526: 0.000006): 0.000006): 0.000006): 0.017738): 0.114792): 0.098315): 0.015105, Fungiacyathus_stephanus_NC_015640: 0.099101): 0.037432): 0.084203, Gardineria_hawaiiensis_NC_056290: 0.160232): 0.245966, (((((Amplexidiscus_fenestrafer_NC_027101: 0.008157, (Discosoma_sp__MH308003: 0.000022, (Rhodactis_indosinensis_NC_027103: 0.000001, Rhodactis_sp__CASIZ_171755_NC_008158: 0.000035): 0.000001): 0.008082): 0.016627, (Discosoma_nummiforme_NC_027100: 0.000022, ((Discosoma_sp__CASIZ_168915_NC_008071: 0.000001, Discosoma_sp__CASIZ_168916_NC_008072: 0.000001): 0.000001, Platyzoanthus_mussoides_NC_027104: 0.000001): 0.000001): 0.000029): 0.126686, (Pseudocorynactis_sp__SIO_KP938437: 0.162734, (Ricordea_florida_NC_008159: 0.000023, Ricordea_yuma_NC_027106: 0.017217): 0.106808): 0.020282): 0.023776, Corynactis_californica_NC_027102: 0.122437): 0.077810, Corallimorphus_profundus_KP938440: 0.082097): 0.088657): 0.276673, ((((((Astrangia_sp__JVK_2006_NC_008161: 0.147377, (((Colpophyllia_natans_NC_008162: 0.021042, Mussa_angulosa_NC_008163: 0.017198): 0.072776, ((((((Dipsastraea_favus_NC_046690: 0.018599, Dipsastraea_rotumana_NC_044074: 0.000006): 0.009201, Favites_abdita_NC_035879: 0.009181): 0.000006, Platygyra_carnosa_NC_020049: 0.009163): 0.000006, (Favites_pentagona_NC_034916: 0.028015, Hydnophora_exesa_NC_042795: 0.047151): 0.000006): 0.000006, ((Orbicella_annularis_NC_007224: 0.000006, Orbicella_faveolata_NC_007226: 0.000006): 0.009522, Orbicella_franksi_NC_007225: 0.000006): 0.135601): 0.000023, Echinophyllia_aspera_NC_040169: 0.018494): 0.144608): 0.000051, Plesiastrea_versipora_NC_042481: 0.411321): 0.000006): 0.181374, (Polycyathus_sp__MFL_2011_NC_015642: 0.027365, Psammocora_nierstraszi_MT576637: 0.042077): 0.000096): 0.781566, Madrepora_oculata_NC_018364: 0.949657): 0.000124, (Madracis_myriaster_NC_011160: 0.051837, ((Pocillopora_damicornis_NC_009797: 0.000006, Pocillopora_grandis_NC_009798: 0.000006): 0.064285, ((Seriatopora_caliendrum_NC_010245: 0.009446, Seriatopora_hystrix_NC_010244: 0.000006): 0.009490, Stylophora_pistillata_NC_011162: 0.000006): 0.004519): 0.151350): 0.660737): 0.139163, ((Desmophyllum_dianthus_NC_034275: 0.000022, Desmophyllum_pertusum_NC_015143: 0.000022): 0.000006, Solenosmilia_variabilis_NC_025472: 0.008382): 0.415431): 69.154116, Paraconotrochus_antarcticus_NC_056275: 69.066249): 6.128649): 0.297126, ((((((Antipathes_cf__dichotoma_NB_2020_MT318841: 0.000000, Stichopathes_abyssicola_MT318856: 0.000000): 0.234173, (Phanopathes_sp__NB_2020_MT318852: 0.024796, Stichopathes_sp__n__NB_2020_MT318857: 0.018648): 0.120531): 0.093000, ((Stichopathes_luetkeni_NC_018377: 0.008729, Stichopathes_sp__SCBUCN_8850_MZ157399: 0.000006): 0.021234, Stichopathes_sp__SCBUCN_8849_MZ157400: 0.005203): 0.425147): 0.104533, ((Myriopathes_japonica_NC_027667: 0.000000, Tanacetipathes_thamnea_NC_046843: 0.000000): 0.053959, Tylopathes_sp__n__NB_2020_MT318859: 0.028380): 0.165247): 0.133791, (((((((Bathypathes_sp__1_NB_2020_MT318844: 0.000006, Bathypathes_sp__n__2_NB_2020_MT318842: 0.000006): 0.000006, Stauropathes_arctica_MT318854: 0.000006): 0.017035, Bathypathes_sp__n__3_NB_2020_MT318843: 0.017078): 0.000006, Stauropathes_cf__punctata_NB_2020_MT318855: 0.025773): 0.000006, Telopathes_sp__NB_2020_MT318858: 0.000006): 0.025465, (Dendrobathypathes_sp__n__NB_2020_MT318845: 0.025901, ((Parantipathes_cf__hirondelle_NB_2020_MT318849: 0.000001, Parantipathes_hirondelle_MT318850: 0.000001): 0.000001, (Parantipathes_sp__NB_2020_MT318851: 0.000001, Sibopathes_cf__macrospina_NB_2020_MT318853: 0.000001): 0.000001): 0.025999): 0.018465): 0.124464, (Chrysopathes_formosa_NC_008411: 0.025937, Trissopathes_cf__tetracrada_NB_2020_MT318840: 0.000006): 0.070543): 0.000027): 0.142521, (Leiopathes_cf__glaberrima_NB_2020_MT318846: 0.000006, Leiopathes_expansa_MT318847: 0.000006): 0.086972): 0.193504): 0.105403, (Epizoanthus_illoricatus_NC_046474: 0.070777, (((Hydrozoanthus_gracilis_NC_046400: 0.117141, ((Neozoanthus_aff__uchina_AP_2020_MN873592: 0.037494, ((Zoanthus_cf__pulchellus_AP_2020_MN873599: 0.000000, ((Zoanthus_cf__sociatus_AP_2020_MN873600: 0.000000, Zoanthus_sociatus_NC_046476: 0.000000): 0.000000, Zoanthus_sp__AP_2020_MN873602: 0.000000): 0.000000): 0.000000, Zoanthus_sansibaricus_NC_035578: 0.000000): 0.033745): 0.018767, (Palythoa_heliodiscus_NC_035579: 0.007253, ((Palythoa_mizigama_NC_046403: 0.000000, Palythoa_mutuki_NC_046404: 0.007712): 0.007734, Sphenopus_marsupialis_NC_046406: 0.007815): 0.008365): 0.064129): 0.005867): 0.041526, ((Parazoanthus_elongatus_NC_046405: 0.007374, Parazoanthus_swiftii_NC_046475: 0.015458): 0.031273, Savalia_savaglia_DQ825686: 0.015876): 0.019810): 0.000006, (Microzoanthus_occultus_NC_046401: 2.433218, Nanozoanthus_harenaceus_NC_046402: 1.661777): 0.661420): 0.226209): 0.648872): 0.204516, (((((((Actinia_equina_NC_039929: 0.000006, Actinia_tenebrosa_NC_044902: 0.000006): 0.008338, (((Anemonia_manjano_NC_037178: 0.008538, Heteractis_aurora_NC_047219: 0.008359): 0.016884, (Anemonia_sulcata_NC_049065: 0.000006, Anemonia_viridis_NC_037177: 0.000022): 0.034210): 0.000006, Anthopleura_anjunae_NC_030274: 0.025723): 0.000022): 0.000006, Phymanthus_crucifer_NC_027614: 0.118090): 0.285708, ((((Bolocera_sp__BZ_2016_KU507297: 0.000002, (Bolocera_tuediae_NC_022470: 0.000002, Liponema_brevicorne_NC_047221: 0.000002): 0.000006): 0.012221, (Entacmaea_quadricolor_NC_049066: 0.034893, Isosicyonis_striata_KR051006: 0.035856): 0.314058): 0.000006, Stichodactyla_haddoni_MW760873: 0.071320): 0.050121, Epiactis_japonica_NC_047217: 0.140947): 0.009739): 0.000032, ((((Alicia_sansibarensis_NC_027610: 0.212369, (Haloclava_producta_NC_047218: 0.052241, Sagartia_ornata_NC_027615: 0.029151): 0.036802): 0.017782, (Diadumene_lineata_NC_045515: 0.397482, Metridium_senile_NC_000933: 0.063052): 0.108080): 0.161426, Paraphelliactis_xishaensis_MT997141: 0.164770): 0.050625, Antholoba_achates_NC_027611: 0.619862): 0.000006): 0.195819, Halcampoides_purpureus_NC_027612: 0.100274): 0.321188, Nematostella_sp__JVK_2006_NC_008164: 1.396273): 0.172532): 1.740927, (((((((Aurelia_aurita_NC_008446: 0.672893, (Aurelia_coerulea_NC_046792: 0.449677, Aurelia_limbata_NC_046691: 0.465667): 0.067404): 0.000321, (Aurelia_sp__3_sensu_Dawson_et_al___2005__LC005413: 0.749789, Aurelia_sp__4_sensu_Dawson_et_al___2005__LC005414: 0.547301): 0.267682): 1.399762, ((Nemopilema_nomurai_NC_035740: 0.604648, Rhopilema_esculentum_NC_035741: 0.897011): 0.000389, Stomolophus_sp__CG_2019_MK157198: 1.682353): 0.254371): 0.000088, Cassiopea_xamachana_NC_016466: 3.402451): 0.000316, (Chrysaora_pacifica_NC_046775: 0.000006, Chrysaora_quinquecirrha_NC_020459: 0.080357): 2.576174): 0.000639, ((((((Blackfordia_virginica_NC_053946: 1.233000, Eutima_sp__BMK_2020_MW066348: 1.108213): 0.182835, Laomedea_flexuosa_JN700945: 1.916357): 0.000436, (Clava_multicornis_NC_016465: 1.022556, (Turritopsis_dohrnii_NC_031213: 0.563561, Turritopsis_lata_NC_056128: 0.791174): 0.000280): 0.250909): 0.493736, Spirocodon_saltatrix_NC_049868: 1.613952): 0.803874, ((Hydra_oligactis_NC_010214: 0.207367, Hydra_sinensis_NC_021406: 1.683828): 2.723254, Nemalecium_lighti_MZ457217: 13.736406): 0.000801): 0.000396, (Craspedacusta_sowerbii_NC_018537: 1.184692, Cubaia_aphrodite_NC_016467: 69.192248): 2.897483): 0.000406): 0.000277, (Haliclystus_antarcticus_NC_030337: 0.000006, OU744455_1_OU744455_1__OU744455_1: 2.365032): 68.961549): 0.000910): 2.570471, Carijoa_riisei_NC_048963: 0.000071): 0.117437, (Sarcophyton_trocheliophorum_MK994517: 0.140715, (((Sinularia_acuta_MW987591: 0.000006, (Sinularia_maxima_MN485891: 0.000006, Sinularia_penghuensis_MW256412: 0.000006): 0.000006): 0.000006, Sinularia_cf__cruciata_GS_2016_NC_034318: 0.071021): 0.009528, (Sinularia_ceramensis_NC_044122: 0.000006, Sinularia_peculiaris_NC_018379: 0.000006): 0.036690): 0.028224): 0.027524): 0.000006, (((((((Antillogorgia_bipinnata_NC_008157: 0.036205, ((((Eugorgia_mutabilis_NC_035665: 0.009127, Leptogorgia_alba_NC_035669: 0.009153): 0.000006, (Leptogorgia_hebes_MN052675: 0.027989, Leptogorgia_virgulata_MK301586: 0.000007): 0.000006): 0.009014, Leptogorgia_sp__USNM_1437444_KY559412: 0.009411): 0.007060, Pacifigorgia_cairnsi_NC_035668: 0.130427): 0.029990): 0.000006, (((Leptogorgia_capverdensis_NC_035663: 0.000006, Leptogorgia_sarmentosa_NC_035670: 0.000006): 0.000006, Leptogorgia_gaini_KY559404: 0.008981): 0.045917, Leptogorgia_cf__palma_AP_2017_KY559406: 0.008993): 0.000006): 0.000006, (Eunicella_albicans_NC_035666: 0.004998, (Eunicella_cavolini_NC_035667: 0.000006, Eunicella_verrucosa_MW588805: 0.000006): 0.023915): 0.044295): 0.017717, (Calicogorgia_granulosa_NC_023345: 0.453403, Euplexaura_crassa_NC_020458: 0.036369): 0.000028): 0.000037, Muricea_crassa_NC_029697: 0.066753): 0.000006, ((((((Dendronephthya_castanea_NC_023343: 0.000006, Dendronephthya_mollis_NC_020456: 0.000006): 0.000006, Dendronephthya_gigantea_NC_013573: 0.000006): 0.009092, (Dendronephthya_putteri_NC_036022: 0.009106, Dendronephthya_suensoni_NC_022809: 0.000006): 0.000006): 0.039973, Scleronephthya_gracillimum_NC_023344: 0.015479): 0.130438, (Echinogorgia_complexa_NC_020457: 0.026427, Paramuricea_clavata_NC_034749: 0.047615): 0.047901): 0.000006, Trachythela_sp__YZ_2021_MW238423: 0.104625): 0.000000): 0.008821, (Incrustatus_comauensis_MT254531: 0.037945, Telestula_humilis_MT254527: 0.022272): 0.033161): 0.000006): 0.152434, Briareum_asbestinum_NC_008073: 0.184955): 0.000006, (Telestula_cf__batoni_AP_2020_MT254530: 0.018823, Telestula_septentrionalis_MT254532: 0.009116): 0.031108): 0.009058, (((Anthomastus_sp__USNM_1081145_KM015353: 0.027310, Anthomastus_sp__USNM_1171062_KM015352: 0.010738): 0.053519, (((Corallium_japonicum_AB595189: 0.008053, Corallium_rubrum_NC_022864: 0.008903): 0.072409, (Pleurocorallium_elatius_NC_022804: 0.000006, Pleurocorallium_konojoi_NC_015406: 0.016770): 0.044176): 0.000006, Sibogagorgia_cauliflora_NC_026193: 0.008891): 0.028202): 0.035444, Paraminabea_aldersladei_NC_018790: 0.032746): 0.045364): 0.031549, (((((((Anthoptilum_grandiflorum_NC_044086: 0.047291, Anthoptilum_sp__1_RH_2019_MK919656: 0.000023): 0.047207, (Umbellula_huxleyi_MK919668: 0.000006, Umbellula_sp__1_RH_2019_MK919669: 0.018189): 0.009165): 0.000023, Virgularia_mirabilis_NC_044091: 0.057116): 0.056907, (Funiculina_quadrangularis_NC_044078: 0.010423, ((Kophobelemnon_sp__1_RH_2019_MK919660: 0.000006, Kophobelemnon_sp__3_RH_2019_MK919661: 0.000006): 0.016532, Kophobelemnon_sp__4_RH_2019_MK919662: 0.022082): 0.053190): 0.000023): 0.000006, (Halipteris_cf__finmarchica_RH_2019_MK919659: 0.064812, Umbellula_sp__2_RH_2019_MK919670: 0.056981): 0.009064): 0.000000, ((((Distichoptilum_gracile_NC_044077: 0.028527, (Pennatula_cf__inflata_RH_2019_MK919666: 0.018413, Pennatula_grandis_NC_044088: 0.026423): 0.025404): 0.000006, ((Pennatula_aculeata_NC_044087: 0.008853, Pennatula_cf__aculeata_RH_2019_MK919664: 0.000006): 0.017700, Renilla_muelleri_NC_018378: 0.100283): 0.033703): 0.029066, Protoptilum_carpenteri_NC_044089: 0.036343): 0.000006, Stylatula_elongata_NC_018380: 0.035810): 0.000047): 0.043601, Junceella_fragilis_NC_024181: 0.111690): 0.051579): 0.017158, Heliopora_coerulea_NC_020375: 0.203195): 0.029323, ((Callogorgia_cf__gracilis_EE_2018_MH719202: 0.055552, (Plumarella_adhaerans_NC_046480: 0.000006, Plumarella_spinosa_NC_046465: 0.000006): 0.035722): 0.009665, Narella_hawaiiensis_NC_026192: 0.000037): 0.020514): 0.061577, Keratoisidinae_sp__BAL208_1_NC_010764: 0.020602);

**dN tree:**

(Acanella_arbuscula_NC_011016: 0.000002, ((((((((((((((((((((((((((((((Acropora_aculeus_KT001202: 0.000001, (Acropora_horrida_NC_022825: 0.000001, LC201842_1_LC201842_1__LC201842_1: 0.000001): 0.000002): 0.000001, LC201815_1_LC201815_1__LC201815_1: 0.000001): 0.000001, ((((Acropora_aspera_NC_022827: 0.000002, Acropora_muricata_NC_022824: 0.000001): 0.000001, LC201828_1_LC201828_1__LC201828_1: 0.000001): 0.000001, (((Acropora_digitifera_NC_022830: 0.000001, LC201849_1_LC201849_1__LC201849_1: 0.000001): 0.000001, Acropora_florida_NC_022828: 0.000001): 0.000001, Acropora_humilis_NC_022823: 0.000001): 0.000001): 0.000001, (Acropora_hyacinthus_NC_022826: 0.000001, LC201817_1_LC201817_1__LC201817_1: 0.000001): 0.000001): 0.000001): 0.000001, (Acropora_divaricata_NC_022832: 0.000001, (((Acropora_nasuta_NC_022831: 0.000001, Acropora_valida_NC_042742: 0.000002): 0.000001, LC201847_1_LC201847_1__LC201847_1: 0.000001): 0.000001, LC201820_1_LC201820_1__LC201820_1: 0.000002): 0.000002): 0.000001): 0.000001, Acropora_robusta_NC_022833: 0.000001): 0.000001, LC201813_1_LC201813_1__LC201813_1: 0.000001): 0.000001, ((Acropora_tenuis_NC_003522: 0.000001, Acropora_yongei_NC_022829: 0.000001): 0.000001, LC201816_1_LC201816_1__LC201816_1: 0.000001): 0.000001): 0.000000, (Isopora_palifera_NC_024091: 0.000001, Isopora_togianensis_NC_024089: 0.000001): 0.000002): 0.005288, ((Anacropora_matthai_NC_006898: 0.000002, Montipora_efflorescens_NC_040137: 0.000002): 0.000001, (Montipora_aequituberculata_NC_037359: 0.000001, Montipora_cactus_NC_006902: 0.000002): 0.000001): 0.008019): 0.000003, (Alveopora_japonica_NC_040136: 0.000001, Alveopora_sp__MFL_2014_KJ634271: 0.000002): 0.005238): 0.000000, (Astreopora_explanata_NC_024090: 0.000001, Astreopora_myriophthalma_NC_024092: 0.000000): 0.002603): 0.000003, ((Agaricia_humilis_NC_008160: 0.000005, Pavona_decussata_NC_026527: 0.005387): 0.013334, (Fimbriaphyllia_ancora_NC_015641: 0.002558, Galaxea_fascicularis_NC_029696: 0.000000): 0.005247): 0.000002): 0.000007, ((Pseudosiderastrea_formosa_NC_026530: 0.000005, Pseudosiderastrea_tayamai_NC_026531: 0.002477): 0.010184, Siderastrea_radians_NC_008167: 0.002539): 0.002572): 0.000001, ((((((Dendrophyllia_arbuscula_NC_027590: 0.000001, Tubastraea_coccinea_NC_026025: 0.000001): 0.005235, ((Tubastraea_diaphana_MK959042: 0.000001, Tubastraea_tagusensis_NC_030352: 0.000001): 0.000001, Tubastraea_micranthus_MK959041: 0.000001): 0.000001): 0.005214, Dendrophyllia_cribrosa_NC_026026: 0.000000): 0.002815, Turbinaria_peltata_NC_024671: 0.007530): 0.005233, (((Goniopora_columna_NC_015643: 0.005272, Goniopora_djiboutiensis_NC_045931: 0.000001): 0.000004, Goniopora_lobata_MN795054: 0.007820): 0.000002, ((Porites_fontanesii_NC_037434: 0.002583, ((Porites_panamensis_NC_024182: 0.000000, Porites_sverdrupi_KU956960: 0.000000): 0.000001, Porites_porites_NC_008166: 0.000001): 0.000005): 0.000000, (Porites_harrisoni_NC_037435: 0.002548, ((Porites_lobata_NC_030186: 0.000000, Porites_okinawensis_NC_015644: 0.000000): 0.000002, (Porites_lutea_NC_029695: 0.000000, Porites_rus_NC_027526: 0.000000): 0.000000): 0.000000): 0.002558): 0.005159): 0.002759): 0.006544, Fungiacyathus_stephanus_NC_015640: 0.005313): 0.002515): 0.012254, Gardineria_hawaiiensis_NC_056290: 0.011801): 0.011839, (((((Amplexidiscus_fenestrafer_NC_027101: 0.008050, (Discosoma_sp__MH308003: 0.002636, (Rhodactis_indosinensis_NC_027103: 0.000002, Rhodactis_sp__CASIZ_171755_NC_008158: 0.005273): 0.000002): 0.002572): 0.005394, (Discosoma_nummiforme_NC_027100: 0.002613, ((Discosoma_sp__CASIZ_168915_NC_008071: 0.000002, Discosoma_sp__CASIZ_168916_NC_008072: 0.000002): 0.000002, Platyzoanthus_mussoides_NC_027104: 0.000002): 0.000002): 0.002619): 0.010633, (Pseudocorynactis_sp__SIO_KP938437: 0.005219, (Ricordea_florida_NC_008159: 0.002523, Ricordea_yuma_NC_027106: 0.002600): 0.007746): 0.002638): 0.005203, Corynactis_californica_NC_027102: 0.054968): 0.001758, Corallimorphus_profundus_KP938440: 0.016284): 0.006939): 0.004924, ((((((Astrangia_sp__JVK_2006_NC_008161: 0.004848, (((Colpophyllia_natans_NC_008162: 0.002516, Mussa_angulosa_NC_008163: 0.027541): 0.002442, ((((((Dipsastraea_favus_NC_046690: 0.000002, Dipsastraea_rotumana_NC_044074: 0.000000): 0.000001, Favites_abdita_NC_035879: 0.000001): 0.000000, Platygyra_carnosa_NC_020049: 0.000001): 0.000000, (Favites_pentagona_NC_034916: 0.000003, Hydnophora_exesa_NC_042795: 0.000005): 0.000000): 0.000000, ((Orbicella_annularis_NC_007224: 0.000000, Orbicella_faveolata_NC_007226: 0.000000): 0.000001, Orbicella_franksi_NC_007225: 0.000000): 0.024870): 0.002420, Echinophyllia_aspera_NC_040169: 0.000002): 0.014535): 0.004933, Plesiastrea_versipora_NC_042481: 0.002418): 0.000000): 0.000018, (Polycyathus_sp__MFL_2011_NC_015642: 0.002413, Psammocora_nierstraszi_MT576637: 0.000004): 0.009678): 0.032084, Madrepora_oculata_NC_018364: 0.042276): 0.009927, (Madracis_myriaster_NC_011160: 0.008114, ((Pocillopora_damicornis_NC_009797: 0.000000, Pocillopora_grandis_NC_009798: 0.000000): 0.000006, ((Seriatopora_caliendrum_NC_010245: 0.002361, Seriatopora_hystrix_NC_010244: 0.000000): 0.002368, Stylophora_pistillata_NC_011162: 0.000000): 0.000000): 0.001677): 0.014433): 0.014046, ((Desmophyllum_dianthus_NC_034275: 0.002515, Desmophyllum_pertusum_NC_015143: 0.002517): 0.000000, Solenosmilia_variabilis_NC_025472: 0.000001): 0.021345): 0.061887, Paraconotrochus_antarcticus_NC_056275: 0.089684): 0.040964): 0.029270, ((((((Antipathes_cf__dichotoma_NB_2020_MT318841: 0.000002, Stichopathes_abyssicola_MT318856: 0.000002): 0.002753, (Phanopathes_sp__NB_2020_MT318852: 0.013598, Stichopathes_sp__n__NB_2020_MT318857: 0.000002): 0.002671): 0.002685, ((Stichopathes_luetkeni_NC_018377: 0.000001, Stichopathes_sp__SCBUCN_8850_MZ157399: 0.000000): 0.000002, Stichopathes_sp__SCBUCN_8849_MZ157400: 0.000001): 0.002589): 0.000010, ((Myriopathes_japonica_NC_027667: 0.000002, Tanacetipathes_thamnea_NC_046843: 0.000002): 0.000005, Tylopathes_sp__n__NB_2020_MT318859: 0.000003): 0.005469): 0.000013, (((((((Bathypathes_sp__1_NB_2020_MT318844: 0.000000, Bathypathes_sp__n__2_NB_2020_MT318842: 0.000000): 0.000000, Stauropathes_arctica_MT318854: 0.000000): 0.000002, Bathypathes_sp__n__3_NB_2020_MT318843: 0.000002): 0.000000, Stauropathes_cf__punctata_NB_2020_MT318855: 0.000003): 0.000000, Telopathes_sp__NB_2020_MT318858: 0.000000): 0.000003, (Dendrobathypathes_sp__n__NB_2020_MT318845: 0.000003, ((Parantipathes_cf__hirondelle_NB_2020_MT318849: 0.000001, Parantipathes_hirondelle_MT318850: 0.000001): 0.000001, (Parantipathes_sp__NB_2020_MT318851: 0.000001, Sibopathes_cf__macrospina_NB_2020_MT318853: 0.000001): 0.000001): 0.000003): 0.002643): 0.013220, (Chrysopathes_formosa_NC_008411: 0.002593, Trissopathes_cf__tetracrada_NB_2020_MT318840: 0.000000): 0.000007): 0.002612): 0.005044, (Leiopathes_cf__glaberrima_NB_2020_MT318846: 0.000000, Leiopathes_expansa_MT318847: 0.000000): 0.008168): 0.016529): 0.019831, (Epizoanthus_illoricatus_NC_046474: 0.002558, (((Hydrozoanthus_gracilis_NC_046400: 0.000012, ((Neozoanthus_aff__uchina_AP_2020_MN873592: 0.000004, ((Zoanthus_cf__pulchellus_AP_2020_MN873599: 0.000002, ((Zoanthus_cf__sociatus_AP_2020_MN873600: 0.000002, Zoanthus_sociatus_NC_046476: 0.000002): 0.000002, Zoanthus_sp__AP_2020_MN873602: 0.000002): 0.000002): 0.000002, Zoanthus_sansibaricus_NC_035578: 0.000002): 0.000003): 0.002872, (Palythoa_heliodiscus_NC_035579: 0.000001, ((Palythoa_mizigama_NC_046403: 0.000002, Palythoa_mutuki_NC_046404: 0.000001): 0.002837, Sphenopus_marsupialis_NC_046406: 0.005680): 0.005734): 0.002858): 0.000001): 0.000004, ((Parazoanthus_elongatus_NC_046405: 0.002892, Parazoanthus_swiftii_NC_046475: 0.000002): 0.000003, Savalia_savaglia_DQ825686: 0.000002): 0.002863): 0.000000, (Microzoanthus_occultus_NC_046401: 0.008083, Nanozoanthus_harenaceus_NC_046402: 0.019138): 0.011318): 0.005673): 0.025473): 0.000020, (((((((Actinia_equina_NC_039929: 0.000000, Actinia_tenebrosa_NC_044902: 0.000000): 0.000001, (((Anemonia_manjano_NC_037178: 0.000001, Heteractis_aurora_NC_047219: 0.002601): 0.000002, (Anemonia_sulcata_NC_049065: 0.000000, Anemonia_viridis_NC_037177: 0.002602): 0.000003): 0.000000, Anthopleura_anjunae_NC_030274: 0.002585): 0.002598): 0.000000, Phymanthus_crucifer_NC_027614: 0.002602): 0.013418, ((((Bolocera_sp__BZ_2016_KU507297: 0.000001, (Bolocera_tuediae_NC_022470: 0.000001, Liponema_brevicorne_NC_047221: 0.000001): 0.000000): 0.002635, (Entacmaea_quadricolor_NC_049066: 0.002590, Isosicyonis_striata_KR051006: 0.005256): 0.010722): 0.000000, Stichodactyla_haddoni_MW760873: 0.000007): 0.000005, Epiactis_japonica_NC_047217: 0.008060): 0.000001): 0.002662, ((((Alicia_sansibarensis_NC_027610: 0.016210, (Haloclava_producta_NC_047218: 0.002384, Sagartia_ornata_NC_027615: 0.010533): 0.015789): 0.000002, (Diadumene_lineata_NC_045515: 0.012792, Metridium_senile_NC_000933: 0.002629): 0.005189): 0.000016, Paraphelliactis_xishaensis_MT997141: 0.018628): 0.000005, Antholoba_achates_NC_027611: 0.021122): 0.000000): 0.002718, Halcampoides_purpureus_NC_027612: 0.000010): 0.004546, Nematostella_sp__JVK_2006_NC_008164: 0.033657): 0.004629): 0.017486, (((((((Aurelia_aurita_NC_008446: 0.016788, (Aurelia_coerulea_NC_046792: 0.013799, Aurelia_limbata_NC_046691: 0.011687): 0.007548): 0.008370, (Aurelia_sp__3_sensu_Dawson_et_al___2005__LC005413: 0.000075, Aurelia_sp__4_sensu_Dawson_et_al___2005__LC005414: 0.023313): 0.000027): 0.022974, ((Nemopilema_nomurai_NC_035740: 0.010176, Rhopilema_esculentum_NC_035741: 0.019394): 0.028304, Stomolophus_sp__CG_2019_MK157198: 0.017717): 0.019837): 0.006131, Cassiopea_xamachana_NC_016466: 0.087095): 0.036817, (Chrysaora_pacifica_NC_046775: 0.000000, Chrysaora_quinquecirrha_NC_020459: 0.002354): 0.046167): 0.103490, ((((((Blackfordia_virginica_NC_053946: 0.023169, Eutima_sp__BMK_2020_MW066348: 0.022951): 0.009502, Laomedea_flexuosa_JN700945: 0.011333): 0.031948, (Clava_multicornis_NC_016465: 0.037700, (Turritopsis_dohrnii_NC_031213: 0.014392, Turritopsis_lata_NC_056128: 0.002418): 0.030403): 0.009225): 0.009798, Spirocodon_saltatrix_NC_049868: 0.032278): 0.020528, ((Hydra_oligactis_NC_010214: 0.019872, Hydra_sinensis_NC_021406: 0.107094): 0.144642, Nemalecium_lighti_MZ457217: 0.085075): 0.044826): 0.046620, (Craspedacusta_sowerbii_NC_018537: 0.007089, Cubaia_aphrodite_NC_016467: 0.049823): 0.043248): 0.045707): 0.016256, (Haliclystus_antarcticus_NC_030337: 0.000000, OU744455_1_OU744455_1__OU744455_1: 0.010421): 0.122807): 0.045988): 0.077347, Carijoa_riisei_NC_048963: 0.005033): 0.000012, (Sarcophyton_trocheliophorum_MK994517: 0.000014, (((Sinularia_acuta_MW987591: 0.000000, (Sinularia_maxima_MN485891: 0.000000, Sinularia_penghuensis_MW256412: 0.000000): 0.000000): 0.000000, Sinularia_cf__cruciata_GS_2016_NC_034318: 0.014375): 0.002604, (Sinularia_ceramensis_NC_044122: 0.000000, Sinularia_peculiaris_NC_018379: 0.000000): 0.000004): 0.005220): 0.002630): 0.000000, (((((((Antillogorgia_bipinnata_NC_008157: 0.000004, ((((Eugorgia_mutabilis_NC_035665: 0.000001, Leptogorgia_alba_NC_035669: 0.000001): 0.000000, (Leptogorgia_hebes_MN052675: 0.002564, Leptogorgia_virgulata_MK301586: 0.000000): 0.000000): 0.000001, Leptogorgia_sp__USNM_1437444_KY559412: 0.000001): 0.000001, Pacifigorgia_cairnsi_NC_035668: 0.000013): 0.000003): 0.000000, (((Leptogorgia_capverdensis_NC_035663: 0.000000, Leptogorgia_sarmentosa_NC_035670: 0.000000): 0.000000, Leptogorgia_gaini_KY559404: 0.000001): 0.000005, Leptogorgia_cf__palma_AP_2017_KY559406: 0.000001): 0.000000): 0.000000, (Eunicella_albicans_NC_035666: 0.007870, (Eunicella_cavolini_NC_035667: 0.000000, Eunicella_verrucosa_MW588805: 0.000000): 0.002601): 0.002638): 0.000002, (Calicogorgia_granulosa_NC_023345: 0.032619, Euplexaura_crassa_NC_020458: 0.002567): 0.002676): 0.005212, Muricea_crassa_NC_029697: 0.007751): 0.000000, ((((((Dendronephthya_castanea_NC_023343: 0.000000, Dendronephthya_mollis_NC_020456: 0.000000): 0.000000, Dendronephthya_gigantea_NC_013573: 0.000000): 0.000001, (Dendronephthya_putteri_NC_036022: 0.000001, Dendronephthya_suensoni_NC_022809: 0.000000): 0.000000): 0.000004, Scleronephthya_gracillimum_NC_023344: 0.000002): 0.000013, (Echinogorgia_complexa_NC_020457: 0.000003, Paramuricea_clavata_NC_034749: 0.002600): 0.002604): 0.000000, Trachythela_sp__YZ_2021_MW238423: 0.005236): 0.000002): 0.003729, (Incrustatus_comauensis_MT254531: 0.009898, Telestula_humilis_MT254527: 0.005883): 0.004613): 0.000000): 0.025566, Briareum_asbestinum_NC_008073: 0.008415): 0.000000, (Telestula_cf__batoni_AP_2020_MT254530: 0.000002, Telestula_septentrionalis_MT254532: 0.010230): 0.005844): 0.001788, (((Anthomastus_sp__USNM_1081145_KM015353: 0.000003, Anthomastus_sp__USNM_1171062_KM015352: 0.000001): 0.000005, (((Corallium_japonicum_AB595189: 0.000001, Corallium_rubrum_NC_022864: 0.005318): 0.000007, (Pleurocorallium_elatius_NC_022804: 0.000000, Pleurocorallium_konojoi_NC_015406: 0.000002): 0.000004): 0.000000, Sibogagorgia_cauliflora_NC_026193: 0.002574): 0.002572): 0.000004, Paraminabea_aldersladei_NC_018790: 0.007650): 0.010273): 0.007706, (((((((Anthoptilum_grandiflorum_NC_044086: 0.007599, Anthoptilum_sp__1_RH_2019_MK919656: 0.002512): 0.002508, (Umbellula_huxleyi_MK919668: 0.000000, Umbellula_sp__1_RH_2019_MK919669: 0.000002): 0.010161): 0.002507, Virgularia_mirabilis_NC_044091: 0.007536): 0.000006, (Funiculina_quadrangularis_NC_044078: 0.002525, ((Kophobelemnon_sp__1_RH_2019_MK919660: 0.000000, Kophobelemnon_sp__3_RH_2019_MK919661: 0.000000): 0.000002, Kophobelemnon_sp__4_RH_2019_MK919662: 0.000002): 0.000005): 0.002523): 0.000000, (Halipteris_cf__finmarchica_RH_2019_MK919659: 0.002571, Umbellula_sp__2_RH_2019_MK919670: 0.002558): 0.005115): 0.000002, ((((Distichoptilum_gracile_NC_044077: 0.005166, (Pennatula_cf__inflata_RH_2019_MK919666: 0.000002, Pennatula_grandis_NC_044088: 0.000003): 0.002536): 0.000000, ((Pennatula_aculeata_NC_044087: 0.000001, Pennatula_cf__aculeata_RH_2019_MK919664: 0.000000): 0.010123, Renilla_muelleri_NC_018378: 0.007858): 0.005236): 0.000003, Protoptilum_carpenteri_NC_044089: 0.000004): 0.000000, Stylatula_elongata_NC_018380: 0.000004): 0.007604): 0.010317, Junceella_fragilis_NC_024181: 0.010300): 0.000005): 0.000002, Heliopora_coerulea_NC_020375: 0.005128): 0.005208, ((Callogorgia_cf__gracilis_EE_2018_MH719202: 0.005194, (Plumarella_adhaerans_NC_046480: 0.000000, Plumarella_spinosa_NC_046465: 0.000000): 0.000004): 0.005198, Narella_hawaiiensis_NC_026192: 0.002567): 0.002605): 0.002601, Keratoisidinae_sp__BAL208_1_NC_010764: 0.002615);

**w ratios as labels for TreeView:**

(Acanella_arbuscula_NC_011016 #0.0001 , ((((((((((((((((((((((((((((((Acropora_aculeus_KT001202 #1.16928 , (Acropora_horrida_NC_022825 #0.0001 , LC201842_1_LC201842_1__LC201842_1 #1.59532 ) #2.38305 ) #1.55312 , LC201815_1_LC201815_1__LC201815_1 #1.10534 ) #1.34668 , ((((Acropora_aspera_NC_022827 #2.39808 , Acropora_muricata_NC_022824 #1.64917 ) #1.61749 , LC201828_1_LC201828_1__LC201828_1 #1.55572 ) #1.59791 , (((Acropora_digitifera_NC_022830 #1.36322 , LC201849_1_LC201849_1__LC201849_1 #1.38294 ) #1.40608 , Acropora_florida_NC_022828 #1.46154 ) #1.67151 , Acropora_humilis_NC_022823 #1.37932 ) #1.37954 ) #1.38054 , (Acropora_hyacinthus_NC_022826 #1.5633 , LC201817_1_LC201817_1__LC201817_1 #1.62051 ) #1.59988 ) #1.43627 ) #1.37946 , (Acropora_divaricata_NC_022832 #1.58152 , (((Acropora_nasuta_NC_022831 #1.3443 , Acropora_valida_NC_042742 #2.15811 ) #1.46708 , LC201847_1_LC201847_1__LC201847_1 #1.36151 ) #1.40031 , LC201820_1_LC201820_1__LC201820_1 #2.15351 ) #0.0001 ) #1.64636 ) #1.18652 , Acropora_robusta_NC_022833 #1.53969 ) #1.05365 , LC201813_1_LC201813_1__LC201813_1 #0.0001 ) #1.71682 , ((Acropora_tenuis_NC_003522 #1.69681 , Acropora_yongei_NC_022829 #1.52536 ) #1.69794 , LC201816_1_LC201816_1__LC201816_1 #1.10318 ) #1.6889 ) #0.0001 , (Isopora_palifera_NC_024091 #0.620533 , Isopora_togianensis_NC_024089 #1.08664 ) #0.0001 ) #0.0624539 , ((Anacropora_matthai_NC_006898 #2.45016 , Montipora_efflorescens_NC_040137 #2.73207 ) #0.0001 , (Montipora_aequituberculata_NC_037359 #0.0001 , Montipora_cactus_NC_006902 #3.52811 ) #1.22278 ) #0.466439 ) #0.0001 , (Alveopora_japonica_NC_040136 #0.0001 , Alveopora_sp__MFL_2014_KJ634271 #0.0001 ) #0.0360262 ) #0.0001 , (Astreopora_explanata_NC_024090 #0.0001 , Astreopora_myriophthalma_NC_024092 #0.0001 ) #0.0432327 ) #0.0001 , ((Agaricia_humilis_NC_008160 #0.0001 , Pavona_decussata_NC_026527 #0.122425 ) #0.0908443 , (Fimbriaphyllia_ancora_NC_015641 #0.0433632 , Galaxea_fascicularis_NC_029696 #0.0001 ) #0.0233293 ) #0.0001 ) #0.0001 , ((Pseudosiderastrea_formosa_NC_026530 #0.0001 , Pseudosiderastrea_tayamai_NC_026531 #99.4881 ) #0.0857096 , Siderastrea_radians_NC_008167 #0.0173631 ) #0.0536217 ) #0.0001 , ((((((Dendrophyllia_arbuscula_NC_027590 #0.0001 , Tubastraea_coccinea_NC_026025 #0.219117 ) #0.31426 , ((Tubastraea_diaphana_MK959042 #1.50373 , Tubastraea_tagusensis_NC_030352 #1.29739 ) #1.55757 , Tubastraea_micranthus_MK959041 #1.53381 ) #0.0001 ) #0.310757 , Dendrophyllia_cribrosa_NC_026026 #0.0001 ) #0.0363732 , Turbinaria_peltata_NC_024671 #0.14046 ) #0.261686 , (((Goniopora_columna_NC_015643 #0.662421 , Goniopora_djiboutiensis_NC_045931 #1.55821 ) #0.0001 , Goniopora_lobata_MN795054 #0.449506 ) #0.0001 , ((Porites_fontanesii_NC_037434 #0.0639122 , ((Porites_panamensis_NC_024182 #0.0001 , Porites_sverdrupi_KU956960 #0.0001 ) #0.0001 , Porites_porites_NC_008166 #0.0001 ) #0.0001 ) #0.0001 , (Porites_harrisoni_NC_037435 #116.828 , ((Porites_lobata_NC_030186 #0.0001 , Porites_okinawensis_NC_015644 #0.0001 ) #0.0001 , (Porites_lutea_NC_029695 #0.0001 , Porites_rus_NC_027526 #0.0001 ) #0.0001 ) #0.0001 ) #0.144231 ) #0.0449435 ) #0.0280669 ) #0.433233 , Fungiacyathus_stephanus_NC_015640 #0.0536117 ) #0.0671864 ) #0.145532 , Gardineria_hawaiiensis_NC_056290 #0.0736466 ) #0.048131 , (((((Amplexidiscus_fenestrafer_NC_027101 #0.986918 , (Discosoma_sp__MH308003 #118.862 , (Rhodactis_indosinensis_NC_027103 #1.94902 , Rhodactis_sp__CASIZ_171755_NC_008158 #149.412 ) #2.42455 ) #0.318237 ) #0.324437 , (Discosoma_nummiforme_NC_027100 #118.286 , ((Discosoma_sp__CASIZ_168915_NC_008071 #2.58671 , Discosoma_sp__CASIZ_168916_NC_008072 #2.35113 ) #2.06419 , Platyzoanthus_mussoides_NC_027104 #2.09361 ) #1.94909 ) #91.6152 ) #0.0839342 , (Pseudocorynactis_sp__SIO_KP938437 #0.0320677 , (Ricordea_florida_NC_008159 #109.077 , Ricordea_yuma_NC_027106 #0.151009 ) #0.0725215 ) #0.13005 ) #0.218832 , Corynactis_californica_NC_027102 #0.44895 ) #0.0225954 , Corallimorphus_profundus_KP938440 #0.198353 ) #0.0782694 ) #0.0177989 , ((((((Astrangia_sp__JVK_2006_NC_008161 #0.0328945 , (((Colpophyllia_natans_NC_008162 #0.119575 , Mussa_angulosa_NC_008163 #1.60138 ) #0.033551 , ((((((Dipsastraea_favus_NC_046690 #0.0001 , Dipsastraea_rotumana_NC_044074 #0.0001 ) #0.0001 , Favites_abdita_NC_035879 #0.0001 ) #0.0001 , Platygyra_carnosa_NC_020049 #0.0001 ) #0.0001 , (Favites_pentagona_NC_034916 #0.0001 , Hydnophora_exesa_NC_042795 #0.0001 ) #0.0001 ) #0.0001 , ((Orbicella_annularis_NC_007224 #0.0001 , Orbicella_faveolata_NC_007226 #0.0001 ) #0.0001 , Orbicella_franksi_NC_007225 #0.0001 ) #0.183405 ) #103.509 , Echinophyllia_aspera_NC_040169 #0.0001 ) #0.100515 ) #97.6528 , Plesiastrea_versipora_NC_042481 #0.00587878 ) #0.0001 ) #0.0001 , (Polycyathus_sp__MFL_2011_NC_015642 #0.088161 , Psammocora_nierstraszi_MT576637 #0.0001 ) #101 ) #0.0410504 , Madrepora_oculata_NC_018364 #0.0445172 ) #79.8703 , (Madracis_myriaster_NC_011160 #0.156525 , ((Pocillopora_damicornis_NC_009797 #0.0001 , Pocillopora_grandis_NC_009798 #0.0001 ) #0.0001 , ((Seriatopora_caliendrum_NC_010245 #0.249989 , Seriatopora_hystrix_NC_010244 #0.0001 ) #0.24955 , Stylophora_pistillata_NC_011162 #0.0001 ) #0.0001 ) #0.0110805 ) #0.021844 ) #0.10093 , ((Desmophyllum_dianthus_NC_034275 #115.432 , Desmophyllum_pertusum_NC_015143 #115.423 ) #0.0001 , Solenosmilia_variabilis_NC_025472 #0.0001 ) #0.0513807 ) #0.000894912 , Paraconotrochus_antarcticus_NC_056275 #0.00129852 ) #0.00668404 ) #0.0985113 , ((((((Antipathes_cf__dichotoma_NB_2020_MT318841 #4.75144 , Stichopathes_abyssicola_MT318856 #4.73121 ) #0.0117568 , (Phanopathes_sp__NB_2020_MT318852 #0.548396 , Stichopathes_sp__n__NB_2020_MT318857 #0.0001 ) #0.022159 ) #0.0288665 , ((Stichopathes_luetkeni_NC_018377 #0.0001 , Stichopathes_sp__SCBUCN_8850_MZ157399 #0.0001 ) #0.0001 , Stichopathes_sp__SCBUCN_8849_MZ157400 #0.0001 ) #0.00608953 ) #0.0001 , ((Myriopathes_japonica_NC_027667 #3.8362 , Tanacetipathes_thamnea_NC_046843 #3.83771 ) #0.0001 , Tylopathes_sp__n__NB_2020_MT318859 #0.0001 ) #0.0330976 ) #0.0001 , (((((((Bathypathes_sp__1_NB_2020_MT318844 #0.0001 , Bathypathes_sp__n__2_NB_2020_MT318842 #0.0001 ) #0.0001 , Stauropathes_arctica_MT318854 #0.0001 ) #0.0001 , Bathypathes_sp__n__3_NB_2020_MT318843 #0.0001 ) #0.0001 , Stauropathes_cf__punctata_NB_2020_MT318855 #0.0001 ) #0.0001 , Telopathes_sp__NB_2020_MT318858 #0.0001 ) #0.0001 , (Dendrobathypathes_sp__n__NB_2020_MT318845 #0.0001 , ((Parantipathes_cf__hirondelle_NB_2020_MT318849 #1.16119 , Parantipathes_hirondelle_MT318850 #1.28186 ) #1.35611 , (Parantipathes_sp__NB_2020_MT318851 #1.06448 , Sibopathes_cf__macrospina_NB_2020_MT318853 #1.18214 ) #1.15247 ) #0.0001 ) #0.14312 ) #0.106219 , (Chrysopathes_formosa_NC_008411 #0.0999901 , Trissopathes_cf__tetracrada_NB_2020_MT318840 #0.0001 ) #0.0001 ) #97.5945 ) #0.0353924 , (Leiopathes_cf__glaberrima_NB_2020_MT318846 #0.0001 , Leiopathes_expansa_MT318847 #0.0001 ) #0.0939094 ) #0.0854204 ) #0.188144 , (Epizoanthus_illoricatus_NC_046474 #0.0361453 , (((Hydrozoanthus_gracilis_NC_046400 #0.0001 , ((Neozoanthus_aff__uchina_AP_2020_MN873592 #0.0001 , ((Zoanthus_cf__pulchellus_AP_2020_MN873599 #5.46073 , ((Zoanthus_cf__sociatus_AP_2020_MN873600 #5.34462 , Zoanthus_sociatus_NC_046476 #5.45709 ) #5.56323 , Zoanthus_sp__AP_2020_MN873602 #5.45878 ) #5.34745 ) #5.42605 , Zoanthus_sansibaricus_NC_035578 #5.3279 ) #0.0001 ) #0.153062 , (Palythoa_heliodiscus_NC_035579 #0.0001 , ((Palythoa_mizigama_NC_046403 #5.41805 , Palythoa_mutuki_NC_046404 #0.0001 ) #0.366788 , Sphenopus_marsupialis_NC_046406 #0.726848 ) #0.685489 ) #0.0445697 ) #0.0001 ) #0.0001 , ((Parazoanthus_elongatus_NC_046405 #0.392217 , Parazoanthus_swiftii_NC_046475 #0.0001 ) #0.0001 , Savalia_savaglia_DQ825686 #0.0001 ) #0.144548 ) #0.0001 , (Microzoanthus_occultus_NC_046401 #0.00332211 , Nanozoanthus_harenaceus_NC_046402 #0.0115166 ) #0.0171121 ) #0.0250804 ) #0.0392567 ) #0.0001 , (((((((Actinia_equina_NC_039929 #0.0001 , Actinia_tenebrosa_NC_044902 #0.0001 ) #0.0001 , (((Anemonia_manjano_NC_037178 #0.0001 , Heteractis_aurora_NC_047219 #0.311091 ) #0.0001 , (Anemonia_sulcata_NC_049065 #0.0001 , Anemonia_viridis_NC_037177 #116.888 ) #0.0001 ) #0.0001 , Anthopleura_anjunae_NC_030274 #0.1005 ) #115.667 ) #0.0001 , Phymanthus_crucifer_NC_027614 #0.0220364 ) #0.0469651 , ((((Bolocera_sp__BZ_2016_KU507297 #0.564639 , (Bolocera_tuediae_NC_022470 #0.504853 , Liponema_brevicorne_NC_047221 #0.709874 ) #0.00010069 ) #0.215623 , (Entacmaea_quadricolor_NC_049066 #0.0742136 , Isosicyonis_striata_KR051006 #0.146593 ) #0.0341397 ) #0.0001 , Stichodactyla_haddoni_MW760873 #0.0001 ) #0.0001 , Epiactis_japonica_NC_047217 #0.0571817 ) #0.0001 ) #84.4069 , ((((Alicia_sansibarensis_NC_027610 #0.0763276 , (Haloclava_producta_NC_047218 #0.0456373 , Sagartia_ornata_NC_027615 #0.361336 ) #0.429025 ) #0.0001 , (Diadumene_lineata_NC_045515 #0.0321826 , Metridium_senile_NC_000933 #0.0416906 ) #0.0480119 ) #0.0001 , Paraphelliactis_xishaensis_MT997141 #0.113052 ) #0.0001 , Antholoba_achates_NC_027611 #0.0340759 ) #0.0001 ) #0.0138823 , Halcampoides_purpureus_NC_027612 #0.0001 ) #0.0141531 , Nematostella_sp__JVK_2006_NC_008164 #0.0241046 ) #0.026832 ) #0.0100443 , (((((((Aurelia_aurita_NC_008446 #0.0249489 , (Aurelia_coerulea_NC_046792 #0.0306854 , Aurelia_limbata_NC_046691 #0.0250968 ) #0.111985 ) #26.0991 , (Aurelia_sp__3_sensu_Dawson_et_al___2005__LC005413 #0.0001 , Aurelia_sp__4_sensu_Dawson_et_al___2005__LC005414 #0.042597 ) #0.0001 ) #0.0164131 , ((Nemopilema_nomurai_NC_035740 #0.0168296 , Rhopilema_esculentum_NC_035741 #0.0216208 ) #72.6978 , Stomolophus_sp__CG_2019_MK157198 #0.0105309 ) #0.077983 ) #69.6587 , Cassiopea_xamachana_NC_016466 #0.0255977 ) #116.613 , (Chrysaora_pacifica_NC_046775 #0.0001 , Chrysaora_quinquecirrha_NC_020459 #0.0292994 ) #0.0179206 ) #162.073 , ((((((Blackfordia_virginica_NC_053946 #0.0187906 , Eutima_sp__BMK_2020_MW066348 #0.0207101 ) #0.0519683 , Laomedea_flexuosa_JN700945 #0.00591398 ) #73.2648 , (Clava_multicornis_NC_016465 #0.0368684 , (Turritopsis_dohrnii_NC_031213 #0.0255371 , Turritopsis_lata_NC_056128 #0.00305611 ) #108.51 ) #0.0367681 ) #0.0198438 , Spirocodon_saltatrix_NC_049868 #0.0199992 ) #0.0255366 , ((Hydra_oligactis_NC_010214 #0.0958314 , Hydra_sinensis_NC_021406 #0.0636014 ) #0.0531138 , Nemalecium_lighti_MZ457217 #0.0061934 ) #55.929 ) #117.696 , (Craspedacusta_sowerbii_NC_018537 #0.00598378 , Cubaia_aphrodite_NC_016467 #0.000720072 ) #0.0149262 ) #112.603 ) #58.6055 , (Haliclystus_antarcticus_NC_030337 #0.0001 , OU744455_1_OU744455_1__OU744455_1 #0.00440634 ) #0.0017808 ) #50.5218 ) #0.0300905 , Carijoa_riisei_NC_048963 #70.927 ) #0.0001 , (Sarcophyton_trocheliophorum_MK994517 #0.0001 , (((Sinularia_acuta_MW987591 #0.0001 , (Sinularia_maxima_MN485891 #0.0001 , Sinularia_penghuensis_MW256412 #0.0001 ) #0.0001 ) #0.0001 , Sinularia_cf__cruciata_GS_2016_NC_034318 #0.202405 ) #0.273266 , (Sinularia_ceramensis_NC_044122 #0.0001 , Sinularia_peculiaris_NC_018379 #0.0001 ) #0.0001 ) #0.184939 ) #0.0955358 ) #0.0001 , (((((((Antillogorgia_bipinnata_NC_008157 #0.0001 , ((((Eugorgia_mutabilis_NC_035665 #0.0001 , Leptogorgia_alba_NC_035669 #0.0001 ) #0.0001 , (Leptogorgia_hebes_MN052675 #0.0916173 , Leptogorgia_virgulata_MK301586 #0.0001 ) #0.0001 ) #0.0001 , Leptogorgia_sp__USNM_1437444_KY559412 #0.0001 ) #0.0001 , Pacifigorgia_cairnsi_NC_035668 #0.0001 ) #0.0001 ) #0.0001 , (((Leptogorgia_capverdensis_NC_035663 #0.0001 , Leptogorgia_sarmentosa_NC_035670 #0.0001 ) #0.0001 , Leptogorgia_gaini_KY559404 #0.0001 ) #0.0001 , Leptogorgia_cf__palma_AP_2017_KY559406 #0.0001 ) #0.0001 ) #0.0001 , (Eunicella_albicans_NC_035666 #1.57469 , (Eunicella_cavolini_NC_035667 #0.0001 , Eunicella_verrucosa_MW588805 #0.0001 ) #0.108762 ) #0.0595505 ) #0.0001 , (Calicogorgia_granulosa_NC_023345 #0.0719434 , Euplexaura_crassa_NC_020458 #0.0705844 ) #96.6685 ) #142.779 , Muricea_crassa_NC_029697 #0.116112 ) #0.0001 , ((((((Dendronephthya_castanea_NC_023343 #0.0001 , Dendronephthya_mollis_NC_020456 #0.0001 ) #0.0001 , Dendronephthya_gigantea_NC_013573 #0.0001 ) #0.0001 , (Dendronephthya_putteri_NC_036022 #0.0001 , Dendronephthya_suensoni_NC_022809 #0.0001 ) #0.0001 ) #0.0001 , Scleronephthya_gracillimum_NC_023344 #0.0001 ) #0.0001 , (Echinogorgia_complexa_NC_020457 #0.0001 , Paramuricea_clavata_NC_034749 #0.0545977 ) #0.0543682 ) #0.0001 , Trachythela_sp__YZ_2021_MW238423 #0.0500454 ) #5.21594 ) #0.42273 , (Incrustatus_comauensis_MT254531 #0.260849 , Telestula_humilis_MT254527 #0.264126 ) #0.139113 ) #0.0001 ) #0.167717 , Briareum_asbestinum_NC_008073 #0.0454973 ) #0.0001 , (Telestula_cf__batoni_AP_2020_MT254530 #0.0001 , Telestula_septentrionalis_MT254532 #1.12212 ) #0.187877 ) #0.19736 , (((Anthomastus_sp__USNM_1081145_KM015353 #0.0001 , Anthomastus_sp__USNM_1171062_KM015352 #0.0001 ) #0.0001 , (((Corallium_japonicum_AB595189 #0.0001 , Corallium_rubrum_NC_022864 #0.597401 ) #0.0001 , (Pleurocorallium_elatius_NC_022804 #0.0001 , Pleurocorallium_konojoi_NC_015406 #0.0001 ) #0.0001 ) #0.0001 , Sibogagorgia_cauliflora_NC_026193 #0.289539 ) #0.0912024 ) #0.0001 , Paraminabea_aldersladei_NC_018790 #0.233605 ) #0.226468 ) #0.244261 , (((((((Anthoptilum_grandiflorum_NC_044086 #0.16069 , Anthoptilum_sp__1_RH_2019_MK919656 #110.927 ) #0.0531285 , (Umbellula_huxleyi_MK919668 #0.0001 , Umbellula_sp__1_RH_2019_MK919669 #0.0001 ) #1.10866 ) #109.161 , Virgularia_mirabilis_NC_044091 #0.13194 ) #0.0001 , (Funiculina_quadrangularis_NC_044078 #0.242265 , ((Kophobelemnon_sp__1_RH_2019_MK919660 #0.0001 , Kophobelemnon_sp__3_RH_2019_MK919661 #0.0001 ) #0.0001 , Kophobelemnon_sp__4_RH_2019_MK919662 #0.0001 ) #0.0001 ) #111.891 ) #0.0001 , (Halipteris_cf__finmarchica_RH_2019_MK919659 #0.039666 , Umbellula_sp__2_RH_2019_MK919670 #0.0448968 ) #0.564358 ) #6.76562 , ((((Distichoptilum_gracile_NC_044077 #0.181107 , (Pennatula_cf__inflata_RH_2019_MK919666 #0.0001 , Pennatula_grandis_NC_044088 #0.0001 ) #0.0998244 ) #0.0001 , ((Pennatula_aculeata_NC_044087 #0.0001 , Pennatula_cf__aculeata_RH_2019_MK919664 #0.0001 ) #0.571915 , Renilla_muelleri_NC_018378 #0.0783562 ) #0.155367 ) #0.0001 , Protoptilum_carpenteri_NC_044089 #0.0001 ) #0.0001 , Stylatula_elongata_NC_018380 #0.0001 ) #161.984 ) #0.23662 , Junceella_fragilis_NC_024181 #0.0922215 ) #0.0001 ) #0.0001 , Heliopora_coerulea_NC_020375 #0.0252369 ) #0.177601 , ((Callogorgia_cf__gracilis_EE_2018_MH719202 #0.0935064 , (Plumarella_adhaerans_NC_046480 #0.0001 , Plumarella_spinosa_NC_046465 #0.0001 ) #0.0001 ) #0.537846 , Narella_hawaiiensis_NC_026192 #69.9853 ) #0.126977 ) #0.0422465 , Keratoisidinae_sp__BAL208_1_NC_010764 #0.126913 );

COX3:

**dS tree:**

(Acanella_arbuscula_NC_011016: 0.050672, ((((((((((((((((((((((((((((((Acropora_aculeus_KT001202: 0.000006, (Acropora_horrida_NC_022825: 0.000006, LC201842_1_LC201842_1__LC201842_1: 0.000006): 0.000006): 0.000006, LC201815_1_LC201815_1__LC201815_1: 0.000006): 0.000006, ((((Acropora_aspera_NC_022827: 0.000006, Acropora_muricata_NC_022824: 0.000006): 0.000006, LC201828_1_LC201828_1__LC201828_1: 0.000006): 0.000006, (((Acropora_digitifera_NC_022830: 0.000006, LC201849_1_LC201849_1__LC201849_1: 0.000006): 0.000006, Acropora_florida_NC_022828: 0.000006): 0.000006, Acropora_humilis_NC_022823: 0.000006): 0.000006): 0.000006, (Acropora_hyacinthus_NC_022826: 0.000006, LC201817_1_LC201817_1__LC201817_1: 0.000006): 0.000006): 0.000006): 0.000006, (Acropora_divaricata_NC_022832: 0.000006, (((Acropora_nasuta_NC_022831: 0.000006, Acropora_valida_NC_042742: 0.000006): 0.000006, LC201847_1_LC201847_1__LC201847_1: 0.000006): 0.000006, LC201820_1_LC201820_1__LC201820_1: 0.000006): 0.000006): 0.000006): 0.000006, Acropora_robusta_NC_022833: 0.000002): 0.000006, LC201813_1_LC201813_1__LC201813_1: 0.015945): 0.000006, ((Acropora_tenuis_NC_003522: 0.007898, Acropora_yongei_NC_022829: 0.000006): 0.000006, LC201816_1_LC201816_1__LC201816_1: 0.000006): 0.000006): 0.000006, (Isopora_palifera_NC_024091: 0.000002, Isopora_togianensis_NC_024089: 0.000006): 0.015819): 0.000013, ((Anacropora_matthai_NC_006898: 0.000006, Montipora_efflorescens_NC_040137: 0.000006): 0.008053, (Montipora_aequituberculata_NC_037359: 0.000006, Montipora_cactus_NC_006902: 0.008064): 0.000006): 0.198838): 0.000006, (Alveopora_japonica_NC_040136: 0.000006, Alveopora_sp__MFL_2014_KJ634271: 0.000006): 0.213826): 0.008123, (Astreopora_explanata_NC_024090: 0.000002, Astreopora_myriophthalma_NC_024092: 0.000002): 0.000027): 0.241901, ((Agaricia_humilis_NC_008160: 0.002570, Pavona_decussata_NC_026527: 0.044072): 0.178831, (Fimbriaphyllia_ancora_NC_015641: 0.083211, Galaxea_fascicularis_NC_029696: 0.033500): 0.123130): 0.064286): 0.032392, ((Pseudosiderastrea_formosa_NC_026530: 0.008308, Pseudosiderastrea_tayamai_NC_026531: 0.007700): 0.045761, Siderastrea_radians_NC_008167: 0.052258): 0.031148): 0.013392, ((((((Dendrophyllia_arbuscula_NC_027590: 0.007757, Tubastraea_coccinea_NC_026025: 0.007808): 0.000006, ((Tubastraea_diaphana_MK959042: 0.000006, Tubastraea_tagusensis_NC_030352: 0.000006): 0.000006, Tubastraea_micranthus_MK959041: 0.000006): 0.000002): 0.007752, Dendrophyllia_cribrosa_NC_026026: 0.015923): 0.056408, Turbinaria_peltata_NC_024671: 0.036285): 0.034403, (((Goniopora_columna_NC_015643: 0.016567, Goniopora_djiboutiensis_NC_045931: 0.000006): 0.000002, Goniopora_lobata_MN795054: 0.008113): 0.060008, ((Porites_fontanesii_NC_037434: 0.050352, ((Porites_panamensis_NC_024182: 0.000006, Porites_sverdrupi_KU956960: 0.000006): 0.000006, Porites_porites_NC_008166: 0.000006): 0.000006): 0.000006, (Porites_harrisoni_NC_037435: 0.008130, ((Porites_lobata_NC_030186: 0.000006, Porites_okinawensis_NC_015644: 0.000006): 0.016416, (Porites_lutea_NC_029695: 0.000006, Porites_rus_NC_027526: 0.000002): 0.000006): 0.000006): 0.024823): 0.071238): 0.027154): 0.009334, Fungiacyathus_stephanus_NC_015640: 0.096416): 0.021520): 0.095509, Gardineria_hawaiiensis_NC_056290: 0.081583): 0.176163, (((((Amplexidiscus_fenestrafer_NC_027101: 0.007776, (Discosoma_sp__MH308003: 0.000004, (Rhodactis_indosinensis_NC_027103: 0.007937, Rhodactis_sp__CASIZ_171755_NC_008158: 0.000002): 0.007916): 0.015933): 0.023492, (Discosoma_nummiforme_NC_027100: 0.000009, ((Discosoma_sp__CASIZ_168915_NC_008071: 0.000006, Discosoma_sp__CASIZ_168916_NC_008072: 0.000006): 0.000006, Platyzoanthus_mussoides_NC_027104: 0.000002): 0.031568): 0.025075): 0.042469, (Pseudocorynactis_sp__SIO_KP938437: 0.159888, (Ricordea_florida_NC_008159: 0.042881, Ricordea_yuma_NC_027106: 0.026145): 0.066142): 0.067529): 0.100546, Corynactis_californica_NC_027102: 0.114654): 0.063655, Corallimorphus_profundus_KP938440: 0.099837): 0.148306): 0.170512, ((((((Astrangia_sp__JVK_2006_NC_008161: 0.039524, (((Colpophyllia_natans_NC_008162: 0.008407, Mussa_angulosa_NC_008163: 0.049681): 0.031833, ((((((Dipsastraea_favus_NC_046690: 0.000002, Dipsastraea_rotumana_NC_044074: 0.007938): 0.025796, Favites_abdita_NC_035879: 0.053084): 0.039249, Platygyra_carnosa_NC_020049: 0.124256): 0.000006, (Favites_pentagona_NC_034916: 0.033235, Hydnophora_exesa_NC_042795: 0.086042): 0.014013): 0.000006, ((Orbicella_annularis_NC_007224: 0.000006, Orbicella_faveolata_NC_007226: 0.000006): 0.000006, Orbicella_franksi_NC_007225: 0.015788): 0.042164): 0.016864, Echinophyllia_aspera_NC_040169: 0.141865): 0.004575): 0.187821, Plesiastrea_versipora_NC_042481: 0.010774): 0.218234): 0.127454, (Polycyathus_sp__MFL_2011_NC_015642: 0.000006, Psammocora_nierstraszi_MT576637: 0.017635): 0.574562): 0.342267, Madrepora_oculata_NC_018364: 1.167640): 0.613289, (Madracis_myriaster_NC_011160: 0.000006, ((Pocillopora_damicornis_NC_009797: 0.000006, Pocillopora_grandis_NC_009798: 0.000006): 0.009398, ((Seriatopora_caliendrum_NC_010245: 0.017025, Seriatopora_hystrix_NC_010244: 0.008626): 0.018233, Stylophora_pistillata_NC_011162: 0.015869): 0.000006): 0.149762): 0.384454): 0.000039, ((Desmophyllum_dianthus_NC_034275: 0.007802, Desmophyllum_pertusum_NC_015143: 0.007841): 0.000006, Solenosmilia_variabilis_NC_025472: 0.000006): 1.870004): 0.828883, Paraconotrochus_antarcticus_NC_056275: 1.671975): 0.285434): 0.511505, ((((((Antipathes_cf__dichotoma_NB_2020_MT318841: 0.000000, Stichopathes_abyssicola_MT318856: 0.000000): 0.224394, (Phanopathes_sp__NB_2020_MT318852: 0.164732, Stichopathes_sp__n__NB_2020_MT318857: 0.172491): 0.000006): 0.093056, ((Stichopathes_luetkeni_NC_018377: 0.000006, Stichopathes_sp__SCBUCN_8850_MZ157399: 0.000006): 0.002042, Stichopathes_sp__SCBUCN_8849_MZ157400: 0.046264): 0.479779): 0.150179, ((Myriopathes_japonica_NC_027667: 0.018051, Tanacetipathes_thamnea_NC_046843: 0.013122): 0.033548, Tylopathes_sp__n__NB_2020_MT318859: 0.046932): 0.186791): 0.037438, (((((((Bathypathes_sp__1_NB_2020_MT318844: 0.000000, Bathypathes_sp__n__2_NB_2020_MT318842: 0.000000): 0.000000, Stauropathes_arctica_MT318854: 0.000000): 0.007208, Bathypathes_sp__n__3_NB_2020_MT318843: 0.000000): 0.000000, Stauropathes_cf__punctata_NB_2020_MT318855: 0.014539): 0.000000, Telopathes_sp__NB_2020_MT318858: 0.014594): 0.030227, (Dendrobathypathes_sp__n__NB_2020_MT318845: 0.091727, ((Parantipathes_cf__hirondelle_NB_2020_MT318849: 0.000000, Parantipathes_hirondelle_MT318850: 0.000000): 0.000000, (Parantipathes_sp__NB_2020_MT318851: 0.000000, Sibopathes_cf__macrospina_NB_2020_MT318853: 0.000000): 0.000000): 0.011084): 0.028043): 0.087574, (Chrysopathes_formosa_NC_008411: 0.026842, Trissopathes_cf__tetracrada_NB_2020_MT318840: 0.027574): 0.003343): 0.075718): 0.040456, (Leiopathes_cf__glaberrima_NB_2020_MT318846: 0.000000, Leiopathes_expansa_MT318847: 0.000002): 0.178161): 0.241086): 0.305913, (Epizoanthus_illoricatus_NC_046474: 0.335363, (((Hydrozoanthus_gracilis_NC_046400: 0.053535, ((Neozoanthus_aff__uchina_AP_2020_MN873592: 0.049726, ((Zoanthus_cf__pulchellus_AP_2020_MN873599: 0.000000, ((Zoanthus_cf__sociatus_AP_2020_MN873600: 0.000000, Zoanthus_sociatus_NC_046476: 0.000000): 0.000000, Zoanthus_sp__AP_2020_MN873602: 0.000000): 0.000000): 0.006811, Zoanthus_sansibaricus_NC_035578: 0.000000): 0.050295): 0.045311, (Palythoa_heliodiscus_NC_035579: 0.027005, ((Palythoa_mizigama_NC_046403: 0.020083, Palythoa_mutuki_NC_046404: 0.006625): 0.000000, Sphenopus_marsupialis_NC_046406: 0.000000): 0.000000): 0.023045): 0.018159): 0.061227, ((Parazoanthus_elongatus_NC_046405: 0.020295, Parazoanthus_swiftii_NC_046475: 0.005972): 0.006909, Savalia_savaglia_DQ825686: 0.019108): 0.014657): 0.000008, (Microzoanthus_occultus_NC_046401: 0.532061, Nanozoanthus_harenaceus_NC_046402: 1.317545): 1.702829): 0.428658): 0.401495): 0.346139, (((((((Actinia_equina_NC_039929: 0.000006, Actinia_tenebrosa_NC_044902: 0.000000): 0.043762, (((Anemonia_manjano_NC_037178: 0.000002, Heteractis_aurora_NC_047219: 0.029471): 0.046375, (Anemonia_sulcata_NC_049065: 0.000000, Anemonia_viridis_NC_037177: 0.000000): 0.014035): 0.014284, Anthopleura_anjunae_NC_030274: 0.008276): 0.000000): 0.000006, Phymanthus_crucifer_NC_027614: 0.086117): 0.048563, ((((Bolocera_sp__BZ_2016_KU507297: 0.022051, (Bolocera_tuediae_NC_022470: 0.000002, Liponema_brevicorne_NC_047221: 0.007244): 0.000000): 0.000000, (Entacmaea_quadricolor_NC_049066: 0.018164, Isosicyonis_striata_KR051006: 0.007247): 0.000006): 0.057618, Stichodactyla_haddoni_MW760873: 0.178291): 0.008120, Epiactis_japonica_NC_047217: 0.120431): 0.000000): 0.140681, ((((Alicia_sansibarensis_NC_027610: 0.725190, (Haloclava_producta_NC_047218: 0.040114, Sagartia_ornata_NC_027615: 0.019933): 0.000018): 0.036942, (Diadumene_lineata_NC_045515: 0.222424, Metridium_senile_NC_000933: 0.067332): 0.066950): 0.139386, Paraphelliactis_xishaensis_MT997141: 0.268748): 0.000257, Antholoba_achates_NC_027611: 0.255068): 0.406986): 0.011161, Halcampoides_purpureus_NC_027612: 0.196502): 0.287308, Nematostella_sp__JVK_2006_NC_008164: 1.075316): 0.409377): 0.695059, (((((((Aurelia_aurita_NC_008446: 0.594159, (Aurelia_coerulea_NC_046792: 0.529180, Aurelia_limbata_NC_046691: 0.454320): 0.021685): 0.253494, (Aurelia_sp__3_sensu_Dawson_et_al___2005__LC005413: 0.547910, Aurelia_sp__4_sensu_Dawson_et_al___2005__LC005414: 1.163115): 0.249732): 1.682664, ((Nemopilema_nomurai_NC_035740: 0.932355, Rhopilema_esculentum_NC_035741: 0.687000): 0.552635, Stomolophus_sp__CG_2019_MK157198: 2.029870): 0.000022): 0.000018, Cassiopea_xamachana_NC_016466: 2.517031): 0.000021, (Chrysaora_pacifica_NC_046775: 0.000006, Chrysaora_quinquecirrha_NC_020459: 0.016340): 1.860967): 0.000055, ((((((Blackfordia_virginica_NC_053946: 0.727089, Eutima_sp__BMK_2020_MW066348: 2.266707): 0.612042, Laomedea_flexuosa_JN700945: 1.532871): 0.366728, (Clava_multicornis_NC_016465: 1.120641, (Turritopsis_dohrnii_NC_031213: 0.923540, Turritopsis_lata_NC_056128: 0.833167): 0.846344): 0.072045): 0.000071, Spirocodon_saltatrix_NC_049868: 1.343025): 0.000023, ((Hydra_oligactis_NC_010214: 0.969113, Hydra_sinensis_NC_021406: 0.992542): 0.666695, Nemalecium_lighti_MZ457217: 1.617514): 0.000041): 0.000238, (Craspedacusta_sowerbii_NC_018537: 3.916853, Cubaia_aphrodite_NC_016467: 2.207346): 0.635600): 0.127797): 0.712639, (Haliclystus_antarcticus_NC_030337: 1.390854, OU744455_1_OU744455_1__OU744455_1: 0.720931): 1.209170): 0.383961): 1.877085, Carijoa_riisei_NC_048963: 0.000007): 0.183821, (Sarcophyton_trocheliophorum_MK994517: 0.051953, (((Sinularia_acuta_MW987591: 0.008473, (Sinularia_maxima_MN485891: 0.008500, Sinularia_penghuensis_MW256412: 0.000002): 0.000006): 0.008405, Sinularia_cf__cruciata_GS_2016_NC_034318: 0.017628): 0.070128, (Sinularia_ceramensis_NC_044122: 0.000006, Sinularia_peculiaris_NC_018379: 0.008401): 0.012750): 0.000006): 0.043716): 0.032120, (((((((Antillogorgia_bipinnata_NC_008157: 0.066897, ((((Eugorgia_mutabilis_NC_035665: 0.016311, Leptogorgia_alba_NC_035669: 0.016317): 0.000004, (Leptogorgia_hebes_MN052675: 0.016135, Leptogorgia_virgulata_MK301586: 0.024385): 0.000006): 0.000006, Leptogorgia_sp__USNM_1437444_KY559412: 0.024536): 0.024493, Pacifigorgia_cairnsi_NC_035668: 0.058571): 0.024696): 0.000006, (((Leptogorgia_capverdensis_NC_035663: 0.000006, Leptogorgia_sarmentosa_NC_035670: 0.000006): 0.000006, Leptogorgia_gaini_KY559404: 0.033070): 0.024551, Leptogorgia_cf__palma_AP_2017_KY559406: 0.041033): 0.000006): 0.008306, (Eunicella_albicans_NC_035666: 0.041826, (Eunicella_cavolini_NC_035667: 0.000006, Eunicella_verrucosa_MW588805: 0.000006): 0.015902): 0.044433): 0.007439, (Calicogorgia_granulosa_NC_023345: 0.425129, Euplexaura_crassa_NC_020458: 0.059433): 0.000006): 0.009173, Muricea_crassa_NC_029697: 0.060402): 0.000006, ((((((Dendronephthya_castanea_NC_023343: 0.000006, Dendronephthya_mollis_NC_020456: 0.007795): 0.000006, Dendronephthya_gigantea_NC_013573: 0.000007): 0.007811, (Dendronephthya_putteri_NC_036022: 0.000006, Dendronephthya_suensoni_NC_022809: 0.000006): 0.000006): 0.033232, Scleronephthya_gracillimum_NC_023344: 0.040703): 0.125221, (Echinogorgia_complexa_NC_020457: 0.008453, Paramuricea_clavata_NC_034749: 0.102263): 0.030822): 0.011307, Trachythela_sp__YZ_2021_MW238423: 0.097014): 0.008482): 0.000006, (Incrustatus_comauensis_MT254531: 0.042489, Telestula_humilis_MT254527: 0.016403): 0.008594): 0.000004): 0.117728, Briareum_asbestinum_NC_008073: 0.153563): 0.004003, (Telestula_cf__batoni_AP_2020_MT254530: 0.016589, Telestula_septentrionalis_MT254532: 0.016794): 0.013878): 0.011854, (((Anthomastus_sp__USNM_1081145_KM015353: 0.041384, Anthomastus_sp__USNM_1171062_KM015352: 0.000006): 0.000006, (((Corallium_japonicum_AB595189: 0.000002, Corallium_rubrum_NC_022864: 0.000002): 0.049549, (Pleurocorallium_elatius_NC_022804: 0.000006, Pleurocorallium_konojoi_NC_015406: 0.000006): 0.058188): 0.000006, Sibogagorgia_cauliflora_NC_026193: 0.066744): 0.016605): 0.025336, Paraminabea_aldersladei_NC_018790: 0.050636): 0.016531): 0.008598, (((((((Anthoptilum_grandiflorum_NC_044086: 0.008187, Anthoptilum_sp__1_RH_2019_MK919656: 0.024972): 0.000002, (Umbellula_huxleyi_MK919668: 0.000006, Umbellula_sp__1_RH_2019_MK919669: 0.008020): 0.016343): 0.000006, Virgularia_mirabilis_NC_044091: 0.077798): 0.008162, (Funiculina_quadrangularis_NC_044078: 0.033127, ((Kophobelemnon_sp__1_RH_2019_MK919660: 0.015925, Kophobelemnon_sp__3_RH_2019_MK919661: 0.007935): 0.000007, Kophobelemnon_sp__4_RH_2019_MK919662: 0.025007): 0.008169): 0.000002): 0.000006, (Halipteris_cf__finmarchica_RH_2019_MK919659: 0.050955, Umbellula_sp__2_RH_2019_MK919670: 0.024540): 0.000006): 0.000002, ((((Distichoptilum_gracile_NC_044077: 0.031663, (Pennatula_cf__inflata_RH_2019_MK919666: 0.007902, Pennatula_grandis_NC_044088: 0.007829): 0.015908): 0.000002, ((Pennatula_aculeata_NC_044087: 0.000006, Pennatula_cf__aculeata_RH_2019_MK919664: 0.007894): 0.043785, Renilla_muelleri_NC_018378: 0.062377): 0.031355): 0.016123, Protoptilum_carpenteri_NC_044089: 0.049811): 0.000006, Stylatula_elongata_NC_018380: 0.091941): 0.016352): 0.019795, Junceella_fragilis_NC_024181: 0.157505): 0.022345): 0.016497, Heliopora_coerulea_NC_020375: 0.069241): 0.008116, ((Callogorgia_cf__gracilis_EE_2018_MH719202: 0.008548, (Plumarella_adhaerans_NC_046480: 0.000006, Plumarella_spinosa_NC_046465: 0.000006): 0.016745): 0.007861, Narella_hawaiiensis_NC_026192: 0.034127): 0.034702): 0.034342, Keratoisidinae_sp__BAL208_1_NC_010764: 0.033112);

**dN tree:**

(Acanella_arbuscula_NC_011016: 0.000005, ((((((((((((((((((((((((((((((Acropora_aculeus_KT001202: 0.000000, (Acropora_horrida_NC_022825: 0.000000, LC201842_1_LC201842_1__LC201842_1: 0.000000): 0.000000): 0.000000, LC201815_1_LC201815_1__LC201815_1: 0.000000): 0.000000, ((((Acropora_aspera_NC_022827: 0.000000, Acropora_muricata_NC_022824: 0.000000): 0.000000, LC201828_1_LC201828_1__LC201828_1: 0.000000): 0.000000, (((Acropora_digitifera_NC_022830: 0.000000, LC201849_1_LC201849_1__LC201849_1: 0.000000): 0.000000, Acropora_florida_NC_022828: 0.000000): 0.000000, Acropora_humilis_NC_022823: 0.000000): 0.000000): 0.000000, (Acropora_hyacinthus_NC_022826: 0.000000, LC201817_1_LC201817_1__LC201817_1: 0.000000): 0.000000): 0.000000): 0.000000, (Acropora_divaricata_NC_022832: 0.000000, (((Acropora_nasuta_NC_022831: 0.000000, Acropora_valida_NC_042742: 0.000000): 0.000000, LC201847_1_LC201847_1__LC201847_1: 0.000000): 0.000000, LC201820_1_LC201820_1__LC201820_1: 0.000000): 0.000000): 0.000000): 0.000000, Acropora_robusta_NC_022833: 0.002092): 0.000000, LC201813_1_LC201813_1__LC201813_1: 0.006321): 0.000000, ((Acropora_tenuis_NC_003522: 0.000001, Acropora_yongei_NC_022829: 0.000000): 0.000000, LC201816_1_LC201816_1__LC201816_1: 0.000000): 0.000000): 0.000000, (Isopora_palifera_NC_024091: 0.002106, Isopora_togianensis_NC_024089: 0.000000): 0.002094): 0.012538, ((Anacropora_matthai_NC_006898: 0.000000, Montipora_efflorescens_NC_040137: 0.000000): 0.000001, (Montipora_aequituberculata_NC_037359: 0.000000, Montipora_cactus_NC_006902: 0.000001): 0.000000): 0.006408): 0.000000, (Alveopora_japonica_NC_040136: 0.000000, Alveopora_sp__MFL_2014_KJ634271: 0.000000): 0.010734): 0.000001, (Astreopora_explanata_NC_024090: 0.002062, Astreopora_myriophthalma_NC_024092: 0.002108): 0.012549): 0.019508, ((Agaricia_humilis_NC_008160: 0.002209, Pavona_decussata_NC_026527: 0.004521): 0.011023, (Fimbriaphyllia_ancora_NC_015641: 0.038864, Galaxea_fascicularis_NC_029696: 0.005218): 0.007946): 0.000006): 0.001037, ((Pseudosiderastrea_formosa_NC_026530: 0.000001, Pseudosiderastrea_tayamai_NC_026531: 0.000001): 0.007774, Siderastrea_radians_NC_008167: 0.013746): 0.000003): 0.000001, ((((((Dendrophyllia_arbuscula_NC_027590: 0.002184, Tubastraea_coccinea_NC_026025: 0.000001): 0.000000, ((Tubastraea_diaphana_MK959042: 0.000000, Tubastraea_tagusensis_NC_030352: 0.000000): 0.000000, Tubastraea_micranthus_MK959041: 0.000000): 0.002172): 0.008752, Dendrophyllia_cribrosa_NC_026026: 0.000002): 0.002167, Turbinaria_peltata_NC_024671: 0.008668): 0.006433, (((Goniopora_columna_NC_015643: 0.002110, Goniopora_djiboutiensis_NC_045931: 0.000000): 0.002109, Goniopora_lobata_MN795054: 0.000001): 0.002107, ((Porites_fontanesii_NC_037434: 0.000005, ((Porites_panamensis_NC_024182: 0.000000, Porites_sverdrupi_KU956960: 0.000000): 0.000000, Porites_porites_NC_008166: 0.000000): 0.000000): 0.000000, (Porites_harrisoni_NC_037435: 0.000001, ((Porites_lobata_NC_030186: 0.000000, Porites_okinawensis_NC_015644: 0.000000): 0.000002, (Porites_lutea_NC_029695: 0.000000, Porites_rus_NC_027526: 0.002104): 0.000000): 0.000000): 0.000002): 0.006313): 0.000003): 0.011348, Fungiacyathus_stephanus_NC_015640: 0.031238): 0.011605): 0.009473, Gardineria_hawaiiensis_NC_056290: 0.023337): 0.016735, (((((Amplexidiscus_fenestrafer_NC_027101: 0.002174, (Discosoma_sp__MH308003: 0.004305, (Rhodactis_indosinensis_NC_027103: 0.004306, Rhodactis_sp__CASIZ_171755_NC_008158: 0.002133): 0.002172): 0.002133): 0.004338, (Discosoma_nummiforme_NC_027100: 0.008702, ((Discosoma_sp__CASIZ_168915_NC_008071: 0.000000, Discosoma_sp__CASIZ_168916_NC_008072: 0.000000): 0.000000, Platyzoanthus_mussoides_NC_027104: 0.002147): 0.004376): 0.000003): 0.009762, (Pseudocorynactis_sp__SIO_KP938437: 0.002168, (Ricordea_florida_NC_008159: 0.000004, Ricordea_yuma_NC_027106: 0.000003): 0.002136): 0.007652): 0.004528, Corynactis_californica_NC_027102: 0.021591): 0.009231, Corallimorphus_profundus_KP938440: 0.010179): 0.008952): 0.014049, ((((((Astrangia_sp__JVK_2006_NC_008161: 0.006350, (((Colpophyllia_natans_NC_008162: 0.002175, Mussa_angulosa_NC_008163: 0.002172): 0.000003, ((((((Dipsastraea_favus_NC_046690: 0.002168, Dipsastraea_rotumana_NC_044074: 0.000001): 0.002156, Favites_abdita_NC_035879: 0.000005): 0.000004, Platygyra_carnosa_NC_020049: 0.006609): 0.000000, (Favites_pentagona_NC_034916: 0.000003, Hydnophora_exesa_NC_042795: 0.000009): 0.000001): 0.000000, ((Orbicella_annularis_NC_007224: 0.000000, Orbicella_faveolata_NC_007226: 0.000000): 0.000000, Orbicella_franksi_NC_007225: 0.000002): 0.004358): 0.000002, Echinophyllia_aspera_NC_040169: 0.004397): 0.000000): 0.007948, Plesiastrea_versipora_NC_042481: 0.002915): 0.004644): 0.007489, (Polycyathus_sp__MFL_2011_NC_015642: 0.000000, Psammocora_nierstraszi_MT576637: 0.000002): 0.017945): 0.000034, Madrepora_oculata_NC_018364: 0.093787): 0.005032, (Madracis_myriaster_NC_011160: 0.000000, ((Pocillopora_damicornis_NC_009797: 0.000000, Pocillopora_grandis_NC_009798: 0.000000): 0.000001, ((Seriatopora_caliendrum_NC_010245: 0.002029, Seriatopora_hystrix_NC_010244: 0.000001): 0.000002, Stylophora_pistillata_NC_011162: 0.000002): 0.000000): 0.002076): 0.031921): 0.025247, ((Desmophyllum_dianthus_NC_034275: 0.002203, Desmophyllum_pertusum_NC_015143: 0.002202): 0.000000, Solenosmilia_variabilis_NC_025472: 0.000000): 0.039189): 0.018885, Paraconotrochus_antarcticus_NC_056275: 0.093494): 0.024437): 0.059283, ((((((Antipathes_cf__dichotoma_NB_2020_MT318841: 0.000002, Stichopathes_abyssicola_MT318856: 0.000002): 0.008994, (Phanopathes_sp__NB_2020_MT318852: 0.010335, Stichopathes_sp__n__NB_2020_MT318857: 0.006350): 0.000000): 0.003247, ((Stichopathes_luetkeni_NC_018377: 0.000000, Stichopathes_sp__SCBUCN_8850_MZ157399: 0.000000): 0.000000, Stichopathes_sp__SCBUCN_8849_MZ157400: 0.000005): 0.006885): 0.002414, ((Myriopathes_japonica_NC_027667: 0.002387, Tanacetipathes_thamnea_NC_046843: 0.000001): 0.000003, Tylopathes_sp__n__NB_2020_MT318859: 0.002471): 0.009832): 0.002790, (((((((Bathypathes_sp__1_NB_2020_MT318844: 0.000002, Bathypathes_sp__n__2_NB_2020_MT318842: 0.000002): 0.000002, Stauropathes_arctica_MT318854: 0.000002): 0.000001, Bathypathes_sp__n__3_NB_2020_MT318843: 0.000002): 0.000002, Stauropathes_cf__punctata_NB_2020_MT318855: 0.000001): 0.000002, Telopathes_sp__NB_2020_MT318858: 0.000001): 0.000003, (Dendrobathypathes_sp__n__NB_2020_MT318845: 0.002447, ((Parantipathes_cf__hirondelle_NB_2020_MT318849: 0.000002, Parantipathes_hirondelle_MT318850: 0.000002): 0.000002, (Parantipathes_sp__NB_2020_MT318851: 0.000002, Sibopathes_cf__macrospina_NB_2020_MT318853: 0.000002): 0.000002): 0.002415): 0.000003): 0.002405, (Chrysopathes_formosa_NC_008411: 0.007224, Trissopathes_cf__tetracrada_NB_2020_MT318840: 0.000003): 0.004793): 0.004452): 0.009718, (Leiopathes_cf__glaberrima_NB_2020_MT318846: 0.000002, Leiopathes_expansa_MT318847: 0.002393): 0.013109): 0.016025): 0.013608, (Epizoanthus_illoricatus_NC_046474: 0.004953, (((Hydrozoanthus_gracilis_NC_046400: 0.000005, ((Neozoanthus_aff__uchina_AP_2020_MN873592: 0.002663, ((Zoanthus_cf__pulchellus_AP_2020_MN873599: 0.000002, ((Zoanthus_cf__sociatus_AP_2020_MN873600: 0.000002, Zoanthus_sociatus_NC_046476: 0.000002): 0.000002, Zoanthus_sp__AP_2020_MN873602: 0.000002): 0.000002): 0.000001, Zoanthus_sansibaricus_NC_035578: 0.000002): 0.000005): 0.000005, (Palythoa_heliodiscus_NC_035579: 0.002676, ((Palythoa_mizigama_NC_046403: 0.000002, Palythoa_mutuki_NC_046404: 0.000001): 0.000002, Sphenopus_marsupialis_NC_046406: 0.000002): 0.000002): 0.002717): 0.000002): 0.002887, ((Parazoanthus_elongatus_NC_046405: 0.000002, Parazoanthus_swiftii_NC_046475: 0.000001): 0.000001, Savalia_savaglia_DQ825686: 0.002811): 0.005562): 0.002851, (Microzoanthus_occultus_NC_046401: 0.014881, Nanozoanthus_harenaceus_NC_046402: 0.042979): 0.024080): 0.005388): 0.026436): 0.004360, (((((((Actinia_equina_NC_039929: 0.000000, Actinia_tenebrosa_NC_044902: 0.000002): 0.002332, (((Anemonia_manjano_NC_037178: 0.002351, Heteractis_aurora_NC_047219: 0.002339): 0.000005, (Anemonia_sulcata_NC_049065: 0.000002, Anemonia_viridis_NC_037177: 0.000002): 0.004689): 0.000001, Anthopleura_anjunae_NC_030274: 0.004729): 0.000002): 0.000000, Phymanthus_crucifer_NC_027614: 0.002340): 0.009431, ((((Bolocera_sp__BZ_2016_KU507297: 0.009680, (Bolocera_tuediae_NC_022470: 0.002381, Liponema_brevicorne_NC_047221: 0.000001): 0.000002): 0.000002, (Entacmaea_quadricolor_NC_049066: 0.010857, Isosicyonis_striata_KR051006: 0.004771): 0.000000): 0.004849, Stichodactyla_haddoni_MW760873: 0.006828): 0.000001, Epiactis_japonica_NC_047217: 0.004821): 0.000002): 0.007452, ((((Alicia_sansibarensis_NC_027610: 0.146332, (Haloclava_producta_NC_047218: 0.004531, Sagartia_ornata_NC_027615: 0.000002): 0.003428): 0.012147, (Diadumene_lineata_NC_045515: 0.006282, Metridium_senile_NC_000933: 0.005174): 0.000007): 0.012192, Paraphelliactis_xishaensis_MT997141: 0.014154): 0.000000, Antholoba_achates_NC_027611: 0.017629): 0.005835): 0.004690, Halcampoides_purpureus_NC_027612: 0.011000): 0.021485, Nematostella_sp__JVK_2006_NC_008164: 0.047178): 0.019661): 0.048220, (((((((Aurelia_aurita_NC_008446: 0.028579, (Aurelia_coerulea_NC_046792: 0.007454, Aurelia_limbata_NC_046691: 0.012424): 0.003279): 0.004194, (Aurelia_sp__3_sensu_Dawson_et_al___2005__LC005413: 0.000055, Aurelia_sp__4_sensu_Dawson_et_al___2005__LC005414: 0.013604): 0.011008): 0.024388, ((Nemopilema_nomurai_NC_035740: 0.023992, Rhopilema_esculentum_NC_035741: 0.014271): 0.009626, Stomolophus_sp__CG_2019_MK157198: 0.025591): 0.004795): 0.005204, Cassiopea_xamachana_NC_016466: 0.083956): 0.011908, (Chrysaora_pacifica_NC_046775: 0.000000, Chrysaora_quinquecirrha_NC_020459: 0.002132): 0.027039): 0.054797, ((((((Blackfordia_virginica_NC_053946: 0.041938, Eutima_sp__BMK_2020_MW066348: 0.039726): 0.003654, Laomedea_flexuosa_JN700945: 0.039136): 0.033691, (Clava_multicornis_NC_016465: 0.051742, (Turritopsis_dohrnii_NC_031213: 0.025297, Turritopsis_lata_NC_056128: 0.015896): 0.025637): 0.031157): 0.024099, Spirocodon_saltatrix_NC_049868: 0.069185): 0.019171, ((Hydra_oligactis_NC_010214: 0.098592, Hydra_sinensis_NC_021406: 0.077296): 0.095950, Nemalecium_lighti_MZ457217: 0.174188): 0.032794): 0.114967, (Craspedacusta_sowerbii_NC_018537: 0.049095, Cubaia_aphrodite_NC_016467: 0.025273): 0.037737): 0.021925): 0.013947, (Haliclystus_antarcticus_NC_030337: 0.004220, OU744455_1_OU744455_1__OU744455_1: 0.017002): 0.067896): 0.021013): 0.076472, Carijoa_riisei_NC_048963: 0.002327): 0.004379, (Sarcophyton_trocheliophorum_MK994517: 0.002155, (((Sinularia_acuta_MW987591: 0.000001, (Sinularia_maxima_MN485891: 0.000001, Sinularia_penghuensis_MW256412: 0.002174): 0.000000): 0.000001, Sinularia_cf__cruciata_GS_2016_NC_034318: 0.004341): 0.002168, (Sinularia_ceramensis_NC_044122: 0.000000, Sinularia_peculiaris_NC_018379: 0.002175): 0.000001): 0.000000): 0.000004): 0.000003, (((((((Antillogorgia_bipinnata_NC_008157: 0.000007, ((((Eugorgia_mutabilis_NC_035665: 0.000002, Leptogorgia_alba_NC_035669: 0.000002): 0.004473, (Leptogorgia_hebes_MN052675: 0.000002, Leptogorgia_virgulata_MK301586: 0.000002): 0.000000): 0.000000, Leptogorgia_sp__USNM_1437444_KY559412: 0.004466): 0.000002, Pacifigorgia_cairnsi_NC_035668: 0.002258): 0.000002): 0.000000, (((Leptogorgia_capverdensis_NC_035663: 0.000000, Leptogorgia_sarmentosa_NC_035670: 0.000000): 0.000000, Leptogorgia_gaini_KY559404: 0.002223): 0.000002, Leptogorgia_cf__palma_AP_2017_KY559406: 0.004473): 0.000000): 0.002220, (Eunicella_albicans_NC_035666: 0.002239, (Eunicella_cavolini_NC_035667: 0.000000, Eunicella_verrucosa_MW588805: 0.000000): 0.002229): 0.011192): 0.000001, (Calicogorgia_granulosa_NC_023345: 0.023681, Euplexaura_crassa_NC_020458: 0.006784): 0.000000): 0.000001, Muricea_crassa_NC_029697: 0.002198): 0.000000, ((((((Dendronephthya_castanea_NC_023343: 0.000000, Dendronephthya_mollis_NC_020456: 0.000001): 0.000000, Dendronephthya_gigantea_NC_013573: 0.000000): 0.002284, (Dendronephthya_putteri_NC_036022: 0.000000, Dendronephthya_suensoni_NC_022809: 0.000000): 0.000000): 0.004556, Scleronephthya_gracillimum_NC_023344: 0.000004): 0.008987, (Echinogorgia_complexa_NC_020457: 0.004477, Paramuricea_clavata_NC_034749: 0.002253): 0.004458): 0.000001, Trachythela_sp__YZ_2021_MW238423: 0.006666): 0.000001): 0.000000, (Incrustatus_comauensis_MT254531: 0.006706, Telestula_humilis_MT254527: 0.002233): 0.002203): 0.004423): 0.006660, Briareum_asbestinum_NC_008073: 0.004468): 0.000000, (Telestula_cf__batoni_AP_2020_MT254530: 0.000002, Telestula_septentrionalis_MT254532: 0.000002): 0.000001): 0.002173, (((Anthomastus_sp__USNM_1081145_KM015353: 0.002198, Anthomastus_sp__USNM_1171062_KM015352: 0.000000): 0.000000, (((Corallium_japonicum_AB595189: 0.002238, Corallium_rubrum_NC_022864: 0.002240): 0.000005, (Pleurocorallium_elatius_NC_022804: 0.000000, Pleurocorallium_konojoi_NC_015406: 0.000000): 0.004489): 0.000000, Sibogagorgia_cauliflora_NC_026193: 0.006695): 0.000002): 0.002210, Paraminabea_aldersladei_NC_018790: 0.006596): 0.006618): 0.000001, (((((((Anthoptilum_grandiflorum_NC_044086: 0.002207, Anthoptilum_sp__1_RH_2019_MK919656: 0.000002): 0.002202, (Umbellula_huxleyi_MK919668: 0.000000, Umbellula_sp__1_RH_2019_MK919669: 0.004411): 0.006621): 0.000000, Virgularia_mirabilis_NC_044091: 0.006582): 0.000001, (Funiculina_quadrangularis_NC_044078: 0.002213, ((Kophobelemnon_sp__1_RH_2019_MK919660: 0.000002, Kophobelemnon_sp__3_RH_2019_MK919661: 0.000001): 0.006696, Kophobelemnon_sp__4_RH_2019_MK919662: 0.008918): 0.004403): 0.002215): 0.000000, (Halipteris_cf__finmarchica_RH_2019_MK919659: 0.006597, Umbellula_sp__2_RH_2019_MK919670: 0.008842): 0.000000): 0.002120, ((((Distichoptilum_gracile_NC_044077: 0.004520, (Pennatula_cf__inflata_RH_2019_MK919666: 0.004529, Pennatula_grandis_NC_044088: 0.000001): 0.000002): 0.002246, ((Pennatula_aculeata_NC_044087: 0.000000, Pennatula_cf__aculeata_RH_2019_MK919664: 0.000001): 0.004502, Renilla_muelleri_NC_018378: 0.008911): 0.006745): 0.000002, Protoptilum_carpenteri_NC_044089: 0.022692): 0.000000, Stylatula_elongata_NC_018380: 0.011276): 0.006771): 0.006724, Junceella_fragilis_NC_024181: 0.013372): 0.000002): 0.000002, Heliopora_coerulea_NC_020375: 0.004429): 0.004426, ((Callogorgia_cf__gracilis_EE_2018_MH719202: 0.000001, (Plumarella_adhaerans_NC_046480: 0.000000, Plumarella_spinosa_NC_046465: 0.000000): 0.006597): 0.002190, Narella_hawaiiensis_NC_026192: 0.004439): 0.002203): 0.006652, Keratoisidinae_sp__BAL208_1_NC_010764: 0.000003);

**w ratios as labels for TreeView:**

(Acanella_arbuscula_NC_011016 #0.0001 , ((((((((((((((((((((((((((((((Acropora_aculeus_KT001202 #0.0001 , (Acropora_horrida_NC_022825 #0.0001 , LC201842_1_LC201842_1__LC201842_1 #0.0001 ) #0.0001 ) #0.0001 , LC201815_1_LC201815_1__LC201815_1 #0.0001 ) #0.0001 , ((((Acropora_aspera_NC_022827 #0.0001 , Acropora_muricata_NC_022824 #0.0001 ) #0.0001 , LC201828_1_LC201828_1__LC201828_1 #0.0001 ) #0.0001 , (((Acropora_digitifera_NC_022830 #0.0001 , LC201849_1_LC201849_1__LC201849_1 #0.0001 ) #0.0001 , Acropora_florida_NC_022828 #0.0001 ) #0.0001 , Acropora_humilis_NC_022823 #0.0001 ) #0.0001 ) #0.0001 , (Acropora_hyacinthus_NC_022826 #0.0001 , LC201817_1_LC201817_1__LC201817_1 #0.0001 ) #0.0001 ) #0.0001 ) #0.0001 , (Acropora_divaricata_NC_022832 #0.0001 , (((Acropora_nasuta_NC_022831 #0.0001 , Acropora_valida_NC_042742 #0.0001 ) #0.0001 , LC201847_1_LC201847_1__LC201847_1 #0.0001 ) #0.0001 , LC201820_1_LC201820_1__LC201820_1 #0.0001 ) #0.0001 ) #0.0001 ) #0.0001 , Acropora_robusta_NC_022833 #983.561 ) #0.0001 , LC201813_1_LC201813_1__LC201813_1 #0.396446 ) #0.0001 , ((Acropora_tenuis_NC_003522 #0.0001 , Acropora_yongei_NC_022829 #0.0001 ) #0.0001 , LC201816_1_LC201816_1__LC201816_1 #0.0001 ) #0.0001 ) #0.0001 , (Isopora_palifera_NC_024091 #987.249 , Isopora_togianensis_NC_024089 #0.0001 ) #0.132369 ) #999 , ((Anacropora_matthai_NC_006898 #0.0001 , Montipora_efflorescens_NC_040137 #0.0001 ) #0.0001 , (Montipora_aequituberculata_NC_037359 #0.0001 , Montipora_cactus_NC_006902 #0.0001 ) #0.0001 ) #0.0322277 ) #0.0001 , (Alveopora_japonica_NC_040136 #0.0001 , Alveopora_sp__MFL_2014_KJ634271 #0.0001 ) #0.0501996 ) #0.0001 , (Astreopora_explanata_NC_024090 #971.93 , Astreopora_myriophthalma_NC_024092 #982.179 ) #458.433 ) #0.0806425 , ((Agaricia_humilis_NC_008160 #0.859692 , Pavona_decussata_NC_026527 #0.102578 ) #0.0616409 , (Fimbriaphyllia_ancora_NC_015641 #0.467053 , Galaxea_fascicularis_NC_029696 #0.155757 ) #0.0645334 ) #0.0001 ) #0.0320134 , ((Pseudosiderastrea_formosa_NC_026530 #0.0001 , Pseudosiderastrea_tayamai_NC_026531 #0.0001 ) #0.169892 , Siderastrea_radians_NC_008167 #0.263038 ) #0.0001 ) #0.0001 , ((((((Dendrophyllia_arbuscula_NC_027590 #0.281575 , Tubastraea_coccinea_NC_026025 #0.0001 ) #0.0001 , ((Tubastraea_diaphana_MK959042 #0.0001 , Tubastraea_tagusensis_NC_030352 #0.0001 ) #0.0001 , Tubastraea_micranthus_MK959041 #0.0001 ) #999 ) #1.12897 , Dendrophyllia_cribrosa_NC_026026 #0.0001 ) #0.0384163 , Turbinaria_peltata_NC_024671 #0.238887 ) #0.187001 , (((Goniopora_columna_NC_015643 #0.127331 , Goniopora_djiboutiensis_NC_045931 #0.0001 ) #958.434 , Goniopora_lobata_MN795054 #0.0001 ) #0.0351176 , ((Porites_fontanesii_NC_037434 #0.0001 , ((Porites_panamensis_NC_024182 #0.0001 , Porites_sverdrupi_KU956960 #0.0001 ) #0.0001 , Porites_porites_NC_008166 #0.0001 ) #0.0001 ) #0.0001 , (Porites_harrisoni_NC_037435 #0.0001 , ((Porites_lobata_NC_030186 #0.0001 , Porites_okinawensis_NC_015644 #0.0001 ) #0.0001 , (Porites_lutea_NC_029695 #0.0001 , Porites_rus_NC_027526 #963.867 ) #0.0001 ) #0.0001 ) #0.0001 ) #0.0886209 ) #0.0001 ) #1.21583 , Fungiacyathus_stephanus_NC_015640 #0.323989 ) #0.539292 ) #0.0991845 , Gardineria_hawaiiensis_NC_056290 #0.286056 ) #0.0949958 , (((((Amplexidiscus_fenestrafer_NC_027101 #0.279533 , (Discosoma_sp__MH308003 #999 , (Rhodactis_indosinensis_NC_027103 #0.542542 , Rhodactis_sp__CASIZ_171755_NC_008158 #979.999 ) #0.274388 ) #0.133871 ) #0.184637 , (Discosoma_nummiforme_NC_027100 #999 , ((Discosoma_sp__CASIZ_168915_NC_008071 #0.0001 , Discosoma_sp__CASIZ_168916_NC_008072 #0.0001 ) #0.0001 , Platyzoanthus_mussoides_NC_027104 #992.926 ) #0.138628 ) #0.0001 ) #0.229857 , (Pseudocorynactis_sp__SIO_KP938437 #0.0135622 , (Ricordea_florida_NC_008159 #0.0001 , Ricordea_yuma_NC_027106 #0.0001 ) #0.0322998 ) #0.113318 ) #0.0450369 , Corynactis_californica_NC_027102 #0.188312 ) #0.145009 , Corallimorphus_profundus_KP938440 #0.101956 ) #0.0603583 ) #0.0823908 , ((((((Astrangia_sp__JVK_2006_NC_008161 #0.160663 , (((Colpophyllia_natans_NC_008162 #0.258724 , Mussa_angulosa_NC_008163 #0.0437148 ) #0.0001 , ((((((Dipsastraea_favus_NC_046690 #949.042 , Dipsastraea_rotumana_NC_044074 #0.0001 ) #0.0835846 , Favites_abdita_NC_035879 #0.0001 ) #0.0001 , Platygyra_carnosa_NC_020049 #0.0531898 ) #0.0001 , (Favites_pentagona_NC_034916 #0.0001 , Hydnophora_exesa_NC_042795 #0.0001 ) #0.0001 ) #0.0001 , ((Orbicella_annularis_NC_007224 #0.0001 , Orbicella_faveolata_NC_007226 #0.0001 ) #0.0001 , Orbicella_franksi_NC_007225 #0.0001 ) #0.103353 ) #0.0001 , Echinophyllia_aspera_NC_040169 #0.0309907 ) #0.0001 ) #0.0423148 , Plesiastrea_versipora_NC_042481 #0.270562 ) #0.0212809 ) #0.0587578 , (Polycyathus_sp__MFL_2011_NC_015642 #0.0001 , Psammocora_nierstraszi_MT576637 #0.0001 ) #0.0312322 ) #0.0001 , Madrepora_oculata_NC_018364 #0.0803216 ) #0.00820415 , (Madracis_myriaster_NC_011160 #0.0001 , ((Pocillopora_damicornis_NC_009797 #0.0001 , Pocillopora_grandis_NC_009798 #0.0001 ) #0.0001 , ((Seriatopora_caliendrum_NC_010245 #0.119151 , Seriatopora_hystrix_NC_010244 #0.0001 ) #0.0001 , Stylophora_pistillata_NC_011162 #0.0001 ) #0.0001 ) #0.0138614 ) #0.0830285 ) #649.421 , ((Desmophyllum_dianthus_NC_034275 #0.282406 , Desmophyllum_pertusum_NC_015143 #0.280799 ) #0.0001 , Solenosmilia_variabilis_NC_025472 #0.0001 ) #0.0209568 ) #0.0227842 , Paraconotrochus_antarcticus_NC_056275 #0.0559184 ) #0.0856134 ) #0.1159 , ((((((Antipathes_cf__dichotoma_NB_2020_MT318841 #38.1582 , Stichopathes_abyssicola_MT318856 #46.3573 ) #0.0400835 , (Phanopathes_sp__NB_2020_MT318852 #0.0627391 , Stichopathes_sp__n__NB_2020_MT318857 #0.036815 ) #0.0001 ) #0.0348926 , ((Stichopathes_luetkeni_NC_018377 #0.0001 , Stichopathes_sp__SCBUCN_8850_MZ157399 #0.0001 ) #0.0001 , Stichopathes_sp__SCBUCN_8849_MZ157400 #0.0001 ) #0.0143506 ) #0.0160711 , ((Myriopathes_japonica_NC_027667 #0.132227 , Tanacetipathes_thamnea_NC_046843 #0.0001 ) #0.0001 , Tylopathes_sp__n__NB_2020_MT318859 #0.0526453 ) #0.0526369 ) #0.0745195 , (((((((Bathypathes_sp__1_NB_2020_MT318844 #105.462 , Bathypathes_sp__n__2_NB_2020_MT318842 #100.939 ) #22.2855 , Stauropathes_arctica_MT318854 #47.5469 ) #0.0001 , Bathypathes_sp__n__3_NB_2020_MT318843 #22.0884 ) #30.1112 , Stauropathes_cf__punctata_NB_2020_MT318855 #0.0001 ) #95.6381 , Telopathes_sp__NB_2020_MT318858 #0.0001 ) #0.0001 , (Dendrobathypathes_sp__n__NB_2020_MT318845 #0.0266771 , ((Parantipathes_cf__hirondelle_NB_2020_MT318849 #117.696 , Parantipathes_hirondelle_MT318850 #86.9995 ) #103.726 , (Parantipathes_sp__NB_2020_MT318851 #91.2299 , Sibopathes_cf__macrospina_NB_2020_MT318853 #71.886 ) #88.9688 ) #0.217926 ) #0.0001 ) #0.0274622 , (Chrysopathes_formosa_NC_008411 #0.269108 , Trissopathes_cf__tetracrada_NB_2020_MT318840 #0.0001 ) #1.434 ) #0.0587974 ) #0.240218 , (Leiopathes_cf__glaberrima_NB_2020_MT318846 #71.1854 , Leiopathes_expansa_MT318847 #999 ) #0.0735792 ) #0.0664699 ) #0.0444832 , (Epizoanthus_illoricatus_NC_046474 #0.0147689 , (((Hydrozoanthus_gracilis_NC_046400 #0.0001 , ((Neozoanthus_aff__uchina_AP_2020_MN873592 #0.0535564 , ((Zoanthus_cf__pulchellus_AP_2020_MN873599 #47.584 , ((Zoanthus_cf__sociatus_AP_2020_MN873600 #47.8526 , Zoanthus_sociatus_NC_046476 #43.4662 ) #64.9778 , Zoanthus_sp__AP_2020_MN873602 #62.8544 ) #62.9902 ) #0.0001 , Zoanthus_sansibaricus_NC_035578 #41.2107 ) #0.0001 ) #0.0001 , (Palythoa_heliodiscus_NC_035579 #0.0990945 , ((Palythoa_mizigama_NC_046403 #0.0001 , Palythoa_mutuki_NC_046404 #0.0001 ) #48.775 , Sphenopus_marsupialis_NC_046406 #74.2914 ) #49.5947 ) #0.117906 ) #0.0001 ) #0.047159 , ((Parazoanthus_elongatus_NC_046405 #0.0001 , Parazoanthus_swiftii_NC_046475 #0.0001 ) #0.0001 , Savalia_savaglia_DQ825686 #0.147125 ) #0.379507 ) #362.481 , (Microzoanthus_occultus_NC_046401 #0.0279694 , Nanozoanthus_harenaceus_NC_046402 #0.0326203 ) #0.0141413 ) #0.0125697 ) #0.0658435 ) #0.0125948 , (((((((Actinia_equina_NC_039929 #0.000100129 , Actinia_tenebrosa_NC_044902 #97.7688 ) #0.0532883 , (((Anemonia_manjano_NC_037178 #999 , Heteractis_aurora_NC_047219 #0.0793825 ) #0.0001 , (Anemonia_sulcata_NC_049065 #94.6371 , Anemonia_viridis_NC_037177 #86.9773 ) #0.334119 ) #0.0001 , Anthopleura_anjunae_NC_030274 #0.57144 ) #45.8279 ) #0.0001 , Phymanthus_crucifer_NC_027614 #0.0271693 ) #0.194195 , ((((Bolocera_sp__BZ_2016_KU507297 #0.438994 , (Bolocera_tuediae_NC_022470 #999 , Liponema_brevicorne_NC_047221 #0.0001 ) #81.2102 ) #47.3843 , (Entacmaea_quadricolor_NC_049066 #0.59771 , Isosicyonis_striata_KR051006 #0.658356 ) #0.0001 ) #0.0841563 , Stichodactyla_haddoni_MW760873 #0.0382962 ) #0.0001 , Epiactis_japonica_NC_047217 #0.040027 ) #48.7269 ) #0.0529683 , ((((Alicia_sansibarensis_NC_027610 #0.201784 , (Haloclava_producta_NC_047218 #0.112952 , Sagartia_ornata_NC_027615 #0.0001 ) #194.827 ) #0.328811 , (Diadumene_lineata_NC_045515 #0.0282414 , Metridium_senile_NC_000933 #0.0768377 ) #0.0001 ) #0.0874679 , Paraphelliactis_xishaensis_MT997141 #0.0526647 ) #0.0001 , Antholoba_achates_NC_027611 #0.069114 ) #0.0143368 ) #0.420222 , Halcampoides_purpureus_NC_027612 #0.0559813 ) #0.0747805 , Nematostella_sp__JVK_2006_NC_008164 #0.0438734 ) #0.0480263 ) #0.0693748 , (((((((Aurelia_aurita_NC_008446 #0.0480995 , (Aurelia_coerulea_NC_046792 #0.0140852 , Aurelia_limbata_NC_046691 #0.027347 ) #0.151224 ) #0.016545 , (Aurelia_sp__3_sensu_Dawson_et_al___2005__LC005413 #0.0001 , Aurelia_sp__4_sensu_Dawson_et_al___2005__LC005414 #0.0116961 ) #0.0440792 ) #0.0144934 , ((Nemopilema_nomurai_NC_035740 #0.025733 , Rhopilema_esculentum_NC_035741 #0.0207732 ) #0.0174177 , Stomolophus_sp__CG_2019_MK157198 #0.0126071 ) #222.517 ) #289.652 , Cassiopea_xamachana_NC_016466 #0.0333552 ) #554.64 , (Chrysaora_pacifica_NC_046775 #0.0001 , Chrysaora_quinquecirrha_NC_020459 #0.130485 ) #0.0145297 ) #999 , ((((((Blackfordia_virginica_NC_053946 #0.05768 , Eutima_sp__BMK_2020_MW066348 #0.0175259 ) #0.00597098 , Laomedea_flexuosa_JN700945 #0.025531 ) #0.0918686 , (Clava_multicornis_NC_016465 #0.0461714 , (Turritopsis_dohrnii_NC_031213 #0.0273918 , Turritopsis_lata_NC_056128 #0.019079 ) #0.0302909 ) #0.432464 ) #339.447 , Spirocodon_saltatrix_NC_049868 #0.0515144 ) #821.121 , ((Hydra_oligactis_NC_010214 #0.101734 , Hydra_sinensis_NC_021406 #0.0778766 ) #0.143919 , Nemalecium_lighti_MZ457217 #0.107689 ) #797.616 ) #483.751 , (Craspedacusta_sowerbii_NC_018537 #0.0125344 , Cubaia_aphrodite_NC_016467 #0.0114493 ) #0.0593725 ) #0.17156 ) #0.0195705 , (Haliclystus_antarcticus_NC_030337 #0.00303402 , OU744455_1_OU744455_1__OU744455_1 #0.0235835 ) #0.0561505 ) #0.0547276 ) #0.0407398 , Carijoa_riisei_NC_048963 #315.136 ) #0.0238212 , (Sarcophyton_trocheliophorum_MK994517 #0.0414861 , (((Sinularia_acuta_MW987591 #0.0001 , (Sinularia_maxima_MN485891 #0.0001 , Sinularia_penghuensis_MW256412 #975.086 ) #0.0001 ) #0.0001 , Sinularia_cf__cruciata_GS_2016_NC_034318 #0.246248 ) #0.0309205 , (Sinularia_ceramensis_NC_044122 #0.0001 , Sinularia_peculiaris_NC_018379 #0.258852 ) #0.0001 ) #0.0001 ) #0.0001 ) #0.0001 , (((((((Antillogorgia_bipinnata_NC_008157 #0.0001 , ((((Eugorgia_mutabilis_NC_035665 #0.0001 , Leptogorgia_alba_NC_035669 #0.0001 ) #999 , (Leptogorgia_hebes_MN052675 #0.0001 , Leptogorgia_virgulata_MK301586 #0.0001 ) #0.0001 ) #0.0001 , Leptogorgia_sp__USNM_1437444_KY559412 #0.182023 ) #0.0001 , Pacifigorgia_cairnsi_NC_035668 #0.0385435 ) #0.0001 ) #0.0001 , (((Leptogorgia_capverdensis_NC_035663 #0.0001 , Leptogorgia_sarmentosa_NC_035670 #0.0001 ) #0.0001 , Leptogorgia_gaini_KY559404 #0.0672133 ) #0.0001 , Leptogorgia_cf__palma_AP_2017_KY559406 #0.109019 ) #0.0001 ) #0.267301 , (Eunicella_albicans_NC_035666 #0.0535414 , (Eunicella_cavolini_NC_035667 #0.0001 , Eunicella_verrucosa_MW588805 #0.0001 ) #0.140146 ) #0.251879 ) #0.0001 , (Calicogorgia_granulosa_NC_023345 #0.0557035 , Euplexaura_crassa_NC_020458 #0.114145 ) #0.0001 ) #0.0001 , Muricea_crassa_NC_029697 #0.0363825 ) #0.0001 , ((((((Dendronephthya_castanea_NC_023343 #0.0001 , Dendronephthya_mollis_NC_020456 #0.0001 ) #0.0001 , Dendronephthya_gigantea_NC_013573 #0.0001 ) #0.292353 , (Dendronephthya_putteri_NC_036022 #0.0001 , Dendronephthya_suensoni_NC_022809 #0.0001 ) #0.0001 ) #0.13711 , Scleronephthya_gracillimum_NC_023344 #0.0001 ) #0.0717712 , (Echinogorgia_complexa_NC_020457 #0.529665 , Paramuricea_clavata_NC_034749 #0.0220351 ) #0.144624 ) #0.0001 , Trachythela_sp__YZ_2021_MW238423 #0.0687113 ) #0.0001 ) #0.0001 , (Incrustatus_comauensis_MT254531 #0.157834 , Telestula_humilis_MT254527 #0.136152 ) #0.256348 ) #999 ) #0.0565747 , Briareum_asbestinum_NC_008073 #0.0290938 ) #0.0001 , (Telestula_cf__batoni_AP_2020_MT254530 #0.0001 , Telestula_septentrionalis_MT254532 #0.0001 ) #0.0001 ) #0.183274 , (((Anthomastus_sp__USNM_1081145_KM015353 #0.0531192 , Anthomastus_sp__USNM_1171062_KM015352 #0.0001 ) #0.0001 , (((Corallium_japonicum_AB595189 #999 , Corallium_rubrum_NC_022864 #999 ) #0.0001 , (Pleurocorallium_elatius_NC_022804 #0.0001 , Pleurocorallium_konojoi_NC_015406 #0.0001 ) #0.0771381 ) #0.0001 , Sibogagorgia_cauliflora_NC_026193 #0.100301 ) #0.0001 ) #0.0872273 , Paraminabea_aldersladei_NC_018790 #0.130257 ) #0.400342 ) #0.0001 , (((((((Anthoptilum_grandiflorum_NC_044086 #0.269543 , Anthoptilum_sp__1_RH_2019_MK919656 #0.0001 ) #982.498 , (Umbellula_huxleyi_MK919668 #0.0001 , Umbellula_sp__1_RH_2019_MK919669 #0.550058 ) #0.405112 ) #0.0001 , Virgularia_mirabilis_NC_044091 #0.0846062 ) #0.0001 , (Funiculina_quadrangularis_NC_044078 #0.0668173 , ((Kophobelemnon_sp__1_RH_2019_MK919660 #0.0001 , Kophobelemnon_sp__3_RH_2019_MK919661 #0.0001 ) #999 , Kophobelemnon_sp__4_RH_2019_MK919662 #0.356635 ) #0.538981 ) #964.635 ) #0.0001 , (Halipteris_cf__finmarchica_RH_2019_MK919659 #0.129469 , Umbellula_sp__2_RH_2019_MK919670 #0.360294 ) #0.0001 ) #968.224 , ((((Distichoptilum_gracile_NC_044077 #0.142764 , (Pennatula_cf__inflata_RH_2019_MK919666 #0.57308 , Pennatula_grandis_NC_044088 #0.0001 ) #0.0001 ) #988.491 , ((Pennatula_aculeata_NC_044087 #0.0001 , Pennatula_cf__aculeata_RH_2019_MK919664 #0.0001 ) #0.102821 , Renilla_muelleri_NC_018378 #0.142854 ) #0.215105 ) #0.0001 , Protoptilum_carpenteri_NC_044089 #0.455572 ) #0.0001 , Stylatula_elongata_NC_018380 #0.122639 ) #0.41406 ) #0.339688 , Junceella_fragilis_NC_024181 #0.0848986 ) #0.0001 ) #0.0001 , Heliopora_coerulea_NC_020375 #0.0639713 ) #0.545254 , ((Callogorgia_cf__gracilis_EE_2018_MH719202 #0.0001 , (Plumarella_adhaerans_NC_046480 #0.0001 , Plumarella_spinosa_NC_046465 #0.0001 ) #0.394 ) #0.278555 , Narella_hawaiiensis_NC_026192 #0.13008 ) #0.063478 ) #0.193706 , Keratoisidinae_sp__BAL208_1_NC_010764 #0.0001 );

NAD1:

**dS tree:**

(Acanella_arbuscula_NC_011016: 0.021431, ((((((((((((((((((((((((((((((Acropora_aculeus_KT001202: 0.000000, (Acropora_horrida_NC_022825: 0.000000, LC201842_1_LC201842_1__LC201842_1: 0.005105): 0.000000): 0.000000, LC201815_1_LC201815_1__LC201815_1: 0.000000): 0.000000, ((((Acropora_aspera_NC_022827: 0.000000, Acropora_muricata_NC_022824: 0.000000): 0.000000, LC201828_1_LC201828_1__LC201828_1: 0.000000): 0.000000, (((Acropora_digitifera_NC_022830: 0.000000, LC201849_1_LC201849_1__LC201849_1: 0.000000): 0.000000, Acropora_florida_NC_022828: 0.000000): 0.000000, Acropora_humilis_NC_022823: 0.000000): 0.000000): 0.000000, (Acropora_hyacinthus_NC_022826: 0.000000, LC201817_1_LC201817_1__LC201817_1: 0.000000): 0.000000): 0.000000): 0.000000, (Acropora_divaricata_NC_022832: 0.000000, (((Acropora_nasuta_NC_022831: 0.000000, Acropora_valida_NC_042742: 0.000000): 0.000000, LC201847_1_LC201847_1__LC201847_1: 0.000000): 0.005113, LC201820_1_LC201820_1__LC201820_1: 0.000000): 0.000000): 0.000000): 0.000000, Acropora_robusta_NC_022833: 0.000000): 0.000002, LC201813_1_LC201813_1__LC201813_1: 0.000000): 0.000005, ((Acropora_tenuis_NC_003522: 0.000000, Acropora_yongei_NC_022829: 0.000000): 0.000000, LC201816_1_LC201816_1__LC201816_1: 0.000000): 0.010229): 0.020658, (Isopora_palifera_NC_024091: 0.000005, Isopora_togianensis_NC_024089: 0.005125): 0.015761): 0.072104, ((Anacropora_matthai_NC_006898: 0.000002, Montipora_efflorescens_NC_040137: 0.005092): 0.000000, (Montipora_aequituberculata_NC_037359: 0.015270, Montipora_cactus_NC_006902: 0.000000): 0.005077): 0.064126): 0.011852, (Alveopora_japonica_NC_040136: 0.002465, Alveopora_sp__MFL_2014_KJ634271: 0.007908): 0.130401): 0.000005, (Astreopora_explanata_NC_024090: 0.000005, Astreopora_myriophthalma_NC_024092: 0.000005): 0.015914): 0.025984, ((Agaricia_humilis_NC_008160: 0.015134, Pavona_decussata_NC_026527: 0.000002): 0.145921, (Fimbriaphyllia_ancora_NC_015641: 0.000005, Galaxea_fascicularis_NC_029696: 0.020793): 0.071672): 0.009349): 0.027073, ((Pseudosiderastrea_formosa_NC_026530: 0.014081, Pseudosiderastrea_tayamai_NC_026531: 0.012118): 0.064552, Siderastrea_radians_NC_008167: 0.042693): 0.049318): 0.037675, ((((((Dendrophyllia_arbuscula_NC_027590: 0.010632, Tubastraea_coccinea_NC_026025: 0.000005): 0.000005, ((Tubastraea_diaphana_MK959042: 0.000005, Tubastraea_tagusensis_NC_030352: 0.000002): 0.000005, Tubastraea_micranthus_MK959041: 0.000005): 0.000002): 0.005303, Dendrophyllia_cribrosa_NC_026026: 0.005343): 0.015293, Turbinaria_peltata_NC_024671: 0.016943): 0.004084, (((Goniopora_columna_NC_015643: 0.005406, Goniopora_djiboutiensis_NC_045931: 0.000005): 0.027924, Goniopora_lobata_MN795054: 0.022728): 0.069778, ((Porites_fontanesii_NC_037434: 0.011046, ((Porites_panamensis_NC_024182: 0.005406, Porites_sverdrupi_KU956960: 0.000005): 0.005294, Porites_porites_NC_008166: 0.005531): 0.022058): 0.000005, (Porites_harrisoni_NC_037435: 0.010885, ((Porites_lobata_NC_030186: 0.000005, Porites_okinawensis_NC_015644: 0.000005): 0.005433, (Porites_lutea_NC_029695: 0.005449, Porites_rus_NC_027526: 0.010960): 0.000005): 0.000005): 0.005579): 0.054951): 0.054433): 0.027610, Fungiacyathus_stephanus_NC_015640: 0.047587): 0.047835): 0.210884, Gardineria_hawaiiensis_NC_056290: 0.194272): 0.075706, (((((Amplexidiscus_fenestrafer_NC_027101: 0.010312, (Discosoma_sp__MH308003: 0.000002, (Rhodactis_indosinensis_NC_027103: 0.010170, Rhodactis_sp__CASIZ_171755_NC_008158: 0.000000): 0.000000): 0.000002): 0.020640, (Discosoma_nummiforme_NC_027100: 0.010290, ((Discosoma_sp__CASIZ_168915_NC_008071: 0.000000, Discosoma_sp__CASIZ_168916_NC_008072: 0.000000): 0.000000, Platyzoanthus_mussoides_NC_027104: 0.005127): 0.005117): 0.005301): 0.017555, (Pseudocorynactis_sp__SIO_KP938437: 0.154047, (Ricordea_florida_NC_008159: 0.015147, Ricordea_yuma_NC_027106: 0.043880): 0.071816): 0.031601): 0.046072, Corynactis_californica_NC_027102: 0.078126): 0.051569, Corallimorphus_profundus_KP938440: 0.115877): 0.084550): 0.233300, ((((((Astrangia_sp__JVK_2006_NC_008161: 0.056502, (((Colpophyllia_natans_NC_008162: 0.005849, Mussa_angulosa_NC_008163: 0.005909): 0.025492, ((((((Dipsastraea_favus_NC_046690: 0.000005, Dipsastraea_rotumana_NC_044074: 0.024040): 0.005932, Favites_abdita_NC_035879: 0.011919): 0.000005, Platygyra_carnosa_NC_020049: 0.005940): 0.000005, (Favites_pentagona_NC_034916: 0.011933, Hydnophora_exesa_NC_042795: 0.011967): 0.000005): 0.017992, ((Orbicella_annularis_NC_007224: 0.000005, Orbicella_faveolata_NC_007226: 0.000005): 0.000005, Orbicella_franksi_NC_007225: 0.005991): 0.050490): 0.042159, Echinophyllia_aspera_NC_040169: 0.038354): 0.006300): 0.104835, Plesiastrea_versipora_NC_042481: 0.052377): 0.091749): 0.072906, (Polycyathus_sp__MFL_2011_NC_015642: 0.003926, Psammocora_nierstraszi_MT576637: 0.015356): 0.345033): 0.183083, Madrepora_oculata_NC_018364: 0.440723): 0.145522, (Madracis_myriaster_NC_011160: 0.074243, ((Pocillopora_damicornis_NC_009797: 0.000005, Pocillopora_grandis_NC_009798: 0.000005): 0.036725, ((Seriatopora_caliendrum_NC_010245: 0.006101, Seriatopora_hystrix_NC_010244: 0.006316): 0.021592, Stylophora_pistillata_NC_011162: 0.017201): 0.005616): 0.036286): 0.471456): 0.057532, ((Desmophyllum_dianthus_NC_034275: 0.005645, Desmophyllum_pertusum_NC_015143: 0.000005): 0.005667, Solenosmilia_variabilis_NC_025472: 0.000005): 0.525339): 0.346841, Paraconotrochus_antarcticus_NC_056275: 1.775090): 0.803382): 0.099182, ((((((Antipathes_cf__dichotoma_NB_2020_MT318841: 0.000000, Stichopathes_abyssicola_MT318856: 0.000000): 0.144750, (Phanopathes_sp__NB_2020_MT318852: 0.127597, Stichopathes_sp__n__NB_2020_MT318857: 0.124463): 0.053921): 0.095403, ((Stichopathes_luetkeni_NC_018377: 0.000005, Stichopathes_sp__SCBUCN_8850_MZ157399: 0.005539): 0.000005, Stichopathes_sp__SCBUCN_8849_MZ157400: 0.011131): 0.264529): 0.032615, ((Myriopathes_japonica_NC_027667: 0.015906, Tanacetipathes_thamnea_NC_046843: 0.009826): 0.011567, Tylopathes_sp__n__NB_2020_MT318859: 0.048061): 0.153895): 0.022068, (((((((Bathypathes_sp__1_NB_2020_MT318844: 0.000000, Bathypathes_sp__n__2_NB_2020_MT318842: 0.000000): 0.000000, Stauropathes_arctica_MT318854: 0.000000): 0.000000, Bathypathes_sp__n__3_NB_2020_MT318843: 0.000000): 0.021058, Stauropathes_cf__punctata_NB_2020_MT318855: 0.015980): 0.004826, Telopathes_sp__NB_2020_MT318858: 0.000004): 0.022611, (Dendrobathypathes_sp__n__NB_2020_MT318845: 0.037670, ((Parantipathes_cf__hirondelle_NB_2020_MT318849: 0.000000, Parantipathes_hirondelle_MT318850: 0.000000): 0.000000, (Parantipathes_sp__NB_2020_MT318851: 0.000000, Sibopathes_cf__macrospina_NB_2020_MT318853: 0.000000): 0.000000): 0.015001): 0.033118): 0.038283, (Chrysopathes_formosa_NC_008411: 0.012331, Trissopathes_cf__tetracrada_NB_2020_MT318840: 0.019882): 0.034614): 0.119990): 0.016003, (Leiopathes_cf__glaberrima_NB_2020_MT318846: 0.000000, Leiopathes_expansa_MT318847: 0.000000): 0.073317): 0.330198): 0.136905, (Epizoanthus_illoricatus_NC_046474: 0.094347, (((Hydrozoanthus_gracilis_NC_046400: 0.078158, ((Neozoanthus_aff__uchina_AP_2020_MN873592: 0.026049, ((Zoanthus_cf__pulchellus_AP_2020_MN873599: 0.009948, ((Zoanthus_cf__sociatus_AP_2020_MN873600: 0.000000, Zoanthus_sociatus_NC_046476: 0.000000): 0.004983, Zoanthus_sp__AP_2020_MN873602: 0.000000): 0.000000): 0.000005, Zoanthus_sansibaricus_NC_035578: 0.004961): 0.030634): 0.023131, (Palythoa_heliodiscus_NC_035579: 0.015238, ((Palythoa_mizigama_NC_046403: 0.000002, Palythoa_mutuki_NC_046404: 0.004965): 0.000005, Sphenopus_marsupialis_NC_046406: 0.014942): 0.010064): 0.007181): 0.015437): 0.034072, ((Parazoanthus_elongatus_NC_046405: 0.010101, Parazoanthus_swiftii_NC_046475: 0.000000): 0.037332, Savalia_savaglia_DQ825686: 0.034959): 0.000000): 0.042398, (Microzoanthus_occultus_NC_046401: 0.835862, Nanozoanthus_harenaceus_NC_046402: 0.576418): 1.826317): 0.000005): 0.451962): 0.226887, (((((((Actinia_equina_NC_039929: 0.000005, Actinia_tenebrosa_NC_044902: 0.000005): 0.005299, (((Anemonia_manjano_NC_037178: 0.026786, Heteractis_aurora_NC_047219: 0.000005): 0.026872, (Anemonia_sulcata_NC_049065: 0.000005, Anemonia_viridis_NC_037177: 0.000005): 0.021487): 0.000005, Anthopleura_anjunae_NC_030274: 0.010647): 0.000005): 0.002253, Phymanthus_crucifer_NC_027614: 0.047006): 0.139723, ((((Bolocera_sp__BZ_2016_KU507297: 0.000000, (Bolocera_tuediae_NC_022470: 0.000000, Liponema_brevicorne_NC_047221: 0.000000): 0.000000): 0.005163, (Entacmaea_quadricolor_NC_049066: 0.070599, Isosicyonis_striata_KR051006: 0.000005): 0.005316): 0.029519, Stichodactyla_haddoni_MW760873: 0.086933): 0.017189, Epiactis_japonica_NC_047217: 0.029761): 0.373329): 0.000005, ((((Alicia_sansibarensis_NC_027610: 0.269048, (Haloclava_producta_NC_047218: 0.018968, Sagartia_ornata_NC_027615: 0.041694): 0.020983): 0.056249, (Diadumene_lineata_NC_045515: 0.253279, Metridium_senile_NC_000933: 0.009065): 0.071164): 0.038128, Paraphelliactis_xishaensis_MT997141: 0.151955): 0.112071, Antholoba_achates_NC_027611: 0.176947): 0.003131): 0.252337, Halcampoides_purpureus_NC_027612: 0.136090): 0.129260, Nematostella_sp__JVK_2006_NC_008164: 0.699122): 0.242218): 1.801890, (((((((Aurelia_aurita_NC_008446: 0.749096, (Aurelia_coerulea_NC_046792: 0.658290, Aurelia_limbata_NC_046691: 0.472970): 0.047731): 0.000057, (Aurelia_sp__3_sensu_Dawson_et_al___2005__LC005413: 0.587191, Aurelia_sp__4_sensu_Dawson_et_al___2005__LC005414: 0.781873): 0.328791): 1.632251, ((Nemopilema_nomurai_NC_035740: 1.288108, Rhopilema_esculentum_NC_035741: 1.001583): 0.000078, Stomolophus_sp__CG_2019_MK157198: 2.199772): 0.259562): 0.000055, Cassiopea_xamachana_NC_016466: 2.058510): 0.240349, (Chrysaora_pacifica_NC_046775: 0.000005, Chrysaora_quinquecirrha_NC_020459: 0.043554): 3.655722): 0.000069, ((((((Blackfordia_virginica_NC_053946: 0.980457, Eutima_sp__BMK_2020_MW066348: 1.754174): 0.000025, Laomedea_flexuosa_JN700945: 0.838241): 0.666805, (Clava_multicornis_NC_016465: 1.199161, (Turritopsis_dohrnii_NC_031213: 2.508696, Turritopsis_lata_NC_056128: 2.192600): 0.117567): 0.220569): 0.000091, Spirocodon_saltatrix_NC_049868: 2.287918): 0.134527, ((Hydra_oligactis_NC_010214: 1.133632, Hydra_sinensis_NC_021406: 0.708604): 0.994141, Nemalecium_lighti_MZ457217: 3.174142): 0.000042): 0.167126, (Craspedacusta_sowerbii_NC_018537: 11.133277, Cubaia_aphrodite_NC_016467: 1.464942): 0.000121): 0.000028): 0.000026, (Haliclystus_antarcticus_NC_030337: 1.190783, OU744455_1_OU744455_1__OU744455_1: 1.442840): 2.590854): 0.000055): 3.841813, Carijoa_riisei_NC_048963: 0.000005): 0.093254, (Sarcophyton_trocheliophorum_MK994517: 0.051082, (((Sinularia_acuta_MW987591: 0.000003, (Sinularia_maxima_MN485891: 0.005550, Sinularia_penghuensis_MW256412: 0.005546): 0.000005): 0.011140, Sinularia_cf__cruciata_GS_2016_NC_034318: 0.000002): 0.016067, (Sinularia_ceramensis_NC_044122: 0.005507, Sinularia_peculiaris_NC_018379: 0.000005): 0.011875): 0.011108): 0.023220): 0.000005, (((((((Antillogorgia_bipinnata_NC_008157: 0.070165, ((((Eugorgia_mutabilis_NC_035665: 0.016330, Leptogorgia_alba_NC_035669: 0.016494): 0.000005, (Leptogorgia_hebes_MN052675: 0.021849, Leptogorgia_virgulata_MK301586: 0.016546): 0.005499): 0.005512, Leptogorgia_sp__USNM_1437444_KY559412: 0.021846): 0.011983, Pacifigorgia_cairnsi_NC_035668: 0.038730): 0.005505): 0.000005, (((Leptogorgia_capverdensis_NC_035663: 0.000005, Leptogorgia_sarmentosa_NC_035670: 0.000002): 0.045600, Leptogorgia_gaini_KY559404: 0.016527): 0.035139, Leptogorgia_cf__palma_AP_2017_KY559406: 0.015751): 0.000005): 0.016030, (Eunicella_albicans_NC_035666: 0.005447, (Eunicella_cavolini_NC_035667: 0.000005, Eunicella_verrucosa_MW588805: 0.000005): 0.016747): 0.011449): 0.002307, (Calicogorgia_granulosa_NC_023345: 0.540098, Euplexaura_crassa_NC_020458: 0.016229): 0.020536): 0.016996, Muricea_crassa_NC_029697: 0.037168): 0.007863, ((((((Dendronephthya_castanea_NC_023343: 0.000005, Dendronephthya_mollis_NC_020456: 0.000005): 0.000005, Dendronephthya_gigantea_NC_013573: 0.000005): 0.005377, (Dendronephthya_putteri_NC_036022: 0.000005, Dendronephthya_suensoni_NC_022809: 0.000005): 0.000005): 0.062835, Scleronephthya_gracillimum_NC_023344: 0.022600): 0.016555, (Echinogorgia_complexa_NC_020457: 0.064477, Paramuricea_clavata_NC_034749: 0.062766): 0.096550): 0.000005, Trachythela_sp__YZ_2021_MW238423: 0.044080): 0.005643): 0.005732, (Incrustatus_comauensis_MT254531: 0.027811, Telestula_humilis_MT254527: 0.000005): 0.016104): 0.005688): 0.103723, Briareum_asbestinum_NC_008073: 0.075329): 0.008491, (Telestula_cf__batoni_AP_2020_MT254530: 0.027505, Telestula_septentrionalis_MT254532: 0.000005): 0.015703): 0.008803, (((Anthomastus_sp__USNM_1081145_KM015353: 0.005486, Anthomastus_sp__USNM_1171062_KM015352: 0.000003): 0.020965, (((Corallium_japonicum_AB595189: 0.011799, Corallium_rubrum_NC_022864: 0.009813): 0.062600, (Pleurocorallium_elatius_NC_022804: 0.000005, Pleurocorallium_konojoi_NC_015406: 0.000005): 0.069251): 0.010582, Sibogagorgia_cauliflora_NC_026193: 0.199607): 0.025209): 0.000000, Paraminabea_aldersladei_NC_018790: 0.064011): 0.080705): 0.021354, (((((((Anthoptilum_grandiflorum_NC_044086: 0.005380, Anthoptilum_sp__1_RH_2019_MK919656: 0.000002): 0.000005, (Umbellula_huxleyi_MK919668: 0.000005, Umbellula_sp__1_RH_2019_MK919669: 0.038464): 0.021783): 0.005508, Virgularia_mirabilis_NC_044091: 0.097833): 0.005246, (Funiculina_quadrangularis_NC_044078: 0.005357, ((Kophobelemnon_sp__1_RH_2019_MK919660: 0.005404, Kophobelemnon_sp__3_RH_2019_MK919661: 0.005288): 0.023069, Kophobelemnon_sp__4_RH_2019_MK919662: 0.034269): 0.004358): 0.000005): 0.000005, (Halipteris_cf__finmarchica_RH_2019_MK919659: 0.000007, Umbellula_sp__2_RH_2019_MK919670: 0.016014): 0.005342): 0.000005, ((((Distichoptilum_gracile_NC_044077: 0.021656, (Pennatula_cf__inflata_RH_2019_MK919666: 0.000005, Pennatula_grandis_NC_044088: 0.005383): 0.016343): 0.000005, ((Pennatula_aculeata_NC_044087: 0.010746, Pennatula_cf__aculeata_RH_2019_MK919664: 0.000005): 0.019682, Renilla_muelleri_NC_018378: 0.054394): 0.060924): 0.010810, Protoptilum_carpenteri_NC_044089: 0.005379): 0.000005, Stylatula_elongata_NC_018380: 0.044029): 0.016198): 0.007643, Junceella_fragilis_NC_024181: 0.122076): 0.007482): 0.010849, Heliopora_coerulea_NC_020375: 0.054448): 0.006601, ((Callogorgia_cf__gracilis_EE_2018_MH719202: 0.021595, (Plumarella_adhaerans_NC_046480: 0.000005, Plumarella_spinosa_NC_046465: 0.000005): 0.005305): 0.000005, Narella_hawaiiensis_NC_026192: 0.016042): 0.000002): 0.021487, Keratoisidinae_sp__BAL208_1_NC_010764: 0.000005);

**dN tree:**

(Acanella_arbuscula_NC_011016: 0.000002, ((((((((((((((((((((((((((((((Acropora_aculeus_KT001202: 0.000002, (Acropora_horrida_NC_022825: 0.000002, LC201842_1_LC201842_1__LC201842_1: 0.000001): 0.000002): 0.000002, LC201815_1_LC201815_1__LC201815_1: 0.000002): 0.000002, ((((Acropora_aspera_NC_022827: 0.000002, Acropora_muricata_NC_022824: 0.000002): 0.000002, LC201828_1_LC201828_1__LC201828_1: 0.000002): 0.000002, (((Acropora_digitifera_NC_022830: 0.000002, LC201849_1_LC201849_1__LC201849_1: 0.000002): 0.000002, Acropora_florida_NC_022828: 0.000002): 0.000002, Acropora_humilis_NC_022823: 0.000002): 0.000002): 0.000002, (Acropora_hyacinthus_NC_022826: 0.000002, LC201817_1_LC201817_1__LC201817_1: 0.000002): 0.000002): 0.000002): 0.000002, (Acropora_divaricata_NC_022832: 0.000002, (((Acropora_nasuta_NC_022831: 0.000002, Acropora_valida_NC_042742: 0.000002): 0.000002, LC201847_1_LC201847_1__LC201847_1: 0.000002): 0.000001, LC201820_1_LC201820_1__LC201820_1: 0.000002): 0.000002): 0.000002): 0.000002, Acropora_robusta_NC_022833: 0.000002): 0.001710, LC201813_1_LC201813_1__LC201813_1: 0.000002): 0.000000, ((Acropora_tenuis_NC_003522: 0.000002, Acropora_yongei_NC_022829: 0.000002): 0.000002, LC201816_1_LC201816_1__LC201816_1: 0.000002): 0.000001): 0.000002, (Isopora_palifera_NC_024091: 0.000000, Isopora_togianensis_NC_024089: 0.000001): 0.000002): 0.001705, ((Anacropora_matthai_NC_006898: 0.001722, Montipora_efflorescens_NC_040137: 0.000001): 0.000002, (Montipora_aequituberculata_NC_037359: 0.001731, Montipora_cactus_NC_006902: 0.000002): 0.000001): 0.005156): 0.000001, (Alveopora_japonica_NC_040136: 0.000000, Alveopora_sp__MFL_2014_KJ634271: 0.000001): 0.008610): 0.000000, (Astreopora_explanata_NC_024090: 0.000000, Astreopora_myriophthalma_NC_024092: 0.000000): 0.001702): 0.006859, ((Agaricia_humilis_NC_008160: 0.001736, Pavona_decussata_NC_026527: 0.001728): 0.000015, (Fimbriaphyllia_ancora_NC_015641: 0.000000, Galaxea_fascicularis_NC_029696: 0.001705): 0.012052): 0.001712): 0.008683, ((Pseudosiderastrea_formosa_NC_026530: 0.001695, Pseudosiderastrea_tayamai_NC_026531: 0.000001): 0.001683, Siderastrea_radians_NC_008167: 0.003485): 0.000005): 0.000004, ((((((Dendrophyllia_arbuscula_NC_027590: 0.000001, Tubastraea_coccinea_NC_026025: 0.000000): 0.000000, ((Tubastraea_diaphana_MK959042: 0.000000, Tubastraea_tagusensis_NC_030352: 0.001683): 0.000000, Tubastraea_micranthus_MK959041: 0.000000): 0.001679): 0.001681, Dendrophyllia_cribrosa_NC_026026: 0.003372): 0.001687, Turbinaria_peltata_NC_024671: 0.000002): 0.008238, (((Goniopora_columna_NC_015643: 0.000001, Goniopora_djiboutiensis_NC_045931: 0.000000): 0.000003, Goniopora_lobata_MN795054: 0.000002): 0.001653, ((Porites_fontanesii_NC_037434: 0.000001, ((Porites_panamensis_NC_024182: 0.000001, Porites_sverdrupi_KU956960: 0.000000): 0.000001, Porites_porites_NC_008166: 0.000001): 0.000002): 0.000000, (Porites_harrisoni_NC_037435: 0.000001, ((Porites_lobata_NC_030186: 0.000000, Porites_okinawensis_NC_015644: 0.000000): 0.000001, (Porites_lutea_NC_029695: 0.000001, Porites_rus_NC_027526: 0.000001): 0.000000): 0.000000): 0.000001): 0.000005): 0.008718): 0.003441, Fungiacyathus_stephanus_NC_015640: 0.015264): 0.001940): 0.012267, Gardineria_hawaiiensis_NC_056290: 0.022229): 0.002886, (((((Amplexidiscus_fenestrafer_NC_027101: 0.000001, (Discosoma_sp__MH308003: 0.001733, (Rhodactis_indosinensis_NC_027103: 0.000001, Rhodactis_sp__CASIZ_171755_NC_008158: 0.000002): 0.000002): 0.001730): 0.003476, (Discosoma_nummiforme_NC_027100: 0.000001, ((Discosoma_sp__CASIZ_168915_NC_008071: 0.000002, Discosoma_sp__CASIZ_168916_NC_008072: 0.000002): 0.000002, Platyzoanthus_mussoides_NC_027104: 0.003446): 0.000001): 0.003474): 0.003462, (Pseudocorynactis_sp__SIO_KP938437: 0.008718, (Ricordea_florida_NC_008159: 0.023278, Ricordea_yuma_NC_027106: 0.003469): 0.003363): 0.000003): 0.000005, Corynactis_californica_NC_027102: 0.008790): 0.002934, Corallimorphus_profundus_KP938440: 0.009389): 0.010823): 0.020233, ((((((Astrangia_sp__JVK_2006_NC_008161: 0.003222, (((Colpophyllia_natans_NC_008162: 0.000001, Mussa_angulosa_NC_008163: 0.000001): 0.000003, ((((((Dipsastraea_favus_NC_046690: 0.000000, Dipsastraea_rotumana_NC_044074: 0.001545): 0.000001, Favites_abdita_NC_035879: 0.000001): 0.000000, Platygyra_carnosa_NC_020049: 0.000001): 0.000000, (Favites_pentagona_NC_034916: 0.000001, Hydnophora_exesa_NC_042795: 0.000001): 0.000000): 0.000002, ((Orbicella_annularis_NC_007224: 0.000000, Orbicella_faveolata_NC_007226: 0.000000): 0.000000, Orbicella_franksi_NC_007225: 0.000001): 0.001547): 0.001540, Echinophyllia_aspera_NC_040169: 0.001555): 0.000001): 0.001533, Plesiastrea_versipora_NC_042481: 0.001517): 0.004375): 0.002152, (Polycyathus_sp__MFL_2011_NC_015642: 0.000000, Psammocora_nierstraszi_MT576637: 0.000002): 0.013222): 0.021144, Madrepora_oculata_NC_018364: 0.030280): 0.025136, (Madracis_myriaster_NC_011160: 0.001902, ((Pocillopora_damicornis_NC_009797: 0.000000, Pocillopora_grandis_NC_009798: 0.000000): 0.000004, ((Seriatopora_caliendrum_NC_010245: 0.000001, Seriatopora_hystrix_NC_010244: 0.000001): 0.003025, Stylophora_pistillata_NC_011162: 0.000002): 0.000001): 0.005844): 0.044466): 0.005163, ((Desmophyllum_dianthus_NC_034275: 0.000001, Desmophyllum_pertusum_NC_015143: 0.000000): 0.000001, Solenosmilia_variabilis_NC_025472: 0.000000): 0.037019): 0.040148, Paraconotrochus_antarcticus_NC_056275: 0.105328): 0.032893): 0.029352, ((((((Antipathes_cf__dichotoma_NB_2020_MT318841: 0.000002, Stichopathes_abyssicola_MT318856: 0.000002): 0.010579, (Phanopathes_sp__NB_2020_MT318852: 0.007383, Stichopathes_sp__n__NB_2020_MT318857: 0.005064): 0.000005): 0.002054, ((Stichopathes_luetkeni_NC_018377: 0.000000, Stichopathes_sp__SCBUCN_8850_MZ157399: 0.000001): 0.000000, Stichopathes_sp__SCBUCN_8849_MZ157400: 0.000001): 0.006863): 0.001715, ((Myriopathes_japonica_NC_027667: 0.000002, Tanacetipathes_thamnea_NC_046843: 0.000001): 0.000001, Tylopathes_sp__n__NB_2020_MT318859: 0.001765): 0.003528): 0.000002, (((((((Bathypathes_sp__1_NB_2020_MT318844: 0.000002, Bathypathes_sp__n__2_NB_2020_MT318842: 0.000002): 0.000002, Stauropathes_arctica_MT318854: 0.000002): 0.000002, Bathypathes_sp__n__3_NB_2020_MT318843: 0.000002): 0.000002, Stauropathes_cf__punctata_NB_2020_MT318855: 0.000002): 0.000000, Telopathes_sp__NB_2020_MT318858: 0.003527): 0.000002, (Dendrobathypathes_sp__n__NB_2020_MT318845: 0.007120, ((Parantipathes_cf__hirondelle_NB_2020_MT318849: 0.000002, Parantipathes_hirondelle_MT318850: 0.000002): 0.000002, (Parantipathes_sp__NB_2020_MT318851: 0.000002, Sibopathes_cf__macrospina_NB_2020_MT318853: 0.000002): 0.000002): 0.000002): 0.000003): 0.000004, (Chrysopathes_formosa_NC_008411: 0.000001, Trissopathes_cf__tetracrada_NB_2020_MT318840: 0.000002): 0.001752): 0.000012): 0.001832, (Leiopathes_cf__glaberrima_NB_2020_MT318846: 0.000002, Leiopathes_expansa_MT318847: 0.000002): 0.001705): 0.009021): 0.005761, (Epizoanthus_illoricatus_NC_046474: 0.005926, (((Hydrozoanthus_gracilis_NC_046400: 0.008595, ((Neozoanthus_aff__uchina_AP_2020_MN873592: 0.001797, ((Zoanthus_cf__pulchellus_AP_2020_MN873599: 0.000001, ((Zoanthus_cf__sociatus_AP_2020_MN873600: 0.000002, Zoanthus_sociatus_NC_046476: 0.000002): 0.000000, Zoanthus_sp__AP_2020_MN873602: 0.000002): 0.000002): 0.000000, Zoanthus_sansibaricus_NC_035578: 0.000000): 0.000003): 0.002306, (Palythoa_heliodiscus_NC_035579: 0.001788, ((Palythoa_mizigama_NC_046403: 0.001823, Palythoa_mutuki_NC_046404: 0.000000): 0.000000, Sphenopus_marsupialis_NC_046406: 0.000001): 0.003665): 0.001326): 0.000002): 0.002269, ((Parazoanthus_elongatus_NC_046405: 0.003628, Parazoanthus_swiftii_NC_046475: 0.000002): 0.000004, Savalia_savaglia_DQ825686: 0.001819): 0.000002): 0.003655, (Microzoanthus_occultus_NC_046401: 0.059973, Nanozoanthus_harenaceus_NC_046402: 0.026284): 0.026182): 0.000000): 0.028734): 0.007631, (((((((Actinia_equina_NC_039929: 0.000000, Actinia_tenebrosa_NC_044902: 0.000000): 0.003411, (((Anemonia_manjano_NC_037178: 0.003433, Heteractis_aurora_NC_047219: 0.000000): 0.000003, (Anemonia_sulcata_NC_049065: 0.000000, Anemonia_viridis_NC_037177: 0.000000): 0.000002): 0.000000, Anthopleura_anjunae_NC_030274: 0.000001): 0.000000): 0.000000, Phymanthus_crucifer_NC_027614: 0.003393): 0.000014, ((((Bolocera_sp__BZ_2016_KU507297: 0.000002, (Bolocera_tuediae_NC_022470: 0.000002, Liponema_brevicorne_NC_047221: 0.000002): 0.000002): 0.000001, (Entacmaea_quadricolor_NC_049066: 0.005232, Isosicyonis_striata_KR051006: 0.000000): 0.000001): 0.000003, Stichodactyla_haddoni_MW760873: 0.001738): 0.000002, Epiactis_japonica_NC_047217: 0.000003): 0.003476): 0.000000, ((((Alicia_sansibarensis_NC_027610: 0.011879, (Haloclava_producta_NC_047218: 0.003384, Sagartia_ornata_NC_027615: 0.000004): 0.001709): 0.003380, (Diadumene_lineata_NC_045515: 0.000025, Metridium_senile_NC_000933: 0.003382): 0.001692): 0.000004, Paraphelliactis_xishaensis_MT997141: 0.000015): 0.001714, Antholoba_achates_NC_027611: 0.001695): 0.000000): 0.003526, Halcampoides_purpureus_NC_027612: 0.009127): 0.012433, Nematostella_sp__JVK_2006_NC_008164: 0.011440): 0.014254): 0.040694, (((((((Aurelia_aurita_NC_008446: 0.019424, (Aurelia_coerulea_NC_046792: 0.022253, Aurelia_limbata_NC_046691: 0.008060): 0.006976): 0.004926, (Aurelia_sp__3_sensu_Dawson_et_al___2005__LC005413: 0.026834, Aurelia_sp__4_sensu_Dawson_et_al___2005__LC005414: 0.013099): 0.018031): 0.061105, ((Nemopilema_nomurai_NC_035740: 0.026435, Rhopilema_esculentum_NC_035741: 0.015976): 0.018670, Stomolophus_sp__CG_2019_MK157198: 0.035034): 0.013955): 0.016652, Cassiopea_xamachana_NC_016466: 0.085035): 0.033841, (Chrysaora_pacifica_NC_046775: 0.000000, Chrysaora_quinquecirrha_NC_020459: 0.001679): 0.071156): 0.069316, ((((((Blackfordia_virginica_NC_053946: 0.023875, Eutima_sp__BMK_2020_MW066348: 0.037609): 0.012845, Laomedea_flexuosa_JN700945: 0.036217): 0.024015, (Clava_multicornis_NC_016465: 0.041841, (Turritopsis_dohrnii_NC_031213: 0.025854, Turritopsis_lata_NC_056128: 0.015595): 0.083471): 0.008678): 0.016727, Spirocodon_saltatrix_NC_049868: 0.050769): 0.017932, ((Hydra_oligactis_NC_010214: 0.044117, Hydra_sinensis_NC_021406: 0.040748): 0.050387, Nemalecium_lighti_MZ457217: 0.193024): 0.024905): 0.047892, (Craspedacusta_sowerbii_NC_018537: 0.071775, Cubaia_aphrodite_NC_016467: 0.033628): 0.048172): 0.014501): 0.020011, (Haliclystus_antarcticus_NC_030337: 0.003623, OU744455_1_OU744455_1__OU744455_1: 0.005211): 0.180032): 0.042089): 0.083948, Carijoa_riisei_NC_048963: 0.000000): 0.003313, (Sarcophyton_trocheliophorum_MK994517: 0.003300, (((Sinularia_acuta_MW987591: 0.003257, (Sinularia_maxima_MN485891: 0.000001, Sinularia_penghuensis_MW256412: 0.000001): 0.000000): 0.001630, Sinularia_cf__cruciata_GS_2016_NC_034318: 0.001633): 0.000002, (Sinularia_ceramensis_NC_044122: 0.000001, Sinularia_peculiaris_NC_018379: 0.000000): 0.004940): 0.000001): 0.000002): 0.000000, (((((((Antillogorgia_bipinnata_NC_008157: 0.000007, ((((Eugorgia_mutabilis_NC_035665: 0.000002, Leptogorgia_alba_NC_035669: 0.000002): 0.000000, (Leptogorgia_hebes_MN052675: 0.000002, Leptogorgia_virgulata_MK301586: 0.001666): 0.000001): 0.001672, Leptogorgia_sp__USNM_1437444_KY559412: 0.001678): 0.001684, Pacifigorgia_cairnsi_NC_035668: 0.001664): 0.001663): 0.000000, (((Leptogorgia_capverdensis_NC_035663: 0.000000, Leptogorgia_sarmentosa_NC_035670: 0.001663): 0.000005, Leptogorgia_gaini_KY559404: 0.003318): 0.000004, Leptogorgia_cf__palma_AP_2017_KY559406: 0.000002): 0.000000): 0.004974, (Eunicella_albicans_NC_035666: 0.000001, (Eunicella_cavolini_NC_035667: 0.000000, Eunicella_verrucosa_MW588805: 0.000000): 0.001644): 0.009978): 0.000000, (Calicogorgia_granulosa_NC_023345: 0.017550, Euplexaura_crassa_NC_020458: 0.000002): 0.000002): 0.000002, Muricea_crassa_NC_029697: 0.003315): 0.000001, ((((((Dendronephthya_castanea_NC_023343: 0.000000, Dendronephthya_mollis_NC_020456: 0.000000): 0.000000, Dendronephthya_gigantea_NC_013573: 0.000000): 0.000001, (Dendronephthya_putteri_NC_036022: 0.000000, Dendronephthya_suensoni_NC_022809: 0.000000): 0.000000): 0.000006, Scleronephthya_gracillimum_NC_023344: 0.000002): 0.005730, (Echinogorgia_complexa_NC_020457: 0.001650, Paramuricea_clavata_NC_034749: 0.000006): 0.002590): 0.000000, Trachythela_sp__YZ_2021_MW238423: 0.003898): 0.002807): 0.000001, (Incrustatus_comauensis_MT254531: 0.000003, Telestula_humilis_MT254527: 0.000000): 0.003319): 0.000001): 0.001668, Briareum_asbestinum_NC_008073: 0.001687): 0.001689, (Telestula_cf__batoni_AP_2020_MT254530: 0.000003, Telestula_septentrionalis_MT254532: 0.000000): 0.001677): 0.000001, (((Anthomastus_sp__USNM_1081145_KM015353: 0.001662, Anthomastus_sp__USNM_1171062_KM015352: 0.003321): 0.000002, (((Corallium_japonicum_AB595189: 0.000001, Corallium_rubrum_NC_022864: 0.000001): 0.001697, (Pleurocorallium_elatius_NC_022804: 0.000000, Pleurocorallium_konojoi_NC_015406: 0.000000): 0.003424): 0.000001, Sibogagorgia_cauliflora_NC_026193: 0.088567): 0.003353): 0.000002, Paraminabea_aldersladei_NC_018790: 0.005035): 0.005062): 0.000002, (((((((Anthoptilum_grandiflorum_NC_044086: 0.000001, Anthoptilum_sp__1_RH_2019_MK919656: 0.001685): 0.000000, (Umbellula_huxleyi_MK919668: 0.000000, Umbellula_sp__1_RH_2019_MK919669: 0.003404): 0.005145): 0.000001, Virgularia_mirabilis_NC_044091: 0.000010): 0.000001, (Funiculina_quadrangularis_NC_044078: 0.003395, ((Kophobelemnon_sp__1_RH_2019_MK919660: 0.000001, Kophobelemnon_sp__3_RH_2019_MK919661: 0.001710): 0.001698, Kophobelemnon_sp__4_RH_2019_MK919662: 0.001689): 0.000000): 0.000000): 0.000000, (Halipteris_cf__finmarchica_RH_2019_MK919659: 0.006848, Umbellula_sp__2_RH_2019_MK919670: 0.000002): 0.005115): 0.000000, ((((Distichoptilum_gracile_NC_044077: 0.000002, (Pennatula_cf__inflata_RH_2019_MK919666: 0.000000, Pennatula_grandis_NC_044088: 0.000001): 0.001692): 0.000000, ((Pennatula_aculeata_NC_044087: 0.000001, Pennatula_cf__aculeata_RH_2019_MK919664: 0.000000): 0.002794, Renilla_muelleri_NC_018378: 0.006036): 0.017205): 0.000001, Protoptilum_carpenteri_NC_044089: 0.001690): 0.000000, Stylatula_elongata_NC_018380: 0.010263): 0.000002): 0.005111, Junceella_fragilis_NC_024181: 0.001681): 0.005121): 0.001683, Heliopora_coerulea_NC_020375: 0.005120): 0.005121, ((Callogorgia_cf__gracilis_EE_2018_MH719202: 0.001686, (Plumarella_adhaerans_NC_046480: 0.000000, Plumarella_spinosa_NC_046465: 0.000000): 0.000001): 0.000000, Narella_hawaiiensis_NC_026192: 0.000002): 0.001698): 0.003396, Keratoisidinae_sp__BAL208_1_NC_010764: 0.000000);

**w ratios as labels for TreeView:**

(Acanella_arbuscula_NC_011016 #0.0001 , ((((((((((((((((((((((((((((((Acropora_aculeus_KT001202 #14.401 , (Acropora_horrida_NC_022825 #14.4856 , LC201842_1_LC201842_1__LC201842_1 #0.0001 ) #15.4665 ) #14.8422 , LC201815_1_LC201815_1__LC201815_1 #14.4886 ) #14.5743 , ((((Acropora_aspera_NC_022827 #14.8519 , Acropora_muricata_NC_022824 #14.7136 ) #14.8863 , LC201828_1_LC201828_1__LC201828_1 #14.6563 ) #14.8724 , (((Acropora_digitifera_NC_022830 #15.7686 , LC201849_1_LC201849_1__LC201849_1 #14.5767 ) #15.8075 , Acropora_florida_NC_022828 #15.6069 ) #14.8115 , Acropora_humilis_NC_022823 #16.5363 ) #12.746 ) #15.0043 , (Acropora_hyacinthus_NC_022826 #14.5685 , LC201817_1_LC201817_1__LC201817_1 #16.7305 ) #202.389 ) #14.6192 ) #14.4704 , (Acropora_divaricata_NC_022832 #14.6515 , (((Acropora_nasuta_NC_022831 #26.2345 , Acropora_valida_NC_042742 #7.99015 ) #26.0298 , LC201847_1_LC201847_1__LC201847_1 #5.59529 ) #0.0001 , LC201820_1_LC201820_1__LC201820_1 #5.71773 ) #14.7953 ) #14.6473 ) #17.1438 , Acropora_robusta_NC_022833 #5.47731 ) #999 , LC201813_1_LC201813_1__LC201813_1 #21.7679 ) #0.0001 , ((Acropora_tenuis_NC_003522 #24.8976 , Acropora_yongei_NC_022829 #20.9229 ) #20.9744 , LC201816_1_LC201816_1__LC201816_1 #24.7516 ) #0.0001 ) #0.0001 , (Isopora_palifera_NC_024091 #0.0001 , Isopora_togianensis_NC_024089 #0.0001 ) #0.0001 ) #0.0236528 , ((Anacropora_matthai_NC_006898 #999 , Montipora_efflorescens_NC_040137 #0.0001 ) #22.3533 , (Montipora_aequituberculata_NC_037359 #0.113325 , Montipora_cactus_NC_006902 #25.7198 ) #0.0001 ) #0.0803985 ) #0.0001 , (Alveopora_japonica_NC_040136 #0.0001 , Alveopora_sp__MFL_2014_KJ634271 #0.0001 ) #0.0660268 ) #0.0001 , (Astreopora_explanata_NC_024090 #0.0001 , Astreopora_myriophthalma_NC_024092 #0.000100003 ) #0.106922 ) #0.263964 , ((Agaricia_humilis_NC_008160 #0.11469 , Pavona_decussata_NC_026527 #965.9 ) #0.0001 , (Fimbriaphyllia_ancora_NC_015641 #0.0001 , Galaxea_fascicularis_NC_029696 #0.0820088 ) #0.16815 ) #0.183111 ) #0.320727 , ((Pseudosiderastrea_formosa_NC_026530 #0.120383 , Pseudosiderastrea_tayamai_NC_026531 #0.0001 ) #0.026068 , Siderastrea_radians_NC_008167 #0.0816344 ) #0.0001 ) #0.0001 , ((((((Dendrophyllia_arbuscula_NC_027590 #0.0001 , Tubastraea_coccinea_NC_026025 #0.0001 ) #0.0001 , ((Tubastraea_diaphana_MK959042 #0.0001 , Tubastraea_tagusensis_NC_030352 #999 ) #0.0001 , Tubastraea_micranthus_MK959041 #0.0001 ) #999 ) #0.316985 , Dendrophyllia_cribrosa_NC_026026 #0.631034 ) #0.110278 , Turbinaria_peltata_NC_024671 #0.0001 ) #2.01722 , (((Goniopora_columna_NC_015643 #0.0001 , Goniopora_djiboutiensis_NC_045931 #0.0001 ) #0.0001 , Goniopora_lobata_MN795054 #0.0001 ) #0.0236902 , ((Porites_fontanesii_NC_037434 #0.0001 , ((Porites_panamensis_NC_024182 #0.0001 , Porites_sverdrupi_KU956960 #0.0001 ) #0.0001 , Porites_porites_NC_008166 #0.0001 ) #0.0001 ) #0.0001 , (Porites_harrisoni_NC_037435 #0.0001 , ((Porites_lobata_NC_030186 #0.0001 , Porites_okinawensis_NC_015644 #0.0001 ) #0.0001 , (Porites_lutea_NC_029695 #0.0001 , Porites_rus_NC_027526 #0.0001 ) #0.0001 ) #0.0001 ) #0.0001 ) #0.0001 ) #0.160165 ) #0.12464 , Fungiacyathus_stephanus_NC_015640 #0.320749 ) #0.0405494 ) #0.0581702 , Gardineria_hawaiiensis_NC_056290 #0.114422 ) #0.0381236 , (((((Amplexidiscus_fenestrafer_NC_027101 #0.0001 , (Discosoma_sp__MH308003 #999 , (Rhodactis_indosinensis_NC_027103 #0.0001 , Rhodactis_sp__CASIZ_171755_NC_008158 #32.9689 ) #30.5149 ) #999 ) #0.168405 , (Discosoma_nummiforme_NC_027100 #0.0001 , ((Discosoma_sp__CASIZ_168915_NC_008071 #26.0719 , Discosoma_sp__CASIZ_168916_NC_008072 #26.2627 ) #11.4223 , Platyzoanthus_mussoides_NC_027104 #0.672063 ) #0.0001 ) #0.655444 ) #0.197224 , (Pseudocorynactis_sp__SIO_KP938437 #0.0565931 , (Ricordea_florida_NC_008159 #1.53677 , Ricordea_yuma_NC_027106 #0.0790477 ) #0.0468287 ) #0.0001 ) #0.0001 , Corynactis_californica_NC_027102 #0.112507 ) #0.0568935 , Corallimorphus_profundus_KP938440 #0.081029 ) #0.128001 ) #0.0867243 , ((((((Astrangia_sp__JVK_2006_NC_008161 #0.057019 , (((Colpophyllia_natans_NC_008162 #0.0001 , Mussa_angulosa_NC_008163 #0.0001 ) #0.0001 , ((((((Dipsastraea_favus_NC_046690 #0.0001 , Dipsastraea_rotumana_NC_044074 #0.0642697 ) #0.0001 , Favites_abdita_NC_035879 #0.0001 ) #0.0001 , Platygyra_carnosa_NC_020049 #0.0001 ) #0.0001 , (Favites_pentagona_NC_034916 #0.0001 , Hydnophora_exesa_NC_042795 #0.0001 ) #0.0001 ) #0.0001 , ((Orbicella_annularis_NC_007224 #0.0001 , Orbicella_faveolata_NC_007226 #0.0001 ) #0.0001 , Orbicella_franksi_NC_007225 #0.0001 ) #0.0306426 ) #0.0365373 , Echinophyllia_aspera_NC_040169 #0.040536 ) #0.0001 ) #0.0146227 , Plesiastrea_versipora_NC_042481 #0.0289608 ) #0.0476803 ) #0.0295129 , (Polycyathus_sp__MFL_2011_NC_015642 #0.0001 , Psammocora_nierstraszi_MT576637 #0.0001 ) #0.0383203 ) #0.115489 , Madrepora_oculata_NC_018364 #0.0687059 ) #0.172729 , (Madracis_myriaster_NC_011160 #0.0256176 , ((Pocillopora_damicornis_NC_009797 #0.0001 , Pocillopora_grandis_NC_009798 #0.0001 ) #0.0001 , ((Seriatopora_caliendrum_NC_010245 #0.0001 , Seriatopora_hystrix_NC_010244 #0.0001 ) #0.140119 , Stylophora_pistillata_NC_011162 #0.0001 ) #0.0001 ) #0.16106 ) #0.0943169 ) #0.0897382 , ((Desmophyllum_dianthus_NC_034275 #0.0001 , Desmophyllum_pertusum_NC_015143 #0.0001 ) #0.0001 , Solenosmilia_variabilis_NC_025472 #0.0001 ) #0.0704669 ) #0.115754 , Paraconotrochus_antarcticus_NC_056275 #0.0593365 ) #0.0409428 ) #0.295945 , ((((((Antipathes_cf__dichotoma_NB_2020_MT318841 #47.2508 , Stichopathes_abyssicola_MT318856 #47.8758 ) #0.0730816 , (Phanopathes_sp__NB_2020_MT318852 #0.057859 , Stichopathes_sp__n__NB_2020_MT318857 #0.0406883 ) #0.0001 ) #0.0215245 , ((Stichopathes_luetkeni_NC_018377 #0.0001 , Stichopathes_sp__SCBUCN_8850_MZ157399 #0.0001 ) #0.0001 , Stichopathes_sp__SCBUCN_8849_MZ157400 #0.0001 ) #0.0259454 ) #0.0525848 , ((Myriopathes_japonica_NC_027667 #0.0001 , Tanacetipathes_thamnea_NC_046843 #0.0001 ) #0.0001 , Tylopathes_sp__n__NB_2020_MT318859 #0.0367244 ) #0.0229239 ) #0.0001 , (((((((Bathypathes_sp__1_NB_2020_MT318844 #35.3767 , Bathypathes_sp__n__2_NB_2020_MT318842 #35.2981 ) #35.4168 , Stauropathes_arctica_MT318854 #34.9017 ) #30.947 , Bathypathes_sp__n__3_NB_2020_MT318843 #35.2768 ) #0.0001 , Stauropathes_cf__punctata_NB_2020_MT318855 #0.0001 ) #0.0001 , Telopathes_sp__NB_2020_MT318858 #999 ) #0.0001 , (Dendrobathypathes_sp__n__NB_2020_MT318845 #0.189015 , ((Parantipathes_cf__hirondelle_NB_2020_MT318849 #32.3238 , Parantipathes_hirondelle_MT318850 #34.8332 ) #31.6741 , (Parantipathes_sp__NB_2020_MT318851 #31.7348 , Sibopathes_cf__macrospina_NB_2020_MT318853 #34.7184 ) #34.7845 ) #0.0001 ) #0.0001 ) #0.0001 , (Chrysopathes_formosa_NC_008411 #0.0001 , Trissopathes_cf__tetracrada_NB_2020_MT318840 #0.0001 ) #0.0506108 ) #0.0001 ) #0.114483 , (Leiopathes_cf__glaberrima_NB_2020_MT318846 #40.1134 , Leiopathes_expansa_MT318847 #39.5983 ) #0.0232484 ) #0.0273191 ) #0.0420794 , (Epizoanthus_illoricatus_NC_046474 #0.0628128 , (((Hydrozoanthus_gracilis_NC_046400 #0.109974 , ((Neozoanthus_aff__uchina_AP_2020_MN873592 #0.0689928 , ((Zoanthus_cf__pulchellus_AP_2020_MN873599 #0.0001 , ((Zoanthus_cf__sociatus_AP_2020_MN873600 #48.2302 , Zoanthus_sociatus_NC_046476 #47.9817 ) #0.0001 , Zoanthus_sp__AP_2020_MN873602 #47.8373 ) #48.8398 ) #0.0001 , Zoanthus_sansibaricus_NC_035578 #0.0001 ) #0.0001 ) #0.0996867 , (Palythoa_heliodiscus_NC_035579 #0.11734 , ((Palythoa_mizigama_NC_046403 #999 , Palythoa_mutuki_NC_046404 #0.0001 ) #0.0001 , Sphenopus_marsupialis_NC_046406 #0.0001 ) #0.3642 ) #0.184605 ) #0.0001 ) #0.0665838 , ((Parazoanthus_elongatus_NC_046405 #0.359162 , Parazoanthus_swiftii_NC_046475 #21.1568 ) #0.0001 , Savalia_savaglia_DQ825686 #0.0520312 ) #48.6205 ) #0.0862153 , (Microzoanthus_occultus_NC_046401 #0.0717498 , Nanozoanthus_harenaceus_NC_046402 #0.0455997 ) #0.0143358 ) #0.0001 ) #0.063577 ) #0.0336335 , (((((((Actinia_equina_NC_039929 #0.0001 , Actinia_tenebrosa_NC_044902 #0.0001 ) #0.643664 , (((Anemonia_manjano_NC_037178 #0.128171 , Heteractis_aurora_NC_047219 #0.0001 ) #0.0001 , (Anemonia_sulcata_NC_049065 #0.0001 , Anemonia_viridis_NC_037177 #0.0001 ) #0.0001 ) #0.0001 , Anthopleura_anjunae_NC_030274 #0.0001 ) #0.0001 ) #0.0001 , Phymanthus_crucifer_NC_027614 #0.0721732 ) #0.0001 , ((((Bolocera_sp__BZ_2016_KU507297 #17.1369 , (Bolocera_tuediae_NC_022470 #16.2246 , Liponema_brevicorne_NC_047221 #10.3371 ) #12.0229 ) #0.0001 , (Entacmaea_quadricolor_NC_049066 #0.0741156 , Isosicyonis_striata_KR051006 #0.0001 ) #0.0001 ) #0.0001 , Stichodactyla_haddoni_MW760873 #0.0199951 ) #0.0001 , Epiactis_japonica_NC_047217 #0.0001 ) #0.00930998 ) #0.0001 , ((((Alicia_sansibarensis_NC_027610 #0.0441511 , (Haloclava_producta_NC_047218 #0.178424 , Sagartia_ornata_NC_027615 #0.0001 ) #0.08147 ) #0.0600835 , (Diadumene_lineata_NC_045515 #0.0001 , Metridium_senile_NC_000933 #0.373068 ) #0.0237785 ) #0.0001 , Paraphelliactis_xishaensis_MT997141 #0.0001 ) #0.0152982 , Antholoba_achates_NC_027611 #0.0095813 ) #0.0001 ) #0.0139729 , Halcampoides_purpureus_NC_027612 #0.0670646 ) #0.0961896 , Nematostella_sp__JVK_2006_NC_008164 #0.016364 ) #0.0588466 ) #0.0225839 , (((((((Aurelia_aurita_NC_008446 #0.0259299 , (Aurelia_coerulea_NC_046792 #0.033804 , Aurelia_limbata_NC_046691 #0.0170417 ) #0.146156 ) #85.8745 , (Aurelia_sp__3_sensu_Dawson_et_al___2005__LC005413 #0.0456986 , Aurelia_sp__4_sensu_Dawson_et_al___2005__LC005414 #0.0167539 ) #0.0548389 ) #0.0374362 , ((Nemopilema_nomurai_NC_035740 #0.0205221 , Rhopilema_esculentum_NC_035741 #0.0159505 ) #239.958 , Stomolophus_sp__CG_2019_MK157198 #0.0159262 ) #0.0537656 ) #301.076 , Cassiopea_xamachana_NC_016466 #0.0413091 ) #0.140798 , (Chrysaora_pacifica_NC_046775 #0.0001 , Chrysaora_quinquecirrha_NC_020459 #0.038548 ) #0.0194644 ) #999 , ((((((Blackfordia_virginica_NC_053946 #0.0243507 , Eutima_sp__BMK_2020_MW066348 #0.0214397 ) #513.137 , Laomedea_flexuosa_JN700945 #0.0432065 ) #0.0360154 , (Clava_multicornis_NC_016465 #0.0348916 , (Turritopsis_dohrnii_NC_031213 #0.0103058 , Turritopsis_lata_NC_056128 #0.00711273 ) #0.709985 ) #0.0393429 ) #182.863 , Spirocodon_saltatrix_NC_049868 #0.0221901 ) #0.133296 , ((Hydra_oligactis_NC_010214 #0.0389165 , Hydra_sinensis_NC_021406 #0.0575048 ) #0.0506837 , Nemalecium_lighti_MZ457217 #0.0608114 ) #594.14 ) #0.286563 , (Craspedacusta_sowerbii_NC_018537 #0.0064469 , Cubaia_aphrodite_NC_016467 #0.0229552 ) #396.632 ) #514.733 ) #779.312 , (Haliclystus_antarcticus_NC_030337 #0.00304275 , OU744455_1_OU744455_1__OU744455_1 #0.00361165 ) #0.0694875 ) #771.581 ) #0.0218512 , Carijoa_riisei_NC_048963 #0.0001 ) #0.0355258 , (Sarcophyton_trocheliophorum_MK994517 #0.0646114 , (((Sinularia_acuta_MW987591 #999 , (Sinularia_maxima_MN485891 #0.0001 , Sinularia_penghuensis_MW256412 #0.0001 ) #0.0001 ) #0.146341 , Sinularia_cf__cruciata_GS_2016_NC_034318 #930.408 ) #0.0001 , (Sinularia_ceramensis_NC_044122 #0.0001 , Sinularia_peculiaris_NC_018379 #0.0001 ) #0.41601 ) #0.0001 ) #0.0001 ) #0.0001 , (((((((Antillogorgia_bipinnata_NC_008157 #0.0001 , ((((Eugorgia_mutabilis_NC_035665 #0.0001 , Leptogorgia_alba_NC_035669 #0.0001 ) #0.0001 , (Leptogorgia_hebes_MN052675 #0.0001 , Leptogorgia_virgulata_MK301586 #0.100681 ) #0.0001 ) #0.303398 , Leptogorgia_sp__USNM_1437444_KY559412 #0.0768315 ) #0.140527 , Pacifigorgia_cairnsi_NC_035668 #0.0429635 ) #0.302178 ) #0.0001 , (((Leptogorgia_capverdensis_NC_035663 #0.0001 , Leptogorgia_sarmentosa_NC_035670 #989.746 ) #0.0001 , Leptogorgia_gaini_KY559404 #0.200757 ) #0.0001 , Leptogorgia_cf__palma_AP_2017_KY559406 #0.0001 ) #0.0001 ) #0.310309 , (Eunicella_albicans_NC_035666 #0.0001 , (Eunicella_cavolini_NC_035667 #0.0001 , Eunicella_verrucosa_MW588805 #0.0001 ) #0.098172 ) #0.87147 ) #0.0001 , (Calicogorgia_granulosa_NC_023345 #0.0324935 , Euplexaura_crassa_NC_020458 #0.0001 ) #0.0001 ) #0.0001 , Muricea_crassa_NC_029697 #0.0891902 ) #0.0001 , ((((((Dendronephthya_castanea_NC_023343 #0.0001 , Dendronephthya_mollis_NC_020456 #0.0001 ) #0.0001 , Dendronephthya_gigantea_NC_013573 #0.0001 ) #0.0001 , (Dendronephthya_putteri_NC_036022 #0.0001 , Dendronephthya_suensoni_NC_022809 #0.0001 ) #0.0001 ) #0.0001 , Scleronephthya_gracillimum_NC_023344 #0.0001 ) #0.346145 , (Echinogorgia_complexa_NC_020457 #0.0255941 , Paramuricea_clavata_NC_034749 #0.0001 ) #0.0268226 ) #0.0001 , Trachythela_sp__YZ_2021_MW238423 #0.0884413 ) #0.497487 ) #0.0001 , (Incrustatus_comauensis_MT254531 #0.0001 , Telestula_humilis_MT254527 #0.0001 ) #0.206114 ) #0.0001 ) #0.016086 , Briareum_asbestinum_NC_008073 #0.0223892 ) #0.198974 , (Telestula_cf__batoni_AP_2020_MT254530 #0.0001 , Telestula_septentrionalis_MT254532 #0.0001 ) #0.106821 ) #0.0001 , (((Anthomastus_sp__USNM_1081145_KM015353 #0.302998 , Anthomastus_sp__USNM_1171062_KM015352 #999 ) #0.0001 , (((Corallium_japonicum_AB595189 #0.0001 , Corallium_rubrum_NC_022864 #0.0001 ) #0.0271084 , (Pleurocorallium_elatius_NC_022804 #0.0001 , Pleurocorallium_konojoi_NC_015406 #0.0001 ) #0.0494386 ) #0.0001 , Sibogagorgia_cauliflora_NC_026193 #0.443706 ) #0.133003 ) #34.3792 , Paraminabea_aldersladei_NC_018790 #0.0786613 ) #0.0627182 ) #0.0001 , (((((((Anthoptilum_grandiflorum_NC_044086 #0.0001 , Anthoptilum_sp__1_RH_2019_MK919656 #999 ) #0.0001 , (Umbellula_huxleyi_MK919668 #0.0001 , Umbellula_sp__1_RH_2019_MK919669 #0.088488 ) #0.236184 ) #0.0001 , Virgularia_mirabilis_NC_044091 #0.0001 ) #0.0001 , (Funiculina_quadrangularis_NC_044078 #0.63365 , ((Kophobelemnon_sp__1_RH_2019_MK919660 #0.0001 , Kophobelemnon_sp__3_RH_2019_MK919661 #0.323362 ) #0.0736129 , Kophobelemnon_sp__4_RH_2019_MK919662 #0.049277 ) #0.0001 ) #0.0001 ) #0.0001 , (Halipteris_cf__finmarchica_RH_2019_MK919659 #999 , Umbellula_sp__2_RH_2019_MK919670 #0.0001 ) #0.957463 ) #0.0001 , ((((Distichoptilum_gracile_NC_044077 #0.0001 , (Pennatula_cf__inflata_RH_2019_MK919666 #0.0001 , Pennatula_grandis_NC_044088 #0.0001 ) #0.103547 ) #0.0001 , ((Pennatula_aculeata_NC_044087 #0.0001 , Pennatula_cf__aculeata_RH_2019_MK919664 #0.0001 ) #0.141964 , Renilla_muelleri_NC_018378 #0.110961 ) #0.282397 ) #0.0001 , Protoptilum_carpenteri_NC_044089 #0.314131 ) #0.0001 , Stylatula_elongata_NC_018380 #0.233102 ) #0.0001 ) #0.668679 , Junceella_fragilis_NC_024181 #0.0137716 ) #0.684405 ) #0.155159 , Heliopora_coerulea_NC_020375 #0.0940271 ) #0.775883 , ((Callogorgia_cf__gracilis_EE_2018_MH719202 #0.0780708 , (Plumarella_adhaerans_NC_046480 #0.0001 , Plumarella_spinosa_NC_046465 #0.0001 ) #0.0001 ) #0.0001 , Narella_hawaiiensis_NC_026192 #0.0001 ) #999 ) #0.15804 , Keratoisidinae_sp__BAL208_1_NC_010764 #0.0001 );

NAD2:

**dS tree:**

(Acanella_arbuscula_NC_011016: 0.017972, ((((((((((((((((((((((((((((((Acropora_aculeus_KT001202: 0.000002, (Acropora_horrida_NC_022825: 0.000001, LC201842_1_LC201842_1__LC201842_1: 0.000005): 0.000005): 0.000001, LC201815_1_LC201815_1__LC201815_1: 0.000005): 0.000001, ((((Acropora_aspera_NC_022827: 0.000005, Acropora_muricata_NC_022824: 0.000005): 0.000001, LC201828_1_LC201828_1__LC201828_1: 0.000004): 0.000001, (((Acropora_digitifera_NC_022830: 0.000005, LC201849_1_LC201849_1__LC201849_1: 0.000001): 0.000005, Acropora_florida_NC_022828: 0.000001): 0.000005, Acropora_humilis_NC_022823: 0.000001): 0.000001): 0.000005, (Acropora_hyacinthus_NC_022826: 0.000005, LC201817_1_LC201817_1__LC201817_1: 0.000005): 0.000001): 0.000001): 0.000001, (Acropora_divaricata_NC_022832: 0.005448, (((Acropora_nasuta_NC_022831: 0.000001, Acropora_valida_NC_042742: 0.000005): 0.000005, LC201847_1_LC201847_1__LC201847_1: 0.000001): 0.000005, LC201820_1_LC201820_1__LC201820_1: 0.000005): 0.005458): 0.000005): 0.000001, Acropora_robusta_NC_022833: 0.005453): 0.000001, LC201813_1_LC201813_1__LC201813_1: 0.000001): 0.000003, ((Acropora_tenuis_NC_003522: 0.000001, Acropora_yongei_NC_022829: 0.000001): 0.000001, LC201816_1_LC201816_1__LC201816_1: 0.000005): 0.000001): 0.027534, (Isopora_palifera_NC_024091: 0.005551, Isopora_togianensis_NC_024089: 0.000005): 0.012483): 0.113106, ((Anacropora_matthai_NC_006898: 0.021659, Montipora_efflorescens_NC_040137: 0.000000): 0.005348, (Montipora_aequituberculata_NC_037359: 0.038819, Montipora_cactus_NC_006902: 0.000009): 0.000005): 0.105527): 0.017942, (Alveopora_japonica_NC_040136: 0.000005, Alveopora_sp__MFL_2014_KJ634271: 0.000005): 0.104992): 0.021740, (Astreopora_explanata_NC_024090: 0.000005, Astreopora_myriophthalma_NC_024092: 0.000009): 0.050123): 0.024491, ((Agaricia_humilis_NC_008160: 0.007067, Pavona_decussata_NC_026527: 0.041100): 0.111273, (Fimbriaphyllia_ancora_NC_015641: 0.041220, Galaxea_fascicularis_NC_029696: 0.038846): 0.179279): 0.038823): 0.038004, ((Pseudosiderastrea_formosa_NC_026530: 0.011377, Pseudosiderastrea_tayamai_NC_026531: 0.011613): 0.030948, Siderastrea_radians_NC_008167: 0.059873): 0.042948): 0.010314, ((((((Dendrophyllia_arbuscula_NC_027590: 0.000005, Tubastraea_coccinea_NC_026025: 0.011355): 0.005638, ((Tubastraea_diaphana_MK959042: 0.000005, Tubastraea_tagusensis_NC_030352: 0.000005): 0.000005, Tubastraea_micranthus_MK959041: 0.000005): 0.005671): 0.011941, Dendrophyllia_cribrosa_NC_026026: 0.005066): 0.030948, Turbinaria_peltata_NC_024671: 0.027964): 0.005453, (((Goniopora_columna_NC_015643: 0.000005, Goniopora_djiboutiensis_NC_045931: 0.000005): 0.010968, Goniopora_lobata_MN795054: 0.022705): 0.064278, ((Porites_fontanesii_NC_037434: 0.027885, ((Porites_panamensis_NC_024182: 0.005444, Porites_sverdrupi_KU956960: 0.000005): 0.000005, Porites_porites_NC_008166: 0.000005): 0.000005): 0.005471, (Porites_harrisoni_NC_037435: 0.010930, ((Porites_lobata_NC_030186: 0.000005, Porites_okinawensis_NC_015644: 0.000005): 0.005454, (Porites_lutea_NC_029695: 0.005439, Porites_rus_NC_027526: 0.000005): 0.000005): 0.000005): 0.000005): 0.058713): 0.077609): 0.000020, Fungiacyathus_stephanus_NC_015640: 0.096120): 0.039318): 0.236995, Gardineria_hawaiiensis_NC_056290: 0.200290): 0.100159, (((((Amplexidiscus_fenestrafer_NC_027101: 0.012192, (Discosoma_sp__MH308003: 0.012144, (Rhodactis_indosinensis_NC_027103: 0.005486, Rhodactis_sp__CASIZ_171755_NC_008158: 0.005500): 0.004246): 0.000005): 0.006837, (Discosoma_nummiforme_NC_027100: 0.004798, ((Discosoma_sp__CASIZ_168915_NC_008071: 0.000005, Discosoma_sp__CASIZ_168916_NC_008072: 0.000005): 0.000005, Platyzoanthus_mussoides_NC_027104: 0.000009): 0.022971): 0.009247): 0.111691, (Pseudocorynactis_sp__SIO_KP938437: 0.170942, (Ricordea_florida_NC_008159: 0.045318, Ricordea_yuma_NC_027106: 0.018174): 0.099129): 0.013353): 0.025767, Corynactis_californica_NC_027102: 0.124970): 0.051508, Corallimorphus_profundus_KP938440: 0.108754): 0.090710): 0.000178, ((((((Astrangia_sp__JVK_2006_NC_008161: 0.176388, (((Colpophyllia_natans_NC_008162: 0.006125, Mussa_angulosa_NC_008163: 0.006304): 0.015183, ((((((Dipsastraea_favus_NC_046690: 0.012462, Dipsastraea_rotumana_NC_044074: 0.006030): 0.006202, Favites_abdita_NC_035879: 0.018649): 0.000005, Platygyra_carnosa_NC_020049: 0.037572): 0.000005, (Favites_pentagona_NC_034916: 0.037630, Hydnophora_exesa_NC_042795: 0.025156): 0.000005): 0.045661, ((Orbicella_annularis_NC_007224: 0.000005, Orbicella_faveolata_NC_007226: 0.000005): 0.000005, Orbicella_franksi_NC_007225: 0.000005): 0.058465): 0.016754, Echinophyllia_aspera_NC_040169: 0.088952): 0.000005): 0.079147, Plesiastrea_versipora_NC_042481: 0.043400): 0.129938): 0.117088, (Polycyathus_sp__MFL_2011_NC_015642: 0.017208, Psammocora_nierstraszi_MT576637: 0.031618): 0.292983): 0.101659, Madrepora_oculata_NC_018364: 0.521713): 0.012507, (Madracis_myriaster_NC_011160: 0.056472, ((Pocillopora_damicornis_NC_009797: 0.000005, Pocillopora_grandis_NC_009798: 0.000005): 0.021449, ((Seriatopora_caliendrum_NC_010245: 0.000005, Seriatopora_hystrix_NC_010244: 0.000005): 0.000032, Stylophora_pistillata_NC_011162: 0.021431): 0.000000): 0.025220): 0.500437): 0.344902, ((Desmophyllum_dianthus_NC_034275: 0.000005, Desmophyllum_pertusum_NC_015143: 0.005935): 0.011013, Solenosmilia_variabilis_NC_025472: 0.013066): 0.506018): 1.075194, Paraconotrochus_antarcticus_NC_056275: 2.301645): 0.417823): 0.585063, ((((((Antipathes_cf__dichotoma_NB_2020_MT318841: 0.000000, Stichopathes_abyssicola_MT318856: 0.000000): 0.086785, (Phanopathes_sp__NB_2020_MT318852: 0.182138, Stichopathes_sp__n__NB_2020_MT318857: 0.101392): 0.065769): 0.101258, ((Stichopathes_luetkeni_NC_018377: 0.005608, Stichopathes_sp__SCBUCN_8850_MZ157399: 0.000005): 0.005041, Stichopathes_sp__SCBUCN_8849_MZ157400: 0.017753): 0.280351): 0.015841, ((Myriopathes_japonica_NC_027667: 0.033373, Tanacetipathes_thamnea_NC_046843: 0.021515): 0.034455, Tylopathes_sp__n__NB_2020_MT318859: 0.003884): 0.227628): 0.021470, (((((((Bathypathes_sp__1_NB_2020_MT318844: 0.005325, Bathypathes_sp__n__2_NB_2020_MT318842: 0.000000): 0.000000, Stauropathes_arctica_MT318854: 0.000000): 0.000009, Bathypathes_sp__n__3_NB_2020_MT318843: 0.000000): 0.005290, Stauropathes_cf__punctata_NB_2020_MT318855: 0.016032): 0.000000, Telopathes_sp__NB_2020_MT318858: 0.016038): 0.017675, (Dendrobathypathes_sp__n__NB_2020_MT318845: 0.055558, ((Parantipathes_cf__hirondelle_NB_2020_MT318849: 0.000000, Parantipathes_hirondelle_MT318850: 0.000000): 0.000000, (Parantipathes_sp__NB_2020_MT318851: 0.000000, Sibopathes_cf__macrospina_NB_2020_MT318853: 0.005361): 0.000000): 0.012476): 0.016411): 0.043348, (Chrysopathes_formosa_NC_008411: 0.005656, Trissopathes_cf__tetracrada_NB_2020_MT318840: 0.026700): 0.035586): 0.045315): 0.069837, (Leiopathes_cf__glaberrima_NB_2020_MT318846: 0.000000, Leiopathes_expansa_MT318847: 0.000000): 0.074706): 0.302765): 0.089936, (Epizoanthus_illoricatus_NC_046474: 0.081888, (((Hydrozoanthus_gracilis_NC_046400: 0.049848, ((Neozoanthus_aff__uchina_AP_2020_MN873592: 0.009916, ((Zoanthus_cf__pulchellus_AP_2020_MN873599: 0.000000, ((Zoanthus_cf__sociatus_AP_2020_MN873600: 0.000000, Zoanthus_sociatus_NC_046476: 0.000000): 0.000000, Zoanthus_sp__AP_2020_MN873602: 0.000000): 0.000000): 0.000000, Zoanthus_sansibaricus_NC_035578: 0.000000): 0.054782): 0.057059, (Palythoa_heliodiscus_NC_035579: 0.025863, ((Palythoa_mizigama_NC_046403: 0.000000, Palythoa_mutuki_NC_046404: 0.005089): 0.000000, Sphenopus_marsupialis_NC_046406: 0.010248): 0.000000): 0.038721): 0.004608): 0.034083, ((Parazoanthus_elongatus_NC_046405: 0.005037, Parazoanthus_swiftii_NC_046475: 0.000000): 0.005069, Savalia_savaglia_DQ825686: 0.025462): 0.000014): 0.120339, (Microzoanthus_occultus_NC_046401: 1.232975, Nanozoanthus_harenaceus_NC_046402: 1.452467): 1.229894): 0.262933): 0.845452): 0.166357, (((((((Actinia_equina_NC_039929: 0.000005, Actinia_tenebrosa_NC_044902: 0.000005): 0.043387, (((Anemonia_manjano_NC_037178: 0.029256, Heteractis_aurora_NC_047219: 0.005526): 0.025060, (Anemonia_sulcata_NC_049065: 0.000005, Anemonia_viridis_NC_037177: 0.000005): 0.015060): 0.000005, Anthopleura_anjunae_NC_030274: 0.046863): 0.007991): 0.025125, Phymanthus_crucifer_NC_027614: 0.037541): 0.268459, ((((Bolocera_sp__BZ_2016_KU507297: 0.000009, (Bolocera_tuediae_NC_022470: 0.000005, Liponema_brevicorne_NC_047221: 0.000005): 0.000009): 0.000005, (Entacmaea_quadricolor_NC_049066: 0.040318, Isosicyonis_striata_KR051006: 0.029157): 0.005270): 0.022311, Stichodactyla_haddoni_MW760873: 0.102773): 0.007845, Epiactis_japonica_NC_047217: 0.036174): 0.184957): 0.001615, ((((Alicia_sansibarensis_NC_027610: 0.497234, (Haloclava_producta_NC_047218: 0.030943, Sagartia_ornata_NC_027615: 0.049435): 0.059455): 0.063485, (Diadumene_lineata_NC_045515: 0.236315, Metridium_senile_NC_000933: 0.075646): 0.090911): 0.132921, Paraphelliactis_xishaensis_MT997141: 0.294860): 0.197397, Antholoba_achates_NC_027611: 0.463453): 0.000005): 0.045074, Halcampoides_purpureus_NC_027612: 0.204903): 0.172775, Nematostella_sp__JVK_2006_NC_008164: 0.984694): 0.217496): 3.435761, (((((((Aurelia_aurita_NC_008446: 1.036143, (Aurelia_coerulea_NC_046792: 0.372704, Aurelia_limbata_NC_046691: 0.684782): 0.120611): 0.000172, (Aurelia_sp__3_sensu_Dawson_et_al___2005__LC005413: 0.978047, Aurelia_sp__4_sensu_Dawson_et_al___2005__LC005414: 0.535419): 0.052214): 1.982779, ((Nemopilema_nomurai_NC_035740: 1.404825, Rhopilema_esculentum_NC_035741: 2.306709): 0.411748, Stomolophus_sp__CG_2019_MK157198: 2.573724): 0.268526): 0.000173, Cassiopea_xamachana_NC_016466: 1.294612): 0.000361, (Chrysaora_pacifica_NC_046775: 0.000018, Chrysaora_quinquecirrha_NC_020459: 0.037674): 2.460974): 0.000660, ((((((Blackfordia_virginica_NC_053946: 2.020674, Eutima_sp__BMK_2020_MW066348: 1.482815): 0.204725, Laomedea_flexuosa_JN700945: 1.669475): 0.000432, (Clava_multicornis_NC_016465: 1.451162, (Turritopsis_dohrnii_NC_031213: 1.342931, Turritopsis_lata_NC_056128: 0.726270): 1.169173): 0.000194): 0.000179, Spirocodon_saltatrix_NC_049868: 2.199195): 0.000036, ((Hydra_oligactis_NC_010214: 2.595868, Hydra_sinensis_NC_021406: 1.682141): 0.000696, Nemalecium_lighti_MZ457217: 3.097486): 0.000309): 0.000625, (Craspedacusta_sowerbii_NC_018537: 4.762763, Cubaia_aphrodite_NC_016467: 64.529558): 0.000472): 0.000151): 0.000324, (Haliclystus_antarcticus_NC_030337: 1.708883, OU744455_1_OU744455_1__OU744455_1: 0.695250): 1.514667): 0.000336): 3.210576, Carijoa_riisei_NC_048963: 0.245203): 0.000075, (Sarcophyton_trocheliophorum_MK994517: 0.090962, (((Sinularia_acuta_MW987591: 0.017227, (Sinularia_maxima_MN485891: 0.017165, Sinularia_penghuensis_MW256412: 0.000001): 0.000005): 0.007103, Sinularia_cf__cruciata_GS_2016_NC_034318: 0.029018): 0.017106, (Sinularia_ceramensis_NC_044122: 0.000005, Sinularia_peculiaris_NC_018379: 0.011480): 0.016418): 0.028685): 0.033974): 0.000005, (((((((Antillogorgia_bipinnata_NC_008157: 0.094326, ((((Eugorgia_mutabilis_NC_035665: 0.022634, Leptogorgia_alba_NC_035669: 0.005576): 0.000000, (Leptogorgia_hebes_MN052675: 0.017129, Leptogorgia_virgulata_MK301586: 0.034667): 0.005464): 0.005555, Leptogorgia_sp__USNM_1437444_KY559412: 0.000000): 0.011189, Pacifigorgia_cairnsi_NC_035668: 0.059314): 0.017400): 0.000005, (((Leptogorgia_capverdensis_NC_035663: 0.000000, Leptogorgia_sarmentosa_NC_035670: 0.000000): 0.005666, Leptogorgia_gaini_KY559404: 0.005623): 0.034822, Leptogorgia_cf__palma_AP_2017_KY559406: 0.040157): 0.000001): 0.052700, (Eunicella_albicans_NC_035666: 0.044823, (Eunicella_cavolini_NC_035667: 0.000000, Eunicella_verrucosa_MW588805: 0.000000): 0.017433): 0.011796): 0.015219, (Calicogorgia_granulosa_NC_023345: 0.202123, Euplexaura_crassa_NC_020458: 0.067537): 0.002894): 0.010441, Muricea_crassa_NC_029697: 0.080492): 0.010344, ((((((Dendronephthya_castanea_NC_023343: 0.000000, Dendronephthya_mollis_NC_020456: 0.000000): 0.000000, Dendronephthya_gigantea_NC_013573: 0.000000): 0.000000, (Dendronephthya_putteri_NC_036022: 0.000000, Dendronephthya_suensoni_NC_022809: 0.000000): 0.000000): 0.019931, Scleronephthya_gracillimum_NC_023344: 0.014539): 0.006155, (Echinogorgia_complexa_NC_020457: 0.025261, Paramuricea_clavata_NC_034749: 0.269257): 0.000005): 0.053190, Trachythela_sp__YZ_2021_MW238423: 0.055337): 0.000005): 0.008900, (Incrustatus_comauensis_MT254531: 0.044044, Telestula_humilis_MT254527: 0.020918): 0.004355): 0.008996): 0.082857, Briareum_asbestinum_NC_008073: 0.041810): 0.004185, (Telestula_cf__batoni_AP_2020_MT254530: 0.000005, Telestula_septentrionalis_MT254532: 0.034526): 0.028977): 0.017470, (((Anthomastus_sp__USNM_1081145_KM015353: 0.025262, Anthomastus_sp__USNM_1171062_KM015352: 0.018103): 0.037357, (((Corallium_japonicum_AB595189: 0.005954, Corallium_rubrum_NC_022864: 0.005777): 0.017683, (Pleurocorallium_elatius_NC_022804: 0.005743, Pleurocorallium_konojoi_NC_015406: 0.000005): 0.041751): 0.017975, Sibogagorgia_cauliflora_NC_026193: 0.000012): 0.024238): 0.024016, Paraminabea_aldersladei_NC_018790: 0.029480): 0.041114): 0.026231, (((((((Anthoptilum_grandiflorum_NC_044086: 0.011129, Anthoptilum_sp__1_RH_2019_MK919656: 0.000000): 0.000010, (Umbellula_huxleyi_MK919668: 0.000001, Umbellula_sp__1_RH_2019_MK919669: 0.033682): 0.016950): 0.000005, Virgularia_mirabilis_NC_044091: 0.063790): 0.005526, (Funiculina_quadrangularis_NC_044078: 0.005644, ((Kophobelemnon_sp__1_RH_2019_MK919660: 0.000000, Kophobelemnon_sp__3_RH_2019_MK919661: 0.011061): 0.016525, Kophobelemnon_sp__4_RH_2019_MK919662: 0.005746): 0.005530): 0.022271): 0.005445, (Halipteris_cf__finmarchica_RH_2019_MK919659: 0.048215, Umbellula_sp__2_RH_2019_MK919670: 0.029876): 0.010641): 0.000011, ((((Distichoptilum_gracile_NC_044077: 0.000005, (Pennatula_cf__inflata_RH_2019_MK919666: 0.005465, Pennatula_grandis_NC_044088: 0.005530): 0.033745): 0.013712, ((Pennatula_aculeata_NC_044087: 0.000000, Pennatula_cf__aculeata_RH_2019_MK919664: 0.000000): 0.010877, Renilla_muelleri_NC_018378: 0.129050): 0.049569): 0.035715, Protoptilum_carpenteri_NC_044089: 0.033014): 0.004557, Stylatula_elongata_NC_018380: 0.066900): 0.032141): 0.013319, Junceella_fragilis_NC_024181: 0.172572): 0.049440): 0.000005, Heliopora_coerulea_NC_020375: 0.116341): 0.022748, ((Callogorgia_cf__gracilis_EE_2018_MH719202: 0.028267, (Plumarella_adhaerans_NC_046480: 0.000000, Plumarella_spinosa_NC_046465: 0.000000): 0.011183): 0.005774, Narella_hawaiiensis_NC_026192: 0.011176): 0.034102): 0.057340, Keratoisidinae_sp__BAL208_1_NC_010764: 0.010910);

**dN tree:**

(Acanella_arbuscula_NC_011016: 0.014003, ((((((((((((((((((((((((((((((Acropora_aculeus_KT001202: 0.000001, (Acropora_horrida_NC_022825: 0.000002, LC201842_1_LC201842_1__LC201842_1: 0.000000): 0.000000): 0.000001, LC201815_1_LC201815_1__LC201815_1: 0.000000): 0.000001, ((((Acropora_aspera_NC_022827: 0.000000, Acropora_muricata_NC_022824: 0.000000): 0.000002, LC201828_1_LC201828_1__LC201828_1: 0.000000): 0.000001, (((Acropora_digitifera_NC_022830: 0.000000, LC201849_1_LC201849_1__LC201849_1: 0.000001): 0.000000, Acropora_florida_NC_022828: 0.000001): 0.000000, Acropora_humilis_NC_022823: 0.000002): 0.000001): 0.000000, (Acropora_hyacinthus_NC_022826: 0.000000, LC201817_1_LC201817_1__LC201817_1: 0.000000): 0.000002): 0.000001): 0.000002, (Acropora_divaricata_NC_022832: 0.000001, (((Acropora_nasuta_NC_022831: 0.000001, Acropora_valida_NC_042742: 0.000000): 0.000000, LC201847_1_LC201847_1__LC201847_1: 0.000002): 0.000000, LC201820_1_LC201820_1__LC201820_1: 0.000000): 0.000001): 0.000000): 0.000002, Acropora_robusta_NC_022833: 0.000001): 0.000002, LC201813_1_LC201813_1__LC201813_1: 0.000001): 0.000001, ((Acropora_tenuis_NC_003522: 0.000001, Acropora_yongei_NC_022829: 0.000001): 0.000002, LC201816_1_LC201816_1__LC201816_1: 0.000000): 0.000002): 0.000003, (Isopora_palifera_NC_024091: 0.000001, Isopora_togianensis_NC_024089: 0.000000): 0.000001): 0.001857, ((Anacropora_matthai_NC_006898: 0.000002, Montipora_efflorescens_NC_040137: 0.000002): 0.000001, (Montipora_aequituberculata_NC_037359: 0.009518, Montipora_cactus_NC_006902: 0.001902): 0.000000): 0.000011): 0.003748, (Alveopora_japonica_NC_040136: 0.000000, Alveopora_sp__MFL_2014_KJ634271: 0.000000): 0.001844): 0.005639, (Astreopora_explanata_NC_024090: 0.000000, Astreopora_myriophthalma_NC_024092: 0.001856): 0.000005): 0.012143, ((Agaricia_humilis_NC_008160: 0.003974, Pavona_decussata_NC_026527: 0.003938): 0.016049, (Fimbriaphyllia_ancora_NC_015641: 0.011854, Galaxea_fascicularis_NC_029696: 0.001657): 0.013234): 0.000004): 0.004766, ((Pseudosiderastrea_formosa_NC_026530: 0.001822, Pseudosiderastrea_tayamai_NC_026531: 0.000001): 0.003833, Siderastrea_radians_NC_008167: 0.007191): 0.001955): 0.001827, ((((((Dendrophyllia_arbuscula_NC_027590: 0.000000, Tubastraea_coccinea_NC_026025: 0.000001): 0.000001, ((Tubastraea_diaphana_MK959042: 0.000000, Tubastraea_tagusensis_NC_030352: 0.000000): 0.000000, Tubastraea_micranthus_MK959041: 0.000000): 0.000001): 0.000001, Dendrophyllia_cribrosa_NC_026026: 0.001830): 0.001838, Turbinaria_peltata_NC_024671: 0.005606): 0.003796, (((Goniopora_columna_NC_015643: 0.000000, Goniopora_djiboutiensis_NC_045931: 0.000000): 0.000001, Goniopora_lobata_MN795054: 0.000002): 0.001836, ((Porites_fontanesii_NC_037434: 0.001836, ((Porites_panamensis_NC_024182: 0.000001, Porites_sverdrupi_KU956960: 0.000000): 0.000000, Porites_porites_NC_008166: 0.000000): 0.000000): 0.000001, (Porites_harrisoni_NC_037435: 0.000001, ((Porites_lobata_NC_030186: 0.000000, Porites_okinawensis_NC_015644: 0.000000): 0.000001, (Porites_lutea_NC_029695: 0.000001, Porites_rus_NC_027526: 0.000000): 0.000000): 0.000000): 0.000000): 0.003716): 0.007456): 0.003700, Fungiacyathus_stephanus_NC_015640: 0.011366): 0.012899): 0.010141, Gardineria_hawaiiensis_NC_056290: 0.028632): 0.011746, (((((Amplexidiscus_fenestrafer_NC_027101: 0.001860, (Discosoma_sp__MH308003: 0.001864, (Rhodactis_indosinensis_NC_027103: 0.000001, Rhodactis_sp__CASIZ_171755_NC_008158: 0.000001): 0.000000): 0.000000): 0.001861, (Discosoma_nummiforme_NC_027100: 0.005578, ((Discosoma_sp__CASIZ_168915_NC_008071: 0.000000, Discosoma_sp__CASIZ_168916_NC_008072: 0.000000): 0.000000, Platyzoanthus_mussoides_NC_027104: 0.001849): 0.001854): 0.000001): 0.009381, (Pseudocorynactis_sp__SIO_KP938437: 0.015096, (Ricordea_florida_NC_008159: 0.009262, Ricordea_yuma_NC_027106: 0.003711): 0.007283): 0.000122): 0.001807, Corynactis_californica_NC_027102: 0.016941): 0.009808, Corallimorphus_profundus_KP938440: 0.022645): 0.005315): 0.022395, ((((((Astrangia_sp__JVK_2006_NC_008161: 0.003509, (((Colpophyllia_natans_NC_008162: 0.000001, Mussa_angulosa_NC_008163: 0.001682): 0.003355, ((((((Dipsastraea_favus_NC_046690: 0.001698, Dipsastraea_rotumana_NC_044074: 0.000001): 0.000001, Favites_abdita_NC_035879: 0.003408): 0.000000, Platygyra_carnosa_NC_020049: 0.001712): 0.000000, (Favites_pentagona_NC_034916: 0.000004, Hydnophora_exesa_NC_042795: 0.003408): 0.000000): 0.001688, ((Orbicella_annularis_NC_007224: 0.000000, Orbicella_faveolata_NC_007226: 0.000000): 0.000000, Orbicella_franksi_NC_007225: 0.000000): 0.001691): 0.001679, Echinophyllia_aspera_NC_040169: 0.006810): 0.000000): 0.004998, Plesiastrea_versipora_NC_042481: 0.001650): 0.001380): 0.005902, (Polycyathus_sp__MFL_2011_NC_015642: 0.001764, Psammocora_nierstraszi_MT576637: 0.004635): 0.018797): 0.015881, Madrepora_oculata_NC_018364: 0.037193): 0.005101, (Madracis_myriaster_NC_011160: 0.006938, ((Pocillopora_damicornis_NC_009797: 0.000000, Pocillopora_grandis_NC_009798: 0.000000): 0.006306, ((Seriatopora_caliendrum_NC_010245: 0.000000, Seriatopora_hystrix_NC_010244: 0.000000): 0.001563, Stylophora_pistillata_NC_011162: 0.000002): 0.000002): 0.004310): 0.031360): 0.009113, ((Desmophyllum_dianthus_NC_034275: 0.000000, Desmophyllum_pertusum_NC_015143: 0.000001): 0.000001, Solenosmilia_variabilis_NC_025472: 0.003569): 0.038274): 0.096533, Paraconotrochus_antarcticus_NC_056275: 0.117836): 0.060282): 0.033847, ((((((Antipathes_cf__dichotoma_NB_2020_MT318841: 0.000002, Stichopathes_abyssicola_MT318856: 0.000002): 0.022382, (Phanopathes_sp__NB_2020_MT318852: 0.008010, Stichopathes_sp__n__NB_2020_MT318857: 0.003931): 0.001958): 0.000010, ((Stichopathes_luetkeni_NC_018377: 0.000001, Stichopathes_sp__SCBUCN_8850_MZ157399: 0.000000): 0.000001, Stichopathes_sp__SCBUCN_8849_MZ157400: 0.000002): 0.001902): 0.003919, ((Myriopathes_japonica_NC_027667: 0.000003, Tanacetipathes_thamnea_NC_046843: 0.000002): 0.000003, Tylopathes_sp__n__NB_2020_MT318859: 0.000000): 0.007885): 0.000002, (((((((Bathypathes_sp__1_NB_2020_MT318844: 0.000001, Bathypathes_sp__n__2_NB_2020_MT318842: 0.000002): 0.000002, Stauropathes_arctica_MT318854: 0.000002): 0.001946, Bathypathes_sp__n__3_NB_2020_MT318843: 0.000002): 0.001948, Stauropathes_cf__punctata_NB_2020_MT318855: 0.000002): 0.000002, Telopathes_sp__NB_2020_MT318858: 0.000002): 0.000002, (Dendrobathypathes_sp__n__NB_2020_MT318845: 0.007818, ((Parantipathes_cf__hirondelle_NB_2020_MT318849: 0.000002, Parantipathes_hirondelle_MT318850: 0.000002): 0.000002, (Parantipathes_sp__NB_2020_MT318851: 0.000002, Sibopathes_cf__macrospina_NB_2020_MT318853: 0.000001): 0.000002): 0.001947): 0.003871): 0.003924, (Chrysopathes_formosa_NC_008411: 0.001948, Trissopathes_cf__tetracrada_NB_2020_MT318840: 0.000003): 0.001955): 0.001956): 0.000007, (Leiopathes_cf__glaberrima_NB_2020_MT318846: 0.000002, Leiopathes_expansa_MT318847: 0.000002): 0.003918): 0.015851): 0.000009, (Epizoanthus_illoricatus_NC_046474: 0.002287, (((Hydrozoanthus_gracilis_NC_046400: 0.008785, ((Neozoanthus_aff__uchina_AP_2020_MN873592: 0.002117, ((Zoanthus_cf__pulchellus_AP_2020_MN873599: 0.000002, ((Zoanthus_cf__sociatus_AP_2020_MN873600: 0.000002, Zoanthus_sociatus_NC_046476: 0.000002): 0.000002, Zoanthus_sp__AP_2020_MN873602: 0.000002): 0.000002): 0.000002, Zoanthus_sansibaricus_NC_035578: 0.000002): 0.002116): 0.000006, (Palythoa_heliodiscus_NC_035579: 0.004322, ((Palythoa_mizigama_NC_046403: 0.000002, Palythoa_mutuki_NC_046404: 0.000001): 0.000002, Sphenopus_marsupialis_NC_046406: 0.000001): 0.000002): 0.002245): 0.000000): 0.002208, ((Parazoanthus_elongatus_NC_046405: 0.000001, Parazoanthus_swiftii_NC_046475: 0.000002): 0.000001, Savalia_savaglia_DQ825686: 0.000003): 0.002228): 0.010920, (Microzoanthus_occultus_NC_046401: 0.055744, Nanozoanthus_harenaceus_NC_046402: 0.031620): 0.079502): 0.006879): 0.020962): 0.000017, (((((((Actinia_equina_NC_039929: 0.000000, Actinia_tenebrosa_NC_044902: 0.000000): 0.001855, (((Anemonia_manjano_NC_037178: 0.001858, Heteractis_aurora_NC_047219: 0.007449): 0.003704, (Anemonia_sulcata_NC_049065: 0.000000, Anemonia_viridis_NC_037177: 0.000000): 0.001871): 0.000000, Anthopleura_anjunae_NC_030274: 0.003731): 0.000001): 0.000003, Phymanthus_crucifer_NC_027614: 0.001856): 0.014430, ((((Bolocera_sp__BZ_2016_KU507297: 0.001888, (Bolocera_tuediae_NC_022470: 0.000000, Liponema_brevicorne_NC_047221: 0.000000): 0.001888): 0.000000, (Entacmaea_quadricolor_NC_049066: 0.001916, Isosicyonis_striata_KR051006: 0.001895): 0.000001): 0.002436, Stichodactyla_haddoni_MW760873: 0.003220): 0.001875, Epiactis_japonica_NC_047217: 0.006234): 0.006329): 0.000578, ((((Alicia_sansibarensis_NC_027610: 0.050371, (Haloclava_producta_NC_047218: 0.001890, Sagartia_ornata_NC_027615: 0.001892): 0.000006): 0.000006, (Diadumene_lineata_NC_045515: 0.001893, Metridium_senile_NC_000933: 0.003816): 0.001902): 0.005830, Paraphelliactis_xishaensis_MT997141: 0.000029): 0.013159, Antholoba_achates_NC_027611: 0.013846): 0.000000): 0.003757, Halcampoides_purpureus_NC_027612: 0.012909): 0.016883, Nematostella_sp__JVK_2006_NC_008164: 0.027056): 0.027810): 0.142702, (((((((Aurelia_aurita_NC_008446: 0.032380, (Aurelia_coerulea_NC_046792: 0.019272, Aurelia_limbata_NC_046691: 0.004628): 0.026928): 0.017157, (Aurelia_sp__3_sensu_Dawson_et_al___2005__LC005413: 0.032045, Aurelia_sp__4_sensu_Dawson_et_al___2005__LC005414: 0.030579): 0.020537): 0.083958, ((Nemopilema_nomurai_NC_035740: 0.045988, Rhopilema_esculentum_NC_035741: 0.060733): 0.037115, Stomolophus_sp__CG_2019_MK157198: 0.119484): 0.030655): 0.017033, Cassiopea_xamachana_NC_016466: 0.226604): 0.090930, (Chrysaora_pacifica_NC_046775: 0.001769, Chrysaora_quinquecirrha_NC_020459: 0.000004): 0.124118): 0.219219, ((((((Blackfordia_virginica_NC_053946: 0.072100, Eutima_sp__BMK_2020_MW066348: 0.130804): 0.033730, Laomedea_flexuosa_JN700945: 0.129759): 0.092334, (Clava_multicornis_NC_016465: 0.102506, (Turritopsis_dohrnii_NC_031213: 0.051661, Turritopsis_lata_NC_056128: 0.056336): 0.236487): 0.045315): 0.041015, Spirocodon_saltatrix_NC_049868: 0.241551): 0.004839, ((Hydra_oligactis_NC_010214: 0.101790, Hydra_sinensis_NC_021406: 0.141809): 0.155760, Nemalecium_lighti_MZ457217: 0.539658): 0.090012): 0.154562, (Craspedacusta_sowerbii_NC_018537: 0.137476, Cubaia_aphrodite_NC_016467: 0.174469): 0.062645): 0.031076): 0.081792, (Haliclystus_antarcticus_NC_030337: 0.041280, OU744455_1_OU744455_1__OU744455_1: 0.045189): 0.379440): 0.048246): 0.185061, Carijoa_riisei_NC_048963: 0.000025): 0.005858, (Sarcophyton_trocheliophorum_MK994517: 0.009586, (((Sinularia_acuta_MW987591: 0.000002, (Sinularia_maxima_MN485891: 0.000002, Sinularia_penghuensis_MW256412: 0.000001): 0.000000): 0.000001, Sinularia_cf__cruciata_GS_2016_NC_034318: 0.000003): 0.005826, (Sinularia_ceramensis_NC_044122: 0.000000, Sinularia_peculiaris_NC_018379: 0.000001): 0.001932): 0.000003): 0.000003): 0.000000, (((((((Antillogorgia_bipinnata_NC_008157: 0.000009, ((((Eugorgia_mutabilis_NC_035665: 0.003958, Leptogorgia_alba_NC_035669: 0.000001): 0.000002, (Leptogorgia_hebes_MN052675: 0.001966, Leptogorgia_virgulata_MK301586: 0.000003): 0.000001): 0.000001, Leptogorgia_sp__USNM_1437444_KY559412: 0.000002): 0.001982, Pacifigorgia_cairnsi_NC_035668: 0.005974): 0.001973): 0.000000, (((Leptogorgia_capverdensis_NC_035663: 0.000002, Leptogorgia_sarmentosa_NC_035670: 0.000002): 0.000001, Leptogorgia_gaini_KY559404: 0.007867): 0.001970, Leptogorgia_cf__palma_AP_2017_KY559406: 0.003967): 0.000002): 0.000005, (Eunicella_albicans_NC_035666: 0.000004, (Eunicella_cavolini_NC_035667: 0.000002, Eunicella_verrucosa_MW588805: 0.000002): 0.001971): 0.000001): 0.000002, (Calicogorgia_granulosa_NC_023345: 0.016276, Euplexaura_crassa_NC_020458: 0.001918): 0.002013): 0.000001, Muricea_crassa_NC_029697: 0.005821): 0.000001, ((((((Dendronephthya_castanea_NC_023343: 0.000002, Dendronephthya_mollis_NC_020456: 0.000002): 0.000002, Dendronephthya_gigantea_NC_013573: 0.000002): 0.000002, (Dendronephthya_putteri_NC_036022: 0.000002, Dendronephthya_suensoni_NC_022809: 0.000002): 0.000002): 0.001949, Scleronephthya_gracillimum_NC_023344: 0.003887): 0.000001, (Echinogorgia_complexa_NC_020457: 0.000003, Paramuricea_clavata_NC_034749: 0.014152): 0.000000): 0.009814, Trachythela_sp__YZ_2021_MW238423: 0.004145): 0.000000): 0.000001, (Incrustatus_comauensis_MT254531: 0.000004, Telestula_humilis_MT254527: 0.000002): 0.003899): 0.000001): 0.015985, Briareum_asbestinum_NC_008073: 0.009978): 0.001777, (Telestula_cf__batoni_AP_2020_MT254530: 0.000000, Telestula_septentrionalis_MT254532: 0.000003): 0.001944): 0.000002, (((Anthomastus_sp__USNM_1081145_KM015353: 0.003919, Anthomastus_sp__USNM_1171062_KM015352: 0.000002): 0.003896, (((Corallium_japonicum_AB595189: 0.000001, Corallium_rubrum_NC_022864: 0.000001): 0.001913, (Pleurocorallium_elatius_NC_022804: 0.000001, Pleurocorallium_konojoi_NC_015406: 0.000000): 0.001918): 0.000002, Sibogagorgia_cauliflora_NC_026193: 0.001910): 0.003854): 0.000002, Paraminabea_aldersladei_NC_018790: 0.009785): 0.001942): 0.000003, (((((((Anthoptilum_grandiflorum_NC_044086: 0.000001, Anthoptilum_sp__1_RH_2019_MK919656: 0.000002): 0.001970, (Umbellula_huxleyi_MK919668: 0.000002, Umbellula_sp__1_RH_2019_MK919669: 0.003926): 0.005902): 0.000000, Virgularia_mirabilis_NC_044091: 0.003935): 0.000001, (Funiculina_quadrangularis_NC_044078: 0.001986, ((Kophobelemnon_sp__1_RH_2019_MK919660: 0.000002, Kophobelemnon_sp__3_RH_2019_MK919661: 0.000001): 0.001976, Kophobelemnon_sp__4_RH_2019_MK919662: 0.001976): 0.000001): 0.000002): 0.000001, (Halipteris_cf__finmarchica_RH_2019_MK919659: 0.000005, Umbellula_sp__2_RH_2019_MK919670: 0.000003): 0.001958): 0.002206, ((((Distichoptilum_gracile_NC_044077: 0.000000, (Pennatula_cf__inflata_RH_2019_MK919666: 0.000001, Pennatula_grandis_NC_044088: 0.002007): 0.000003): 0.000001, ((Pennatula_aculeata_NC_044087: 0.000002, Pennatula_cf__aculeata_RH_2019_MK919664: 0.000002): 0.001941, Renilla_muelleri_NC_018378: 0.010007): 0.006054): 0.001979, Protoptilum_carpenteri_NC_044089: 0.000003): 0.000000, Stylatula_elongata_NC_018380: 0.001971): 0.005714): 0.003956, Junceella_fragilis_NC_024181: 0.011584): 0.002194): 0.000000, Heliopora_coerulea_NC_020375: 0.013716): 0.000002, ((Callogorgia_cf__gracilis_EE_2018_MH719202: 0.000003, (Plumarella_adhaerans_NC_046480: 0.000002, Plumarella_spinosa_NC_046465: 0.000002): 0.005929): 0.000001, Narella_hawaiiensis_NC_026192: 0.001964): 0.003933): 0.003948, Keratoisidinae_sp__BAL208_1_NC_010764: 0.001887);

**w ratios as labels for TreeView:**

(Acanella_arbuscula_NC_011016 #0.779173 , ((((((((((((((((((((((((((((((Acropora_aculeus_KT001202 #0.809589 , (Acropora_horrida_NC_022825 #2.39384 , LC201842_1_LC201842_1__LC201842_1 #0.0001 ) #0.0001 ) #1.67324 , LC201815_1_LC201815_1__LC201815_1 #0.0001 ) #1.31852 , ((((Acropora_aspera_NC_022827 #0.0001 , Acropora_muricata_NC_022824 #0.000100002 ) #2.44345 , LC201828_1_LC201828_1__LC201828_1 #0.0810424 ) #1.6913 , (((Acropora_digitifera_NC_022830 #0.000100001 , LC201849_1_LC201849_1__LC201849_1 #1.68223 ) #0.0001 , Acropora_florida_NC_022828 #1.42231 ) #0.0001 , Acropora_humilis_NC_022823 #2.43155 ) #1.41576 ) #0.0001 , (Acropora_hyacinthus_NC_022826 #0.0001 , LC201817_1_LC201817_1__LC201817_1 #0.0001 ) #2.36029 ) #1.00995 ) #2.19528 , (Acropora_divaricata_NC_022832 #0.0001 , (((Acropora_nasuta_NC_022831 #1.22284 , Acropora_valida_NC_042742 #0.0001 ) #0.0001 , LC201847_1_LC201847_1__LC201847_1 #1.87603 ) #0.0001 , LC201820_1_LC201820_1__LC201820_1 #0.0001 ) #0.0001 ) #0.0001 ) #1.77992 , Acropora_robusta_NC_022833 #0.0001 ) #2.31662 , LC201813_1_LC201813_1__LC201813_1 #1.21506 ) #0.190538 , ((Acropora_tenuis_NC_003522 #1.20623 , Acropora_yongei_NC_022829 #1.41518 ) #2.14904 , LC201816_1_LC201816_1__LC201816_1 #0.0001 ) #1.95285 ) #0.0001 , (Isopora_palifera_NC_024091 #0.0001 , Isopora_togianensis_NC_024089 #0.0001 ) #0.0001 ) #0.0164177 , ((Anacropora_matthai_NC_006898 #0.0001 , Montipora_efflorescens_NC_040137 #4.92434 ) #0.0001 , (Montipora_aequituberculata_NC_037359 #0.245186 , Montipora_cactus_NC_006902 #206.7 ) #0.0001 ) #0.0001 ) #0.208894 , (Alveopora_japonica_NC_040136 #0.0001 , Alveopora_sp__MFL_2014_KJ634271 #0.0001 ) #0.0175644 ) #0.259371 , (Astreopora_explanata_NC_024090 #0.0001 , Astreopora_myriophthalma_NC_024092 #203.148 ) #0.0001 ) #0.495836 , ((Agaricia_humilis_NC_008160 #0.562258 , Pavona_decussata_NC_026527 #0.0958038 ) #0.144229 , (Fimbriaphyllia_ancora_NC_015641 #0.287575 , Galaxea_fascicularis_NC_029696 #0.042647 ) #0.0738173 ) #0.0001 ) #0.125414 , ((Pseudosiderastrea_formosa_NC_026530 #0.160125 , Pseudosiderastrea_tayamai_NC_026531 #0.0001 ) #0.123841 , Siderastrea_radians_NC_008167 #0.120105 ) #0.0455227 ) #0.177166 , ((((((Dendrophyllia_arbuscula_NC_027590 #0.0001 , Tubastraea_coccinea_NC_026025 #0.0001 ) #0.0001 , ((Tubastraea_diaphana_MK959042 #0.0001 , Tubastraea_tagusensis_NC_030352 #0.0001 ) #0.0001 , Tubastraea_micranthus_MK959041 #0.0001 ) #0.0001 ) #0.0001 , Dendrophyllia_cribrosa_NC_026026 #0.361276 ) #0.059377 , Turbinaria_peltata_NC_024671 #0.200475 ) #0.69622 , (((Goniopora_columna_NC_015643 #0.0001 , Goniopora_djiboutiensis_NC_045931 #0.0001 ) #0.0001 , Goniopora_lobata_MN795054 #0.0001 ) #0.0285646 , ((Porites_fontanesii_NC_037434 #0.0658326 , ((Porites_panamensis_NC_024182 #0.0001 , Porites_sverdrupi_KU956960 #0.0001 ) #0.0001 , Porites_porites_NC_008166 #0.0001 ) #0.0001 ) #0.0001 , (Porites_harrisoni_NC_037435 #0.0001 , ((Porites_lobata_NC_030186 #0.0001 , Porites_okinawensis_NC_015644 #0.0001 ) #0.0001 , (Porites_lutea_NC_029695 #0.0001 , Porites_rus_NC_027526 #0.0001 ) #0.0001 ) #0.0001 ) #0.0001 ) #0.0632952 ) #0.0960724 ) #188.403 , Fungiacyathus_stephanus_NC_015640 #0.118244 ) #0.328072 ) #0.0427911 , Gardineria_hawaiiensis_NC_056290 #0.142954 ) #0.117268 , (((((Amplexidiscus_fenestrafer_NC_027101 #0.152565 , (Discosoma_sp__MH308003 #0.153482 , (Rhodactis_indosinensis_NC_027103 #0.0001 , Rhodactis_sp__CASIZ_171755_NC_008158 #0.0001 ) #0.0001 ) #0.0001 ) #0.272163 , (Discosoma_nummiforme_NC_027100 #1.16255 , ((Discosoma_sp__CASIZ_168915_NC_008071 #0.0001 , Discosoma_sp__CASIZ_168916_NC_008072 #0.0001 ) #0.0001 , Platyzoanthus_mussoides_NC_027104 #203.79 ) #0.0806939 ) #0.0001 ) #0.0839917 , (Pseudocorynactis_sp__SIO_KP938437 #0.0883118 , (Ricordea_florida_NC_008159 #0.204385 , Ricordea_yuma_NC_027106 #0.204203 ) #0.0734704 ) #0.00916011 ) #0.0701123 , Corynactis_californica_NC_027102 #0.135562 ) #0.190413 , Corallimorphus_profundus_KP938440 #0.208226 ) #0.0585972 ) #125.801 , ((((((Astrangia_sp__JVK_2006_NC_008161 #0.0198918 , (((Colpophyllia_natans_NC_008162 #0.0001 , Mussa_angulosa_NC_008163 #0.266789 ) #0.221009 , ((((((Dipsastraea_favus_NC_046690 #0.136274 , Dipsastraea_rotumana_NC_044074 #0.0001 ) #0.0001 , Favites_abdita_NC_035879 #0.182757 ) #0.0001 , Platygyra_carnosa_NC_020049 #0.0455745 ) #0.0001 , (Favites_pentagona_NC_034916 #0.0001 , Hydnophora_exesa_NC_042795 #0.135457 ) #0.0001 ) #0.0369767 , ((Orbicella_annularis_NC_007224 #0.0001 , Orbicella_faveolata_NC_007226 #0.0001 ) #0.0001 , Orbicella_franksi_NC_007225 #0.0001 ) #0.0289178 ) #0.100195 , Echinophyllia_aspera_NC_040169 #0.0765541 ) #0.0001 ) #0.0631422 , Plesiastrea_versipora_NC_042481 #0.0380157 ) #0.0106228 ) #0.0504049 , (Polycyathus_sp__MFL_2011_NC_015642 #0.102526 , Psammocora_nierstraszi_MT576637 #0.146585 ) #0.0641556 ) #0.156214 , Madrepora_oculata_NC_018364 #0.0712894 ) #0.407856 , (Madracis_myriaster_NC_011160 #0.122858 , ((Pocillopora_damicornis_NC_009797 #0.0001 , Pocillopora_grandis_NC_009798 #0.0001 ) #0.293996 , ((Seriatopora_caliendrum_NC_010245 #0.0001 , Seriatopora_hystrix_NC_010244 #0.0001 ) #49.3039 , Stylophora_pistillata_NC_011162 #0.0001 ) #10.3889 ) #0.170914 ) #0.0626649 ) #0.0264227 , ((Desmophyllum_dianthus_NC_034275 #0.0001 , Desmophyllum_pertusum_NC_015143 #0.0001 ) #0.0001 , Solenosmilia_variabilis_NC_025472 #0.273157 ) #0.0756385 ) #0.0897821 , Paraconotrochus_antarcticus_NC_056275 #0.0511963 ) #0.144276 ) #0.0578522 , ((((((Antipathes_cf__dichotoma_NB_2020_MT318841 #10.0026 , Stichopathes_abyssicola_MT318856 #10.1126 ) #0.2579 , (Phanopathes_sp__NB_2020_MT318852 #0.043978 , Stichopathes_sp__n__NB_2020_MT318857 #0.0387741 ) #0.0297708 ) #0.0001 , ((Stichopathes_luetkeni_NC_018377 #0.0001 , Stichopathes_sp__SCBUCN_8850_MZ157399 #0.0001 ) #0.0001 , Stichopathes_sp__SCBUCN_8849_MZ157400 #0.0001 ) #0.00678397 ) #0.247366 , ((Myriopathes_japonica_NC_027667 #0.0001 , Tanacetipathes_thamnea_NC_046843 #0.0001 ) #0.0001 , Tylopathes_sp__n__NB_2020_MT318859 #0.0001 ) #0.0346389 ) #0.0001 , (((((((Bathypathes_sp__1_NB_2020_MT318844 #0.0001 , Bathypathes_sp__n__2_NB_2020_MT318842 #7.78846 ) #7.62748 , Stauropathes_arctica_MT318854 #7.85019 ) #209.675 , Bathypathes_sp__n__3_NB_2020_MT318843 #7.80901 ) #0.368177 , Stauropathes_cf__punctata_NB_2020_MT318855 #0.0001 ) #5.67922 , Telopathes_sp__NB_2020_MT318858 #0.0001 ) #0.0001 , (Dendrobathypathes_sp__n__NB_2020_MT318845 #0.140724 , ((Parantipathes_cf__hirondelle_NB_2020_MT318849 #6.4368 , Parantipathes_hirondelle_MT318850 #6.41201 ) #6.49453 , (Parantipathes_sp__NB_2020_MT318851 #6.45897 , Sibopathes_cf__macrospina_NB_2020_MT318853 #0.0001 ) #6.56601 ) #0.156065 ) #0.235883 ) #0.0905177 , (Chrysopathes_formosa_NC_008411 #0.344489 , Trissopathes_cf__tetracrada_NB_2020_MT318840 #0.0001 ) #0.054946 ) #0.043171 ) #0.0001 , (Leiopathes_cf__glaberrima_NB_2020_MT318846 #7.07785 , Leiopathes_expansa_MT318847 #7.53506 ) #0.0524487 ) #0.0523546 ) #0.0001 , (Epizoanthus_illoricatus_NC_046474 #0.0279226 , (((Hydrozoanthus_gracilis_NC_046400 #0.176229 , ((Neozoanthus_aff__uchina_AP_2020_MN873592 #0.213499 , ((Zoanthus_cf__pulchellus_AP_2020_MN873599 #10.294 , ((Zoanthus_cf__sociatus_AP_2020_MN873600 #9.90273 , Zoanthus_sociatus_NC_046476 #10.3573 ) #10.1665 , Zoanthus_sp__AP_2020_MN873602 #10.1061 ) #10 ) #10.247 , Zoanthus_sansibaricus_NC_035578 #9.93239 ) #0.038624 ) #0.0001 , (Palythoa_heliodiscus_NC_035579 #0.167111 , ((Palythoa_mizigama_NC_046403 #11.1044 , Palythoa_mutuki_NC_046404 #0.0001 ) #11.2848 , Sphenopus_marsupialis_NC_046406 #0.0001 ) #10.1391 ) #0.0579685 ) #0.0001 ) #0.0647865 , ((Parazoanthus_elongatus_NC_046405 #0.0001 , Parazoanthus_swiftii_NC_046475 #11.8339 ) #0.0001 , Savalia_savaglia_DQ825686 #0.0001 ) #156.427 ) #0.0907463 , (Microzoanthus_occultus_NC_046401 #0.0452106 , Nanozoanthus_harenaceus_NC_046402 #0.0217702 ) #0.0646417 ) #0.0261618 ) #0.0247939 ) #0.0001 , (((((((Actinia_equina_NC_039929 #0.0001 , Actinia_tenebrosa_NC_044902 #0.0001 ) #0.0427439 , (((Anemonia_manjano_NC_037178 #0.063516 , Heteractis_aurora_NC_047219 #1.34782 ) #0.147809 , (Anemonia_sulcata_NC_049065 #0.0001 , Anemonia_viridis_NC_037177 #0.0001 ) #0.124242 ) #0.0001 , Anthopleura_anjunae_NC_030274 #0.0796193 ) #0.0001 ) #0.0001 , Phymanthus_crucifer_NC_027614 #0.0494433 ) #0.0537505 , ((((Bolocera_sp__BZ_2016_KU507297 #203.111 , (Bolocera_tuediae_NC_022470 #0.0001 , Liponema_brevicorne_NC_047221 #0.0001 ) #203.15 ) #0.0001 , (Entacmaea_quadricolor_NC_049066 #0.0475128 , Isosicyonis_striata_KR051006 #0.0649923 ) #0.0001 ) #0.109193 , Stichodactyla_haddoni_MW760873 #0.0313269 ) #0.239058 , Epiactis_japonica_NC_047217 #0.172327 ) #0.0342176 ) #0.358251 , ((((Alicia_sansibarensis_NC_027610 #0.101302 , (Haloclava_producta_NC_047218 #0.0610832 , Sagartia_ornata_NC_027615 #0.0382658 ) #0.0001 ) #0.0001 , (Diadumene_lineata_NC_045515 #0.00800865 , Metridium_senile_NC_000933 #0.0504442 ) #0.020925 ) #0.0438603 , Paraphelliactis_xishaensis_MT997141 #0.0001 ) #0.0666603 , Antholoba_achates_NC_027611 #0.0298767 ) #0.0001 ) #0.0833563 , Halcampoides_purpureus_NC_027612 #0.0630026 ) #0.0977187 , Nematostella_sp__JVK_2006_NC_008164 #0.0274762 ) #0.127863 ) #0.0415343 , (((((((Aurelia_aurita_NC_008446 #0.0312504 , (Aurelia_coerulea_NC_046792 #0.0517083 , Aurelia_limbata_NC_046691 #0.00675767 ) #0.223267 ) #99.8846 , (Aurelia_sp__3_sensu_Dawson_et_al___2005__LC005413 #0.0327648 , Aurelia_sp__4_sensu_Dawson_et_al___2005__LC005414 #0.0571121 ) #0.393322 ) #0.0423437 , ((Nemopilema_nomurai_NC_035740 #0.0327357 , Rhopilema_esculentum_NC_035741 #0.0263289 ) #0.0901409 , Stomolophus_sp__CG_2019_MK157198 #0.0464244 ) #0.114161 ) #98.4773 , Cassiopea_xamachana_NC_016466 #0.175036 ) #252.118 , (Chrysaora_pacifica_NC_046775 #98.6401 , Chrysaora_quinquecirrha_NC_020459 #0.0001 ) #0.0504345 ) #331.922 , ((((((Blackfordia_virginica_NC_053946 #0.0356811 , Eutima_sp__BMK_2020_MW066348 #0.0882134 ) #0.164756 , Laomedea_flexuosa_JN700945 #0.0777246 ) #213.661 , (Clava_multicornis_NC_016465 #0.0706374 , (Turritopsis_dohrnii_NC_031213 #0.038469 , Turritopsis_lata_NC_056128 #0.0775692 ) #0.202269 ) #234.077 ) #229.503 , Spirocodon_saltatrix_NC_049868 #0.109836 ) #134.308 , ((Hydra_oligactis_NC_010214 #0.0392123 , Hydra_sinensis_NC_021406 #0.0843027 ) #223.912 , Nemalecium_lighti_MZ457217 #0.174224 ) #291.113 ) #247.433 , (Craspedacusta_sowerbii_NC_018537 #0.0288648 , Cubaia_aphrodite_NC_016467 #0.0027037 ) #132.772 ) #206.04 ) #252.171 , (Haliclystus_antarcticus_NC_030337 #0.0241563 , OU744455_1_OU744455_1__OU744455_1 #0.0649967 ) #0.25051 ) #143.637 ) #0.057641 , Carijoa_riisei_NC_048963 #0.0001 ) #78.0419 , (Sarcophyton_trocheliophorum_MK994517 #0.105382 , (((Sinularia_acuta_MW987591 #0.0001 , (Sinularia_maxima_MN485891 #0.0001 , Sinularia_penghuensis_MW256412 #1.69331 ) #0.0001 ) #0.0001 , Sinularia_cf__cruciata_GS_2016_NC_034318 #0.0001 ) #0.340607 , (Sinularia_ceramensis_NC_044122 #0.0001 , Sinularia_peculiaris_NC_018379 #0.0001 ) #0.117671 ) #0.0001 ) #0.0001 ) #0.0001 , (((((((Antillogorgia_bipinnata_NC_008157 #0.0001 , ((((Eugorgia_mutabilis_NC_035665 #0.17485 , Leptogorgia_alba_NC_035669 #0.0001 ) #4.83045 , (Leptogorgia_hebes_MN052675 #0.11477 , Leptogorgia_virgulata_MK301586 #0.0001 ) #0.0001 ) #0.0001 , Leptogorgia_sp__USNM_1437444_KY559412 #5.67964 ) #0.177165 , Pacifigorgia_cairnsi_NC_035668 #0.10072 ) #0.113363 ) #0.0001 , (((Leptogorgia_capverdensis_NC_035663 #3.81935 , Leptogorgia_sarmentosa_NC_035670 #3.77071 ) #0.0001 , Leptogorgia_gaini_KY559404 #1.39896 ) #0.056572 , Leptogorgia_cf__palma_AP_2017_KY559406 #0.0987753 ) #3.16219 ) #0.0001 , (Eunicella_albicans_NC_035666 #0.0001 , (Eunicella_cavolini_NC_035667 #5.40466 , Eunicella_verrucosa_MW588805 #5.53133 ) #0.113052 ) #0.0001 ) #0.0001 , (Calicogorgia_granulosa_NC_023345 #0.0805231 , Euplexaura_crassa_NC_020458 #0.0283986 ) #0.695661 ) #0.0001 , Muricea_crassa_NC_029697 #0.0723136 ) #0.0001 , ((((((Dendronephthya_castanea_NC_023343 #3.62014 , Dendronephthya_mollis_NC_020456 #3.50849 ) #3.44291 , Dendronephthya_gigantea_NC_013573 #3.43818 ) #3.77834 , (Dendronephthya_putteri_NC_036022 #3.62986 , Dendronephthya_suensoni_NC_022809 #3.30369 ) #3.6233 ) #0.0978061 , Scleronephthya_gracillimum_NC_023344 #0.267359 ) #0.0001 , (Echinogorgia_complexa_NC_020457 #0.0001 , Paramuricea_clavata_NC_034749 #0.052559 ) #0.0001 ) #0.184506 , Trachythela_sp__YZ_2021_MW238423 #0.0749115 ) #0.0001 ) #0.0001 , (Incrustatus_comauensis_MT254531 #0.0001 , Telestula_humilis_MT254527 #0.0001 ) #0.895413 ) #0.0001 ) #0.192924 , Briareum_asbestinum_NC_008073 #0.238648 ) #0.424735 , (Telestula_cf__batoni_AP_2020_MT254530 #0.0001 , Telestula_septentrionalis_MT254532 #0.0001 ) #0.0671032 ) #0.0001 , (((Anthomastus_sp__USNM_1081145_KM015353 #0.15513 , Anthomastus_sp__USNM_1171062_KM015352 #0.0001 ) #0.104284 , (((Corallium_japonicum_AB595189 #0.0001 , Corallium_rubrum_NC_022864 #0.0001 ) #0.108196 , (Pleurocorallium_elatius_NC_022804 #0.0001 , Pleurocorallium_konojoi_NC_015406 #0.0001 ) #0.0459317 ) #0.0001 , Sibogagorgia_cauliflora_NC_026193 #156.596 ) #0.158998 ) #0.0001 , Paraminabea_aldersladei_NC_018790 #0.33192 ) #0.0472392 ) #0.0001 , (((((((Anthoptilum_grandiflorum_NC_044086 #0.0001 , Anthoptilum_sp__1_RH_2019_MK919656 #5.74162 ) #200.094 , (Umbellula_huxleyi_MK919668 #2.30718 , Umbellula_sp__1_RH_2019_MK919669 #0.116548 ) #0.34822 ) #0.0001 , Virgularia_mirabilis_NC_044091 #0.0616805 ) #0.0001 , (Funiculina_quadrangularis_NC_044078 #0.351854 , ((Kophobelemnon_sp__1_RH_2019_MK919660 #5.54841 , Kophobelemnon_sp__3_RH_2019_MK919661 #0.0001 ) #0.119589 , Kophobelemnon_sp__4_RH_2019_MK919662 #0.343867 ) #0.0001 ) #0.0001 ) #0.0001 , (Halipteris_cf__finmarchica_RH_2019_MK919659 #0.0001 , Umbellula_sp__2_RH_2019_MK919670 #0.0001 ) #0.184021 ) #207.226 , ((((Distichoptilum_gracile_NC_044077 #0.0001 , (Pennatula_cf__inflata_RH_2019_MK919666 #0.0001 , Pennatula_grandis_NC_044088 #0.362997 ) #0.0001 ) #0.0001 , ((Pennatula_aculeata_NC_044087 #6.41113 , Pennatula_cf__aculeata_RH_2019_MK919664 #6.84234 ) #0.178412 , Renilla_muelleri_NC_018378 #0.0775459 ) #0.122135 ) #0.0554098 , Protoptilum_carpenteri_NC_044089 #0.0001 ) #0.0001 , Stylatula_elongata_NC_018380 #0.0294659 ) #0.177783 ) #0.297053 , Junceella_fragilis_NC_024181 #0.0671229 ) #0.0443768 ) #0.0001 , Heliopora_coerulea_NC_020375 #0.117897 ) #0.0001 , ((Callogorgia_cf__gracilis_EE_2018_MH719202 #0.0001 , (Plumarella_adhaerans_NC_046480 #5.62756 , Plumarella_spinosa_NC_046465 #5.56976 ) #0.530135 ) #0.0001 , Narella_hawaiiensis_NC_026192 #0.175701 ) #0.115327 ) #0.0688534 , Keratoisidinae_sp__BAL208_1_NC_010764 #0.172994 );

NAD3:

**dS tree:**

(Acanella_arbuscula_NC_011016: 0.000005, ((((((((((((((((((((((((((((((Acropora_aculeus_KT001202: 0.018567, (Acropora_horrida_NC_022825: 0.000005, LC201842_1_LC201842_1__LC201842_1: 0.000005): 0.000005): 0.000005, LC201815_1_LC201815_1__LC201815_1: 0.000005): 0.000005, ((((Acropora_aspera_NC_022827: 0.000005, Acropora_muricata_NC_022824: 0.000005): 0.000005, LC201828_1_LC201828_1__LC201828_1: 0.000005): 0.000005, (((Acropora_digitifera_NC_022830: 0.000005, LC201849_1_LC201849_1__LC201849_1: 0.000005): 0.000005, Acropora_florida_NC_022828: 0.000005): 0.000005, Acropora_humilis_NC_022823: 0.000005): 0.000005): 0.000005, (Acropora_hyacinthus_NC_022826: 0.000005, LC201817_1_LC201817_1__LC201817_1: 0.000005): 0.000005): 0.000055): 0.000005, (Acropora_divaricata_NC_022832: 0.000005, (((Acropora_nasuta_NC_022831: 0.000005, Acropora_valida_NC_042742: 0.000005): 0.000005, LC201847_1_LC201847_1__LC201847_1: 0.000005): 0.000005, LC201820_1_LC201820_1__LC201820_1: 0.000005): 0.018525): 0.000005): 0.000005, Acropora_robusta_NC_022833: 0.000005): 0.000005, LC201813_1_LC201813_1__LC201813_1: 0.018557): 0.000005, ((Acropora_tenuis_NC_003522: 0.038391, Acropora_yongei_NC_022829: 0.000005): 0.000056, LC201816_1_LC201816_1__LC201816_1: 0.000005): 0.000005): 0.037755, (Isopora_palifera_NC_024091: 0.000005, Isopora_togianensis_NC_024089: 0.000005): 0.000005): 0.019019, ((Anacropora_matthai_NC_006898: 0.000005, Montipora_efflorescens_NC_040137: 0.000005): 0.000005, (Montipora_aequituberculata_NC_037359: 0.000005, Montipora_cactus_NC_006902: 0.000005): 0.000005): 0.058609): 0.017130, (Alveopora_japonica_NC_040136: 0.000005, Alveopora_sp__MFL_2014_KJ634271: 0.000005): 0.058158): 0.041904, (Astreopora_explanata_NC_024090: 0.000005, Astreopora_myriophthalma_NC_024092: 0.000005): 0.033315): 0.055996, ((Agaricia_humilis_NC_008160: 0.055103, Pavona_decussata_NC_026527: 0.072841): 0.235961, (Fimbriaphyllia_ancora_NC_015641: 0.019307, Galaxea_fascicularis_NC_029696: 0.000058): 0.164351): 0.000005): 0.051651, ((Pseudosiderastrea_formosa_NC_026530: 0.000005, Pseudosiderastrea_tayamai_NC_026531: 0.000005): 0.037345, Siderastrea_radians_NC_008167: 0.037470): 0.051326): 0.000005, ((((((Dendrophyllia_arbuscula_NC_027590: 0.000005, Tubastraea_coccinea_NC_026025: 0.000005): 0.018982, ((Tubastraea_diaphana_MK959042: 0.000005, Tubastraea_tagusensis_NC_030352: 0.000005): 0.000005, Tubastraea_micranthus_MK959041: 0.000005): 0.000005): 0.000055, Dendrophyllia_cribrosa_NC_026026: 0.062429): 0.000005, Turbinaria_peltata_NC_024671: 0.039165): 0.061396, (((Goniopora_columna_NC_015643: 0.000005, Goniopora_djiboutiensis_NC_045931: 0.000005): 0.056675, Goniopora_lobata_MN795054: 0.000005): 0.075791, ((Porites_fontanesii_NC_037434: 0.000005, ((Porites_panamensis_NC_024182: 0.000005, Porites_sverdrupi_KU956960: 0.000005): 0.000005, Porites_porites_NC_008166: 0.000005): 0.000005): 0.000005, (Porites_harrisoni_NC_037435: 0.000005, ((Porites_lobata_NC_030186: 0.018186, Porites_okinawensis_NC_015644: 0.000005): 0.000005, (Porites_lutea_NC_029695: 0.000005, Porites_rus_NC_027526: 0.000005): 0.000005): 0.000005): 0.000005): 0.048599): 0.105425): 0.060127, Fungiacyathus_stephanus_NC_015640: 0.062449): 0.065787): 0.111379, Gardineria_hawaiiensis_NC_056290: 0.221901): 0.281117, (((((Amplexidiscus_fenestrafer_NC_027101: 0.035678, (Discosoma_sp__MH308003: 0.000005, (Rhodactis_indosinensis_NC_027103: 0.000005, Rhodactis_sp__CASIZ_171755_NC_008158: 0.000005): 0.000005): 0.000005): 0.000005, (Discosoma_nummiforme_NC_027100: 0.000005, ((Discosoma_sp__CASIZ_168915_NC_008071: 0.000005, Discosoma_sp__CASIZ_168916_NC_008072: 0.000005): 0.000005, Platyzoanthus_mussoides_NC_027104: 0.000056): 0.000005): 0.017586): 0.136541, (Pseudocorynactis_sp__SIO_KP938437: 0.219389, (Ricordea_florida_NC_008159: 0.016663, Ricordea_yuma_NC_027106: 0.000005): 0.146627): 0.010347): 0.065692, Corynactis_californica_NC_027102: 0.236356): 0.032772, Corallimorphus_profundus_KP938440: 0.045362): 0.000005): 0.452719, ((((((Astrangia_sp__JVK_2006_NC_008161: 0.152551, (((Colpophyllia_natans_NC_008162: 0.000005, Mussa_angulosa_NC_008163: 0.036424): 0.016503, ((((((Dipsastraea_favus_NC_046690: 0.000005, Dipsastraea_rotumana_NC_044074: 0.037955): 0.018966, Favites_abdita_NC_035879: 0.000005): 0.000005, Platygyra_carnosa_NC_020049: 0.000005): 0.000005, (Favites_pentagona_NC_034916: 0.059277, Hydnophora_exesa_NC_042795: 0.018921): 0.000005): 0.018638, ((Orbicella_annularis_NC_007224: 0.000005, Orbicella_faveolata_NC_007226: 0.000005): 0.000005, Orbicella_franksi_NC_007225: 0.000005): 0.038215): 0.000005, Echinophyllia_aspera_NC_040169: 0.000005): 0.022941): 0.080575, Plesiastrea_versipora_NC_042481: 0.083255): 0.015676): 0.097124, (Polycyathus_sp__MFL_2011_NC_015642: 0.082097, Psammocora_nierstraszi_MT576637: 0.018110): 0.033690): 0.139859, Madrepora_oculata_NC_018364: 1.569687): 0.000005, (Madracis_myriaster_NC_011160: 0.000150, ((Pocillopora_damicornis_NC_009797: 0.000005, Pocillopora_grandis_NC_009798: 0.021009): 0.024667, ((Seriatopora_caliendrum_NC_010245: 0.000054, Seriatopora_hystrix_NC_010244: 0.000005): 0.085284, Stylophora_pistillata_NC_011162: 0.000005): 0.017487): 0.116980): 0.556221): 0.806355, ((Desmophyllum_dianthus_NC_034275: 0.000005, Desmophyllum_pertusum_NC_015143: 0.000005): 0.000005, Solenosmilia_variabilis_NC_025472: 0.000005): 0.000421): 1.924254, Paraconotrochus_antarcticus_NC_056275: 1.538209): 0.092603): 0.000250, ((((((Antipathes_cf__dichotoma_NB_2020_MT318841: 0.000005, Stichopathes_abyssicola_MT318856: 0.000005): 0.066912, (Phanopathes_sp__NB_2020_MT318852: 0.151627, Stichopathes_sp__n__NB_2020_MT318857: 0.199774): 0.000005): 0.094522, ((Stichopathes_luetkeni_NC_018377: 0.000005, Stichopathes_sp__SCBUCN_8850_MZ157399: 0.000005): 0.000005, Stichopathes_sp__SCBUCN_8849_MZ157400: 0.018930): 0.187355): 0.000005, ((Myriopathes_japonica_NC_027667: 0.000005, Tanacetipathes_thamnea_NC_046843: 0.017894): 0.000005, Tylopathes_sp__n__NB_2020_MT318859: 0.036185): 0.161358): 0.110428, (((((((Bathypathes_sp__1_NB_2020_MT318844: 0.000005, Bathypathes_sp__n__2_NB_2020_MT318842: 0.000005): 0.000005, Stauropathes_arctica_MT318854: 0.000005): 0.000005, Bathypathes_sp__n__3_NB_2020_MT318843: 0.000055): 0.000055, Stauropathes_cf__punctata_NB_2020_MT318855: 0.000005): 0.000005, Telopathes_sp__NB_2020_MT318858: 0.000005): 0.000005, (Dendrobathypathes_sp__n__NB_2020_MT318845: 0.054699, ((Parantipathes_cf__hirondelle_NB_2020_MT318849: 0.000005, Parantipathes_hirondelle_MT318850: 0.000005): 0.000005, (Parantipathes_sp__NB_2020_MT318851: 0.000005, Sibopathes_cf__macrospina_NB_2020_MT318853: 0.000005): 0.000005): 0.018014): 0.000055): 0.172376, (Chrysopathes_formosa_NC_008411: 0.084220, Trissopathes_cf__tetracrada_NB_2020_MT318840: 0.017136): 0.074647): 0.091841): 0.023630, (Leiopathes_cf__glaberrima_NB_2020_MT318846: 0.000005, Leiopathes_expansa_MT318847: 0.000005): 0.022407): 0.185899): 0.000005, (Epizoanthus_illoricatus_NC_046474: 0.137559, (((Hydrozoanthus_gracilis_NC_046400: 0.015382, ((Neozoanthus_aff__uchina_AP_2020_MN873592: 0.015636, ((Zoanthus_cf__pulchellus_AP_2020_MN873599: 0.000001, ((Zoanthus_cf__sociatus_AP_2020_MN873600: 0.000001, Zoanthus_sociatus_NC_046476: 0.000001): 0.000001, Zoanthus_sp__AP_2020_MN873602: 0.000001): 0.000001): 0.000001, Zoanthus_sansibaricus_NC_035578: 0.000001): 0.031895): 0.000000, (Palythoa_heliodiscus_NC_035579: 0.000000, ((Palythoa_mizigama_NC_046403: 0.000000, Palythoa_mutuki_NC_046404: 0.015677): 0.000000, Sphenopus_marsupialis_NC_046406: 0.000000): 0.000000): 0.000059): 0.031685): 0.000000, ((Parazoanthus_elongatus_NC_046405: 0.032337, Parazoanthus_swiftii_NC_046475: 0.000000): 0.000000, Savalia_savaglia_DQ825686: 0.015840): 0.000059): 0.204962, (Microzoanthus_occultus_NC_046401: 3.025824, Nanozoanthus_harenaceus_NC_046402: 0.368880): 2.384375): 0.000005): 0.404169): 0.512980, (((((((Actinia_equina_NC_039929: 0.000005, Actinia_tenebrosa_NC_044902: 0.000005): 0.094132, (((Anemonia_manjano_NC_037178: 0.000005, Heteractis_aurora_NC_047219: 0.054132): 0.000005, (Anemonia_sulcata_NC_049065: 0.000005, Anemonia_viridis_NC_037177: 0.000005): 0.000056): 0.000005, Anthopleura_anjunae_NC_030274: 0.000005): 0.000005): 0.000005, Phymanthus_crucifer_NC_027614: 0.116274): 0.116000, ((((Bolocera_sp__BZ_2016_KU507297: 0.000001, (Bolocera_tuediae_NC_022470: 0.000001, Liponema_brevicorne_NC_047221: 0.000001): 0.000001): 0.018789, (Entacmaea_quadricolor_NC_049066: 0.018631, Isosicyonis_striata_KR051006: 0.089011): 0.000005): 0.099831, Stichodactyla_haddoni_MW760873: 0.099742): 0.000005, Epiactis_japonica_NC_047217: 0.012608): 0.123393): 0.000005, ((((Alicia_sansibarensis_NC_027610: 0.677637, (Haloclava_producta_NC_047218: 0.044578, Sagartia_ornata_NC_027615: 0.036236): 0.052977): 0.077957, (Diadumene_lineata_NC_045515: 0.226417, Metridium_senile_NC_000933: 0.042016): 0.028363): 0.086774, Paraphelliactis_xishaensis_MT997141: 0.074534): 0.148968, Antholoba_achates_NC_027611: 0.287258): 0.053678): 0.167193, Halcampoides_purpureus_NC_027612: 0.000131): 0.143800, Nematostella_sp__JVK_2006_NC_008164: 0.685612): 0.000424): 62.227594, (((((((Aurelia_aurita_NC_008446: 0.814180, (Aurelia_coerulea_NC_046792: 0.084703, Aurelia_limbata_NC_046691: 1.076327): 0.601711): 0.000892, (Aurelia_sp__3_sensu_Dawson_et_al___2005__LC005413: 0.397527, Aurelia_sp__4_sensu_Dawson_et_al___2005__LC005414: 0.979793): 0.369301): 1.290336, ((Nemopilema_nomurai_NC_035740: 0.388346, Rhopilema_esculentum_NC_035741: 1.931279): 0.570486, Stomolophus_sp__CG_2019_MK157198: 1.286080): 0.001634): 0.040654, Cassiopea_xamachana_NC_016466: 2.405549): 0.000149, (Chrysaora_pacifica_NC_046775: 0.000000, Chrysaora_quinquecirrha_NC_020459: 0.000117): 3.039015): 0.000805, ((((((Blackfordia_virginica_NC_053946: 1.580288, Eutima_sp__BMK_2020_MW066348: 1.041541): 0.000000, Laomedea_flexuosa_JN700945: 1.155932): 0.353450, (Clava_multicornis_NC_016465: 0.765133, (Turritopsis_dohrnii_NC_031213: 1.083090, Turritopsis_lata_NC_056128: 1.718925): 1.035411): 0.000399): 0.000001, Spirocodon_saltatrix_NC_049868: 0.317033): 0.052782, ((Hydra_oligactis_NC_010214: 3.700446, Hydra_sinensis_NC_021406: 1.451793): 0.000918, Nemalecium_lighti_MZ457217: 1.611893): 0.649522): 0.000267, (Craspedacusta_sowerbii_NC_018537: 62.136958, Cubaia_aphrodite_NC_016467: 2.337366): 0.000819): 0.298163): 0.000689, (Haliclystus_antarcticus_NC_030337: 2.862068, OU744455_1_OU744455_1__OU744455_1: 3.152981): 0.003335): 3.205730): 0.545672, Carijoa_riisei_NC_048963: 0.115985): 0.000206, (Sarcophyton_trocheliophorum_MK994517: 0.114976, (((Sinularia_acuta_MW987591: 0.000001, (Sinularia_maxima_MN485891: 0.000057, Sinularia_penghuensis_MW256412: 0.000001): 0.000001): 0.000094, Sinularia_cf__cruciata_GS_2016_NC_034318: 0.033631): 0.033295, (Sinularia_ceramensis_NC_044122: 0.000001, Sinularia_peculiaris_NC_018379: 0.000001): 0.016325): 0.076703): 0.034960): 0.021627, (((((((Antillogorgia_bipinnata_NC_008157: 0.072976, ((((Eugorgia_mutabilis_NC_035665: 0.016889, Leptogorgia_alba_NC_035669: 0.000001): 0.000001, (Leptogorgia_hebes_MN052675: 0.000001, Leptogorgia_virgulata_MK301586: 0.000001): 0.000001): 0.000001, Leptogorgia_sp__USNM_1437444_KY559412: 0.000001): 0.000005, Pacifigorgia_cairnsi_NC_035668: 0.050881): 0.016946): 0.000005, (((Leptogorgia_capverdensis_NC_035663: 0.000001, Leptogorgia_sarmentosa_NC_035670: 0.000058): 0.000001, Leptogorgia_gaini_KY559404: 0.053182): 0.000001, Leptogorgia_cf__palma_AP_2017_KY559406: 0.017180): 0.000001): 0.000001, (Eunicella_albicans_NC_035666: 0.038457, (Eunicella_cavolini_NC_035667: 0.017639, Eunicella_verrucosa_MW588805: 0.000005): 0.040519): 0.014806): 0.000001, (Calicogorgia_granulosa_NC_023345: 0.624543, Euplexaura_crassa_NC_020458: 0.017085): 0.000005): 0.000001, Muricea_crassa_NC_029697: 0.054143): 0.000001, ((((((Dendronephthya_castanea_NC_023343: 0.000001, Dendronephthya_mollis_NC_020456: 0.000001): 0.000001, Dendronephthya_gigantea_NC_013573: 0.000001): 0.000001, (Dendronephthya_putteri_NC_036022: 0.000001, Dendronephthya_suensoni_NC_022809: 0.000001): 0.000001): 0.089729, Scleronephthya_gracillimum_NC_023344: 0.017263): 0.000005, (Echinogorgia_complexa_NC_020457: 0.000005, Paramuricea_clavata_NC_034749: 0.071131): 0.090422): 0.000005, Trachythela_sp__YZ_2021_MW238423: 0.034957): 0.000001): 0.000001, (Incrustatus_comauensis_MT254531: 0.000058, Telestula_humilis_MT254527: 0.017219): 0.000001): 0.011815): 0.159429, Briareum_asbestinum_NC_008073: 0.038292): 0.000076, (Telestula_cf__batoni_AP_2020_MT254530: 0.017812, Telestula_septentrionalis_MT254532: 0.000005): 0.039293): 0.014301, (((Anthomastus_sp__USNM_1081145_KM015353: 0.017212, Anthomastus_sp__USNM_1171062_KM015352: 0.000004): 0.073519, (((Corallium_japonicum_AB595189: 0.000058, Corallium_rubrum_NC_022864: 0.000001): 0.017237, (Pleurocorallium_elatius_NC_022804: 0.000002, Pleurocorallium_konojoi_NC_015406: 0.000002): 0.035692): 0.018510, Sibogagorgia_cauliflora_NC_026193: 0.069379): 0.000064): 0.000005, Paraminabea_aldersladei_NC_018790: 0.132054): 0.052337): 0.000005, (((((((Anthoptilum_grandiflorum_NC_044086: 0.035911, Anthoptilum_sp__1_RH_2019_MK919656: 0.000005): 0.000005, (Umbellula_huxleyi_MK919668: 0.000005, Umbellula_sp__1_RH_2019_MK919669: 0.052842): 0.035767): 0.000005, Virgularia_mirabilis_NC_044091: 0.073761): 0.000005, (Funiculina_quadrangularis_NC_044078: 0.000058, ((Kophobelemnon_sp__1_RH_2019_MK919660: 0.017413, Kophobelemnon_sp__3_RH_2019_MK919661: 0.000005): 0.000002, Kophobelemnon_sp__4_RH_2019_MK919662: 0.000003): 0.017674): 0.000005): 0.000005, (Halipteris_cf__finmarchica_RH_2019_MK919659: 0.000092, Umbellula_sp__2_RH_2019_MK919670: 0.017404): 0.000001): 0.000005, ((((Distichoptilum_gracile_NC_044077: 0.035118, (Pennatula_cf__inflata_RH_2019_MK919666: 0.000001, Pennatula_grandis_NC_044088: 0.000001): 0.000005): 0.035539, ((Pennatula_aculeata_NC_044087: 0.000001, Pennatula_cf__aculeata_RH_2019_MK919664: 0.000001): 0.022089, Renilla_muelleri_NC_018378: 0.200818): 0.092954): 0.000001, Protoptilum_carpenteri_NC_044089: 0.034808): 0.000058, Stylatula_elongata_NC_018380: 0.035802): 0.035069): 0.000005, Junceella_fragilis_NC_024181: 0.134523): 0.000005): 0.000058, Heliopora_coerulea_NC_020375: 0.073822): 0.000003, ((Callogorgia_cf__gracilis_EE_2018_MH719202: 0.000058, (Plumarella_adhaerans_NC_046480: 0.000005, Plumarella_spinosa_NC_046465: 0.000005): 0.000004): 0.000005, Narella_hawaiiensis_NC_026192: 0.017168): 0.000004): 0.034781, Keratoisidinae_sp__BAL208_1_NC_010764: 0.034580);

**dN tree:**

(Acanella_arbuscula_NC_011016: 0.000000, ((((((((((((((((((((((((((((((Acropora_aculeus_KT001202: 0.005760, (Acropora_horrida_NC_022825: 0.000000, LC201842_1_LC201842_1__LC201842_1: 0.000000): 0.000000): 0.000000, LC201815_1_LC201815_1__LC201815_1: 0.000000): 0.000000, ((((Acropora_aspera_NC_022827: 0.000000, Acropora_muricata_NC_022824: 0.000000): 0.000000, LC201828_1_LC201828_1__LC201828_1: 0.000000): 0.000000, (((Acropora_digitifera_NC_022830: 0.000000, LC201849_1_LC201849_1__LC201849_1: 0.000000): 0.000000, Acropora_florida_NC_022828: 0.000000): 0.000000, Acropora_humilis_NC_022823: 0.000000): 0.000000): 0.000000, (Acropora_hyacinthus_NC_022826: 0.000000, LC201817_1_LC201817_1__LC201817_1: 0.000000): 0.000000): 0.005699): 0.000000, (Acropora_divaricata_NC_022832: 0.000000, (((Acropora_nasuta_NC_022831: 0.000000, Acropora_valida_NC_042742: 0.000000): 0.000000, LC201847_1_LC201847_1__LC201847_1: 0.000000): 0.000000, LC201820_1_LC201820_1__LC201820_1: 0.000000): 0.000002): 0.000000): 0.000000, Acropora_robusta_NC_022833: 0.000000): 0.000000, LC201813_1_LC201813_1__LC201813_1: 0.000002): 0.000000, ((Acropora_tenuis_NC_003522: 0.023450, Acropora_yongei_NC_022829: 0.000000): 0.005702, LC201816_1_LC201816_1__LC201816_1: 0.000000): 0.000000): 0.000004, (Isopora_palifera_NC_024091: 0.000000, Isopora_togianensis_NC_024089: 0.000000): 0.000000): 0.000002, ((Anacropora_matthai_NC_006898: 0.000000, Montipora_efflorescens_NC_040137: 0.000000): 0.000000, (Montipora_aequituberculata_NC_037359: 0.000000, Montipora_cactus_NC_006902: 0.000000): 0.000000): 0.000006): 0.000002, (Alveopora_japonica_NC_040136: 0.000000, Alveopora_sp__MFL_2014_KJ634271: 0.000000): 0.005830): 0.000004, (Astreopora_explanata_NC_024090: 0.000000, Astreopora_myriophthalma_NC_024092: 0.000000): 0.000003): 0.000006, ((Agaricia_humilis_NC_008160: 0.006376, Pavona_decussata_NC_026527: 0.006282): 0.018528, (Fimbriaphyllia_ancora_NC_015641: 0.000002, Galaxea_fascicularis_NC_029696: 0.005734): 0.011762): 0.000000): 0.000005, ((Pseudosiderastrea_formosa_NC_026530: 0.000000, Pseudosiderastrea_tayamai_NC_026531: 0.000000): 0.011804, Siderastrea_radians_NC_008167: 0.000004): 0.011728): 0.000000, ((((((Dendrophyllia_arbuscula_NC_027590: 0.000000, Tubastraea_coccinea_NC_026025: 0.000000): 0.000002, ((Tubastraea_diaphana_MK959042: 0.000000, Tubastraea_tagusensis_NC_030352: 0.000000): 0.000000, Tubastraea_micranthus_MK959041: 0.000000): 0.000000): 0.005533, Dendrophyllia_cribrosa_NC_026026: 0.000006): 0.000000, Turbinaria_peltata_NC_024671: 0.005581): 0.011244, (((Goniopora_columna_NC_015643: 0.000000, Goniopora_djiboutiensis_NC_045931: 0.000000): 0.000006, Goniopora_lobata_MN795054: 0.000000): 0.011888, ((Porites_fontanesii_NC_037434: 0.000000, ((Porites_panamensis_NC_024182: 0.000000, Porites_sverdrupi_KU956960: 0.000000): 0.000000, Porites_porites_NC_008166: 0.000000): 0.000000): 0.000000, (Porites_harrisoni_NC_037435: 0.000000, ((Porites_lobata_NC_030186: 0.000002, Porites_okinawensis_NC_015644: 0.000000): 0.000000, (Porites_lutea_NC_029695: 0.000000, Porites_rus_NC_027526: 0.000000): 0.000000): 0.000000): 0.000000): 0.000005): 0.011512): 0.000006, Fungiacyathus_stephanus_NC_015640: 0.011552): 0.005850): 0.016726, Gardineria_hawaiiensis_NC_056290: 0.016270): 0.037341, (((((Amplexidiscus_fenestrafer_NC_027101: 0.000004, (Discosoma_sp__MH308003: 0.000000, (Rhodactis_indosinensis_NC_027103: 0.000000, Rhodactis_sp__CASIZ_171755_NC_008158: 0.000000): 0.000000): 0.000000): 0.000000, (Discosoma_nummiforme_NC_027100: 0.000000, ((Discosoma_sp__CASIZ_168915_NC_008071: 0.000000, Discosoma_sp__CASIZ_168916_NC_008072: 0.000000): 0.000000, Platyzoanthus_mussoides_NC_027104: 0.005829): 0.000000): 0.005867): 0.006022, (Pseudocorynactis_sp__SIO_KP938437: 0.018370, (Ricordea_florida_NC_008159: 0.000002, Ricordea_yuma_NC_027106: 0.000000): 0.000015): 0.000001): 0.006805, Corynactis_californica_NC_027102: 0.045070): 0.000003, Corallimorphus_profundus_KP938440: 0.026213): 0.000000): 0.023090, ((((((Astrangia_sp__JVK_2006_NC_008161: 0.005321, (((Colpophyllia_natans_NC_008162: 0.000000, Mussa_angulosa_NC_008163: 0.000004): 0.000002, ((((((Dipsastraea_favus_NC_046690: 0.000000, Dipsastraea_rotumana_NC_044074: 0.000004): 0.000002, Favites_abdita_NC_035879: 0.000000): 0.000000, Platygyra_carnosa_NC_020049: 0.000000): 0.000000, (Favites_pentagona_NC_034916: 0.005276, Hydnophora_exesa_NC_042795: 0.000002): 0.000000): 0.000002, ((Orbicella_annularis_NC_007224: 0.000000, Orbicella_faveolata_NC_007226: 0.000000): 0.000000, Orbicella_franksi_NC_007225: 0.000000): 0.005389): 0.000000, Echinophyllia_aspera_NC_040169: 0.000000): 0.005337): 0.010845, Plesiastrea_versipora_NC_042481: 0.005436): 0.000002): 0.000010, (Polycyathus_sp__MFL_2011_NC_015642: 0.000008, Psammocora_nierstraszi_MT576637: 0.000002): 0.016769): 0.011771, Madrepora_oculata_NC_018364: 0.059611): 0.000000, (Madracis_myriaster_NC_011160: 0.005642, ((Pocillopora_damicornis_NC_009797: 0.000000, Pocillopora_grandis_NC_009798: 0.000002): 0.000002, ((Seriatopora_caliendrum_NC_010245: 0.005241, Seriatopora_hystrix_NC_010244: 0.000000): 0.000009, Stylophora_pistillata_NC_011162: 0.000000): 0.000002): 0.009991): 0.092340): 0.049035, ((Desmophyllum_dianthus_NC_034275: 0.000000, Desmophyllum_pertusum_NC_015143: 0.000000): 0.000000, Solenosmilia_variabilis_NC_025472: 0.000000): 0.025848): 0.153107, Paraconotrochus_antarcticus_NC_056275: 0.146992): 0.048788): 0.014288, ((((((Antipathes_cf__dichotoma_NB_2020_MT318841: 0.000000, Stichopathes_abyssicola_MT318856: 0.000000): 0.005626, (Phanopathes_sp__NB_2020_MT318852: 0.011182, Stichopathes_sp__n__NB_2020_MT318857: 0.016693): 0.000000): 0.005536, ((Stichopathes_luetkeni_NC_018377: 0.000000, Stichopathes_sp__SCBUCN_8850_MZ157399: 0.000000): 0.000000, Stichopathes_sp__SCBUCN_8849_MZ157400: 0.000002): 0.005453): 0.000000, ((Myriopathes_japonica_NC_027667: 0.000000, Tanacetipathes_thamnea_NC_046843: 0.000002): 0.000000, Tylopathes_sp__n__NB_2020_MT318859: 0.005683): 0.011150): 0.000011, (((((((Bathypathes_sp__1_NB_2020_MT318844: 0.000000, Bathypathes_sp__n__2_NB_2020_MT318842: 0.000000): 0.000000, Stauropathes_arctica_MT318854: 0.000000): 0.000000, Bathypathes_sp__n__3_NB_2020_MT318843: 0.005530): 0.005579, Stauropathes_cf__punctata_NB_2020_MT318855: 0.000000): 0.000000, Telopathes_sp__NB_2020_MT318858: 0.000000): 0.000000, (Dendrobathypathes_sp__n__NB_2020_MT318845: 0.000005, ((Parantipathes_cf__hirondelle_NB_2020_MT318849: 0.000000, Parantipathes_hirondelle_MT318850: 0.000000): 0.000000, (Parantipathes_sp__NB_2020_MT318851: 0.000000, Sibopathes_cf__macrospina_NB_2020_MT318853: 0.000000): 0.000000): 0.000002): 0.005626): 0.006100, (Chrysopathes_formosa_NC_008411: 0.000008, Trissopathes_cf__tetracrada_NB_2020_MT318840: 0.000002): 0.005567): 0.000009): 0.005615, (Leiopathes_cf__glaberrima_NB_2020_MT318846: 0.000000, Leiopathes_expansa_MT318847: 0.000000): 0.000002): 0.017619): 0.000000, (Epizoanthus_illoricatus_NC_046474: 0.006266, (((Hydrozoanthus_gracilis_NC_046400: 0.000002, ((Neozoanthus_aff__uchina_AP_2020_MN873592: 0.000002, ((Zoanthus_cf__pulchellus_AP_2020_MN873599: 0.000002, ((Zoanthus_cf__sociatus_AP_2020_MN873600: 0.000002, Zoanthus_sociatus_NC_046476: 0.000002): 0.000002, Zoanthus_sp__AP_2020_MN873602: 0.000002): 0.000002): 0.000002, Zoanthus_sansibaricus_NC_035578: 0.000002): 0.000003): 0.000002, (Palythoa_heliodiscus_NC_035579: 0.000002, ((Palythoa_mizigama_NC_046403: 0.000002, Palythoa_mutuki_NC_046404: 0.000002): 0.000002, Sphenopus_marsupialis_NC_046406: 0.000002): 0.000002): 0.006691): 0.000003): 0.000002, ((Parazoanthus_elongatus_NC_046405: 0.000003, Parazoanthus_swiftii_NC_046475: 0.000002): 0.000002, Savalia_savaglia_DQ825686: 0.000002): 0.006676): 0.006872, (Microzoanthus_occultus_NC_046401: 0.037087, Nanozoanthus_harenaceus_NC_046402: 0.052599): 0.037479): 0.000000): 0.012169): 0.011978, (((((((Actinia_equina_NC_039929: 0.000000, Actinia_tenebrosa_NC_044902: 0.000000): 0.000009, (((Anemonia_manjano_NC_037178: 0.000000, Heteractis_aurora_NC_047219: 0.005935): 0.000000, (Anemonia_sulcata_NC_049065: 0.000000, Anemonia_viridis_NC_037177: 0.000000): 0.005953): 0.000000, Anthopleura_anjunae_NC_030274: 0.000000): 0.000000): 0.000000, Phymanthus_crucifer_NC_027614: 0.005878): 0.000012, ((((Bolocera_sp__BZ_2016_KU507297: 0.000001, (Bolocera_tuediae_NC_022470: 0.000001, Liponema_brevicorne_NC_047221: 0.000001): 0.000001): 0.000002, (Entacmaea_quadricolor_NC_049066: 0.012783, Isosicyonis_striata_KR051006: 0.000009): 0.000000): 0.000010, Stichodactyla_haddoni_MW760873: 0.000010): 0.000000, Epiactis_japonica_NC_047217: 0.018571): 0.000012): 0.000000, ((((Alicia_sansibarensis_NC_027610: 0.029604, (Haloclava_producta_NC_047218: 0.000004, Sagartia_ornata_NC_027615: 0.000004): 0.012254): 0.000008, (Diadumene_lineata_NC_045515: 0.006068, Metridium_senile_NC_000933: 0.018457): 0.012189): 0.000009, Paraphelliactis_xishaensis_MT997141: 0.000007): 0.012492, Antholoba_achates_NC_027611: 0.012039): 0.000005): 0.006387, Halcampoides_purpureus_NC_027612: 0.006346): 0.011357, Nematostella_sp__JVK_2006_NC_008164: 0.027311): 0.013421): 0.085482, (((((((Aurelia_aurita_NC_008446: 0.048053, (Aurelia_coerulea_NC_046792: 0.016054, Aurelia_limbata_NC_046691: 0.011652): 0.008711): 0.025273, (Aurelia_sp__3_sensu_Dawson_et_al___2005__LC005413: 0.023464, Aurelia_sp__4_sensu_Dawson_et_al___2005__LC005414: 0.041505): 0.008177): 0.016496, ((Nemopilema_nomurai_NC_035740: 0.052434, Rhopilema_esculentum_NC_035741: 0.027491): 0.017120, Stomolophus_sp__CG_2019_MK157198: 0.024257): 0.039015): 0.022016, Cassiopea_xamachana_NC_016466: 0.111137): 0.007317, (Chrysaora_pacifica_NC_046775: 0.000002, Chrysaora_quinquecirrha_NC_020459: 0.017584): 0.106808): 0.118673, ((((((Blackfordia_virginica_NC_053946: 0.047743, Eutima_sp__BMK_2020_MW066348: 0.061666): 0.000002, Laomedea_flexuosa_JN700945: 0.119134): 0.067928, (Clava_multicornis_NC_016465: 0.045257, (Turritopsis_dohrnii_NC_031213: 0.045774, Turritopsis_lata_NC_056128: 0.028165): 0.103926): 0.026726): 0.000002, Spirocodon_saltatrix_NC_049868: 0.104307): 0.000005, ((Hydra_oligactis_NC_010214: 0.055460, Hydra_sinensis_NC_021406: 0.104337): 0.061977, Nemalecium_lighti_MZ457217: 0.418655): 0.142429): 0.017674, (Craspedacusta_sowerbii_NC_018537: 0.118467, Cubaia_aphrodite_NC_016467: 0.111470): 0.056495): 0.036242): 0.034127, (Haliclystus_antarcticus_NC_030337: 0.000288, OU744455_1_OU744455_1__OU744455_1: 3.069334): 0.432722): 0.030405): 0.150106, Carijoa_riisei_NC_048963: 0.000012): 0.006277, (Sarcophyton_trocheliophorum_MK994517: 0.006293, (((Sinularia_acuta_MW987591: 0.000002, (Sinularia_maxima_MN485891: 0.006377, Sinularia_penghuensis_MW256412: 0.000002): 0.000002): 0.012820, Sinularia_cf__cruciata_GS_2016_NC_034318: 0.000003): 0.000003, (Sinularia_ceramensis_NC_044122: 0.000002, Sinularia_peculiaris_NC_018379: 0.000002): 0.006442): 0.006373): 0.000003): 0.000002, (((((((Antillogorgia_bipinnata_NC_008157: 0.006354, ((((Eugorgia_mutabilis_NC_035665: 0.006319, Leptogorgia_alba_NC_035669: 0.000002): 0.000002, (Leptogorgia_hebes_MN052675: 0.000002, Leptogorgia_virgulata_MK301586: 0.000002): 0.000002): 0.000002, Leptogorgia_sp__USNM_1437444_KY559412: 0.000002): 0.000000, Pacifigorgia_cairnsi_NC_035668: 0.000005): 0.000002): 0.000000, (((Leptogorgia_capverdensis_NC_035663: 0.000001, Leptogorgia_sarmentosa_NC_035670: 0.006285): 0.000001, Leptogorgia_gaini_KY559404: 0.000005): 0.000001, Leptogorgia_cf__palma_AP_2017_KY559406: 0.000002): 0.000001): 0.000001, (Eunicella_albicans_NC_035666: 0.000004, (Eunicella_cavolini_NC_035667: 0.000002, Eunicella_verrucosa_MW588805: 0.000000): 0.000004): 0.000001): 0.000001, (Calicogorgia_granulosa_NC_023345: 0.032581, Euplexaura_crassa_NC_020458: 0.006279): 0.000000): 0.000001, Muricea_crassa_NC_029697: 0.006181): 0.000001, ((((((Dendronephthya_castanea_NC_023343: 0.000002, Dendronephthya_mollis_NC_020456: 0.000002): 0.000002, Dendronephthya_gigantea_NC_013573: 0.000002): 0.000002, (Dendronephthya_putteri_NC_036022: 0.000002, Dendronephthya_suensoni_NC_022809: 0.000002): 0.000002): 0.006337, Scleronephthya_gracillimum_NC_023344: 0.000002): 0.000000, (Echinogorgia_complexa_NC_020457: 0.000000, Paramuricea_clavata_NC_034749: 0.000007): 0.000009): 0.000000, Trachythela_sp__YZ_2021_MW238423: 0.000003): 0.000001): 0.000001, (Incrustatus_comauensis_MT254531: 0.006273, Telestula_humilis_MT254527: 0.000002): 0.000001): 0.000001): 0.006382, Briareum_asbestinum_NC_008073: 0.012568): 0.006335, (Telestula_cf__batoni_AP_2020_MT254530: 0.000002, Telestula_septentrionalis_MT254532: 0.000000): 0.000004): 0.000001, (((Anthomastus_sp__USNM_1081145_KM015353: 0.000002, Anthomastus_sp__USNM_1171062_KM015352: 0.000000): 0.000007, (((Corallium_japonicum_AB595189: 0.006352, Corallium_rubrum_NC_022864: 0.000002): 0.000002, (Pleurocorallium_elatius_NC_022804: 0.000001, Pleurocorallium_konojoi_NC_015406: 0.000001): 0.006341): 0.000002, Sibogagorgia_cauliflora_NC_026193: 0.026102): 0.006286): 0.000000, Paraminabea_aldersladei_NC_018790: 0.018782): 0.006261): 0.000000, (((((((Anthoptilum_grandiflorum_NC_044086: 0.000004, Anthoptilum_sp__1_RH_2019_MK919656: 0.000000): 0.000000, (Umbellula_huxleyi_MK919668: 0.000000, Umbellula_sp__1_RH_2019_MK919669: 0.012698): 0.000004): 0.000000, Virgularia_mirabilis_NC_044091: 0.012347): 0.000000, (Funiculina_quadrangularis_NC_044078: 0.006191, ((Kophobelemnon_sp__1_RH_2019_MK919660: 0.000002, Kophobelemnon_sp__3_RH_2019_MK919661: 0.000000): 0.000001, Kophobelemnon_sp__4_RH_2019_MK919662: 0.000001): 0.000002): 0.000000): 0.000000, (Halipteris_cf__finmarchica_RH_2019_MK919659: 0.012497, Umbellula_sp__2_RH_2019_MK919670: 0.006258): 0.000001): 0.000000, ((((Distichoptilum_gracile_NC_044077: 0.000004, (Pennatula_cf__inflata_RH_2019_MK919666: 0.000001, Pennatula_grandis_NC_044088: 0.000001): 0.000000): 0.000004, ((Pennatula_aculeata_NC_044087: 0.000002, Pennatula_cf__aculeata_RH_2019_MK919664: 0.000002): 0.006255, Renilla_muelleri_NC_018378: 0.012803): 0.006395): 0.000002, Protoptilum_carpenteri_NC_044089: 0.000003): 0.006203, Stylatula_elongata_NC_018380: 0.000004): 0.000004): 0.000000, Junceella_fragilis_NC_024181: 0.012501): 0.000000): 0.006207, Heliopora_coerulea_NC_020375: 0.000007): 0.000001, ((Callogorgia_cf__gracilis_EE_2018_MH719202: 0.006231, (Plumarella_adhaerans_NC_046480: 0.000000, Plumarella_spinosa_NC_046465: 0.000000): 0.000000): 0.000000, Narella_hawaiiensis_NC_026192: 0.000002): 0.000000): 0.006292, Keratoisidinae_sp__BAL208_1_NC_010764: 0.000003);

**w ratios as labels for TreeView:**

(Acanella_arbuscula_NC_011016 #0.000100001 , ((((((((((((((((((((((((((((((Acropora_aculeus_KT001202 #0.310255 , (Acropora_horrida_NC_022825 #0.0001 , LC201842_1_LC201842_1__LC201842_1 #0.0001 ) #0.0001 ) #0.0001 , LC201815_1_LC201815_1__LC201815_1 #0.0001 ) #0.0001 , ((((Acropora_aspera_NC_022827 #0.0001 , Acropora_muricata_NC_022824 #0.0001 ) #0.0001 , LC201828_1_LC201828_1__LC201828_1 #0.0001 ) #0.0001 , (((Acropora_digitifera_NC_022830 #0.0001 , LC201849_1_LC201849_1__LC201849_1 #0.0001 ) #0.0001 , Acropora_florida_NC_022828 #0.0001 ) #0.0001 , Acropora_humilis_NC_022823 #0.0001 ) #0.0001 ) #0.0001 , (Acropora_hyacinthus_NC_022826 #0.0001 , LC201817_1_LC201817_1__LC201817_1 #0.0001 ) #0.0001 ) #102.759 ) #0.0001 , (Acropora_divaricata_NC_022832 #0.0001 , (((Acropora_nasuta_NC_022831 #0.0001 , Acropora_valida_NC_042742 #0.0001 ) #0.0001 , LC201847_1_LC201847_1__LC201847_1 #0.0001 ) #0.0001 , LC201820_1_LC201820_1__LC201820_1 #0.0001 ) #0.0001 ) #0.0001 ) #0.0001 , Acropora_robusta_NC_022833 #0.0001 ) #0.0001 , LC201813_1_LC201813_1__LC201813_1 #0.0001 ) #0.0001 , ((Acropora_tenuis_NC_003522 #0.610806 , Acropora_yongei_NC_022829 #0.0001 ) #102.513 , LC201816_1_LC201816_1__LC201816_1 #0.0001 ) #0.0001 ) #0.0001 , (Isopora_palifera_NC_024091 #0.0001 , Isopora_togianensis_NC_024089 #0.0001 ) #0.0001 ) #0.0001 , ((Anacropora_matthai_NC_006898 #0.0001 , Montipora_efflorescens_NC_040137 #0.0001 ) #0.0001 , (Montipora_aequituberculata_NC_037359 #0.0001 , Montipora_cactus_NC_006902 #0.0001 ) #0.0001 ) #0.0001 ) #0.0001 , (Alveopora_japonica_NC_040136 #0.0001 , Alveopora_sp__MFL_2014_KJ634271 #0.0001 ) #0.100244 ) #0.0001 , (Astreopora_explanata_NC_024090 #0.0001 , Astreopora_myriophthalma_NC_024092 #0.0001 ) #0.0001 ) #0.0001 , ((Agaricia_humilis_NC_008160 #0.115709 , Pavona_decussata_NC_026527 #0.0862468 ) #0.0785228 , (Fimbriaphyllia_ancora_NC_015641 #0.0001 , Galaxea_fascicularis_NC_029696 #98.6597 ) #0.0715674 ) #0.0001 ) #0.0001 , ((Pseudosiderastrea_formosa_NC_026530 #0.0001 , Pseudosiderastrea_tayamai_NC_026531 #0.0001 ) #0.316068 , Siderastrea_radians_NC_008167 #0.0001 ) #0.22849 ) #0.0001 , ((((((Dendrophyllia_arbuscula_NC_027590 #0.0001 , Tubastraea_coccinea_NC_026025 #0.0001 ) #0.0001 , ((Tubastraea_diaphana_MK959042 #0.0001 , Tubastraea_tagusensis_NC_030352 #0.0001 ) #0.0001 , Tubastraea_micranthus_MK959041 #0.0001 ) #0.0001 ) #99.8638 , Dendrophyllia_cribrosa_NC_026026 #0.0001 ) #0.0001 , Turbinaria_peltata_NC_024671 #0.1425 ) #0.183144 , (((Goniopora_columna_NC_015643 #0.0001 , Goniopora_djiboutiensis_NC_045931 #0.0001 ) #0.0001 , Goniopora_lobata_MN795054 #0.0001 ) #0.156857 , ((Porites_fontanesii_NC_037434 #0.0001 , ((Porites_panamensis_NC_024182 #0.0001 , Porites_sverdrupi_KU956960 #0.0001 ) #0.0001 , Porites_porites_NC_008166 #0.0001 ) #0.0001 ) #0.0001 , (Porites_harrisoni_NC_037435 #0.0001 , ((Porites_lobata_NC_030186 #0.0001 , Porites_okinawensis_NC_015644 #0.0001 ) #0.0001 , (Porites_lutea_NC_029695 #0.0001 , Porites_rus_NC_027526 #0.0001 ) #0.0001 ) #0.0001 ) #0.0001 ) #0.0001 ) #0.109197 ) #0.0001 , Fungiacyathus_stephanus_NC_015640 #0.184982 ) #0.0889201 ) #0.150171 , Gardineria_hawaiiensis_NC_056290 #0.0733199 ) #0.132832 , (((((Amplexidiscus_fenestrafer_NC_027101 #0.0001 , (Discosoma_sp__MH308003 #0.0001 , (Rhodactis_indosinensis_NC_027103 #0.0001 , Rhodactis_sp__CASIZ_171755_NC_008158 #0.0001 ) #0.0001 ) #0.0001 ) #0.0001 , (Discosoma_nummiforme_NC_027100 #0.0001 , ((Discosoma_sp__CASIZ_168915_NC_008071 #0.0001 , Discosoma_sp__CASIZ_168916_NC_008072 #0.0001 ) #0.0001 , Platyzoanthus_mussoides_NC_027104 #104.751 ) #0.0001 ) #0.333603 ) #0.0441023 , (Pseudocorynactis_sp__SIO_KP938437 #0.0837325 , (Ricordea_florida_NC_008159 #0.0001 , Ricordea_yuma_NC_027106 #0.0001 ) #0.0001 ) #0.0001 ) #0.103584 , Corynactis_californica_NC_027102 #0.190686 ) #0.0001 , Corallimorphus_profundus_KP938440 #0.577871 ) #0.0001 ) #0.0510021 , ((((((Astrangia_sp__JVK_2006_NC_008161 #0.0348799 , (((Colpophyllia_natans_NC_008162 #0.0001 , Mussa_angulosa_NC_008163 #0.0001 ) #0.0001 , ((((((Dipsastraea_favus_NC_046690 #0.0001 , Dipsastraea_rotumana_NC_044074 #0.0001 ) #0.0001 , Favites_abdita_NC_035879 #0.0001 ) #0.0001 , Platygyra_carnosa_NC_020049 #0.0001 ) #0.0001 , (Favites_pentagona_NC_034916 #0.0890041 , Hydnophora_exesa_NC_042795 #0.0001 ) #0.0001 ) #0.0001 , ((Orbicella_annularis_NC_007224 #0.0001 , Orbicella_faveolata_NC_007226 #0.0001 ) #0.0001 , Orbicella_franksi_NC_007225 #0.0001 ) #0.141026 ) #0.0001 , Echinophyllia_aspera_NC_040169 #0.0001 ) #0.232624 ) #0.134589 , Plesiastrea_versipora_NC_042481 #0.0652929 ) #0.0001 ) #0.0001 , (Polycyathus_sp__MFL_2011_NC_015642 #0.0001 , Psammocora_nierstraszi_MT576637 #0.0001 ) #0.49773 ) #0.0841616 , Madrepora_oculata_NC_018364 #0.0379763 ) #0.0001 , (Madracis_myriaster_NC_011160 #37.6192 , ((Pocillopora_damicornis_NC_009797 #0.0001 , Pocillopora_grandis_NC_009798 #0.0001 ) #0.0001 , ((Seriatopora_caliendrum_NC_010245 #97.7654 , Seriatopora_hystrix_NC_010244 #0.0001 ) #0.0001 , Stylophora_pistillata_NC_011162 #0.0001 ) #0.0001 ) #0.0854081 ) #0.166014 ) #0.0608108 , ((Desmophyllum_dianthus_NC_034275 #0.0001 , Desmophyllum_pertusum_NC_015143 #0.0001 ) #0.0001 , Solenosmilia_variabilis_NC_025472 #0.0001 ) #61.4392 ) #0.0795672 , Paraconotrochus_antarcticus_NC_056275 #0.0955604 ) #0.526852 ) #57.2128 , ((((((Antipathes_cf__dichotoma_NB_2020_MT318841 #0.0001 , Stichopathes_abyssicola_MT318856 #0.0001 ) #0.0840734 , (Phanopathes_sp__NB_2020_MT318852 #0.0737496 , Stichopathes_sp__n__NB_2020_MT318857 #0.0835572 ) #0.0001 ) #0.058564 , ((Stichopathes_luetkeni_NC_018377 #0.0001 , Stichopathes_sp__SCBUCN_8850_MZ157399 #0.0001 ) #0.0001 , Stichopathes_sp__SCBUCN_8849_MZ157400 #0.0001 ) #0.0291031 ) #0.0001 , ((Myriopathes_japonica_NC_027667 #0.0001 , Tanacetipathes_thamnea_NC_046843 #0.0001 ) #0.0001 , Tylopathes_sp__n__NB_2020_MT318859 #0.157054 ) #0.0690998 ) #0.0001 , (((((((Bathypathes_sp__1_NB_2020_MT318844 #0.0001 , Bathypathes_sp__n__2_NB_2020_MT318842 #0.0001 ) #0.0001 , Stauropathes_arctica_MT318854 #0.0001 ) #0.0001 , Bathypathes_sp__n__3_NB_2020_MT318843 #101.399 ) #101.979 , Stauropathes_cf__punctata_NB_2020_MT318855 #0.0001 ) #0.0001 , Telopathes_sp__NB_2020_MT318858 #0.0001 ) #0.0001 , (Dendrobathypathes_sp__n__NB_2020_MT318845 #0.0001 , ((Parantipathes_cf__hirondelle_NB_2020_MT318849 #0.0001 , Parantipathes_hirondelle_MT318850 #0.0001 ) #0.0001 , (Parantipathes_sp__NB_2020_MT318851 #0.0001 , Sibopathes_cf__macrospina_NB_2020_MT318853 #0.0001 ) #0.0001 ) #0.0001 ) #101.764 ) #0.0353885 , (Chrysopathes_formosa_NC_008411 #0.0001 , Trissopathes_cf__tetracrada_NB_2020_MT318840 #0.0001 ) #0.074576 ) #0.0001 ) #0.237624 , (Leiopathes_cf__glaberrima_NB_2020_MT318846 #0.0001 , Leiopathes_expansa_MT318847 #0.0001 ) #0.0001 ) #0.0947784 ) #0.0001 , (Epizoanthus_illoricatus_NC_046474 #0.0455485 , (((Hydrozoanthus_gracilis_NC_046400 #0.0001 , ((Neozoanthus_aff__uchina_AP_2020_MN873592 #0.0001 , ((Zoanthus_cf__pulchellus_AP_2020_MN873599 #2.99193 , ((Zoanthus_cf__sociatus_AP_2020_MN873600 #2.97786 , Zoanthus_sociatus_NC_046476 #3.02575 ) #3.03553 , Zoanthus_sp__AP_2020_MN873602 #3.00204 ) #3.01872 ) #3.08555 , Zoanthus_sansibaricus_NC_035578 #3.06219 ) #0.0001 ) #3.30299 , (Palythoa_heliodiscus_NC_035579 #3.43857 , ((Palythoa_mizigama_NC_046403 #3.47308 , Palythoa_mutuki_NC_046404 #0.0001 ) #3.42997 , Sphenopus_marsupialis_NC_046406 #3.45509 ) #3.43129 ) #113.986 ) #0.0001 ) #3.70888 , ((Parazoanthus_elongatus_NC_046405 #0.0001 , Parazoanthus_swiftii_NC_046475 #3.55728 ) #3.52937 , Savalia_savaglia_DQ825686 #0.0001 ) #112.338 ) #0.0335264 , (Microzoanthus_occultus_NC_046401 #0.0122569 , Nanozoanthus_harenaceus_NC_046402 #0.142591 ) #0.0157186 ) #0.0001 ) #0.0301097 ) #0.0233501 , (((((((Actinia_equina_NC_039929 #0.0001 , Actinia_tenebrosa_NC_044902 #0.0001 ) #0.0001 , (((Anemonia_manjano_NC_037178 #0.0001 , Heteractis_aurora_NC_047219 #0.10964 ) #0.0001 , (Anemonia_sulcata_NC_049065 #0.0001 , Anemonia_viridis_NC_037177 #0.0001 ) #105.797 ) #0.0001 , Anthopleura_anjunae_NC_030274 #0.0001 ) #0.0001 ) #0.0001 , Phymanthus_crucifer_NC_027614 #0.0505555 ) #0.0001 , ((((Bolocera_sp__BZ_2016_KU507297 #1.44796 , (Bolocera_tuediae_NC_022470 #1.51429 , Liponema_brevicorne_NC_047221 #1.5091 ) #1.54707 ) #0.0001 , (Entacmaea_quadricolor_NC_049066 #0.686123 , Isosicyonis_striata_KR051006 #0.0001 ) #0.0001 ) #0.0001 , Stichodactyla_haddoni_MW760873 #0.0001 ) #0.0001 , Epiactis_japonica_NC_047217 #1.47298 ) #0.0001 ) #0.0001 , ((((Alicia_sansibarensis_NC_027610 #0.0436875 , (Haloclava_producta_NC_047218 #0.0001 , Sagartia_ornata_NC_027615 #0.0001 ) #0.231306 ) #0.0001 , (Diadumene_lineata_NC_045515 #0.0268012 , Metridium_senile_NC_000933 #0.439285 ) #0.429752 ) #0.0001 , Paraphelliactis_xishaensis_MT997141 #0.0001 ) #0.083855 , Antholoba_achates_NC_027611 #0.0419092 ) #0.0001 ) #0.0382005 , Halcampoides_purpureus_NC_027612 #48.3971 ) #0.0789794 , Nematostella_sp__JVK_2006_NC_008164 #0.0398348 ) #31.6278 ) #0.0013737 , (((((((Aurelia_aurita_NC_008446 #0.0590198 , (Aurelia_coerulea_NC_046792 #0.189538 , Aurelia_limbata_NC_046691 #0.0108259 ) #0.0144767 ) #28.3187 , (Aurelia_sp__3_sensu_Dawson_et_al___2005__LC005413 #0.0590258 , Aurelia_sp__4_sensu_Dawson_et_al___2005__LC005414 #0.0423614 ) #0.0221423 ) #0.0127839 , ((Nemopilema_nomurai_NC_035740 #0.13502 , Rhopilema_esculentum_NC_035741 #0.0142348 ) #0.03001 , Stomolophus_sp__CG_2019_MK157198 #0.018861 ) #23.8798 ) #0.541559 , Cassiopea_xamachana_NC_016466 #0.0462003 ) #49.0644 , (Chrysaora_pacifica_NC_046775 #3.7091 , Chrysaora_quinquecirrha_NC_020459 #149.813 ) #0.0351456 ) #147.431 , ((((((Blackfordia_virginica_NC_053946 #0.0302117 , Eutima_sp__BMK_2020_MW066348 #0.0592067 ) #8.47787 , Laomedea_flexuosa_JN700945 #0.103064 ) #0.192186 , (Clava_multicornis_NC_016465 #0.0591487 , (Turritopsis_dohrnii_NC_031213 #0.0422625 , Turritopsis_lata_NC_056128 #0.0163853 ) #0.100371 ) #67.0021 ) #1.90337 , Spirocodon_saltatrix_NC_049868 #0.32901 ) #0.0001 , ((Hydra_oligactis_NC_010214 #0.0149873 , Hydra_sinensis_NC_021406 #0.0718675 ) #67.5239 , Nemalecium_lighti_MZ457217 #0.259729 ) #0.219282 ) #66.2216 , (Craspedacusta_sowerbii_NC_018537 #0.00190655 , Cubaia_aphrodite_NC_016467 #0.0476904 ) #68.945 ) #0.121552 ) #49.507 , (Haliclystus_antarcticus_NC_030337 #0.000100559 , OU744455_1_OU744455_1__OU744455_1 #0.973471 ) #129.744 ) #0.00948463 ) #0.275086 , Carijoa_riisei_NC_048963 #0.0001 ) #30.4614 , (Sarcophyton_trocheliophorum_MK994517 #0.0547341 , (((Sinularia_acuta_MW987591 #2.82435 , (Sinularia_maxima_MN485891 #111.34 , Sinularia_penghuensis_MW256412 #2.86152 ) #2.79973 ) #136.945 , Sinularia_cf__cruciata_GS_2016_NC_034318 #0.0001 ) #0.0001 , (Sinularia_ceramensis_NC_044122 #2.75309 , Sinularia_peculiaris_NC_018379 #2.83708 ) #0.394627 ) #0.0830829 ) #0.0001 ) #0.0001 , (((((((Antillogorgia_bipinnata_NC_008157 #0.0870661 , ((((Eugorgia_mutabilis_NC_035665 #0.374142 , Leptogorgia_alba_NC_035669 #2.03198 ) #2.20424 , (Leptogorgia_hebes_MN052675 #2.09863 , Leptogorgia_virgulata_MK301586 #2.09485 ) #2.16888 ) #2.21622 , Leptogorgia_sp__USNM_1437444_KY559412 #2.25398 ) #0.0001 , Pacifigorgia_cairnsi_NC_035668 #0.0001 ) #0.0001 ) #0.0001 , (((Leptogorgia_capverdensis_NC_035663 #1.07474 , Leptogorgia_sarmentosa_NC_035670 #108.476 ) #1.05907 , Leptogorgia_gaini_KY559404 #0.0001 ) #1.07165 , Leptogorgia_cf__palma_AP_2017_KY559406 #0.0001 ) #1.05709 ) #1.11753 , (Eunicella_albicans_NC_035666 #0.0001 , (Eunicella_cavolini_NC_035667 #0.0001 , Eunicella_verrucosa_MW588805 #0.0001 ) #0.0001 ) #0.0001 ) #1.26116 , (Calicogorgia_granulosa_NC_023345 #0.052168 , Euplexaura_crassa_NC_020458 #0.367497 ) #0.0001 ) #1.05628 , Muricea_crassa_NC_029697 #0.114164 ) #1.06152 , ((((((Dendronephthya_castanea_NC_023343 #2.06612 , Dendronephthya_mollis_NC_020456 #2.04875 ) #2.11 , Dendronephthya_gigantea_NC_013573 #2.06752 ) #2.10194 , (Dendronephthya_putteri_NC_036022 #2.02385 , Dendronephthya_suensoni_NC_022809 #2.00993 ) #2.06924 ) #0.0706291 , Scleronephthya_gracillimum_NC_023344 #0.0001 ) #0.0001 , (Echinogorgia_complexa_NC_020457 #0.0001 , Paramuricea_clavata_NC_034749 #0.0001 ) #0.0001 ) #0.0001 , Trachythela_sp__YZ_2021_MW238423 #0.0001 ) #1.03609 ) #1.05182 , (Incrustatus_comauensis_MT254531 #108.313 , Telestula_humilis_MT254527 #0.0001 ) #1.04983 ) #0.0001 ) #0.0400291 , Briareum_asbestinum_NC_008073 #0.328206 ) #83.3247 , (Telestula_cf__batoni_AP_2020_MT254530 #0.0001 , Telestula_septentrionalis_MT254532 #0.0001 ) #0.0001 ) #0.0001 , (((Anthomastus_sp__USNM_1081145_KM015353 #0.0001 , Anthomastus_sp__USNM_1171062_KM015352 #0.083203 ) #0.0001 , (((Corallium_japonicum_AB595189 #109.483 , Corallium_rubrum_NC_022864 #2.12277 ) #0.0001 , (Pleurocorallium_elatius_NC_022804 #0.557115 , Pleurocorallium_konojoi_NC_015406 #0.559283 ) #0.177664 ) #0.0001 , Sibogagorgia_cauliflora_NC_026193 #0.376229 ) #98.2724 ) #0.0001 , Paraminabea_aldersladei_NC_018790 #0.142227 ) #0.119637 ) #0.0001 , (((((((Anthoptilum_grandiflorum_NC_044086 #0.0001 , Anthoptilum_sp__1_RH_2019_MK919656 #0.0001 ) #0.0001 , (Umbellula_huxleyi_MK919668 #0.0001 , Umbellula_sp__1_RH_2019_MK919669 #0.24031 ) #0.0001 ) #0.0001 , Virgularia_mirabilis_NC_044091 #0.167398 ) #0.0001 , (Funiculina_quadrangularis_NC_044078 #107.044 , ((Kophobelemnon_sp__1_RH_2019_MK919660 #0.0001 , Kophobelemnon_sp__3_RH_2019_MK919661 #0.0001 ) #0.378839 , Kophobelemnon_sp__4_RH_2019_MK919662 #0.302312 ) #0.0001 ) #0.0001 ) #0.0001 , (Halipteris_cf__finmarchica_RH_2019_MK919659 #135.518 , Umbellula_sp__2_RH_2019_MK919670 #0.359595 ) #1.02649 ) #0.0001 , ((((Distichoptilum_gracile_NC_044077 #0.0001 , (Pennatula_cf__inflata_RH_2019_MK919666 #1.17714 , Pennatula_grandis_NC_044088 #1.13912 ) #0.0001 ) #0.0001 , ((Pennatula_aculeata_NC_044087 #2.22554 , Pennatula_cf__aculeata_RH_2019_MK919664 #2.26619 ) #0.283155 , Renilla_muelleri_NC_018378 #0.0637539 ) #0.0687944 ) #2.1313 , Protoptilum_carpenteri_NC_044089 #0.0001 ) #106.691 , Stylatula_elongata_NC_018380 #0.0001 ) #0.0001 ) #0.0001 , Junceella_fragilis_NC_024181 #0.0929278 ) #0.0001 ) #107.463 , Heliopora_coerulea_NC_020375 #0.0001 ) #0.184581 , ((Callogorgia_cf__gracilis_EE_2018_MH719202 #107.645 , (Plumarella_adhaerans_NC_046480 #0.0001 , Plumarella_spinosa_NC_046465 #0.0001 ) #0.0606879 ) #0.0001 , Narella_hawaiiensis_NC_026192 #0.0001 ) #0.0518466 ) #0.180892 , Keratoisidinae_sp__BAL208_1_NC_010764 #0.0001 );

NAD4:

**dS tree:**

(Acanella_arbuscula_NC_011016: 0.011406, ((((((((((((((((((((((((((((((Acropora_aculeus_KT001202: 0.000005, (Acropora_horrida_NC_022825: 0.000005, LC201842_1_LC201842_1__LC201842_1: 0.000005): 0.000002): 0.000005, LC201815_1_LC201815_1__LC201815_1: 0.000005): 0.000005, ((((Acropora_aspera_NC_022827: 0.000005, Acropora_muricata_NC_022824: 0.000005): 0.000005, LC201828_1_LC201828_1__LC201828_1: 0.000005): 0.000005, (((Acropora_digitifera_NC_022830: 0.000005, LC201849_1_LC201849_1__LC201849_1: 0.000005): 0.000005, Acropora_florida_NC_022828: 0.000005): 0.000005, Acropora_humilis_NC_022823: 0.000005): 0.000005): 0.000005, (Acropora_hyacinthus_NC_022826: 0.000005, LC201817_1_LC201817_1__LC201817_1: 0.000005): 0.000005): 0.000005): 0.000005, (Acropora_divaricata_NC_022832: 0.003776, (((Acropora_nasuta_NC_022831: 0.000005, Acropora_valida_NC_042742: 0.000005): 0.000005, LC201847_1_LC201847_1__LC201847_1: 0.000005): 0.000005, LC201820_1_LC201820_1__LC201820_1: 0.000005): 0.000005): 0.000005): 0.000005, Acropora_robusta_NC_022833: 0.003778): 0.000005, LC201813_1_LC201813_1__LC201813_1: 0.000002): 0.000005, ((Acropora_tenuis_NC_003522: 0.000005, Acropora_yongei_NC_022829: 0.000005): 0.000005, LC201816_1_LC201816_1__LC201816_1: 0.000005): 0.000005): 0.007642, (Isopora_palifera_NC_024091: 0.003790, Isopora_togianensis_NC_024089: 0.003807): 0.003783): 0.041021, ((Anacropora_matthai_NC_006898: 0.000003, Montipora_efflorescens_NC_040137: 0.000005): 0.003894, (Montipora_aequituberculata_NC_037359: 0.000005, Montipora_cactus_NC_006902: 0.003729): 0.003570): 0.108565): 0.008474, (Alveopora_japonica_NC_040136: 0.003815, Alveopora_sp__MFL_2014_KJ634271: 0.000005): 0.126298): 0.003979, (Astreopora_explanata_NC_024090: 0.003761, Astreopora_myriophthalma_NC_024092: 0.000005): 0.036775): 0.103862, ((Agaricia_humilis_NC_008160: 0.033308, Pavona_decussata_NC_026527: 0.023080): 0.176546, (Fimbriaphyllia_ancora_NC_015641: 0.006845, Galaxea_fascicularis_NC_029696: 0.035377): 0.179985): 0.000002): 0.023214, ((Pseudosiderastrea_formosa_NC_026530: 0.024611, Pseudosiderastrea_tayamai_NC_026531: 0.002864): 0.085748, Siderastrea_radians_NC_008167: 0.097882): 0.066981): 0.050750, ((((((Dendrophyllia_arbuscula_NC_027590: 0.003849, Tubastraea_coccinea_NC_026025: 0.003856): 0.003902, ((Tubastraea_diaphana_MK959042: 0.000005, Tubastraea_tagusensis_NC_030352: 0.000005): 0.000005, Tubastraea_micranthus_MK959041: 0.000005): 0.011728): 0.007615, Dendrophyllia_cribrosa_NC_026026: 0.003999): 0.052342, Turbinaria_peltata_NC_024671: 0.063679): 0.029125, (((Goniopora_columna_NC_015643: 0.007528, Goniopora_djiboutiensis_NC_045931: 0.000002): 0.000005, Goniopora_lobata_MN795054: 0.038588): 0.079663, ((Porites_fontanesii_NC_037434: 0.067682, ((Porites_panamensis_NC_024182: 0.000005, Porites_sverdrupi_KU956960: 0.003813): 0.000005, Porites_porites_NC_008166: 0.000002): 0.005268): 0.011876, (Porites_harrisoni_NC_037435: 0.011525, ((Porites_lobata_NC_030186: 0.000005, Porites_okinawensis_NC_015644: 0.000005): 0.007678, (Porites_lutea_NC_029695: 0.003841, Porites_rus_NC_027526: 0.003826): 0.000005): 0.000005): 0.022434): 0.056283): 0.111153): 0.020134, Fungiacyathus_stephanus_NC_015640: 0.085289): 0.062055): 0.181401, Gardineria_hawaiiensis_NC_056290: 0.276179): 0.129633, (((((Amplexidiscus_fenestrafer_NC_027101: 0.004127, (Discosoma_sp__MH308003: 0.007856, (Rhodactis_indosinensis_NC_027103: 0.000005, Rhodactis_sp__CASIZ_171755_NC_008158: 0.011757): 0.003926): 0.007500): 0.030156, (Discosoma_nummiforme_NC_027100: 0.016690, ((Discosoma_sp__CASIZ_168915_NC_008071: 0.000005, Discosoma_sp__CASIZ_168916_NC_008072: 0.000005): 0.000005, Platyzoanthus_mussoides_NC_027104: 0.000002): 0.011027): 0.014960): 0.132677, (Pseudocorynactis_sp__SIO_KP938437: 0.199234, (Ricordea_florida_NC_008159: 0.011899, Ricordea_yuma_NC_027106: 0.036734): 0.170367): 0.073847): 0.034206, Corynactis_californica_NC_027102: 0.097654): 0.061059, Corallimorphus_profundus_KP938440: 0.118907): 0.100954): 0.194944, ((((((Astrangia_sp__JVK_2006_NC_008161: 0.180892, (((Colpophyllia_natans_NC_008162: 0.012979, Mussa_angulosa_NC_008163: 0.003491): 0.048473, ((((((Dipsastraea_favus_NC_046690: 0.016905, Dipsastraea_rotumana_NC_044074: 0.003723): 0.017075, Favites_abdita_NC_035879: 0.051085): 0.000005, Platygyra_carnosa_NC_020049: 0.029420): 0.000000, (Favites_pentagona_NC_034916: 0.016505, Hydnophora_exesa_NC_042795: 0.008225): 0.000005): 0.047848, ((Orbicella_annularis_NC_007224: 0.000005, Orbicella_faveolata_NC_007226: 0.000002): 0.000005, Orbicella_franksi_NC_007225: 0.004077): 0.047592): 0.014531, Echinophyllia_aspera_NC_040169: 0.114601): 0.002195): 0.109799, Plesiastrea_versipora_NC_042481: 0.035937): 0.107633): 0.062651, (Polycyathus_sp__MFL_2011_NC_015642: 0.013779, Psammocora_nierstraszi_MT576637: 0.018054): 0.202427): 0.300459, Madrepora_oculata_NC_018364: 0.528604): 0.087112, (Madracis_myriaster_NC_011160: 0.032219, ((Pocillopora_damicornis_NC_009797: 0.000005, Pocillopora_grandis_NC_009798: 0.000005): 0.062066, ((Seriatopora_caliendrum_NC_010245: 0.004317, Seriatopora_hystrix_NC_010244: 0.018990): 0.000003, Stylophora_pistillata_NC_011162: 0.008659): 0.000003): 0.056181): 0.403734): 0.232066, ((Desmophyllum_dianthus_NC_034275: 0.007944, Desmophyllum_pertusum_NC_015143: 0.003998): 0.012098, Solenosmilia_variabilis_NC_025472: 0.000005): 0.776924): 0.928473, Paraconotrochus_antarcticus_NC_056275: 1.404425): 1.014432): 0.144602, ((((((Antipathes_cf__dichotoma_NB_2020_MT318841: 0.006190, Stichopathes_abyssicola_MT318856: 0.004666): 0.190938, (Phanopathes_sp__NB_2020_MT318852: 0.052963, Stichopathes_sp__n__NB_2020_MT318857: 0.020102): 0.167747): 0.179987, ((Stichopathes_luetkeni_NC_018377: 0.003918, Stichopathes_sp__SCBUCN_8850_MZ157399: 0.000005): 0.019786, Stichopathes_sp__SCBUCN_8849_MZ157400: 0.000005): 0.320378): 0.113211, ((Myriopathes_japonica_NC_027667: 0.006962, Tanacetipathes_thamnea_NC_046843: 0.015446): 0.041710, Tylopathes_sp__n__NB_2020_MT318859: 0.039360): 0.214275): 0.045922, (((((((Bathypathes_sp__1_NB_2020_MT318844: 0.000000, Bathypathes_sp__n__2_NB_2020_MT318842: 0.000000): 0.000000, Stauropathes_arctica_MT318854: 0.000000): 0.000005, Bathypathes_sp__n__3_NB_2020_MT318843: 0.003670): 0.011129, Stauropathes_cf__punctata_NB_2020_MT318855: 0.007386): 0.000005, Telopathes_sp__NB_2020_MT318858: 0.007409): 0.040296, (Dendrobathypathes_sp__n__NB_2020_MT318845: 0.074447, ((Parantipathes_cf__hirondelle_NB_2020_MT318849: 0.000002, Parantipathes_hirondelle_MT318850: 0.000000): 0.000000, (Parantipathes_sp__NB_2020_MT318851: 0.000000, Sibopathes_cf__macrospina_NB_2020_MT318853: 0.000000): 0.000000): 0.046786): 0.040694): 0.095330, (Chrysopathes_formosa_NC_008411: 0.019067, Trissopathes_cf__tetracrada_NB_2020_MT318840: 0.026212): 0.042238): 0.022462): 0.067949, (Leiopathes_cf__glaberrima_NB_2020_MT318846: 0.003707, Leiopathes_expansa_MT318847: 0.000005): 0.089361): 0.415107): 0.036810, (Epizoanthus_illoricatus_NC_046474: 0.203952, (((Hydrozoanthus_gracilis_NC_046400: 0.069595, ((Neozoanthus_aff__uchina_AP_2020_MN873592: 0.029300, ((Zoanthus_cf__pulchellus_AP_2020_MN873599: 0.000000, ((Zoanthus_cf__sociatus_AP_2020_MN873600: 0.000000, Zoanthus_sociatus_NC_046476: 0.003533): 0.000000, Zoanthus_sp__AP_2020_MN873602: 0.000000): 0.000000): 0.003508, Zoanthus_sansibaricus_NC_035578: 0.000004): 0.039868): 0.024947, (Palythoa_heliodiscus_NC_035579: 0.039717, ((Palythoa_mizigama_NC_046403: 0.006927, Palythoa_mutuki_NC_046404: 0.003451): 0.000000, Sphenopus_marsupialis_NC_046406: 0.000000): 0.007572): 0.002958): 0.009279): 0.051023, ((Parazoanthus_elongatus_NC_046405: 0.010403, Parazoanthus_swiftii_NC_046475: 0.000003): 0.020988, Savalia_savaglia_DQ825686: 0.014148): 0.021385): 0.024459, (Microzoanthus_occultus_NC_046401: 1.621194, Nanozoanthus_harenaceus_NC_046402: 1.045842): 0.854258): 0.095007): 0.710567): 0.000037, (((((((Actinia_equina_NC_039929: 0.000005, Actinia_tenebrosa_NC_044902: 0.000005): 0.023331, (((Anemonia_manjano_NC_037178: 0.019064, Heteractis_aurora_NC_047219: 0.015446): 0.030951, (Anemonia_sulcata_NC_049065: 0.000005, Anemonia_viridis_NC_037177: 0.000005): 0.030799): 0.000002, Anthopleura_anjunae_NC_030274: 0.018937): 0.003307): 0.012575, Phymanthus_crucifer_NC_027614: 0.120708): 0.009194, ((((Bolocera_sp__BZ_2016_KU507297: 0.011316, (Bolocera_tuediae_NC_022470: 0.000002, Liponema_brevicorne_NC_047221: 0.007486): 0.000005): 0.008879, (Entacmaea_quadricolor_NC_049066: 0.002959, Isosicyonis_striata_KR051006: 0.027636): 0.038164): 0.000002, Stichodactyla_haddoni_MW760873: 0.106930): 0.000005, Epiactis_japonica_NC_047217: 0.133834): 0.000256): 0.034890, ((((Alicia_sansibarensis_NC_027610: 0.349498, (Haloclava_producta_NC_047218: 0.037602, Sagartia_ornata_NC_027615: 0.056857): 0.033291): 0.067784, (Diadumene_lineata_NC_045515: 0.189317, Metridium_senile_NC_000933: 0.037617): 0.076463): 0.096379, Paraphelliactis_xishaensis_MT997141: 0.109797): 0.012288, Antholoba_achates_NC_027611: 0.562037): 0.061463): 0.056776, Halcampoides_purpureus_NC_027612: 0.295068): 0.315970, Nematostella_sp__JVK_2006_NC_008164: 0.651538): 0.563506): 3.576793, (((((((Aurelia_aurita_NC_008446: 0.554450, (Aurelia_coerulea_NC_046792: 0.462210, Aurelia_limbata_NC_046691: 0.766701): 0.153629): 0.352994, (Aurelia_sp__3_sensu_Dawson_et_al___2005__LC005413: 0.638227, Aurelia_sp__4_sensu_Dawson_et_al___2005__LC005414: 0.651828): 0.178351): 0.982032, ((Nemopilema_nomurai_NC_035740: 1.313386, Rhopilema_esculentum_NC_035741: 0.989524): 0.239806, Stomolophus_sp__CG_2019_MK157198: 2.046932): 0.000095): 0.000047, Cassiopea_xamachana_NC_016466: 1.611194): 0.000048, (Chrysaora_pacifica_NC_046775: 0.000006, Chrysaora_quinquecirrha_NC_020459: 0.056651): 2.294219): 0.037110, ((((((Blackfordia_virginica_NC_053946: 1.433722, Eutima_sp__BMK_2020_MW066348: 2.140478): 0.218256, Laomedea_flexuosa_JN700945: 1.221764): 0.000057, (Clava_multicornis_NC_016465: 1.088346, (Turritopsis_dohrnii_NC_031213: 2.048371, Turritopsis_lata_NC_056128: 1.244799): 0.000174): 0.000057): 0.000028, Spirocodon_saltatrix_NC_049868: 1.993821): 0.000111, ((Hydra_oligactis_NC_010214: 0.823480, Hydra_sinensis_NC_021406: 0.997718): 0.566799, Nemalecium_lighti_MZ457217: 1.563881): 0.000057): 0.367987, (Craspedacusta_sowerbii_NC_018537: 4.209672, Cubaia_aphrodite_NC_016467: 3.079302): 0.000362): 0.000038): 0.153986, (Haliclystus_antarcticus_NC_030337: 1.912178, OU744455_1_OU744455_1__OU744455_1: 1.479299): 1.576939): 0.000166): 2.867699, Carijoa_riisei_NC_048963: 0.000005): 0.143933, (Sarcophyton_trocheliophorum_MK994517: 0.184282, (((Sinularia_acuta_MW987591: 0.000005, (Sinularia_maxima_MN485891: 0.004014, Sinularia_penghuensis_MW256412: 0.008068): 0.004023): 0.011670, Sinularia_cf__cruciata_GS_2016_NC_034318: 0.016929): 0.035517, (Sinularia_ceramensis_NC_044122: 0.012116, Sinularia_peculiaris_NC_018379: 0.004131): 0.036028): 0.022450): 0.021719): 0.015929, (((((((Antillogorgia_bipinnata_NC_008157: 0.076506, ((((Eugorgia_mutabilis_NC_035665: 0.041791, Leptogorgia_alba_NC_035669: 0.012115): 0.003326, (Leptogorgia_hebes_MN052675: 0.069016, Leptogorgia_virgulata_MK301586: 0.004570): 0.000005): 0.003636, Leptogorgia_sp__USNM_1437444_KY559412: 0.008552): 0.039542, Pacifigorgia_cairnsi_NC_035668: 0.095797): 0.014162): 0.027005, (((Leptogorgia_capverdensis_NC_035663: 0.000005, Leptogorgia_sarmentosa_NC_035670: 0.000005): 0.044239, Leptogorgia_gaini_KY559404: 0.031820): 0.018724, Leptogorgia_cf__palma_AP_2017_KY559406: 0.044483): 0.000005): 0.024203, (Eunicella_albicans_NC_035666: 0.011510, (Eunicella_cavolini_NC_035667: 0.000005, Eunicella_verrucosa_MW588805: 0.003923): 0.028587): 0.027058): 0.019081, (Calicogorgia_granulosa_NC_023345: 0.570412, Euplexaura_crassa_NC_020458: 0.065474): 0.008853): 0.005084, Muricea_crassa_NC_029697: 0.075831): 0.004086, ((((((Dendronephthya_castanea_NC_023343: 0.000005, Dendronephthya_mollis_NC_020456: 0.000005): 0.000005, Dendronephthya_gigantea_NC_013573: 0.000005): 0.003679, (Dendronephthya_putteri_NC_036022: 0.000005, Dendronephthya_suensoni_NC_022809: 0.000005): 0.004150): 0.045545, Scleronephthya_gracillimum_NC_023344: 0.043596): 0.011995, (Echinogorgia_complexa_NC_020457: 0.090885, Paramuricea_clavata_NC_034749: 0.214377): 0.000005): 0.021048, Trachythela_sp__YZ_2021_MW238423: 0.025660): 0.022301): 0.015141, (Incrustatus_comauensis_MT254531: 0.037143, Telestula_humilis_MT254527: 0.018487): 0.034964): 0.004908): 0.132633, Briareum_asbestinum_NC_008073: 0.066319): 0.018678, (Telestula_cf__batoni_AP_2020_MT254530: 0.012163, Telestula_septentrionalis_MT254532: 0.003879): 0.020689): 0.000005, (((Anthomastus_sp__USNM_1081145_KM015353: 0.012028, Anthomastus_sp__USNM_1171062_KM015352: 0.000002): 0.037165, (((Corallium_japonicum_AB595189: 0.007758, Corallium_rubrum_NC_022864: 0.012135): 0.016747, (Pleurocorallium_elatius_NC_022804: 0.000005, Pleurocorallium_konojoi_NC_015406: 0.003903): 0.083622): 0.005835, Sibogagorgia_cauliflora_NC_026193: 0.049858): 0.003715): 0.012218, Paraminabea_aldersladei_NC_018790: 0.079271): 0.070879): 0.007565, (((((((Anthoptilum_grandiflorum_NC_044086: 0.003960, Anthoptilum_sp__1_RH_2019_MK919656: 0.003980): 0.004036, (Umbellula_huxleyi_MK919668: 0.000005, Umbellula_sp__1_RH_2019_MK919669: 0.019909): 0.028360): 0.007708, Virgularia_mirabilis_NC_044091: 0.067815): 0.012285, (Funiculina_quadrangularis_NC_044078: 0.016014, ((Kophobelemnon_sp__1_RH_2019_MK919660: 0.015931, Kophobelemnon_sp__3_RH_2019_MK919661: 0.003918): 0.007951, Kophobelemnon_sp__4_RH_2019_MK919662: 0.000003): 0.015930): 0.003967): 0.000002, (Halipteris_cf__finmarchica_RH_2019_MK919659: 0.044680, Umbellula_sp__2_RH_2019_MK919670: 0.020122): 0.000005): 0.000002, ((((Distichoptilum_gracile_NC_044077: 0.030699, (Pennatula_cf__inflata_RH_2019_MK919666: 0.003839, Pennatula_grandis_NC_044088: 0.000002): 0.045844): 0.006925, ((Pennatula_aculeata_NC_044087: 0.003818, Pennatula_cf__aculeata_RH_2019_MK919664: 0.003853): 0.013371, Renilla_muelleri_NC_018378: 0.064223): 0.083021): 0.004696, Protoptilum_carpenteri_NC_044089: 0.007677): 0.011922, Stylatula_elongata_NC_018380: 0.088825): 0.032797): 0.012172, Junceella_fragilis_NC_024181: 0.130138): 0.020474): 0.016116, Heliopora_coerulea_NC_020375: 0.090069): 0.030598, ((Callogorgia_cf__gracilis_EE_2018_MH719202: 0.032704, (Plumarella_adhaerans_NC_046480: 0.000005, Plumarella_spinosa_NC_046465: 0.000005): 0.024132): 0.003868, Narella_hawaiiensis_NC_026192: 0.000002): 0.007007): 0.020924, Keratoisidinae_sp__BAL208_1_NC_010764: 0.011719);

**dN tree:**

(Acanella_arbuscula_NC_011016: 0.005246, ((((((((((((((((((((((((((((((Acropora_aculeus_KT001202: 0.000000, (Acropora_horrida_NC_022825: 0.000000, LC201842_1_LC201842_1__LC201842_1: 0.000000): 0.001307): 0.000000, LC201815_1_LC201815_1__LC201815_1: 0.000000): 0.000000, ((((Acropora_aspera_NC_022827: 0.000000, Acropora_muricata_NC_022824: 0.000000): 0.000000, LC201828_1_LC201828_1__LC201828_1: 0.000000): 0.000000, (((Acropora_digitifera_NC_022830: 0.000000, LC201849_1_LC201849_1__LC201849_1: 0.000000): 0.000000, Acropora_florida_NC_022828: 0.000000): 0.000000, Acropora_humilis_NC_022823: 0.000000): 0.000000): 0.000000, (Acropora_hyacinthus_NC_022826: 0.000000, LC201817_1_LC201817_1__LC201817_1: 0.000000): 0.000000): 0.000000): 0.000000, (Acropora_divaricata_NC_022832: 0.000000, (((Acropora_nasuta_NC_022831: 0.000000, Acropora_valida_NC_042742: 0.000000): 0.000000, LC201847_1_LC201847_1__LC201847_1: 0.000000): 0.000000, LC201820_1_LC201820_1__LC201820_1: 0.000000): 0.000000): 0.000000): 0.000000, Acropora_robusta_NC_022833: 0.000000): 0.000000, LC201813_1_LC201813_1__LC201813_1: 0.001305): 0.000000, ((Acropora_tenuis_NC_003522: 0.000000, Acropora_yongei_NC_022829: 0.000000): 0.000000, LC201816_1_LC201816_1__LC201816_1: 0.000000): 0.000000): 0.000001, (Isopora_palifera_NC_024091: 0.001302, Isopora_togianensis_NC_024089: 0.000000): 0.001303): 0.002588, ((Anacropora_matthai_NC_006898: 0.002646, Montipora_efflorescens_NC_040137: 0.000000): 0.000000, (Montipora_aequituberculata_NC_037359: 0.000000, Montipora_cactus_NC_006902: 0.001322): 0.000000): 0.003979): 0.000001, (Alveopora_japonica_NC_040136: 0.002588, Alveopora_sp__MFL_2014_KJ634271: 0.000000): 0.005250): 0.001662, (Astreopora_explanata_NC_024090: 0.000000, Astreopora_myriophthalma_NC_024092: 0.000000): 0.004934): 0.006614, ((Agaricia_humilis_NC_008160: 0.002745, Pavona_decussata_NC_026527: 0.002696): 0.013860, (Fimbriaphyllia_ancora_NC_015641: 0.006681, Galaxea_fascicularis_NC_029696: 0.002571): 0.017942): 0.000958): 0.001256, ((Pseudosiderastrea_formosa_NC_026530: 0.001278, Pseudosiderastrea_tayamai_NC_026531: 0.000000): 0.003920, Siderastrea_radians_NC_008167: 0.010660): 0.002740): 0.002558, ((((((Dendrophyllia_arbuscula_NC_027590: 0.000000, Tubastraea_coccinea_NC_026025: 0.000000): 0.000000, ((Tubastraea_diaphana_MK959042: 0.000000, Tubastraea_tagusensis_NC_030352: 0.000000): 0.000000, Tubastraea_micranthus_MK959041: 0.000000): 0.000001): 0.000001, Dendrophyllia_cribrosa_NC_026026: 0.000000): 0.002570, Turbinaria_peltata_NC_024671: 0.002609): 0.002597, (((Goniopora_columna_NC_015643: 0.000001, Goniopora_djiboutiensis_NC_045931: 0.001305): 0.000000, Goniopora_lobata_MN795054: 0.000004): 0.002602, ((Porites_fontanesii_NC_037434: 0.002590, ((Porites_panamensis_NC_024182: 0.000000, Porites_sverdrupi_KU956960: 0.000000): 0.000000, Porites_porites_NC_008166: 0.001292): 0.001291): 0.000001, (Porites_harrisoni_NC_037435: 0.000001, ((Porites_lobata_NC_030186: 0.000000, Porites_okinawensis_NC_015644: 0.000000): 0.000001, (Porites_lutea_NC_029695: 0.000000, Porites_rus_NC_027526: 0.000000): 0.000000): 0.000000): 0.000002): 0.005198): 0.005249): 0.004023, Fungiacyathus_stephanus_NC_015640: 0.003773): 0.001320): 0.015396, Gardineria_hawaiiensis_NC_056290: 0.027216): 0.004854, (((((Amplexidiscus_fenestrafer_NC_027101: 0.002567, (Discosoma_sp__MH308003: 0.001279, (Rhodactis_indosinensis_NC_027103: 0.000000, Rhodactis_sp__CASIZ_171755_NC_008158: 0.000001): 0.000000): 0.000001): 0.000003, (Discosoma_nummiforme_NC_027100: 0.003833, ((Discosoma_sp__CASIZ_168915_NC_008071: 0.000000, Discosoma_sp__CASIZ_168916_NC_008072: 0.000000): 0.000000, Platyzoanthus_mussoides_NC_027104: 0.001268): 0.003828): 0.005128): 0.007863, (Pseudocorynactis_sp__SIO_KP938437: 0.004068, (Ricordea_florida_NC_008159: 0.007647, Ricordea_yuma_NC_027106: 0.001305): 0.015547): 0.007750): 0.011000, Corynactis_californica_NC_027102: 0.010313): 0.001937, Corallimorphus_profundus_KP938440: 0.009431): 0.009790): 0.017521, ((((((Astrangia_sp__JVK_2006_NC_008161: 0.003872, (((Colpophyllia_natans_NC_008162: 0.000001, Mussa_angulosa_NC_008163: 0.003577): 0.003671, ((((((Dipsastraea_favus_NC_046690: 0.000002, Dipsastraea_rotumana_NC_044074: 0.002393): 0.001193, Favites_abdita_NC_035879: 0.003602): 0.000000, Platygyra_carnosa_NC_020049: 0.001189): 0.000002, (Favites_pentagona_NC_034916: 0.000002, Hydnophora_exesa_NC_042795: 0.003580): 0.000000): 0.000005, ((Orbicella_annularis_NC_007224: 0.000000, Orbicella_faveolata_NC_007226: 0.001198): 0.000000, Orbicella_franksi_NC_007225: 0.001196): 0.006003): 0.007228, Echinophyllia_aspera_NC_040169: 0.014409): 0.002561): 0.010029, Plesiastrea_versipora_NC_042481: 0.002708): 0.001392): 0.001702, (Polycyathus_sp__MFL_2011_NC_015642: 0.001107, Psammocora_nierstraszi_MT576637: 0.003386): 0.016590): 0.020890, Madrepora_oculata_NC_018364: 0.040008): 0.007732, (Madracis_myriaster_NC_011160: 0.005097, ((Pocillopora_damicornis_NC_009797: 0.000000, Pocillopora_grandis_NC_009798: 0.000000): 0.011899, ((Seriatopora_caliendrum_NC_010245: 0.002270, Seriatopora_hystrix_NC_010244: 0.012304): 0.002319, Stylophora_pistillata_NC_011162: 0.000001): 0.003434): 0.005471): 0.048134): 0.008051, ((Desmophyllum_dianthus_NC_034275: 0.000001, Desmophyllum_pertusum_NC_015143: 0.000000): 0.002415, Solenosmilia_variabilis_NC_025472: 0.000000): 0.032042): 0.086527, Paraconotrochus_antarcticus_NC_056275: 0.112836): 0.049746): 0.037853, ((((((Antipathes_cf__dichotoma_NB_2020_MT318841: 0.000001, Stichopathes_abyssicola_MT318856: 0.000000): 0.017122, (Phanopathes_sp__NB_2020_MT318852: 0.011066, Stichopathes_sp__n__NB_2020_MT318857: 0.002375): 0.013601): 0.004339, ((Stichopathes_luetkeni_NC_018377: 0.000000, Stichopathes_sp__SCBUCN_8850_MZ157399: 0.000000): 0.000002, Stichopathes_sp__SCBUCN_8849_MZ157400: 0.000000): 0.001884): 0.003458, ((Myriopathes_japonica_NC_027667: 0.002767, Tanacetipathes_thamnea_NC_046843: 0.000002): 0.002743, Tylopathes_sp__n__NB_2020_MT318859: 0.007097): 0.007016): 0.001414, (((((((Bathypathes_sp__1_NB_2020_MT318844: 0.000002, Bathypathes_sp__n__2_NB_2020_MT318842: 0.000002): 0.000002, Stauropathes_arctica_MT318854: 0.000002): 0.000000, Bathypathes_sp__n__3_NB_2020_MT318843: 0.001354): 0.002705, Stauropathes_cf__punctata_NB_2020_MT318855: 0.002706): 0.000000, Telopathes_sp__NB_2020_MT318858: 0.000001): 0.000004, (Dendrobathypathes_sp__n__NB_2020_MT318845: 0.004111, ((Parantipathes_cf__hirondelle_NB_2020_MT318849: 0.001370, Parantipathes_hirondelle_MT318850: 0.000002): 0.000002, (Parantipathes_sp__NB_2020_MT318851: 0.000002, Sibopathes_cf__macrospina_NB_2020_MT318853: 0.000002): 0.000002): 0.002729): 0.001357): 0.006803, (Chrysopathes_formosa_NC_008411: 0.000002, Trissopathes_cf__tetracrada_NB_2020_MT318840: 0.000003): 0.002705): 0.005427): 0.001305, (Leiopathes_cf__glaberrima_NB_2020_MT318846: 0.000000, Leiopathes_expansa_MT318847: 0.000000): 0.001423): 0.010287): 0.002742, (Epizoanthus_illoricatus_NC_046474: 0.008487, (((Hydrozoanthus_gracilis_NC_046400: 0.002950, ((Neozoanthus_aff__uchina_AP_2020_MN873592: 0.002951, ((Zoanthus_cf__pulchellus_AP_2020_MN873599: 0.000002, ((Zoanthus_cf__sociatus_AP_2020_MN873600: 0.000002, Zoanthus_sociatus_NC_046476: 0.000000): 0.000002, Zoanthus_sp__AP_2020_MN873602: 0.000002): 0.000002): 0.000000, Zoanthus_sansibaricus_NC_035578: 0.004378): 0.000004): 0.000002, (Palythoa_heliodiscus_NC_035579: 0.010452, ((Palythoa_mizigama_NC_046403: 0.001480, Palythoa_mutuki_NC_046404: 0.002972): 0.000002, Sphenopus_marsupialis_NC_046406: 0.000002): 0.001484): 0.000000): 0.003015): 0.001547, ((Parazoanthus_elongatus_NC_046405: 0.001678, Parazoanthus_swiftii_NC_046475: 0.002782): 0.000002, Savalia_savaglia_DQ825686: 0.001676): 0.001261): 0.005520, (Microzoanthus_occultus_NC_046401: 0.049248, Nanozoanthus_harenaceus_NC_046402: 0.042538): 0.062367): 0.005386): 0.024963): 0.010489, (((((((Actinia_equina_NC_039929: 0.000000, Actinia_tenebrosa_NC_044902: 0.000000): 0.003991, (((Anemonia_manjano_NC_037178: 0.001317, Heteractis_aurora_NC_047219: 0.000002): 0.001325, (Anemonia_sulcata_NC_049065: 0.000000, Anemonia_viridis_NC_037177: 0.000000): 0.000003): 0.001328, Anthopleura_anjunae_NC_030274: 0.003994): 0.000000): 0.000001, Phymanthus_crucifer_NC_027614: 0.013254): 0.001330, ((((Bolocera_sp__BZ_2016_KU507297: 0.001330, (Bolocera_tuediae_NC_022470: 0.001332, Liponema_brevicorne_NC_047221: 0.000001): 0.000000): 0.001329, (Entacmaea_quadricolor_NC_049066: 0.002669, Isosicyonis_striata_KR051006: 0.000003): 0.008050): 0.001336, Stichodactyla_haddoni_MW760873: 0.001322): 0.000000, Epiactis_japonica_NC_047217: 0.005335): 0.001336): 0.000003, ((((Alicia_sansibarensis_NC_027610: 0.027937, (Haloclava_producta_NC_047218: 0.000004, Sagartia_ornata_NC_027615: 0.000006): 0.000003): 0.000007, (Diadumene_lineata_NC_045515: 0.007910, Metridium_senile_NC_000933: 0.001316): 0.000008): 0.002627, Paraphelliactis_xishaensis_MT997141: 0.003946): 0.001327, Antholoba_achates_NC_027611: 0.013132): 0.000006): 0.002506, Halcampoides_purpureus_NC_027612: 0.010072): 0.014866, Nematostella_sp__JVK_2006_NC_008164: 0.033743): 0.006786): 0.100479, (((((((Aurelia_aurita_NC_008446: 0.020156, (Aurelia_coerulea_NC_046792: 0.014895, Aurelia_limbata_NC_046691: 0.009489): 0.016906): 0.017466, (Aurelia_sp__3_sensu_Dawson_et_al___2005__LC005413: 0.027228, Aurelia_sp__4_sensu_Dawson_et_al___2005__LC005414: 0.035598): 0.015607): 0.063130, ((Nemopilema_nomurai_NC_035740: 0.021997, Rhopilema_esculentum_NC_035741: 0.042848): 0.048220, Stomolophus_sp__CG_2019_MK157198: 0.060553): 0.038086): 0.024469, Cassiopea_xamachana_NC_016466: 0.118855): 0.040737, (Chrysaora_pacifica_NC_046775: 0.000000, Chrysaora_quinquecirrha_NC_020459: 0.003884): 0.071642): 0.116900, ((((((Blackfordia_virginica_NC_053946: 0.038641, Eutima_sp__BMK_2020_MW066348: 0.058481): 0.025761, Laomedea_flexuosa_JN700945: 0.035790): 0.054205, (Clava_multicornis_NC_016465: 0.062421, (Turritopsis_dohrnii_NC_031213: 0.031505, Turritopsis_lata_NC_056128: 0.026744): 0.113196): 0.026421): 0.017988, Spirocodon_saltatrix_NC_049868: 0.098137): 0.021288, ((Hydra_oligactis_NC_010214: 0.048564, Hydra_sinensis_NC_021406: 0.117477): 0.056981, Nemalecium_lighti_MZ457217: 0.305694): 0.051515): 0.086691, (Craspedacusta_sowerbii_NC_018537: 0.088242, Cubaia_aphrodite_NC_016467: 0.078435): 0.052903): 0.026693): 0.011469, (Haliclystus_antarcticus_NC_030337: 0.031425, OU744455_1_OU744455_1__OU744455_1: 0.027836): 0.342126): 0.033670): 0.117869, Carijoa_riisei_NC_048963: 0.000000): 0.001235, (Sarcophyton_trocheliophorum_MK994517: 0.003714, (((Sinularia_acuta_MW987591: 0.000000, (Sinularia_maxima_MN485891: 0.000000, Sinularia_penghuensis_MW256412: 0.000001): 0.000000): 0.000001, Sinularia_cf__cruciata_GS_2016_NC_034318: 0.000002): 0.003736, (Sinularia_ceramensis_NC_044122: 0.000001, Sinularia_peculiaris_NC_018379: 0.000000): 0.001234): 0.001243): 0.000002): 0.000002, (((((((Antillogorgia_bipinnata_NC_008157: 0.002498, ((((Eugorgia_mutabilis_NC_035665: 0.001265, Leptogorgia_alba_NC_035669: 0.000001): 0.000000, (Leptogorgia_hebes_MN052675: 0.002550, Leptogorgia_virgulata_MK301586: 0.000000): 0.000000): 0.000000, Leptogorgia_sp__USNM_1437444_KY559412: 0.001270): 0.001260, Pacifigorgia_cairnsi_NC_035668: 0.007538): 0.000001): 0.000003, (((Leptogorgia_capverdensis_NC_035663: 0.000000, Leptogorgia_sarmentosa_NC_035670: 0.000000): 0.002480, Leptogorgia_gaini_KY559404: 0.001247): 0.000002, Leptogorgia_cf__palma_AP_2017_KY559406: 0.004992): 0.000000): 0.000002, (Eunicella_albicans_NC_035666: 0.002534, (Eunicella_cavolini_NC_035667: 0.000000, Eunicella_verrucosa_MW588805: 0.000000): 0.002539): 0.001254): 0.000002, (Calicogorgia_granulosa_NC_023345: 0.022181, Euplexaura_crassa_NC_020458: 0.003482): 0.001558): 0.000001, Muricea_crassa_NC_029697: 0.002463): 0.000000, ((((((Dendronephthya_castanea_NC_023343: 0.000000, Dendronephthya_mollis_NC_020456: 0.000000): 0.000000, Dendronephthya_gigantea_NC_013573: 0.000000): 0.000000, (Dendronephthya_putteri_NC_036022: 0.000000, Dendronephthya_suensoni_NC_022809: 0.000000): 0.000000): 0.001260, Scleronephthya_gracillimum_NC_023344: 0.003782): 0.000001, (Echinogorgia_complexa_NC_020457: 0.002516, Paramuricea_clavata_NC_034749: 0.007701): 0.000000): 0.002498, Trachythela_sp__YZ_2021_MW238423: 0.003738): 0.000002): 0.001253, (Incrustatus_comauensis_MT254531: 0.001241, Telestula_humilis_MT254527: 0.001237): 0.002502): 0.001248): 0.007601, Briareum_asbestinum_NC_008073: 0.002465): 0.006379, (Telestula_cf__batoni_AP_2020_MT254530: 0.001235, Telestula_septentrionalis_MT254532: 0.000000): 0.002471): 0.000000, (((Anthomastus_sp__USNM_1081145_KM015353: 0.000001, Anthomastus_sp__USNM_1171062_KM015352: 0.001231): 0.001234, (((Corallium_japonicum_AB595189: 0.000001, Corallium_rubrum_NC_022864: 0.000001): 0.003721, (Pleurocorallium_elatius_NC_022804: 0.000000, Pleurocorallium_konojoi_NC_015406: 0.000000): 0.003753): 0.000001, Sibogagorgia_cauliflora_NC_026193: 0.007499): 0.000000): 0.002468, Paraminabea_aldersladei_NC_018790: 0.006297): 0.002510): 0.000001, (((((((Anthoptilum_grandiflorum_NC_044086: 0.001239, Anthoptilum_sp__1_RH_2019_MK919656: 0.002477): 0.000000, (Umbellula_huxleyi_MK919668: 0.000000, Umbellula_sp__1_RH_2019_MK919669: 0.000002): 0.002480): 0.001226, Virgularia_mirabilis_NC_044091: 0.006320): 0.001251, (Funiculina_quadrangularis_NC_044078: 0.003721, ((Kophobelemnon_sp__1_RH_2019_MK919660: 0.000002, Kophobelemnon_sp__3_RH_2019_MK919661: 0.000000): 0.000001, Kophobelemnon_sp__4_RH_2019_MK919662: 0.002482): 0.000002): 0.000000): 0.001239, (Halipteris_cf__finmarchica_RH_2019_MK919659: 0.003747, Umbellula_sp__2_RH_2019_MK919670: 0.000002): 0.000000): 0.001462, ((((Distichoptilum_gracile_NC_044077: 0.002542, (Pennatula_cf__inflata_RH_2019_MK919666: 0.002555, Pennatula_grandis_NC_044088: 0.001268): 0.001267): 0.001264, ((Pennatula_aculeata_NC_044087: 0.000000, Pennatula_cf__aculeata_RH_2019_MK919664: 0.000000): 0.000001, Renilla_muelleri_NC_018378: 0.007640): 0.006343): 0.000000, Protoptilum_carpenteri_NC_044089: 0.002512): 0.000001, Stylatula_elongata_NC_018380: 0.003815): 0.003538): 0.000001, Junceella_fragilis_NC_024181: 0.006101): 0.001467): 0.001246, Heliopora_coerulea_NC_020375: 0.005038): 0.002509, ((Callogorgia_cf__gracilis_EE_2018_MH719202: 0.001243, (Plumarella_adhaerans_NC_046480: 0.000000, Plumarella_spinosa_NC_046465: 0.000000): 0.001252): 0.000000, Narella_hawaiiensis_NC_026192: 0.001249): 0.001245): 0.003793, Keratoisidinae_sp__BAL208_1_NC_010764: 0.005126);

**w ratios as labels for TreeView:**

(Acanella_arbuscula_NC_011016 #0.459934 , ((((((((((((((((((((((((((((((Acropora_aculeus_KT001202 #0.0001 , (Acropora_horrida_NC_022825 #0.0001 , LC201842_1_LC201842_1__LC201842_1 #0.0001 ) #801.788 ) #0.0001 , LC201815_1_LC201815_1__LC201815_1 #0.0001 ) #0.0001 , ((((Acropora_aspera_NC_022827 #0.0001 , Acropora_muricata_NC_022824 #0.0001 ) #0.0001 , LC201828_1_LC201828_1__LC201828_1 #0.0001 ) #0.0001 , (((Acropora_digitifera_NC_022830 #0.0001 , LC201849_1_LC201849_1__LC201849_1 #0.0001 ) #0.0001 , Acropora_florida_NC_022828 #0.0001 ) #0.0001 , Acropora_humilis_NC_022823 #0.0001 ) #0.0001 ) #0.0001 , (Acropora_hyacinthus_NC_022826 #0.0001 , LC201817_1_LC201817_1__LC201817_1 #0.0001 ) #0.000100001 ) #0.0001 ) #0.0001 , (Acropora_divaricata_NC_022832 #0.0001 , (((Acropora_nasuta_NC_022831 #0.0001 , Acropora_valida_NC_042742 #0.0001 ) #0.0001 , LC201847_1_LC201847_1__LC201847_1 #0.000100001 ) #0.0001 , LC201820_1_LC201820_1__LC201820_1 #0.0001 ) #0.0001 ) #0.0001 ) #0.0001 , Acropora_robusta_NC_022833 #0.0001 ) #0.0001 , LC201813_1_LC201813_1__LC201813_1 #801.844 ) #0.0001 , ((Acropora_tenuis_NC_003522 #0.0001 , Acropora_yongei_NC_022829 #0.0001 ) #0.0001 , LC201816_1_LC201816_1__LC201816_1 #0.0001 ) #0.0001 ) #0.0001 , (Isopora_palifera_NC_024091 #0.343575 , Isopora_togianensis_NC_024089 #0.0001 ) #0.344444 ) #0.0630815 , ((Anacropora_matthai_NC_006898 #999 , Montipora_efflorescens_NC_040137 #0.0001 ) #0.0001 , (Montipora_aequituberculata_NC_037359 #0.000100001 , Montipora_cactus_NC_006902 #0.354481 ) #0.0001 ) #0.0366492 ) #0.0001 , (Alveopora_japonica_NC_040136 #0.678308 , Alveopora_sp__MFL_2014_KJ634271 #0.0001 ) #0.0415665 ) #0.417687 , (Astreopora_explanata_NC_024090 #0.0001 , Astreopora_myriophthalma_NC_024092 #0.0001 ) #0.134156 ) #0.0636768 , ((Agaricia_humilis_NC_008160 #0.0824231 , Pavona_decussata_NC_026527 #0.116819 ) #0.0785036 , (Fimbriaphyllia_ancora_NC_015641 #0.976101 , Galaxea_fascicularis_NC_029696 #0.0726708 ) #0.0996879 ) #467.321 ) #0.0541003 , ((Pseudosiderastrea_formosa_NC_026530 #0.0519238 , Pseudosiderastrea_tayamai_NC_026531 #0.0001 ) #0.045721 , Siderastrea_radians_NC_008167 #0.108906 ) #0.0409008 ) #0.05041 , ((((((Dendrophyllia_arbuscula_NC_027590 #0.0001 , Tubastraea_coccinea_NC_026025 #0.0001 ) #0.0001 , ((Tubastraea_diaphana_MK959042 #0.0001 , Tubastraea_tagusensis_NC_030352 #0.0001 ) #0.0001 , Tubastraea_micranthus_MK959041 #0.0001 ) #0.0001 ) #0.0001 , Dendrophyllia_cribrosa_NC_026026 #0.0001 ) #0.0491061 , Turbinaria_peltata_NC_024671 #0.0409736 ) #0.0891497 , (((Goniopora_columna_NC_015643 #0.0001 , Goniopora_djiboutiensis_NC_045931 #801.961 ) #0.0001 , Goniopora_lobata_MN795054 #0.0001 ) #0.0326636 , ((Porites_fontanesii_NC_037434 #0.0382688 , ((Porites_panamensis_NC_024182 #0.0001 , Porites_sverdrupi_KU956960 #0.0001 ) #0.0001 , Porites_porites_NC_008166 #797.365 ) #0.245032 ) #0.0001 , (Porites_harrisoni_NC_037435 #0.0001 , ((Porites_lobata_NC_030186 #0.0001 , Porites_okinawensis_NC_015644 #0.0001 ) #0.0001 , (Porites_lutea_NC_029695 #0.0001 , Porites_rus_NC_027526 #0.0001 ) #0.0001 ) #0.0001 ) #0.0001 ) #0.0923594 ) #0.0472203 ) #0.199837 , Fungiacyathus_stephanus_NC_015640 #0.0442323 ) #0.0212704 ) #0.0848748 , Gardineria_hawaiiensis_NC_056290 #0.0985437 ) #0.0374469 , (((((Amplexidiscus_fenestrafer_NC_027101 #0.621908 , (Discosoma_sp__MH308003 #0.162835 , (Rhodactis_indosinensis_NC_027103 #0.0001 , Rhodactis_sp__CASIZ_171755_NC_008158 #0.0001 ) #0.0001 ) #0.0001 ) #0.0001 , (Discosoma_nummiforme_NC_027100 #0.229667 , ((Discosoma_sp__CASIZ_168915_NC_008071 #0.0001 , Discosoma_sp__CASIZ_168916_NC_008072 #0.0001 ) #0.0001 , Platyzoanthus_mussoides_NC_027104 #788.208 ) #0.347164 ) #0.342791 ) #0.0592679 , (Pseudocorynactis_sp__SIO_KP938437 #0.0204184 , (Ricordea_florida_NC_008159 #0.642697 , Ricordea_yuma_NC_027106 #0.0355129 ) #0.0912531 ) #0.104943 ) #0.321584 , Corynactis_californica_NC_027102 #0.105609 ) #0.031725 , Corallimorphus_profundus_KP938440 #0.0793168 ) #0.0969797 ) #0.0898749 , ((((((Astrangia_sp__JVK_2006_NC_008161 #0.0214058 , (((Colpophyllia_natans_NC_008162 #0.0001 , Mussa_angulosa_NC_008163 #1.02471 ) #0.0757279 , ((((((Dipsastraea_favus_NC_046690 #0.0001 , Dipsastraea_rotumana_NC_044074 #0.64277 ) #0.0698518 , Favites_abdita_NC_035879 #0.0705073 ) #0.0001 , Platygyra_carnosa_NC_020049 #0.0404206 ) #57.413 , (Favites_pentagona_NC_034916 #0.0001 , Hydnophora_exesa_NC_042795 #0.435263 ) #0.0001 ) #0.0001 , ((Orbicella_annularis_NC_007224 #0.0001 , Orbicella_faveolata_NC_007226 #760.184 ) #0.0001 , Orbicella_franksi_NC_007225 #0.293347 ) #0.126142 ) #0.497403 , Echinophyllia_aspera_NC_040169 #0.125733 ) #1.16664 ) #0.0913415 , Plesiastrea_versipora_NC_042481 #0.0753587 ) #0.0129292 ) #0.0271611 , (Polycyathus_sp__MFL_2011_NC_015642 #0.08034 , Psammocora_nierstraszi_MT576637 #0.18754 ) #0.0819533 ) #0.0695257 , Madrepora_oculata_NC_018364 #0.0756866 ) #0.0887536 , (Madracis_myriaster_NC_011160 #0.158195 , ((Pocillopora_damicornis_NC_009797 #0.0001 , Pocillopora_grandis_NC_009798 #0.0001 ) #0.191719 , ((Seriatopora_caliendrum_NC_010245 #0.525956 , Seriatopora_hystrix_NC_010244 #0.647921 ) #924.968 , Stylophora_pistillata_NC_011162 #0.0001 ) #987.612 ) #0.0973746 ) #0.119221 ) #0.0346914 , ((Desmophyllum_dianthus_NC_034275 #0.0001 , Desmophyllum_pertusum_NC_015143 #0.0001 ) #0.199631 , Solenosmilia_variabilis_NC_025472 #0.0001 ) #0.0412427 ) #0.0931925 , Paraconotrochus_antarcticus_NC_056275 #0.0803429 ) #0.0490381 ) #0.261773 , ((((((Antipathes_cf__dichotoma_NB_2020_MT318841 #0.0001 , Stichopathes_abyssicola_MT318856 #0.0001 ) #0.0896737 , (Phanopathes_sp__NB_2020_MT318852 #0.208939 , Stichopathes_sp__n__NB_2020_MT318857 #0.118133 ) #0.0810813 ) #0.0241096 , ((Stichopathes_luetkeni_NC_018377 #0.0001 , Stichopathes_sp__SCBUCN_8850_MZ157399 #0.0001 ) #0.0001 , Stichopathes_sp__SCBUCN_8849_MZ157400 #0.0001 ) #0.00588012 ) #0.0305451 , ((Myriopathes_japonica_NC_027667 #0.397491 , Tanacetipathes_thamnea_NC_046843 #0.0001 ) #0.0657597 , Tylopathes_sp__n__NB_2020_MT318859 #0.180301 ) #0.0327432 ) #0.0307983 , (((((((Bathypathes_sp__1_NB_2020_MT318844 #98.6063 , Bathypathes_sp__n__2_NB_2020_MT318842 #78.3274 ) #59.4239 , Stauropathes_arctica_MT318854 #67.9254 ) #0.00010005 , Bathypathes_sp__n__3_NB_2020_MT318843 #0.369014 ) #0.243073 , Stauropathes_cf__punctata_NB_2020_MT318855 #0.366349 ) #0.0001 , Telopathes_sp__NB_2020_MT318858 #0.0001 ) #0.0001 , (Dendrobathypathes_sp__n__NB_2020_MT318845 #0.0552197 , ((Parantipathes_cf__hirondelle_NB_2020_MT318849 #823.849 , Parantipathes_hirondelle_MT318850 #34.3949 ) #39.6149 , (Parantipathes_sp__NB_2020_MT318851 #39.7561 , Sibopathes_cf__macrospina_NB_2020_MT318853 #34.9617 ) #48.8295 ) #0.0583389 ) #0.0333434 ) #0.071359 , (Chrysopathes_formosa_NC_008411 #0.0001 , Trissopathes_cf__tetracrada_NB_2020_MT318840 #0.0001 ) #0.0640365 ) #0.241616 ) #0.0192028 , (Leiopathes_cf__glaberrima_NB_2020_MT318846 #0.0001 , Leiopathes_expansa_MT318847 #0.0001 ) #0.015926 ) #0.0247809 ) #0.074495 , (Epizoanthus_illoricatus_NC_046474 #0.0416142 , (((Hydrozoanthus_gracilis_NC_046400 #0.0423843 , ((Neozoanthus_aff__uchina_AP_2020_MN873592 #0.100725 , ((Zoanthus_cf__pulchellus_AP_2020_MN873599 #51.2621 , ((Zoanthus_cf__sociatus_AP_2020_MN873600 #51.2716 , Zoanthus_sociatus_NC_046476 #0.0001 ) #50.197 , Zoanthus_sp__AP_2020_MN873602 #54.3324 ) #50.6195 ) #0.0001 , Zoanthus_sansibaricus_NC_035578 #999 ) #0.0001 ) #0.0001 , (Palythoa_heliodiscus_NC_035579 #0.26316 , ((Palythoa_mizigama_NC_046403 #0.213719 , Palythoa_mutuki_NC_046404 #0.861015 ) #56.8714 , Sphenopus_marsupialis_NC_046406 #55.9037 ) #0.195947 ) #0.0001 ) #0.324868 ) #0.0303294 , ((Parazoanthus_elongatus_NC_046405 #0.161302 , Parazoanthus_swiftii_NC_046475 #999 ) #0.0001 , Savalia_savaglia_DQ825686 #0.118447 ) #0.058979 ) #0.225666 , (Microzoanthus_occultus_NC_046401 #0.0303777 , Nanozoanthus_harenaceus_NC_046402 #0.0406737 ) #0.073007 ) #0.0566923 ) #0.0351315 ) #286.207 , (((((((Actinia_equina_NC_039929 #0.0001 , Actinia_tenebrosa_NC_044902 #0.0001 ) #0.171065 , (((Anemonia_manjano_NC_037178 #0.0690612 , Heteractis_aurora_NC_047219 #0.0001 ) #0.0427952 , (Anemonia_sulcata_NC_049065 #0.0001 , Anemonia_viridis_NC_037177 #0.000100003 ) #0.0001 ) #780.816 , Anthopleura_anjunae_NC_030274 #0.210926 ) #0.0001 ) #0.0001 , Phymanthus_crucifer_NC_027614 #0.109804 ) #0.144639 , ((((Bolocera_sp__BZ_2016_KU507297 #0.117517 , (Bolocera_tuediae_NC_022470 #809.9 , Liponema_brevicorne_NC_047221 #0.0001 ) #0.0001 ) #0.149624 , (Entacmaea_quadricolor_NC_049066 #0.902119 , Isosicyonis_striata_KR051006 #0.0001 ) #0.210938 ) #750.658 , Stichodactyla_haddoni_MW760873 #0.0123629 ) #0.0001 , Epiactis_japonica_NC_047217 #0.0398635 ) #5.22456 ) #0.0001 , ((((Alicia_sansibarensis_NC_027610 #0.0799343 , (Haloclava_producta_NC_047218 #0.0001 , Sagartia_ornata_NC_027615 #0.0001 ) #0.0001 ) #0.0001 , (Diadumene_lineata_NC_045515 #0.0417827 , Metridium_senile_NC_000933 #0.0349918 ) #0.0001 ) #0.0272593 , Paraphelliactis_xishaensis_MT997141 #0.0359392 ) #0.108001 , Antholoba_achates_NC_027611 #0.0233656 ) #0.0001 ) #0.0441439 , Halcampoides_purpureus_NC_027612 #0.0341361 ) #0.04705 , Nematostella_sp__JVK_2006_NC_008164 #0.0517893 ) #0.0120433 ) #0.0280919 , (((((((Aurelia_aurita_NC_008446 #0.0363533 , (Aurelia_coerulea_NC_046792 #0.0322247 , Aurelia_limbata_NC_046691 #0.0123762 ) #0.110047 ) #0.0494787 , (Aurelia_sp__3_sensu_Dawson_et_al___2005__LC005413 #0.0426627 , Aurelia_sp__4_sensu_Dawson_et_al___2005__LC005414 #0.054613 ) #0.0875091 ) #0.0642847 , ((Nemopilema_nomurai_NC_035740 #0.0167483 , Rhopilema_esculentum_NC_035741 #0.0433013 ) #0.201077 , Stomolophus_sp__CG_2019_MK157198 #0.0295822 ) #399.976 ) #516.093 , Cassiopea_xamachana_NC_016466 #0.0737682 ) #848.961 , (Chrysaora_pacifica_NC_046775 #0.0001 , Chrysaora_quinquecirrha_NC_020459 #0.0685569 ) #0.0312274 ) #3.15012 , ((((((Blackfordia_virginica_NC_053946 #0.0269512 , Eutima_sp__BMK_2020_MW066348 #0.0273213 ) #0.118032 , Laomedea_flexuosa_JN700945 #0.0292933 ) #946.162 , (Clava_multicornis_NC_016465 #0.0573543 , (Turritopsis_dohrnii_NC_031213 #0.0153804 , Turritopsis_lata_NC_056128 #0.0214845 ) #652.063 ) #466.357 ) #652.863 , Spirocodon_saltatrix_NC_049868 #0.0492205 ) #192.629 , ((Hydra_oligactis_NC_010214 #0.0589743 , Hydra_sinensis_NC_021406 #0.117746 ) #0.100532 , Nemalecium_lighti_MZ457217 #0.195471 ) #897.429 ) #0.235583 , (Craspedacusta_sowerbii_NC_018537 #0.0209618 , Cubaia_aphrodite_NC_016467 #0.0254718 ) #146.333 ) #704.835 ) #0.0744811 , (Haliclystus_antarcticus_NC_030337 #0.016434 , OU744455_1_OU744455_1__OU744455_1 #0.0188168 ) #0.216955 ) #202.649 ) #0.0411023 , Carijoa_riisei_NC_048963 #0.0001 ) #0.00857803 , (Sarcophyton_trocheliophorum_MK994517 #0.0201538 , (((Sinularia_acuta_MW987591 #0.0001 , (Sinularia_maxima_MN485891 #0.0001 , Sinularia_penghuensis_MW256412 #0.0001 ) #0.0001 ) #0.0001 , Sinularia_cf__cruciata_GS_2016_NC_034318 #0.0001 ) #0.105199 , (Sinularia_ceramensis_NC_044122 #0.0001 , Sinularia_peculiaris_NC_018379 #0.0001 ) #0.0342483 ) #0.0553888 ) #0.0001 ) #0.0001 , (((((((Antillogorgia_bipinnata_NC_008157 #0.0326539 , ((((Eugorgia_mutabilis_NC_035665 #0.0302757 , Leptogorgia_alba_NC_035669 #0.0001 ) #0.0001 , (Leptogorgia_hebes_MN052675 #0.0369525 , Leptogorgia_virgulata_MK301586 #0.0001 ) #0.0001 ) #0.0001 , Leptogorgia_sp__USNM_1437444_KY559412 #0.148495 ) #0.0318699 , Pacifigorgia_cairnsi_NC_035668 #0.0786834 ) #0.0001 ) #0.0001 , (((Leptogorgia_capverdensis_NC_035663 #0.0001 , Leptogorgia_sarmentosa_NC_035670 #0.0001 ) #0.0560509 , Leptogorgia_gaini_KY559404 #0.0391973 ) #0.0001 , Leptogorgia_cf__palma_AP_2017_KY559406 #0.112213 ) #0.0001 ) #0.0001 , (Eunicella_albicans_NC_035666 #0.220198 , (Eunicella_cavolini_NC_035667 #0.0001 , Eunicella_verrucosa_MW588805 #0.0001 ) #0.0888062 ) #0.046352 ) #0.0001 , (Calicogorgia_granulosa_NC_023345 #0.0388863 , Euplexaura_crassa_NC_020458 #0.0531882 ) #0.175959 ) #0.0001 , Muricea_crassa_NC_029697 #0.0324795 ) #0.0001 , ((((((Dendronephthya_castanea_NC_023343 #0.0001 , Dendronephthya_mollis_NC_020456 #0.0001 ) #0.0001 , Dendronephthya_gigantea_NC_013573 #0.0001 ) #0.0001 , (Dendronephthya_putteri_NC_036022 #0.0001 , Dendronephthya_suensoni_NC_022809 #0.0001 ) #0.0001 ) #0.0276603 , Scleronephthya_gracillimum_NC_023344 #0.0867449 ) #0.0001 , (Echinogorgia_complexa_NC_020457 #0.0276802 , Paramuricea_clavata_NC_034749 #0.0359247 ) #0.0001 ) #0.118667 , Trachythela_sp__YZ_2021_MW238423 #0.145668 ) #0.0001 ) #0.0827504 , (Incrustatus_comauensis_MT254531 #0.0334168 , Telestula_humilis_MT254527 #0.0669078 ) #0.071546 ) #0.254314 ) #0.0573065 , Briareum_asbestinum_NC_008073 #0.0371629 ) #0.341524 , (Telestula_cf__batoni_AP_2020_MT254530 #0.10153 , Telestula_septentrionalis_MT254532 #0.0001 ) #0.119459 ) #0.0001 , (((Anthomastus_sp__USNM_1081145_KM015353 #0.0001 , Anthomastus_sp__USNM_1171062_KM015352 #672.472 ) #0.0332163 , (((Corallium_japonicum_AB595189 #0.0001 , Corallium_rubrum_NC_022864 #0.0001 ) #0.222196 , (Pleurocorallium_elatius_NC_022804 #0.0001 , Pleurocorallium_konojoi_NC_015406 #0.0001 ) #0.044878 ) #0.0001 , Sibogagorgia_cauliflora_NC_026193 #0.1504 ) #0.0001 ) #0.202004 , Paraminabea_aldersladei_NC_018790 #0.0794404 ) #0.0354106 ) #0.0001 , (((((((Anthoptilum_grandiflorum_NC_044086 #0.31284 , Anthoptilum_sp__1_RH_2019_MK919656 #0.622391 ) #0.0001 , (Umbellula_huxleyi_MK919668 #0.0001 , Umbellula_sp__1_RH_2019_MK919669 #0.0001 ) #0.087444 ) #0.159035 , Virgularia_mirabilis_NC_044091 #0.0931906 ) #0.101816 , (Funiculina_quadrangularis_NC_044078 #0.23239 , ((Kophobelemnon_sp__1_RH_2019_MK919660 #0.0001 , Kophobelemnon_sp__3_RH_2019_MK919661 #0.0001 ) #0.0001 , Kophobelemnon_sp__4_RH_2019_MK919662 #906.785 ) #0.0001 ) #0.0001 ) #770.818 , (Halipteris_cf__finmarchica_RH_2019_MK919659 #0.0838537 , Umbellula_sp__2_RH_2019_MK919670 #0.0001 ) #0.0001 ) #794.579 , ((((Distichoptilum_gracile_NC_044077 #0.0827959 , (Pennatula_cf__inflata_RH_2019_MK919666 #0.665671 , Pennatula_grandis_NC_044088 #785.096 ) #0.0276317 ) #0.182457 , ((Pennatula_aculeata_NC_044087 #0.0001 , Pennatula_cf__aculeata_RH_2019_MK919664 #0.0001 ) #0.0001 , Renilla_muelleri_NC_018378 #0.118956 ) #0.0764067 ) #0.0001 , Protoptilum_carpenteri_NC_044089 #0.327158 ) #0.0001 , Stylatula_elongata_NC_018380 #0.0429489 ) #0.107862 ) #0.0001 , Junceella_fragilis_NC_024181 #0.0468848 ) #0.0716547 ) #0.0772986 , Heliopora_coerulea_NC_020375 #0.0559371 ) #0.082011 , ((Callogorgia_cf__gracilis_EE_2018_MH719202 #0.0380028 , (Plumarella_adhaerans_NC_046480 #0.0001 , Plumarella_spinosa_NC_046465 #0.0001 ) #0.0518948 ) #0.0001 , Narella_hawaiiensis_NC_026192 #777.845 ) #0.177646 ) #0.181286 , Keratoisidinae_sp__BAL208_1_NC_010764 #0.437438 );

NAD4L:

**dS tree:**
[truncated: 175,088 more chars]
